# Supplementary material for: VGEA: an RNA viral assembly toolkit
Source: PeerJ. 2021 Sep 6;9:e12129. doi: 10.7717/peerj.12129 (PMC8428259; doi:10.7717/peerj.12129)
Supplement: Supplemental Information 1 [file peerj-09-12129-s001.docx]

**Supplementary File 1**

The **shiver** Method in More Detail (from *Wymant et al.,* 2018)

**shiver** is under continuing development; if at a later date description here contradicts descriptions at github.com/ChrisHIV/shiver, the latter has precedence.

**Existing References** *(HIV is used as an example)*

An alignment of existing reference sequences is required as input for shiver. Construction of a custom reference for mapping involves identifying the existing references that are closest to the sample under consideration. The greater the number and diversity of existing references given as input, the denser and broader the coverage of sequence space is, and the closer the closest reference is expected to be, with corresponding benefits for the accuracy of the results. However these existing references should be aligned to each other accurately, in order for the addition of each sample’s contigs to the alignment to be meaningful; this means that producing such an input by automatically aligning a large number of diverse sequences without checking the results would be a bad idea. This alignment will be used as input for every sample in a dataset processed by shiver, and so the user is advised to put a little thought into sequence selection and manually curating the alignment if needed.

**Constructing a Tailored Reference Using the Contigs**

Custom reference construction begins with contig preprocessing as follows. Matches between the contigs and any existing reference from the alignment are searched for using BLASTN with default settings, except for the -max target seqs 1 option (specifying that all reported hits are to a single reference only), and with -word size set to 17 (this can be changed in the configuration file). Contaminant sequence is inevitable in high-throughput NGS; any contig that has no BLASTN hit to any of the references is taken to be contamination, and is put aside for later use, leaving contigs that are putatively the organism of interest. The BLASTN results are used to correct the contigs in three ways.

1. Where a single contig has multiple BLASTN hits (discarding any hit wholly contained inside another hit), this is considered as evidence that the contig is spliced – concatenating two separated regions of the genome – due to errors in silico or during sequencing, as mentioned in the introduction. This is corrected by cutting the contig into separate contigs at the midpoint between the hits.
2. Any part of the contig that was not spanned by a BLASTN hit is trimmed off. The ends of contigs are by definition points at which the assembler has been unable to continue extending the sequence, either because of lack of reads, or because the within-sample diversity has become too great for a single, meaningful, representative sequence to be chosen. The latter possibility also means erroneous bases are more common in short stretches of sequence at the end of a contig. Trimming such sequence from the ends of contigs means the corresponding sequence from the closest existing reference will be used instead, giving a better reference for mapping. (Some assembly algorithms trim a fixed length from the ends of contigs for precisely this reason; however trimming a variable length dependent on its match to known sequence is clearly preferable.)
3. Any contig whose BLASTN hit is in the opposite orientation is reverse-complemented. If the assembler does not orientate the contigs, on average half of them will be in the reverse orientation. IVA orientates contigs such that the longest open reading frame is on the forward strand, however for very short contigs this may fail. In the process of assembling a spliced contig, an assembler may concatenate different regions in different orientations; shiver considers whether each separate part of a split contig requires reverse-complementation.

Contigs are then aligned to the existing reference alignment using MAFFT, trying both --add and --addfragments modes and using the one with the smallest maximum gap fraction (the maximum calculated over all contigs in each alignment). After alignment, a contig found to have an overly large internal deletion (by default 160bp) is split into two separate contigs at that point. This has the same role as BLASTN-based correction step 1 above, serving as a backup.

The alignment of contigs to the set of existing references should be visually inspected at this point. It is important to always verify whether the sequence data are aligned unambiguously and, if necessary, manually correct the alignment.” The commonness of indels in HIV for example makes alignment more difficult, as does the fact that the contigs may be an imperfect representation of the true sample even after correction. Geneious (*Kearse et al*., 2012) can be used for sequence visualisation and editing where needed.

As well as revealing alignment error, inspection of the aligned contigs allows the user to check for any remaining problems with the contigs. It is suggested that in general the user inspects both the alignment of the existing references with the raw contigs of the organism of interest (before any correction by shiver), and the alignment of the existing references with the corrected contigs, as a check that all shiver’s modifications of the contigs are desired. An example of when this might not be the case is when the sample contains an indel not observed in the existing reference set, that is large enough to cause the contig to be split in two at that point, but which the user thinks might be genuine rather than an a misassembly (through previous/expert knowledge, or perhaps simply observing the same indel in multiple samples in a dataset). With sufficiently accurate mapping, reads will map here correctly whether or not the reference constructed from the contigs contains the indel, making the question moot; however with mapping inaccuracies possible, it’s best to get the reference’s structure as correct as possible before mapping.

Using the alignment of contigs to existing references, the set of contigs is flattened into a single sequence as follows. At positions covered by one contig, its base (or gap character, for a deletion) is used. At positions covered by multiple contigs, whatever the longest contig has (be it base or gap) is used. This heuristic was used expecting that, where sufficiently distinct haplotypes exist to result in multiple contigs covering the same place, haplotypes supported by a higher depth of reads would tend to be assembled into longer contigs. The sequence resulting from this flattening of the contigs is compared to each existing reference in the alignment in turn: we count towards similarity shared bases and gaps within contigs (known deletions), but not gaps between contigs (missing information). The existing references are ranked by their similarity to the contigs. As existing references have variable lengths (the long terminal repeat regions that flank the clinical genome are sometimes sequenced only partially or not at all), the closest reference is extended outwards using any overhanging sequence from the second closest reference, then the third longest sequence etc. terminating when both edges of the alignment are reached. This sequence – the elongated closest reference – is used to fill in any gaps between (but not inside of) the flattened contigs. **This completes production of the reference tailored for this sample**.

**Preparing and Mapping the Reads**

Before mapping to this reference, the reads are trimmed and cleaned as follows. Adapters, primers and low quality bases are trimmed using Trimmomatic and Fastaq. Contaminant reads from non-HIV sources are then considered. Most of these would presumably be discarded by mapping to an HIV reference, due to lack of similarity. However there is ample opportunity for traces of human DNA to end up in a sample, and sequence of endogenous retroviruses in human DNA may resemble HIV. As a guard against this, and against any other contamination resembling HIV, BLASTN is used to find all read pairs that are a better match to one of the contigs previously found to be contamination, than to the tailored reference. These pairs are discarded.

The cleaned reads are mapped to the tailored reference, using SMALT by default (with BWA and bowtie as optional alternatives), giving a file in BAM format. Using SAMtools the BAM file is read into pileup format, which is parsed to give base frequencies at each position in the genome. Note that within-host diversity does not consist exclusively of point mutations: indels can be present in some reads and not others, which must be dealt with in the pileup. Where some reads have a deletion relative to the reference and others do not, the deletion/gap character can simply be considered as a fifth base whose frequency can be counted like the others. Where some reads have an insertion relative to the reference and others do not, or more generally where insertions of two or more sizes are present, the most common insertion size is found and, inside that insertion, only those reads with an insertion of that size is considered (thus avoiding any ambiguity in the alignment of the inserted sequences to each other). Finally, the base frequency file is parsed to call the consensus base at each position. By default the most common base is called to give the consensus, using an ambiguity code only for an exact tie in the frequency of two or more bases; optionally ambiguity codes can be used more readily, when the frequency of the most common base or bases is below a specified threshold. A consensus base is only called if the coverage equals or exceeds a minimum threshold specified by the user, to protect against the effect of residual low-coverage contaminant reads in genomic regions lacking genuine reads of the organism of interest. By default this is 15, but this is likely to need adjusting for different datasets.

**Aligning Multiple Consensuses**

Since we know how the consensus aligns to the reference used for mapping, and we know how that reference (constructed from the contigs) aligns to the input alignment of existing references, we can construct a global alignment of the consensuses from all samples merely by coordinate translation, negating the need for further alignment and manual curation. Two things must be excised from the consensus for this global alignment reconstruction: insertions present in the majority of reads but not in their tailored reference (which are rare, since the reference is constructed from the contigs which are constructed from the reads), and insertions present in the contigs but none of the existing references (which are rare provided the set of existing references is large and diverse). In both cases this is sequence whose alignment to the common anchor of the existing references is not known, and so coordinate translation cannot align it.
