# Supplementary material for: VGEA: an RNA viral assembly toolkit
Source: PeerJ. 2021 Sep 6;9:e12129. doi: 10.7717/peerj.12129 (PMC8428259; doi:10.7717/peerj.12129)
Supplement: Supplemental Information 2 [file peerj-09-12129-s002.zip › Supplementary_File_2/Performance_Evaluation_Data_for_Table_1/multiqc/multiqc_report.html]

MultiQC Report


# Toggle navigation v1.10.1 (eef03df)

Loading report..

- General Stats
- QUAST
  - Assembly Statistics
  - Number of Contigs
- Samtools
- fastp
  - Filtered Reads
  - Duplication Rates
  - Insert Sizes
  - Sequence Quality
  - GC Content
  - N content

Toolbox

### MultiQC Toolbox

#### Apply Highlight Samples

+

Regex mode off
help
 Clear

#### Apply Rename Samples

+

Click here for bulk input.

Paste two columns of a tab-delimited table here (eg. from Excel).

First column should be the old name, second column the new name.

Add

Regex mode off
help
 Clear

#### Apply Show / Hide Samples

Hide matching samples

Show only matching samples

+

Regex mode off
help
 Clear

#### Export Plots

- Images
- Data

px

px

Aspect ratio

PNG
JPEG
SVG

Plot scaling

X

Download the raw data used to create the plots in this report below:

Format:

Tab-separated
Comma-separated
JSON

Note that additional data was saved in `multiqc_data` when this report was generated.

---

##### Choose Plots

 All
 None

---


   Download Plot Images

If you use plots from MultiQC in a publication or presentation, please cite:

> **MultiQC: Summarize analysis results for multiple tools and samples in a single report**  
> *Philip Ewels, Måns Magnusson, Sverker Lundin and Max Käller*  
> Bioinformatics (2016)  
> doi: 10.1093/bioinformatics/btw354  
> PMID: 27312411

#### Save Settings

You can save the toolbox settings for this report to the browser.

 Save


---

#### Load Settings

Choose a saved report profile from the dropdown box below:

[ select ]

Load
 Delete
 Set default
 Clear default

#### About MultiQC

This report was generated using MultiQC, version 1.10.1 (eef03df)

You can see a YouTube video describing how to use MultiQC reports here:
https://youtu.be/qPbIlO\_KWN0

For more information about MultiQC, including other videos and
extensive documentation, please visit http://multiqc.info

You can report bugs, suggest improvements and find the source code for MultiQC on GitHub:
https://github.com/ewels/MultiQC

MultiQC is published in Bioinformatics:

> **MultiQC: Summarize analysis results for multiple tools and samples in a single report**  
> *Philip Ewels, Måns Magnusson, Sverker Lundin and Max Käller*  
> Bioinformatics (2016)  
> doi: 10.1093/bioinformatics/btw354  
> PMID: 27312411

# 

A modular tool to aggregate results from bioinformatics analyses across many samples into a single report.

#### JavaScript Disabled

MultiQC reports use JavaScript for plots and toolbox functions. It looks like
you have JavaScript disabled in your web browser. Please note that many of the report
functions will not work as intended.

Loading report..

Report
generated on 2021-05-18, 21:36
based on data in:
`/mnt/c/Users/paule/Documents/VGEA/results`

---

×
don't show again

**Welcome!** Not sure where to start?  
Watch a tutorial video
  *(6:06)*

## General Statistics

 Copy table

 Configure Columns

 Sort by highlight

 Plot
Showing 2/2 rows and 7/10 columns.

| Sample Name | M Reads | M Reads Mapped | N50 (Kbp) | Length (Mbp) | % Duplication | % > Q30 | Mb Q30 bases | GC content | % PF | % Adapter |
| --- | --- | --- | --- | --- | --- | --- | --- | --- | --- | --- |
| CV18 | 2.9 | 0.0 | 29.9Kbp | 0.0Mbp | 38.5% | 97.3% | 320.4 | 50.2% | 99.7% | 2.2% |
| CV29 | 1.5 | 0.0 | 30.0Kbp | 0.0Mbp | 30.9% | 96.9% | 175.4 | 45.7% | 99.3% | 0.3% |

×

#### General Statistics: Columns

Uncheck the tick box to hide columns. Click and drag the handle on the left to change order.

Show All
Show None

| Sort | Visible | Group | Column | Description | ID | Scale |
| --- | --- | --- | --- | --- | --- | --- |
| || |  | Samtools | M Reads | Total reads in the bam file (millions) | `flagstat_total` | read\_count |
| || |  | Samtools | M Reads Mapped | Reads Mapped in the bam file (millions) | `mapped_passed` | read\_count |
| || |  | QUAST | N50 (Kbp) | N50 is the contig length such that using longer or equal length contigs produces half (50%) of the bases of the assembly (kilo base pairs) | `N50` | None |
| || |  | QUAST | Length (Mbp) | The total number of bases in the assembly (mega base pairs). | `Total length` | None |
| || |  | fastp | % Duplication | Duplication rate before filtering | `pct_duplication` | None |
| || |  | fastp | % > Q30 | Percentage of reads > Q30 after filtering | `after_filtering_q30_rate` | None |
| || |  | fastp | Mb Q30 bases | Bases > Q30 after filtering (millions) | `after_filtering_q30_bases` | base\_count |
| || |  | fastp | GC content | GC content after filtering | `after_filtering_gc_content` | None |
| || |  | fastp | % PF | Percent reads passing filter | `pct_surviving` | None |
| || |  | fastp | % Adapter | Percentage adapter-trimmed reads | `pct_adapter` | None |

Close

## QUAST

QUAST is a quality assessment tool for genome assemblies, written by the Center for Algorithmic Biotechnology.

### Assembly Statistics

Copy table

 Configure Columns

 Sort by highlight

 Plot
Showing 2/2 rows and 10/10 columns.

| Sample Name | N50 (Kbp) | N75 (Kbp) | L50 (K) | L75 (K) | Largest contig (Kbp) | Length (Mbp) | Misassemblies | Mismatches/100kbp | Indels/100kbp | Genome Fraction |
| --- | --- | --- | --- | --- | --- | --- | --- | --- | --- | --- |
| CV18 | 29.9Kbp | 29.9Kbp | 0.0K | 1.0K | 29.9Kbp | 0.0Mbp | 0.0 | 33.52 | 0.00 | 99.8% |
| CV29 | 30.0Kbp | 30.0Kbp | 0.0K | 1.0K | 30.0Kbp | 0.0Mbp | 0.0 | 23.59 | 0.00 | 99.2% |

×

#### Quast Table: Columns

Uncheck the tick box to hide columns. Click and drag the handle on the left to change order.

Show All
Show None

| Sort | Visible | Group | Column | Description | ID | Scale |
| --- | --- | --- | --- | --- | --- | --- |
| || |  | QUAST | N50 (Kbp) | N50 is the contig length such that using longer or equal length contigs produces half (50%) of the bases of the assembly. | `N50` | None |
| || |  | QUAST | N75 (Kbp) | N75 is the contig length such that using longer or equal length contigs produces 75% of the bases of the assembly | `N75` | None |
| || |  | QUAST | L50 (K) | L50 is the number of contigs larger than N50, i.e. the minimum number of contigs comprising 50% of the total assembly length. | `L50` | None |
| || |  | QUAST | L75 (K) | L75 is the number of contigs larger than N75, i.e. the minimum number of contigs comprising 75% of the total assembly length. | `L75` | None |
| || |  | QUAST | Largest contig (Kbp) | The size of the largest contig of the assembly | `Largest contig` | None |
| || |  | QUAST | Length (Mbp) | The total number of bases in the assembly. | `Total length` | None |
| || |  | QUAST | Misassemblies | The number of positions in the assembled contigs where the left flanking sequence aligns over 1 kbp away from the right flanking sequence on the reference (relocation) or they overlap on more than 1 kbp (relocation) or flanking sequences align on different strands (inversion) or different chromosomes (translocation). | `# misassemblies` | None |
| || |  | QUAST | Mismatches/100kbp | The number of mismatches per 100 kbp | `# mismatches per 100 kbp` | None |
| || |  | QUAST | Indels/100kbp | The number of indels per 100 kbp | `# indels per 100 kbp` | None |
| || |  | QUAST | Genome Fraction | The total number of aligned bases in the reference, divided by the genome size. | `Genome fraction (%)` | None |

Close

---

### Number of Contigs

This plot shows the number of contigs found for each assembly, broken
down by length.

Counts
Percentages

loading..

---

## Samtools

Samtools is a suite of programs for interacting with high-throughput sequencing data.

### Samtools Flagstat

This module parses the output from `samtools flagstat`. All numbers in millions.

loading..

---

## fastp

fastp An ultra-fast all-in-one FASTQ preprocessor (QC, adapters, trimming, filtering, splitting...)

### Filtered Reads

Filtering statistics of sampled reads.

Number of Reads
Percentages

loading..

---

### Duplication Rates

Duplication rates of sampled reads.

loading..

---

### Insert Sizes

Insert size estimation of sampled reads.

loading..

---

### Sequence Quality

Average sequencing quality over each base of all reads.

Read 1: Before filtering
Read 1: After filtering
Read 2: Before filtering
Read 2: After filtering

loading..

---

### GC Content

Average GC content over each base of all reads.

Read 1: Before filtering
Read 1: After filtering
Read 2: Before filtering
Read 2: After filtering

loading..

---

### N content

Average N content over each base of all reads.

Read 1: Before filtering
Read 1: After filtering
Read 2: Before filtering
Read 2: After filtering

loading..

**MultiQC v1.10.1 (eef03df)**
- Written by Phil Ewels,
available on GitHub.

This report uses HighCharts,
jQuery,
jQuery UI,
Bootstrap,
FileSaver.js and
clipboard.js.

×

### Plot Table Data

Select Column

Select Column

Please select two table columns.

Close

×

### Regex Help

Toolbox search strings can behave as regular expressions (regexes). Click a button below to see an example of it in action. Try modifying them yourself in the text box.

`^` (start of string)
`$` (end of string)
`[]` (character choice)
`\d` (shorthand for `[0-9]`)
`\w` (shorthand for `[0-9a-zA-Z_]`)
`.` (any character)
`\.` (literal full stop)
`()` `|` (group / separator)
`*` (prev char 0 or more)
`+` (prev char 1 or more)
`?` (prev char 0 or 1)
`{}` (char num times)
`{,}` (count range)

```
samp_1
samp_1_edited
samp_2
samp_2_edited
samp_3
samp_3_edited
prepended_samp_1
tmp_samp_1_edited
tmpp_samp_1_edited
tmppp_samp_1_edited
#samp_1_edited.tmp
samp_11
samp_11111
```

See regex101.com for a more heavy duty testing suite.

Close
