# Supplementary material for: VGEA: an RNA viral assembly toolkit
Source: PeerJ. 2021 Sep 6;9:e12129. doi: 10.7717/peerj.12129 (PMC8428259; doi:10.7717/peerj.12129)
Supplement: Supplemental Information 2 [file peerj-09-12129-s002.zip › Supplementary_File_2/Performance_Comparison_Assembly_Pipelines_Table_2/CV115/vgea/quast_results/results_2021_05_13_23_06_24/report.pdf]

Report

	CV115_contigs
# contigs (>= 0 bp)	28
# contigs (>= 1000 bp)	15
# contigs (>= 5000 bp)	1
# contigs (>= 10000 bp)	0
# contigs (>= 25000 bp)	0
# contigs (>= 50000 bp)	0
Total length (>= 0 bp)	44340
Total length (>= 1000 bp)	33480
Total length (>= 5000 bp)	5225
Total length (>= 10000 bp)	0
Total length (>= 25000 bp)	0
Total length (>= 50000 bp)	0
# contigs	28
Largest contig	5225
Total length	44340
Reference length	29903
GC (%)	43.55
Reference GC (%)	37.97
N50	2258
NG50	3060
N75	1013
NG75	2258
L50	7
LG50	4
L75	15
LG75	7
# misassemblies	0
# misassembled contigs	0
Misassembled contigs length	0
# local misassemblies	0
# scaffold gap ext. mis.	0
# scaffold gap loc. mis.	0
# unaligned mis. contigs	0
# unaligned contigs	16 + 0 part
Unaligned length	14595
Genome fraction (%)	96.957
Duplication ratio	1.026
# N's per 100 kbp	0.00
# mismatches per 100 kbp	41.39
# indels per 100 kbp	0.00
# genomic features	38 + 21 part
Largest alignment	5152
Total aligned length	29438
NA50	2179
NGA50	3060
NGA75	2179
LA50	7
LGA50	4
LGA75	7

All statistics are based on contigs of size >= 500 bp, unless otherwise noted (e.g., "# contigs (>= 0 bp)" and "Total length (>= 0 bp)" include all contigs).

Misassemblies report

	CV115_contigs
# misassemblies	0
# contig misassemblies	0
# c. relocations	0
# c. translocations	0
# c. inversions	0
# scaffold misassemblies	0
# s. relocations	0
# s. translocations	0
# s. inversions	0
# misassembled contigs	0
Misassembled contigs length	0
# local misassemblies	0
# scaffold gap ext. mis.	0
# scaffold gap loc. mis.	0
# unaligned mis. contigs	0
# mismatches	12
# indels	0
# indels (<= 5 bp)	0
# indels (> 5 bp)	0
Indels length	0

All statistics are based on contigs of size >= 500 bp, unless otherwise noted (e.g., "# contigs (>= 0 bp)" and "Total length (>= 0 bp)" include all contigs).

Unaligned report

	CV115_contigs
# fully unaligned contigs	16
Fully unaligned length	14595
# partially unaligned contigs	0
Partially unaligned length	0
# N's	0

All statistics are based on contigs of size >= 500 bp, unless otherwise noted
(e.g., "# contigs (>= 0 bp)" and "Total length (>= 0 bp)" include all contigs).

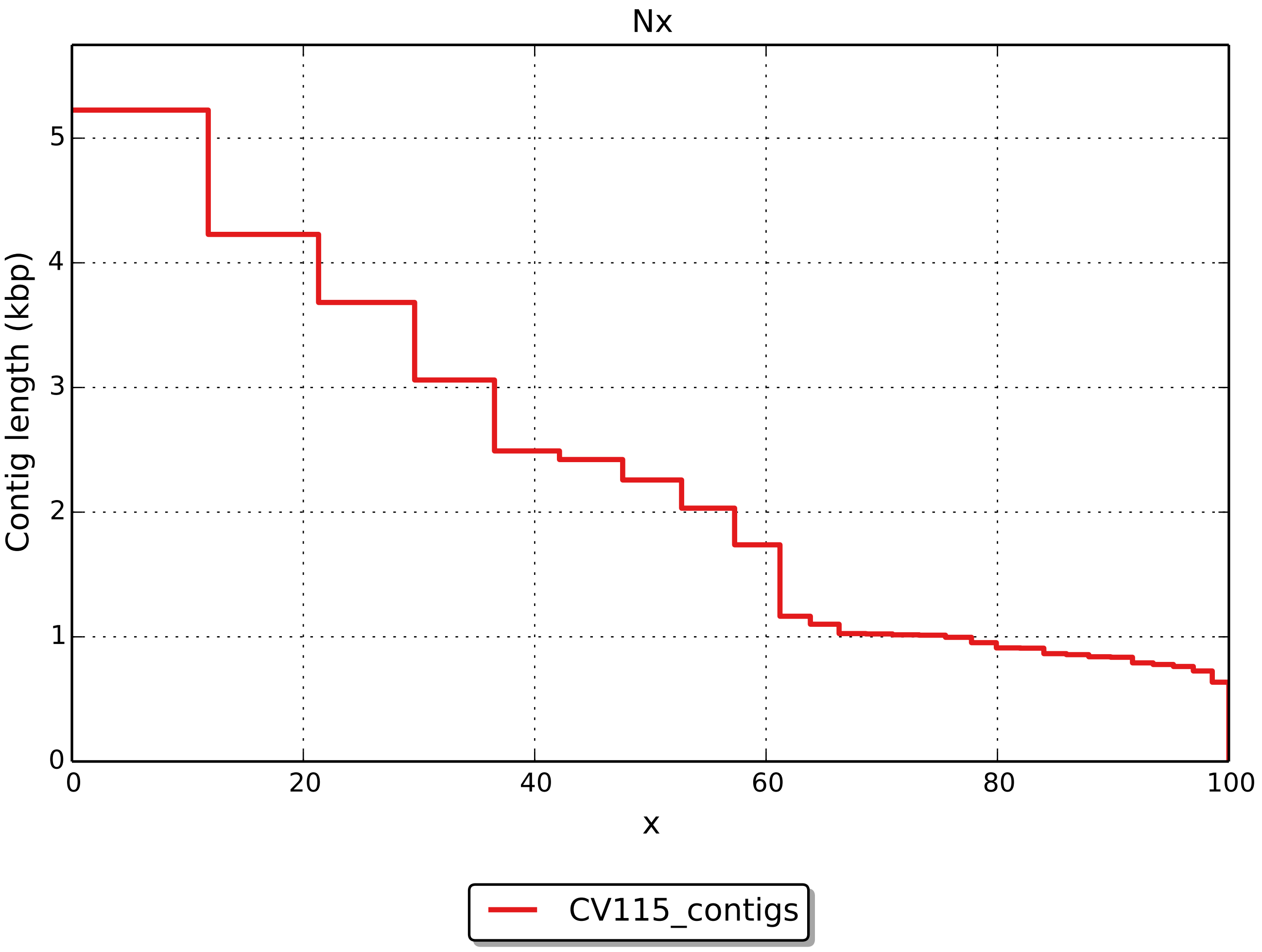

Nx
Contig length (kbp)
0
1
2
3
4
5
0
20
40
60
80
100
x
CV115_contigs

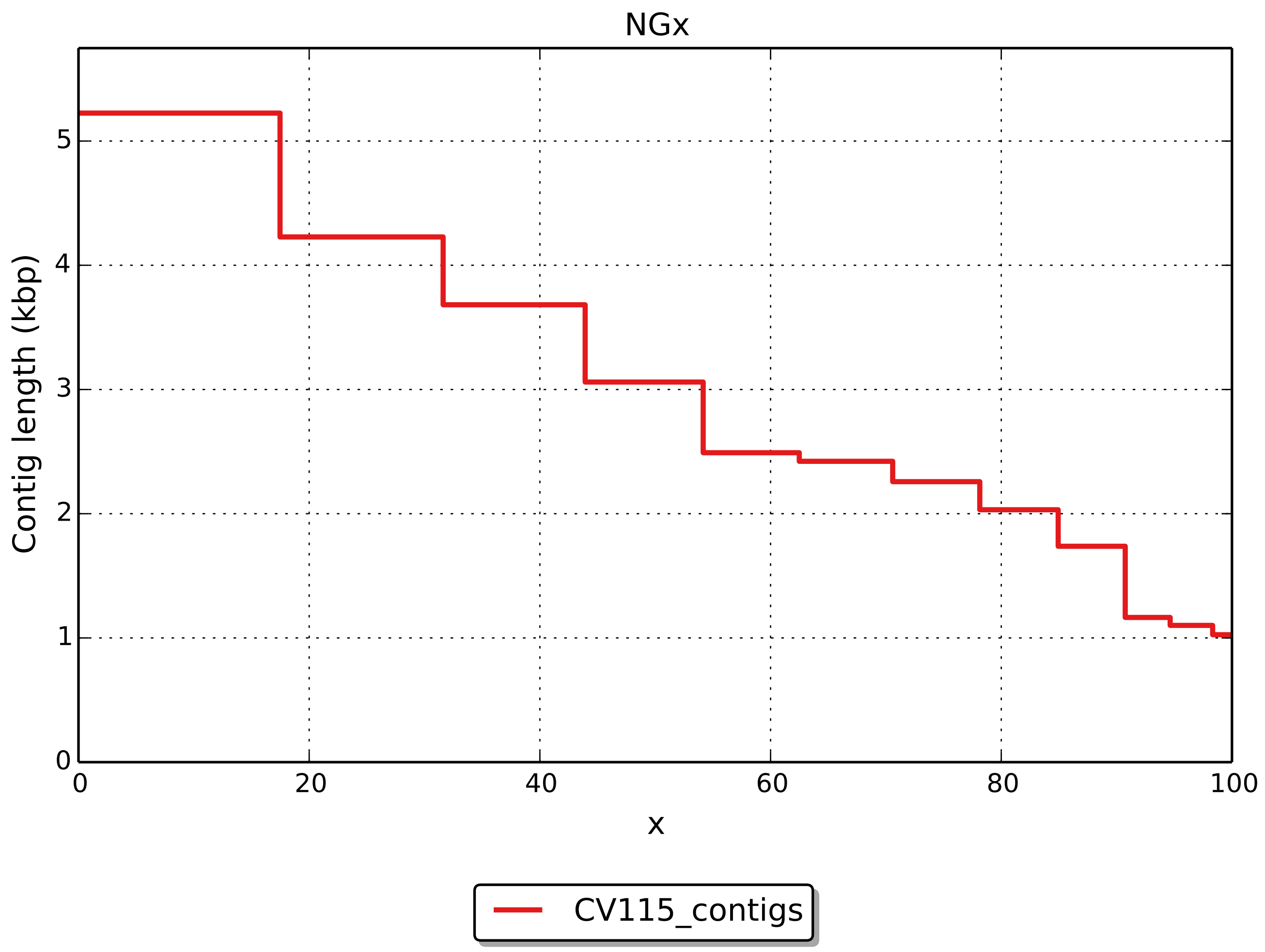
NGx
Contig length (kbp)
0
1
2
3
4
5
0
20
40
60
80
100
x
CV115_contigs

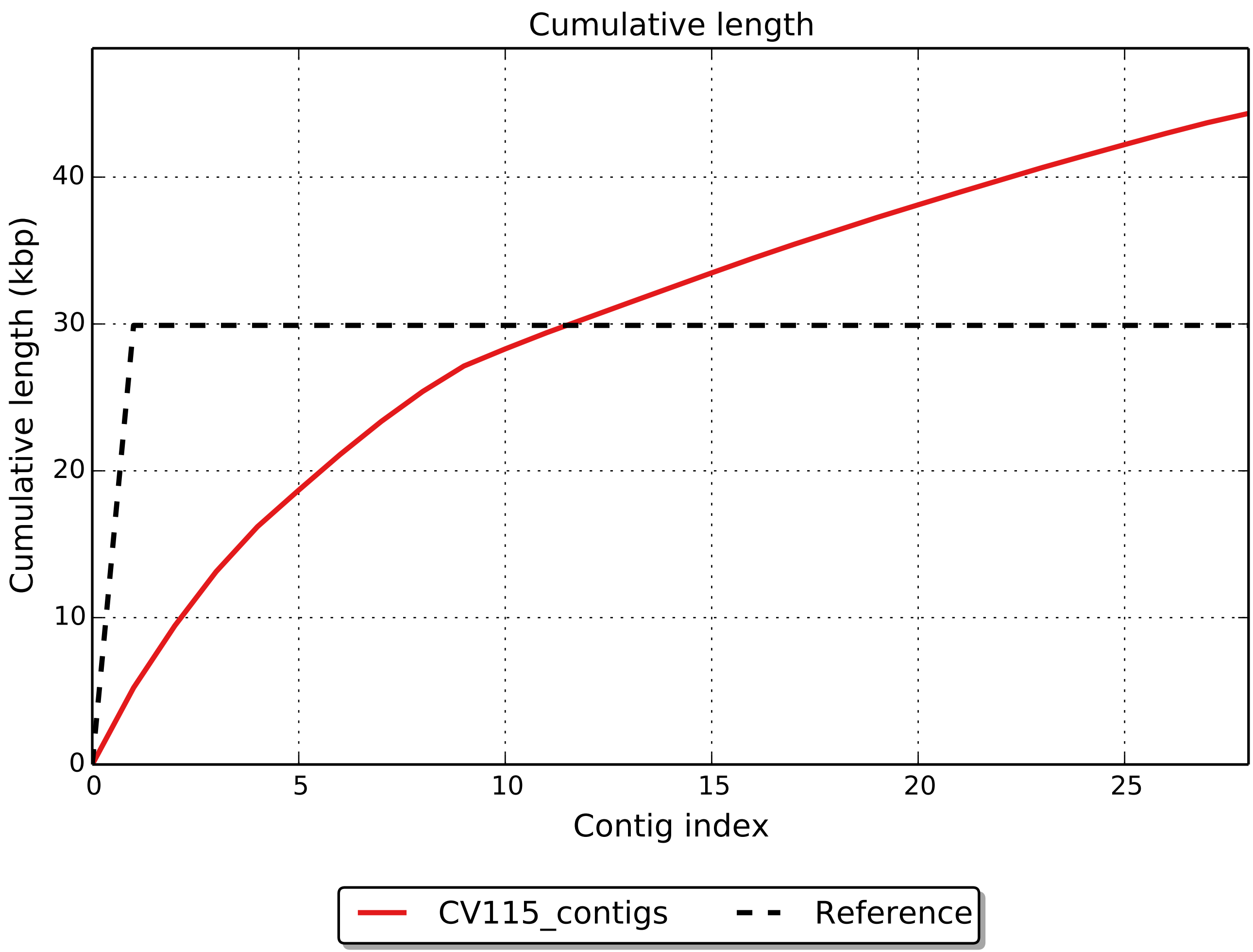

Cumulative length
Cumulative length (kbp)
0
10
20
30
40
0
5
10
15
20
25
Contig index
CV115_contigs
Reference

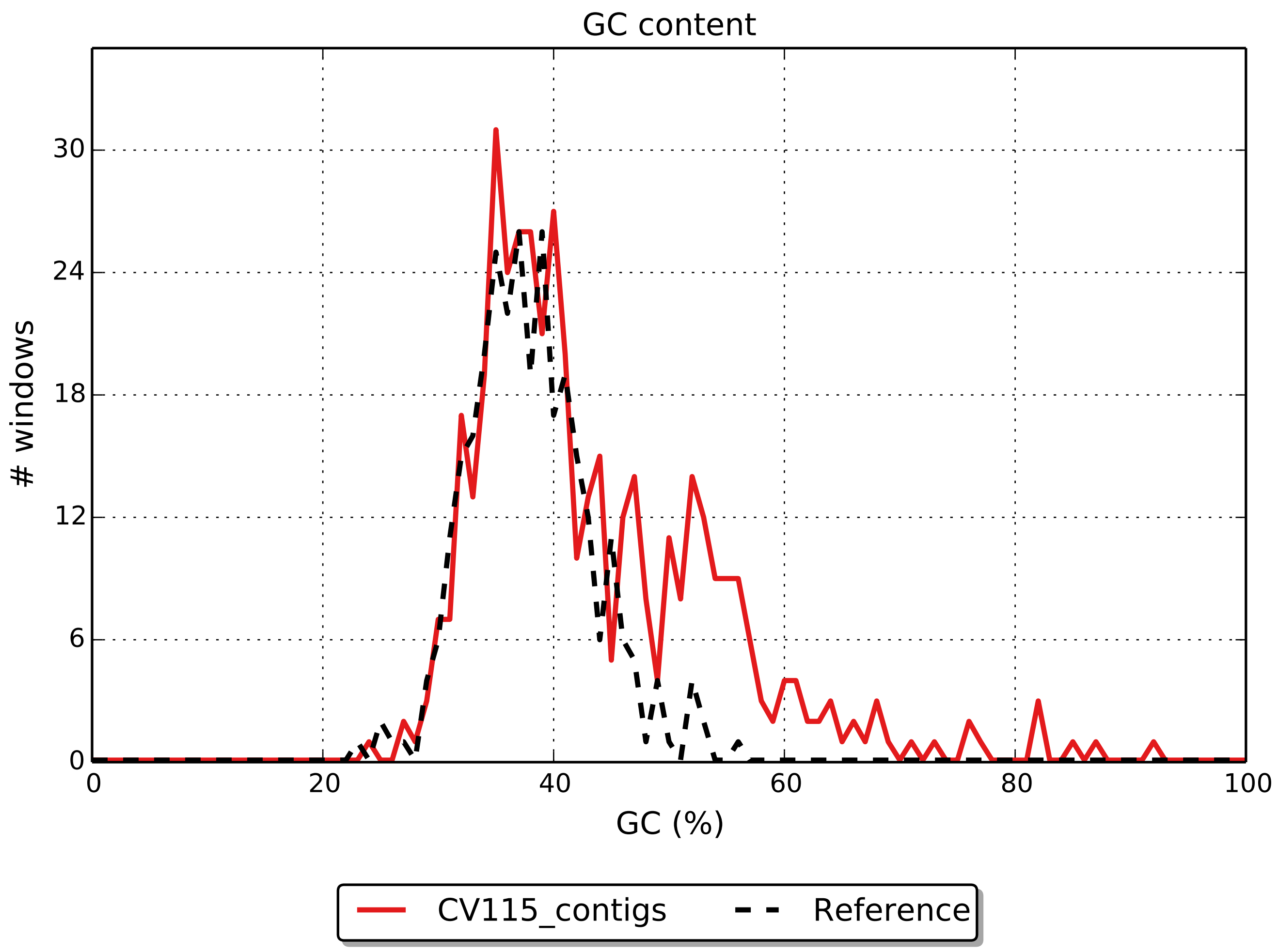

GC content
windows
0
6
12
18
24
30
0
20
40
60
80
100
GC (%)
CV115_contigs
Reference

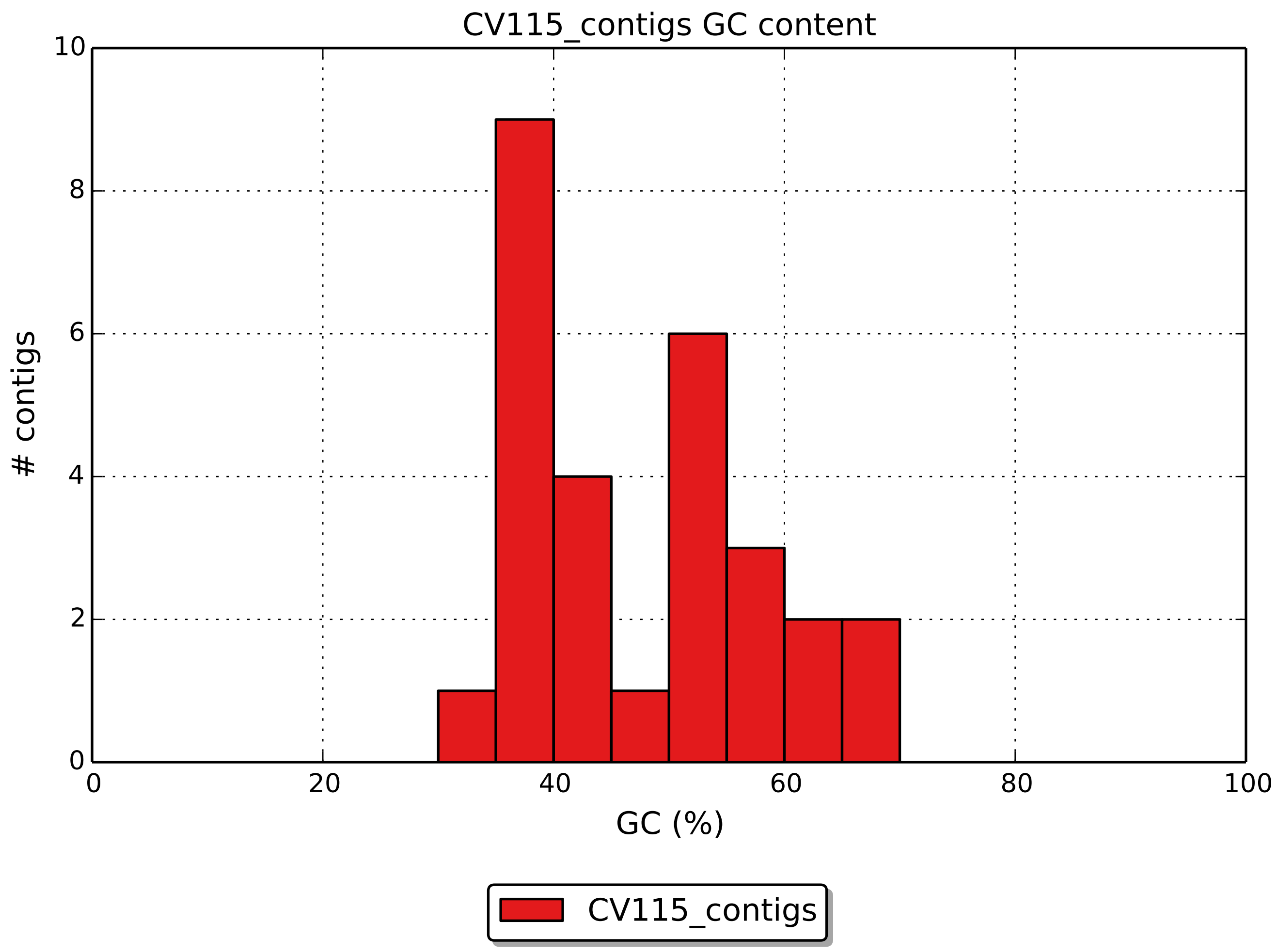
CV115_contigs GC content
contigs
GC (%)
0
2
4
6
8
10
0
20
40
60
80
100
CV115_contigs

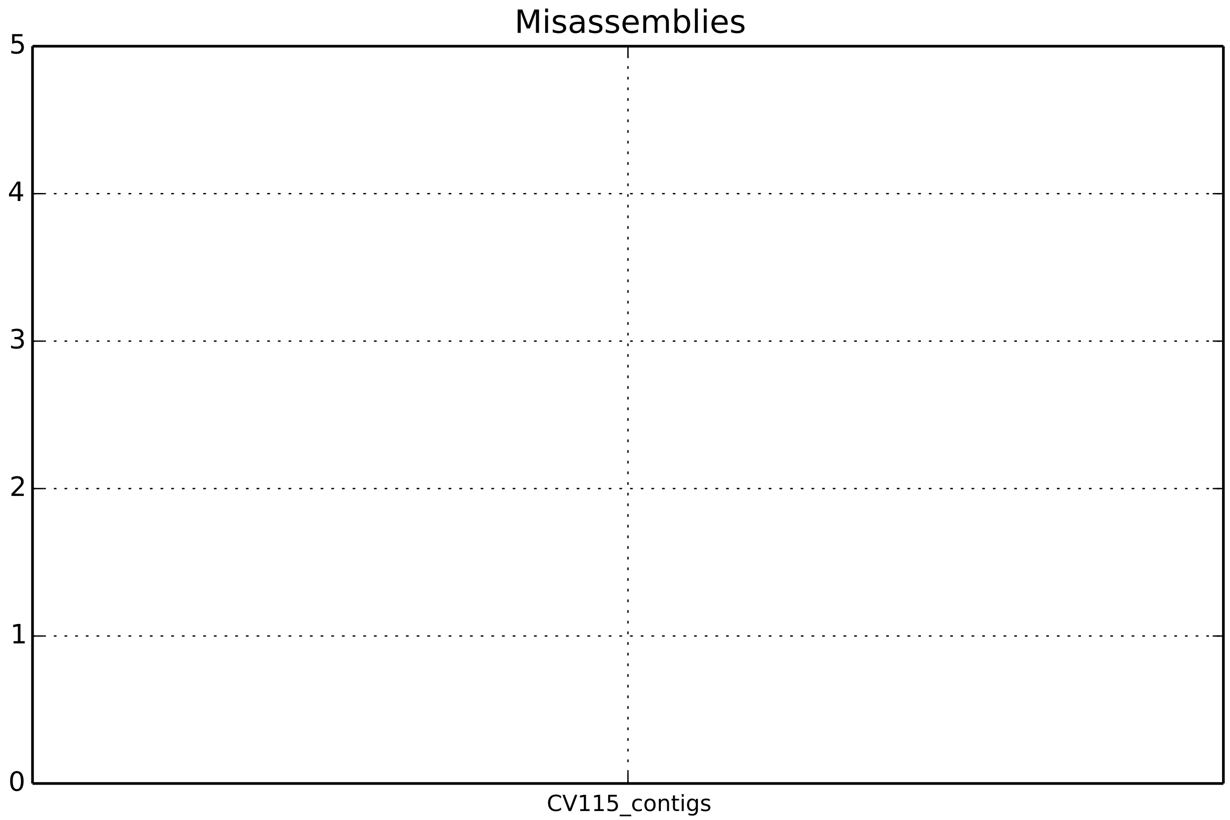
Misassemblies
5
4
3
2
1
0
CV115_contigs

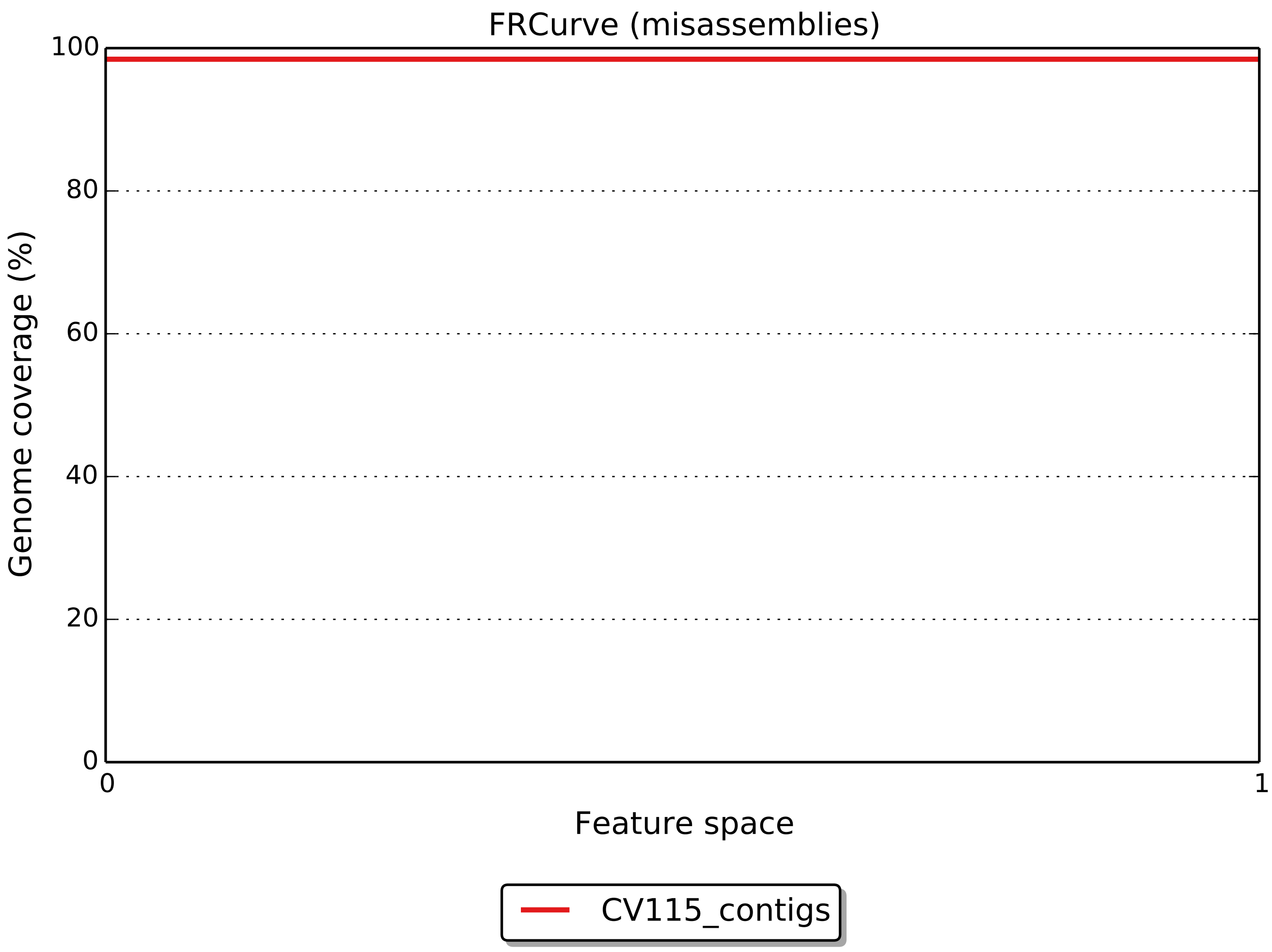
FRCurve (misassemblies)
Genome coverage (%)
100
80
60
40
20
0
0
1
Feature space
CV115_contigs

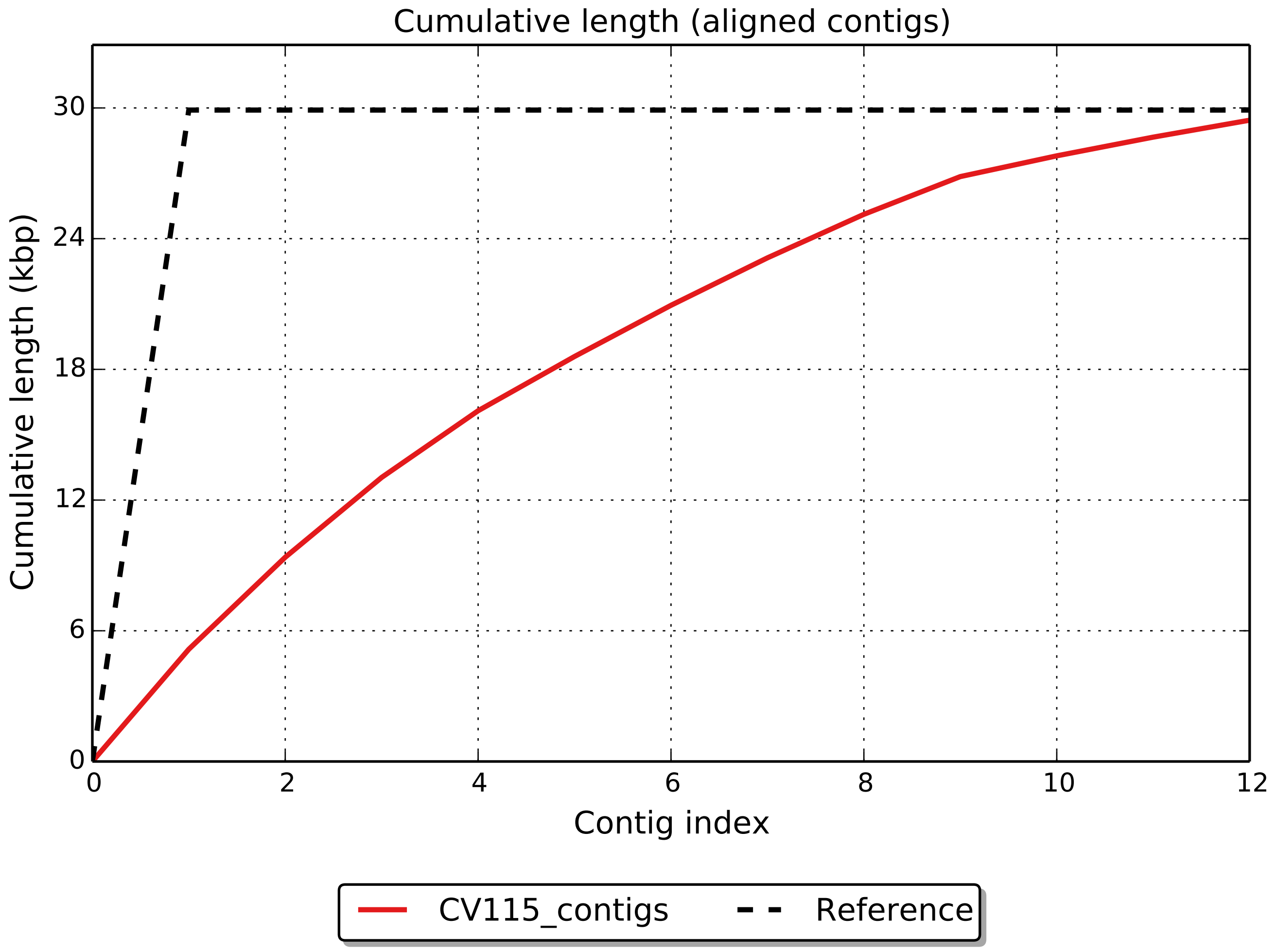
Cumulative length (aligned contigs)
Cumulative length (kbp)
0
6
12
18
24
30
0
2
4
6
8
10
12
Contig index
CV115_contigs
Reference

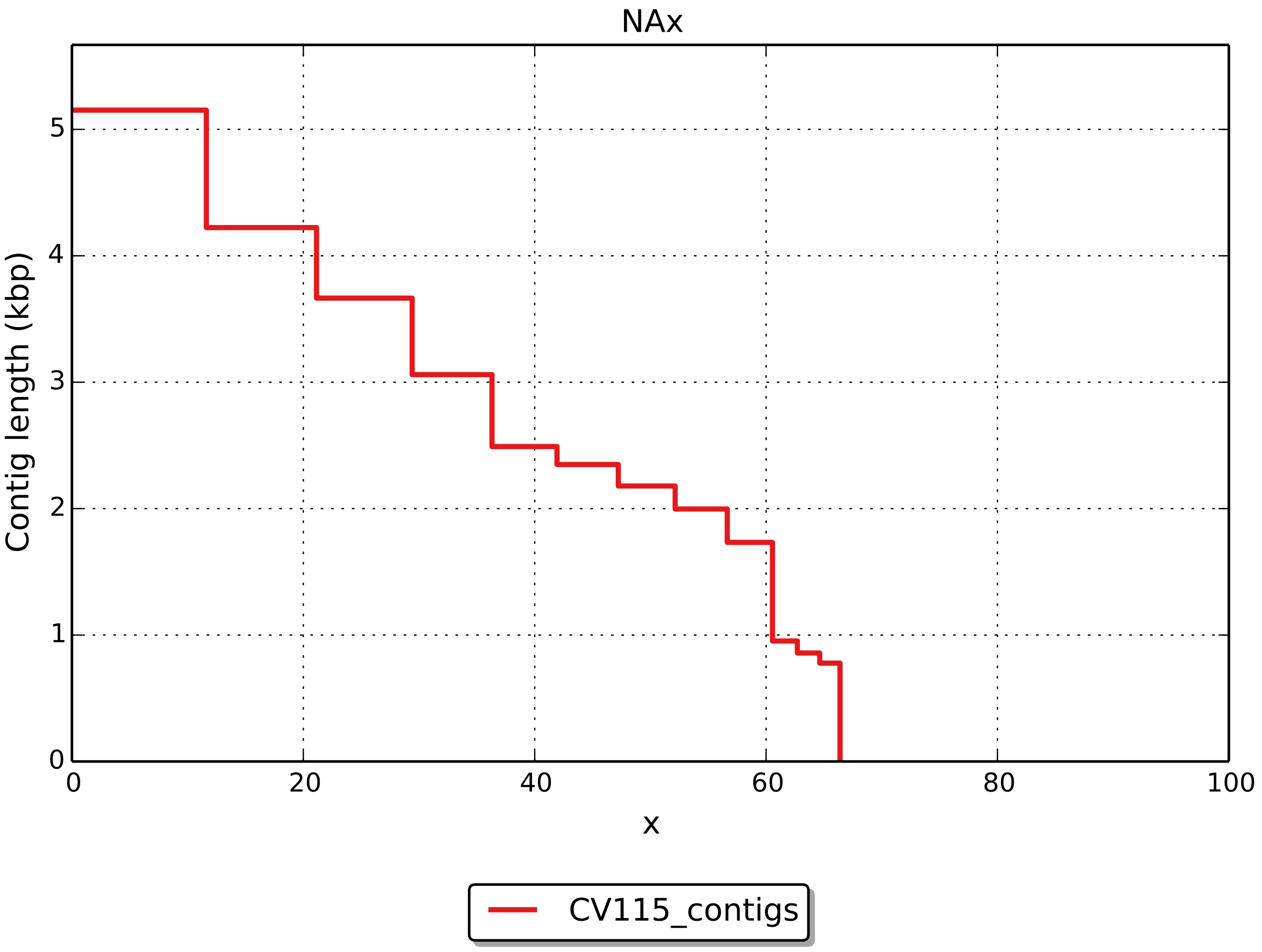
NAx
Contig length (kbp)
0
1
2
3
4
5
0
20
40
60
80
100
x
CV115_contigs

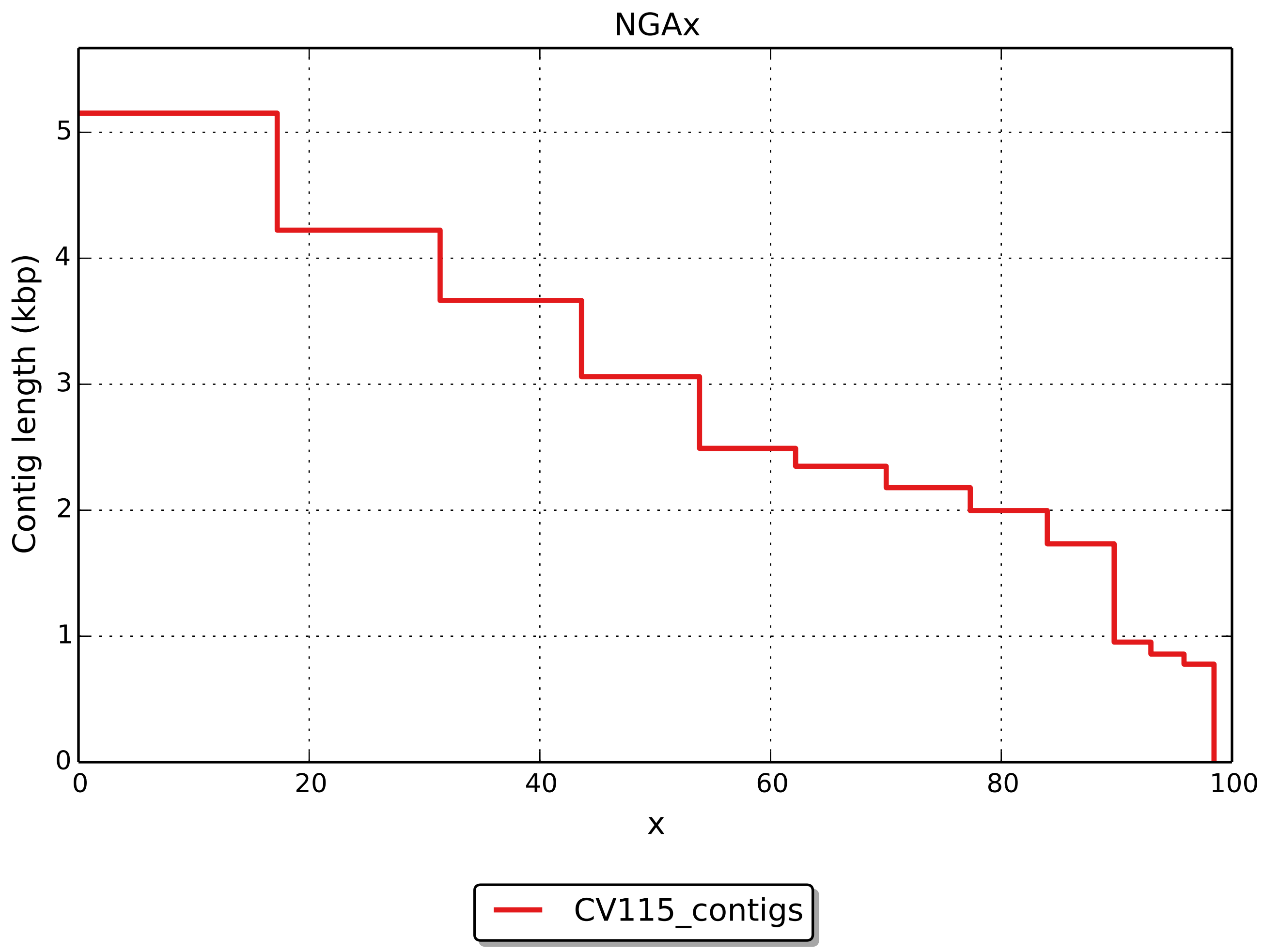
NGAx
Contig length (kbp)
0
1
2
3
4
5
0
20
40
60
80
100
x
CV115_contigs

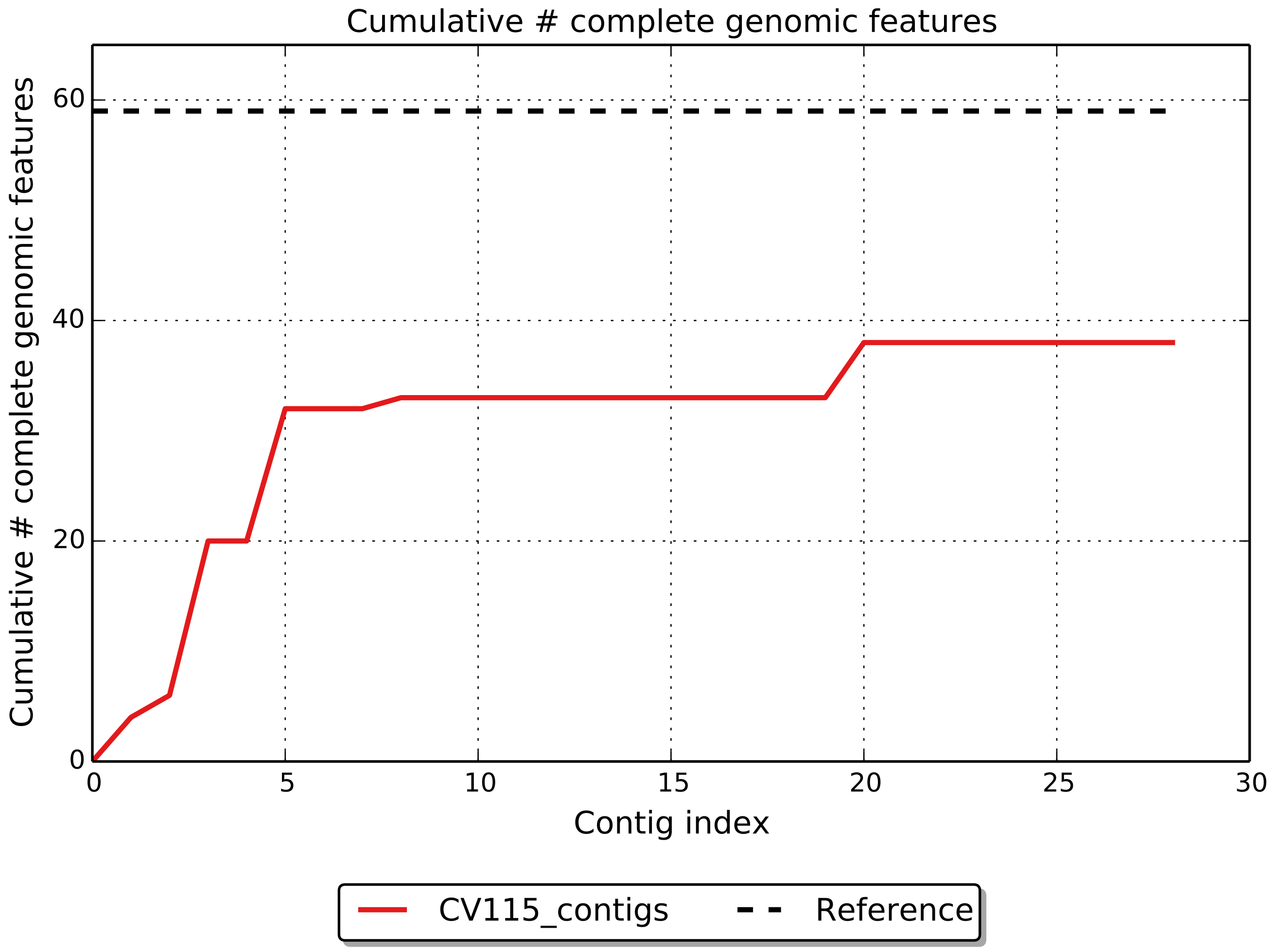

Cumulative # complete genomic features
Cumulative # complete genomic features
0
20
40
60
0
5
10
15
20
25
30
Contig index
CV115_contigs
Reference

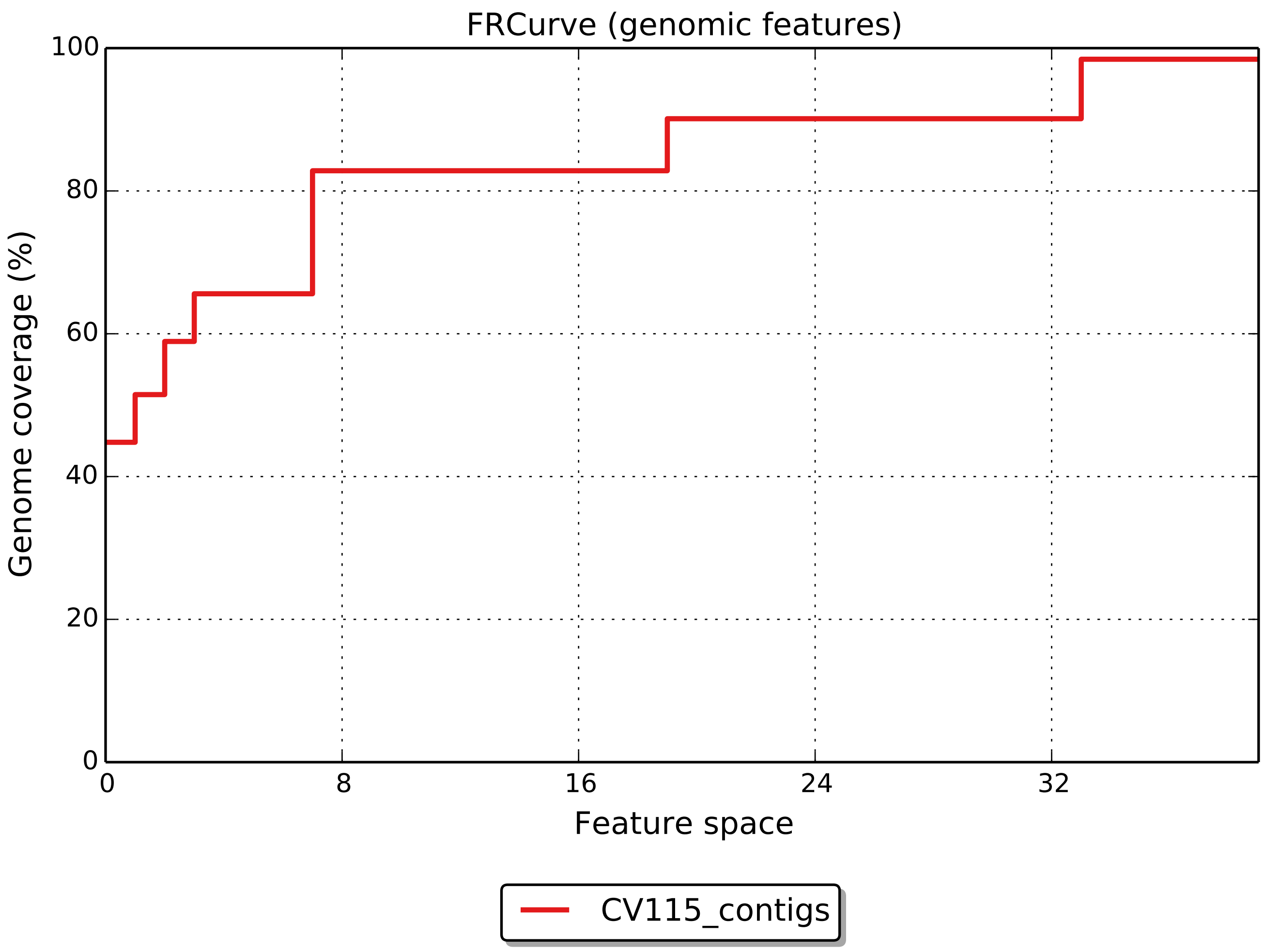
FRCurve (genomic features)
Genome coverage (%)
0
20
40
60
80
100
0
8
16
24
32
Feature space
CV115_contigs
