# Supplementary material for: VGEA: an RNA viral assembly toolkit
Source: PeerJ. 2021 Sep 6;9:e12129. doi: 10.7717/peerj.12129 (PMC8428259; doi:10.7717/peerj.12129)
Supplement: Supplemental Information 2 [file peerj-09-12129-s002.zip › Supplementary_File_2/Performance_Comparison_Assembly_Pipelines_Table_2/CV29/velvet/quast_results/results_2021_05_14_16_35_58/report.pdf]

Report

	CV29_velvet_contigs
# contigs (>= 0 bp)	541
# contigs (>= 1000 bp)	16
# contigs (>= 5000 bp)	0
# contigs (>= 10000 bp)	0
# contigs (>= 25000 bp)	0
# contigs (>= 50000 bp)	0
Total length (>= 0 bp)	163765
Total length (>= 1000 bp)	24443
Total length (>= 5000 bp)	0
Total length (>= 10000 bp)	0
Total length (>= 25000 bp)	0
Total length (>= 50000 bp)	0
# contigs	66
Largest contig	2877
Total length	59242
Reference length	29903
GC (%)	46.83
Reference GC (%)	37.97
N50	942
NG50	1380
N75	680
NG75	1093
L50	22
LG50	9
L75	41
LG75	15
# misassemblies	0
# misassembled contigs	0
Misassembled contigs length	0
# local misassemblies	0
# scaffold gap ext. mis.	0
# scaffold gap loc. mis.	0
# unaligned mis. contigs	0
# unaligned contigs	65 + 0 part
Unaligned length	58650
Genome fraction (%)	1.729
Duplication ratio	1.145
# N's per 100 kbp	126.60
# mismatches per 100 kbp	0.00
# indels per 100 kbp	0.00
# genomic features	0 + 6 part
Largest alignment	517
Total aligned length	517
NGA50	-

All statistics are based on contigs of size >= 500 bp, unless otherwise noted (e.g., "# contigs (>= 0 bp)" and "Total length (>= 0 bp)" include all contigs).

Misassemblies report

	CV29_velvet_contigs
# misassemblies	0
# contig misassemblies	0
# c. relocations	0
# c. translocations	0
# c. inversions	0
# scaffold misassemblies	0
# s. relocations	0
# s. translocations	0
# s. inversions	0
# misassembled contigs	0
Misassembled contigs length	0
# local misassemblies	0
# scaffold gap ext. mis.	0
# scaffold gap loc. mis.	0
# unaligned mis. contigs	0
# mismatches	0
# indels	0
# indels (<= 5 bp)	0
# indels (> 5 bp)	0
Indels length	0

All statistics are based on contigs of size >= 500 bp, unless otherwise noted (e.g., "# contigs (>= 0 bp)" and "Total length (>= 0 bp)" include all contigs).

Unaligned report

	CV29_velvet_contigs
# fully unaligned contigs	65
Fully unaligned length	58650
# partially unaligned contigs	0
Partially unaligned length	0
# N's	75

All statistics are based on contigs of size >= 500 bp, unless otherwise noted (e.g., "# contigs (>= 0 bp)" and "Total length (>= 0 bp)" include all contigs).

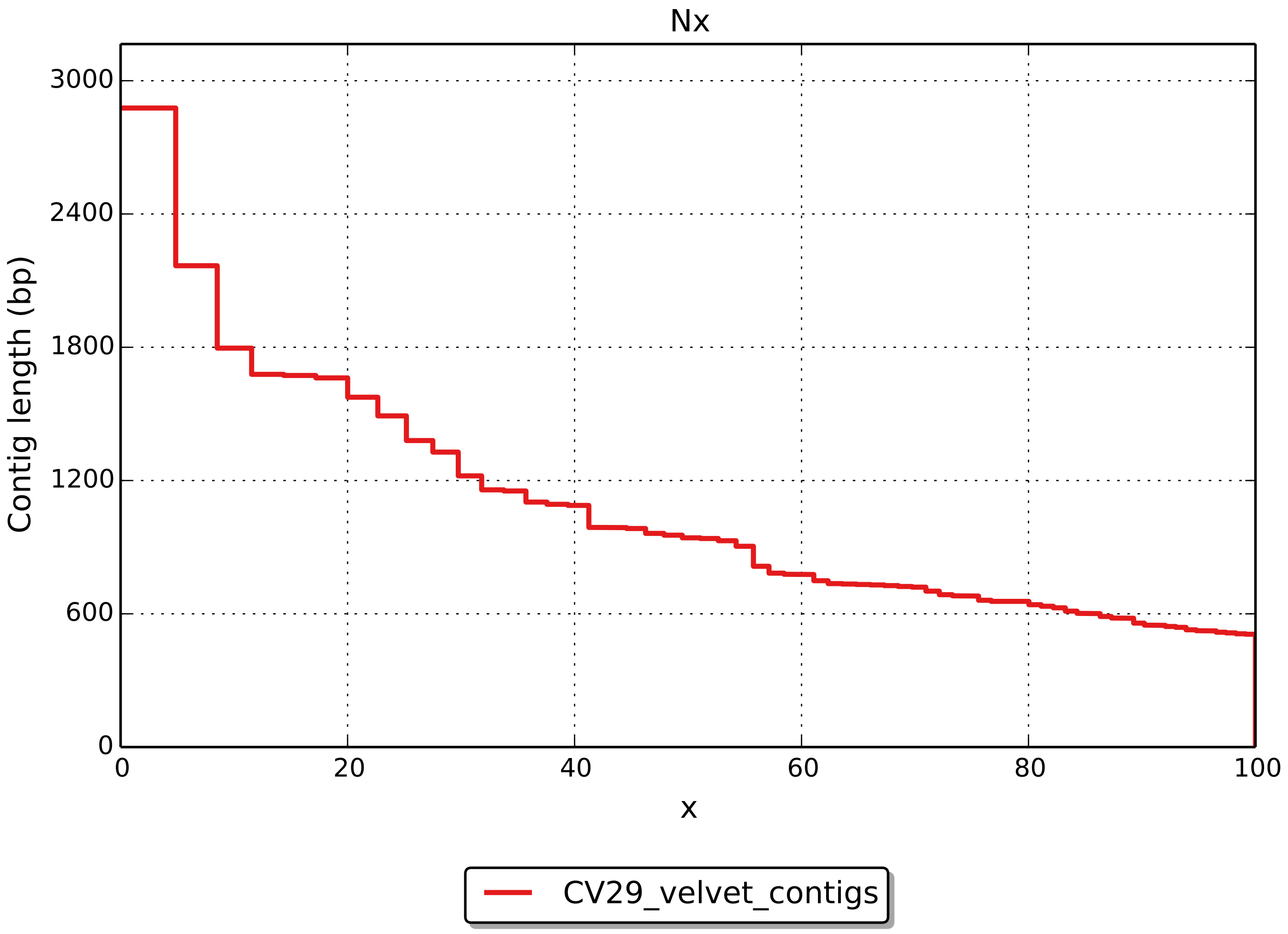
Nx
Contig length (bp)
3000
2400
1800
1200
600
0
0
20
40
60
80
100
x
CV29_velvet_contigs

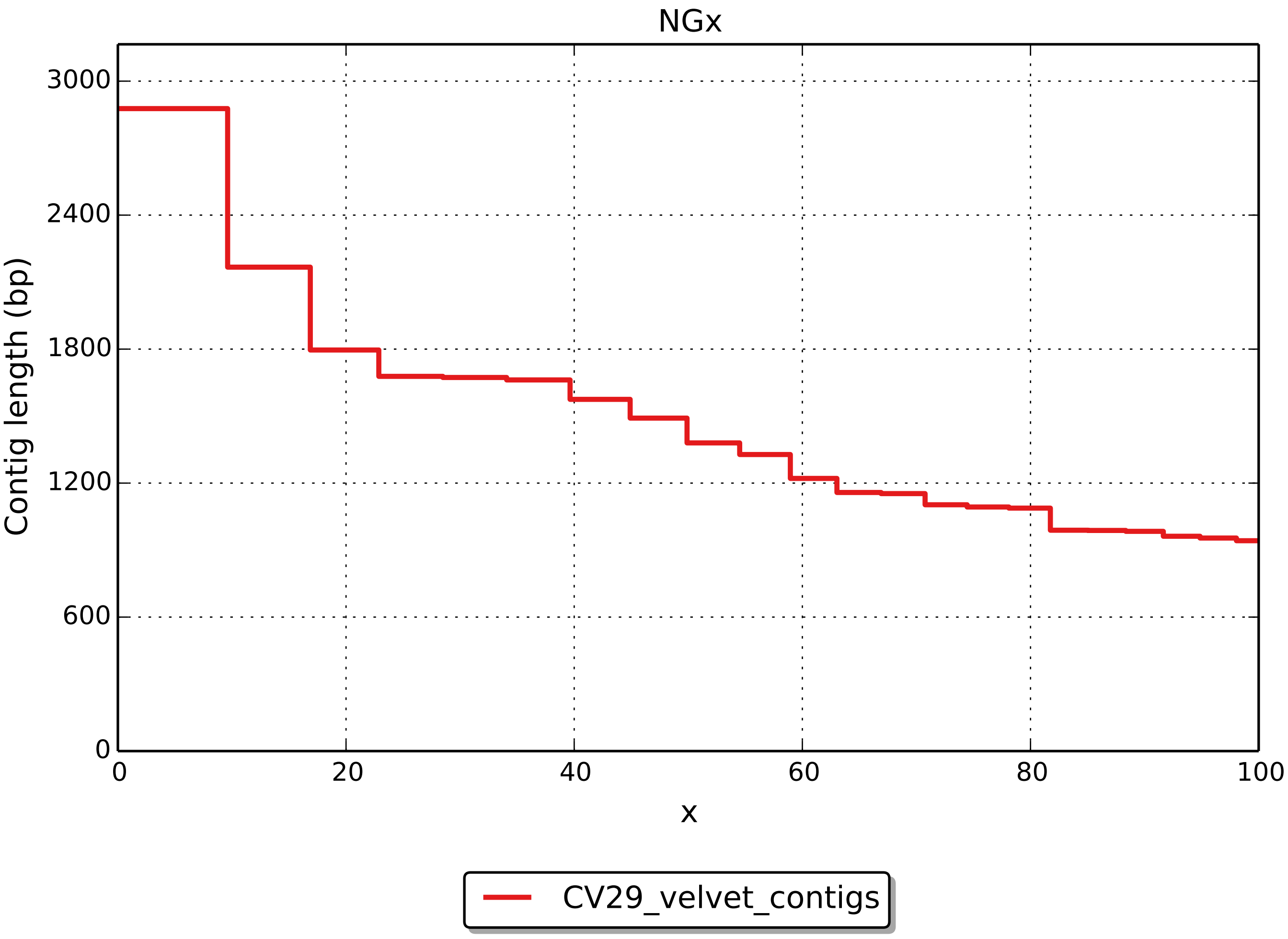

NGx
3000
2400
1800
1200
600
0
0
20
40
60
80
100
x
Contig length (bp)
CV29_velvet_contigs

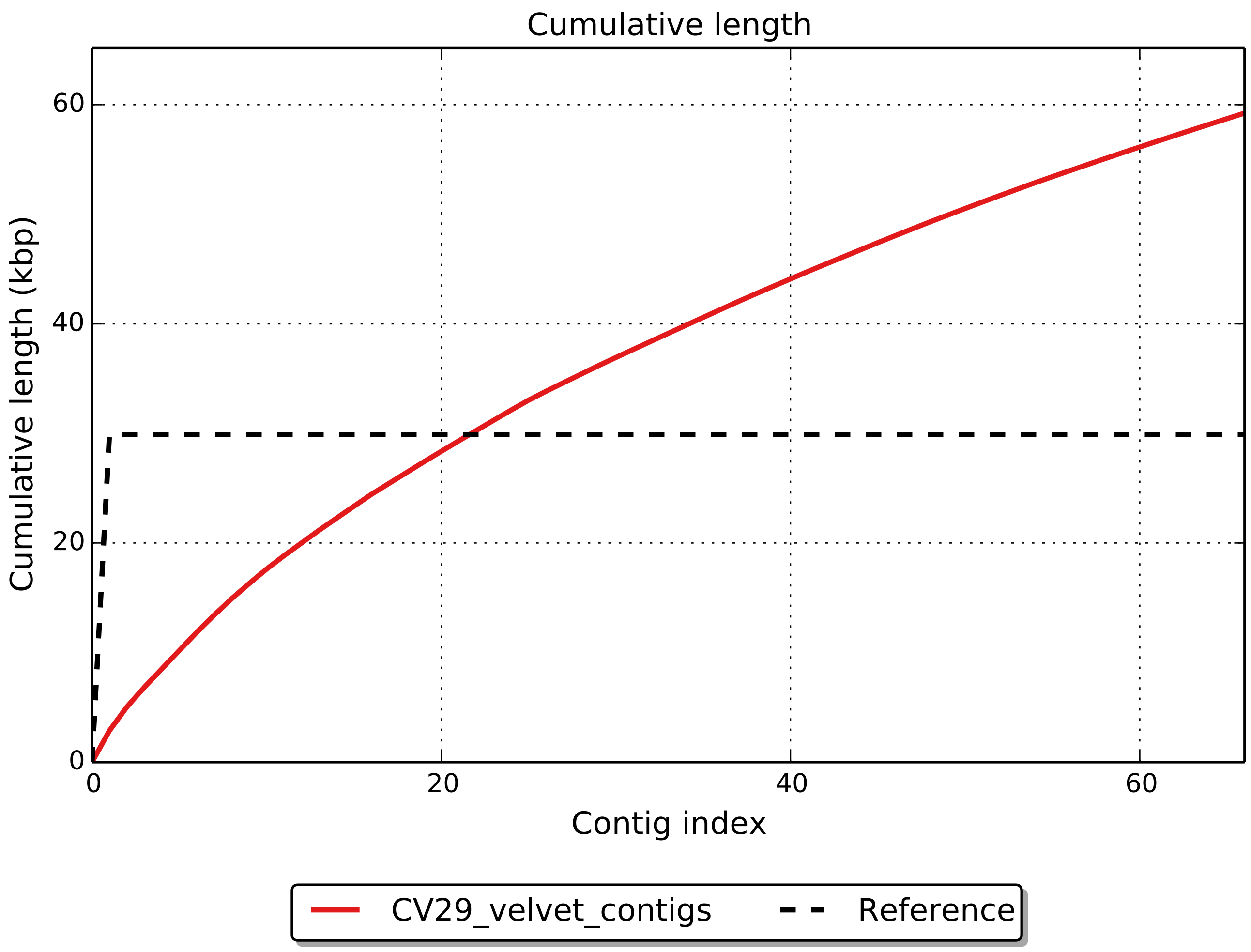
Cumulative length
Cumulative length (kbp)
0
20
40
60
0
20
40
60
Contig index
CV29_velvet_contigs
Reference

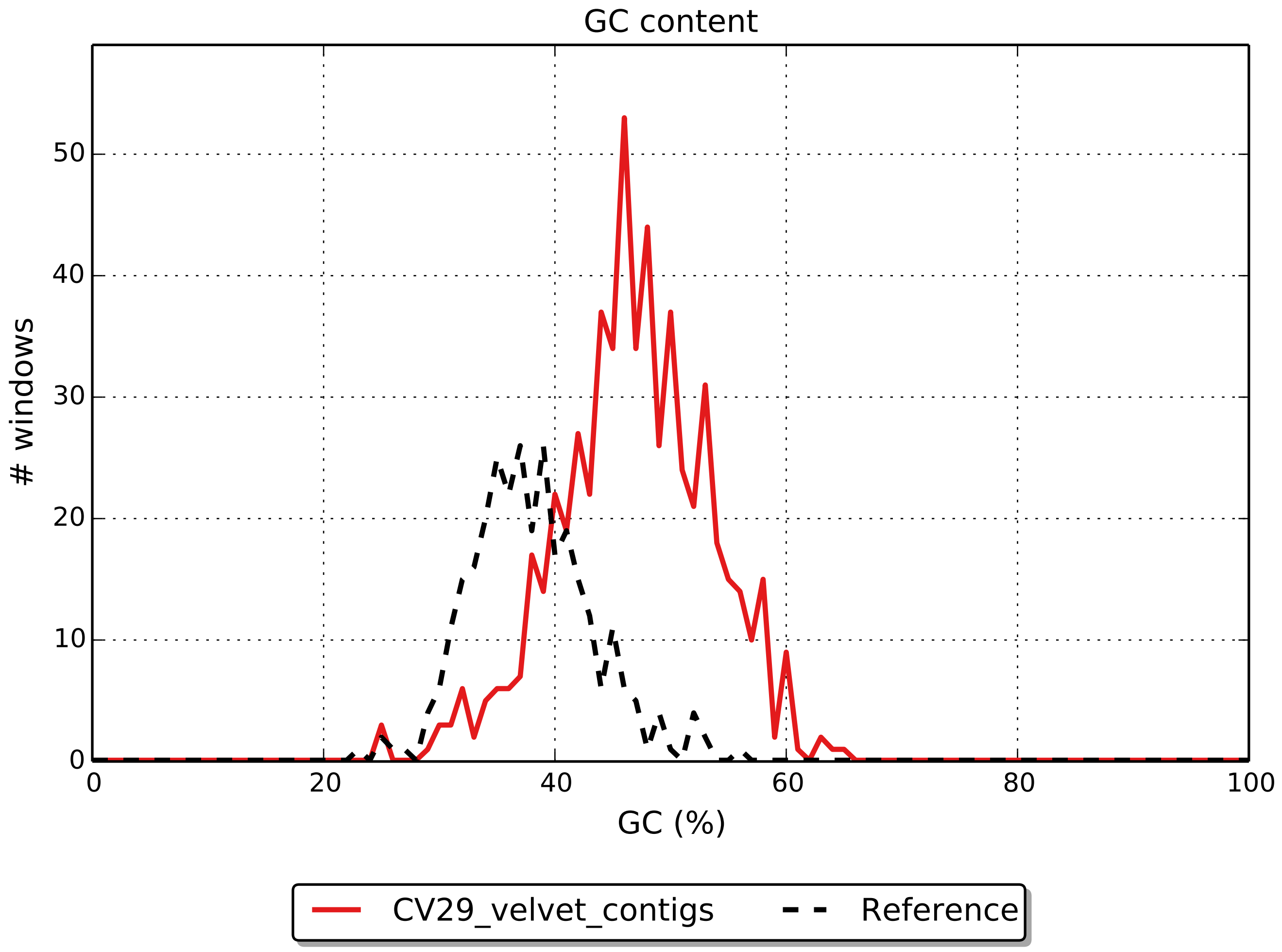
GC content
windows
GC (%)
0
10
20
30
40
50
0
20
40
60
80
100
CV29_velvet_contigs
Reference

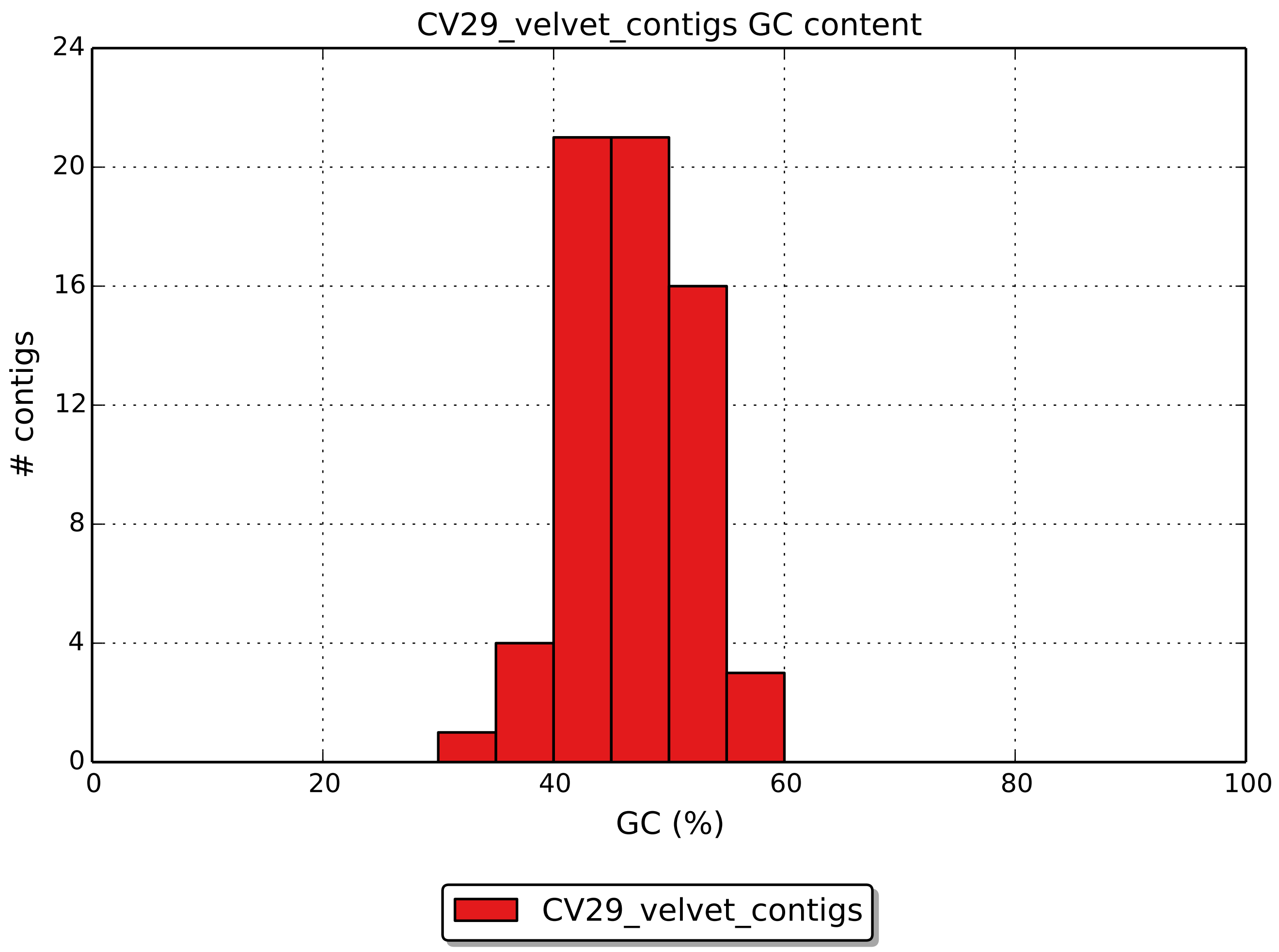

CV29_velvet_contigs GC content
contigs
GC (%)
0
4
8
12
16
20
24
0
20
40
60
80
100
CV29_velvet_contigs

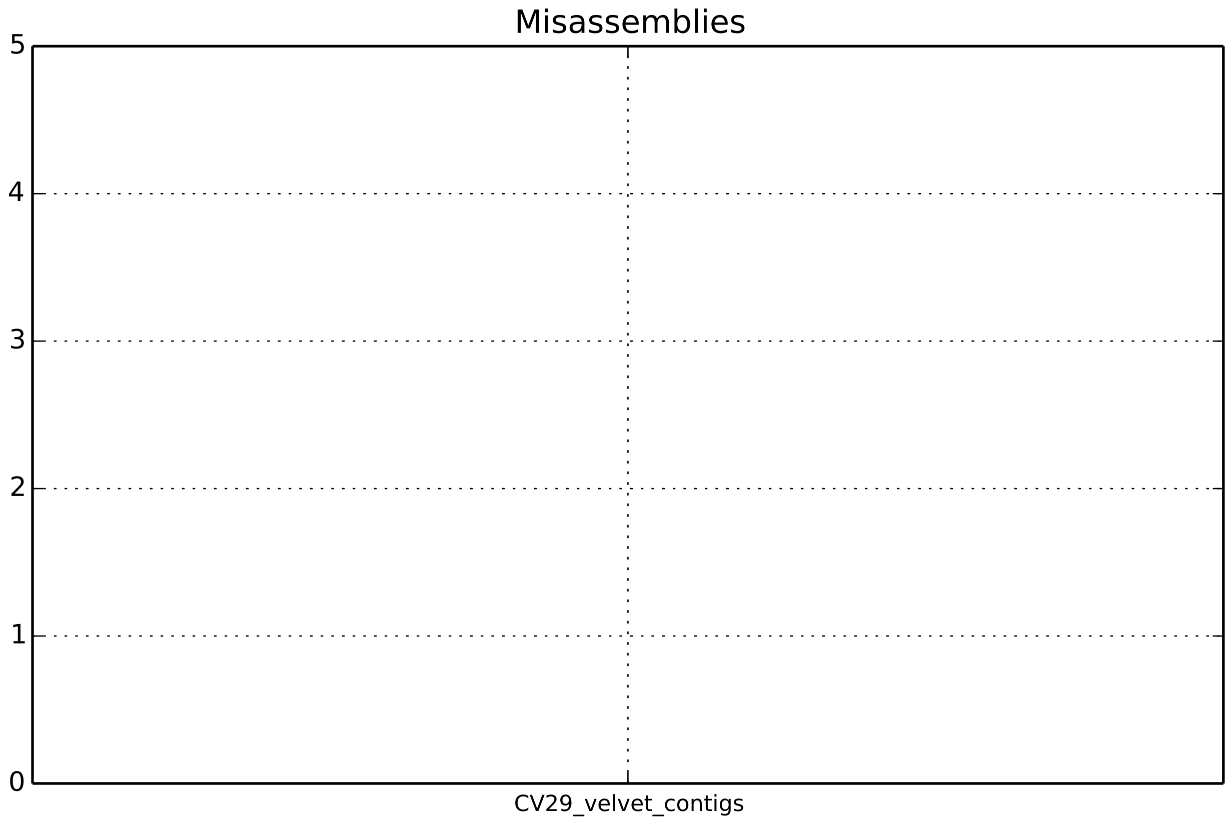
Misassemblies
5
4
3
2
1
0
CV29_velvet_contigs

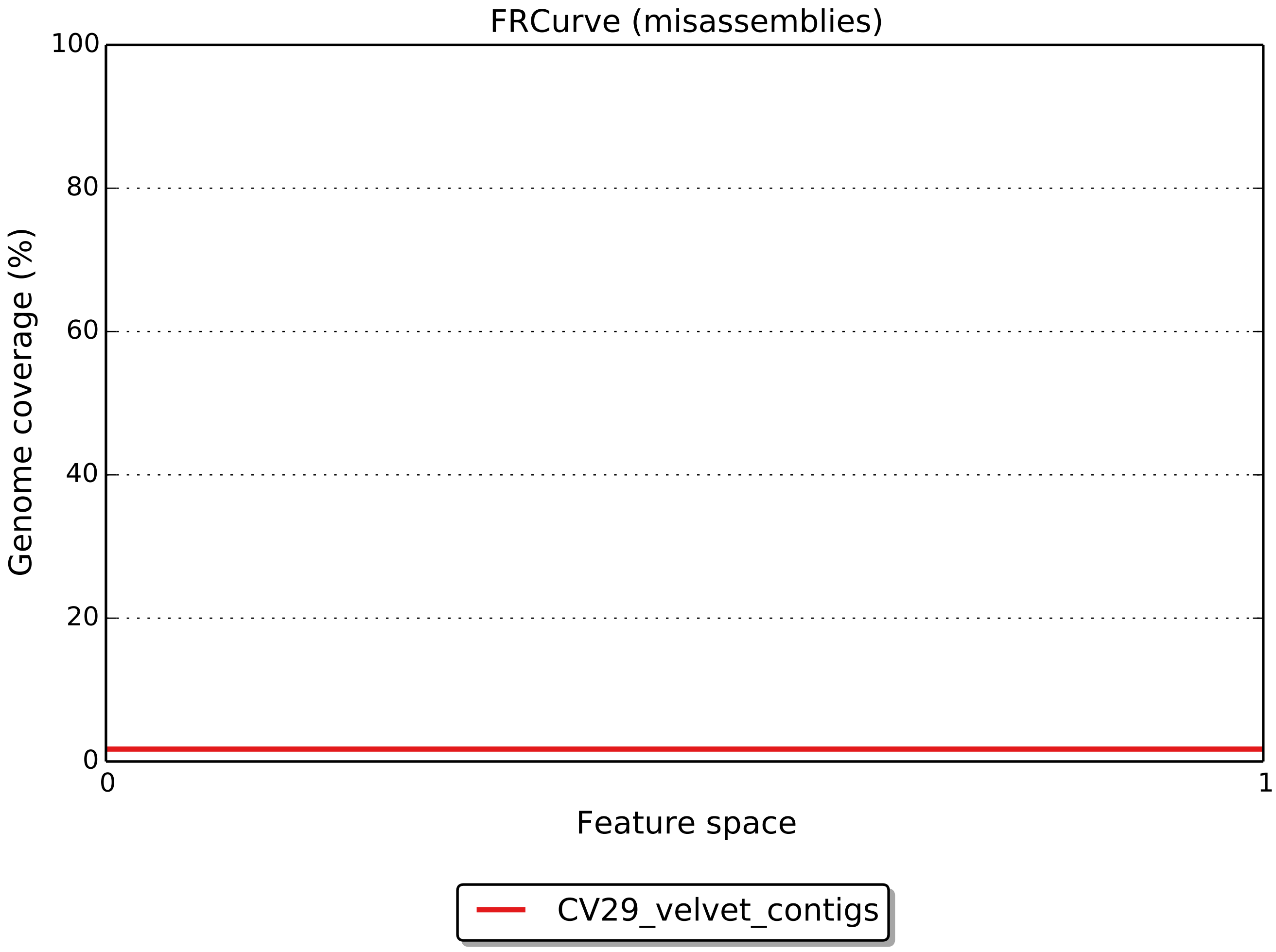

FRCurve (misassemblies)
100
80
60
40
20
0
0
1
Genome coverage (%)
Feature space
CV29_velvet_contigs

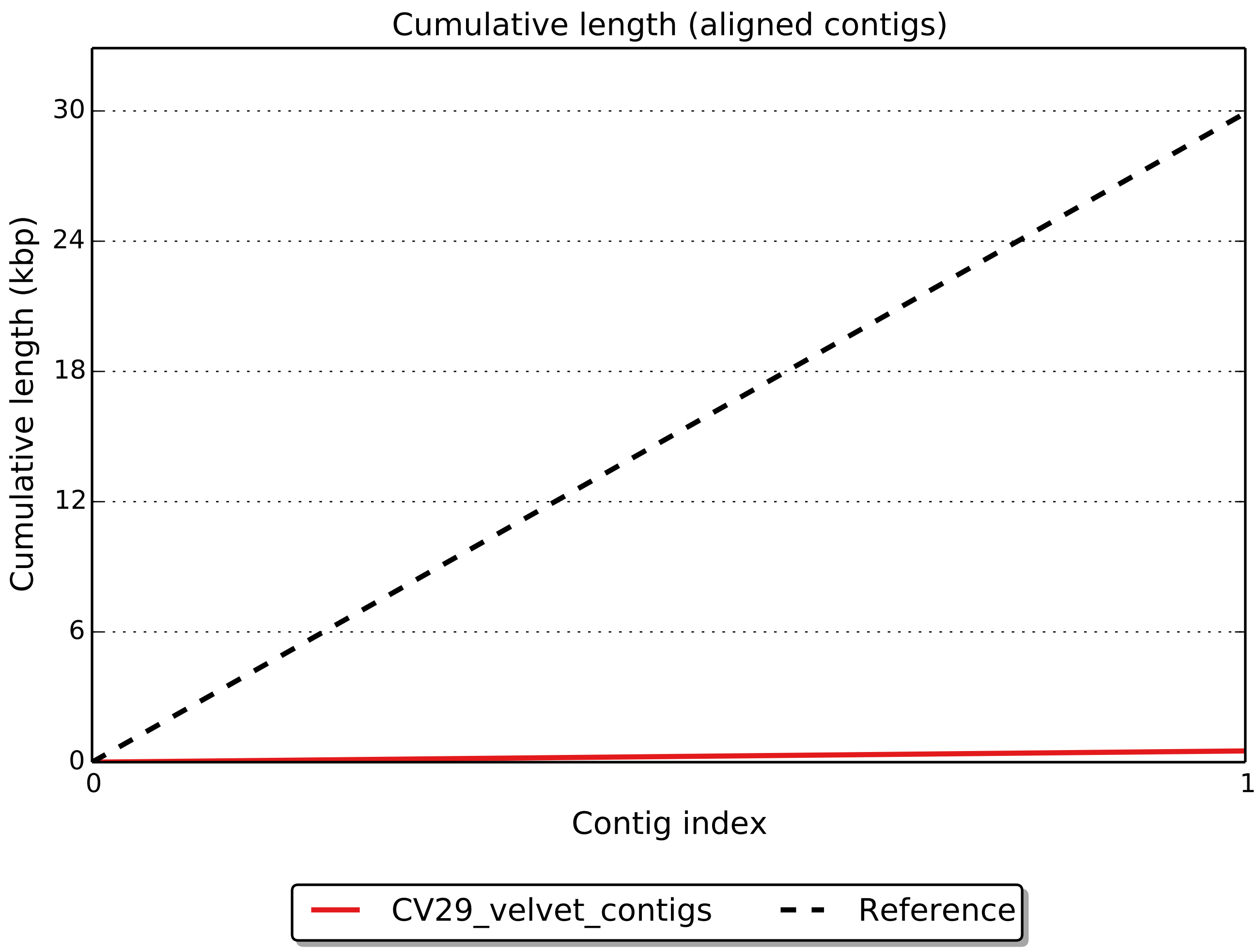
Cumulative length (aligned contigs)
Cumulative length (kbp)
0
6
12
18
24
30
0
1
Contig index
CV29_velvet_contigs
Reference

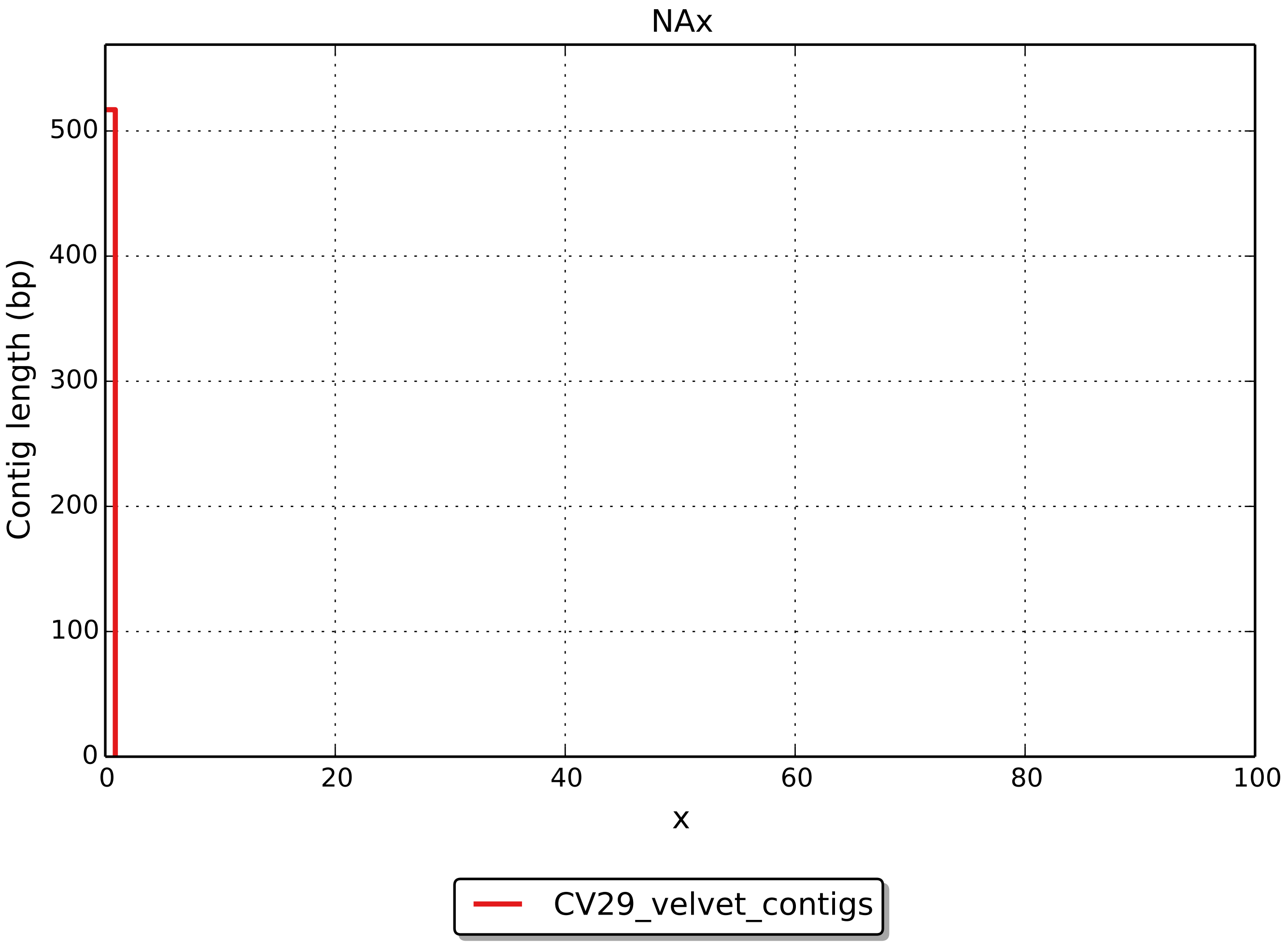

NAx
Contig length (bp)
500
400
300
200
100
0
0
20
40
60
80
100
x
CV29_velvet_contigs

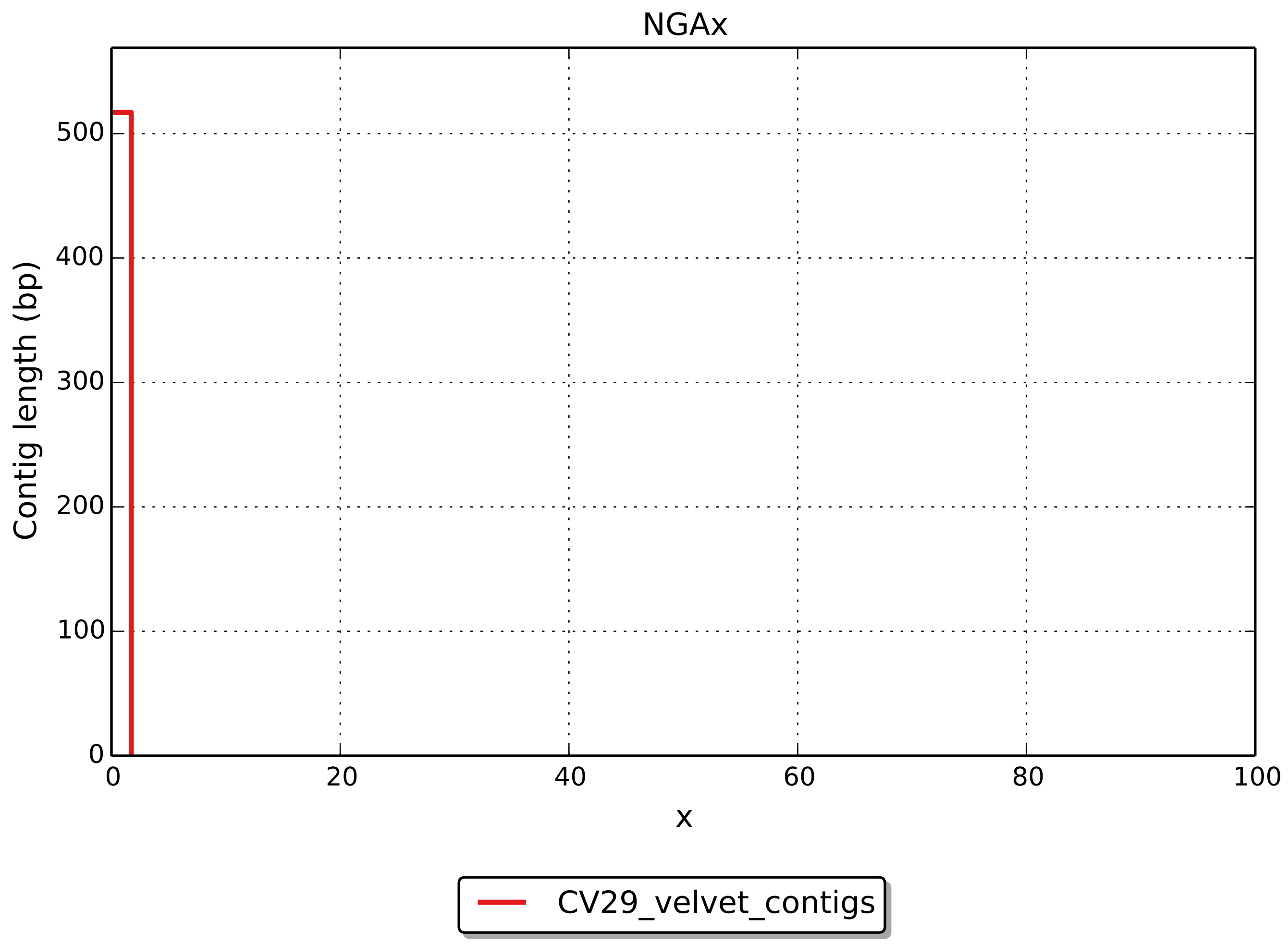
NGAx
Contig length (bp)
500
400
300
200
100
0
0
20
40
60
80
100
x
CV29_velvet_contigs

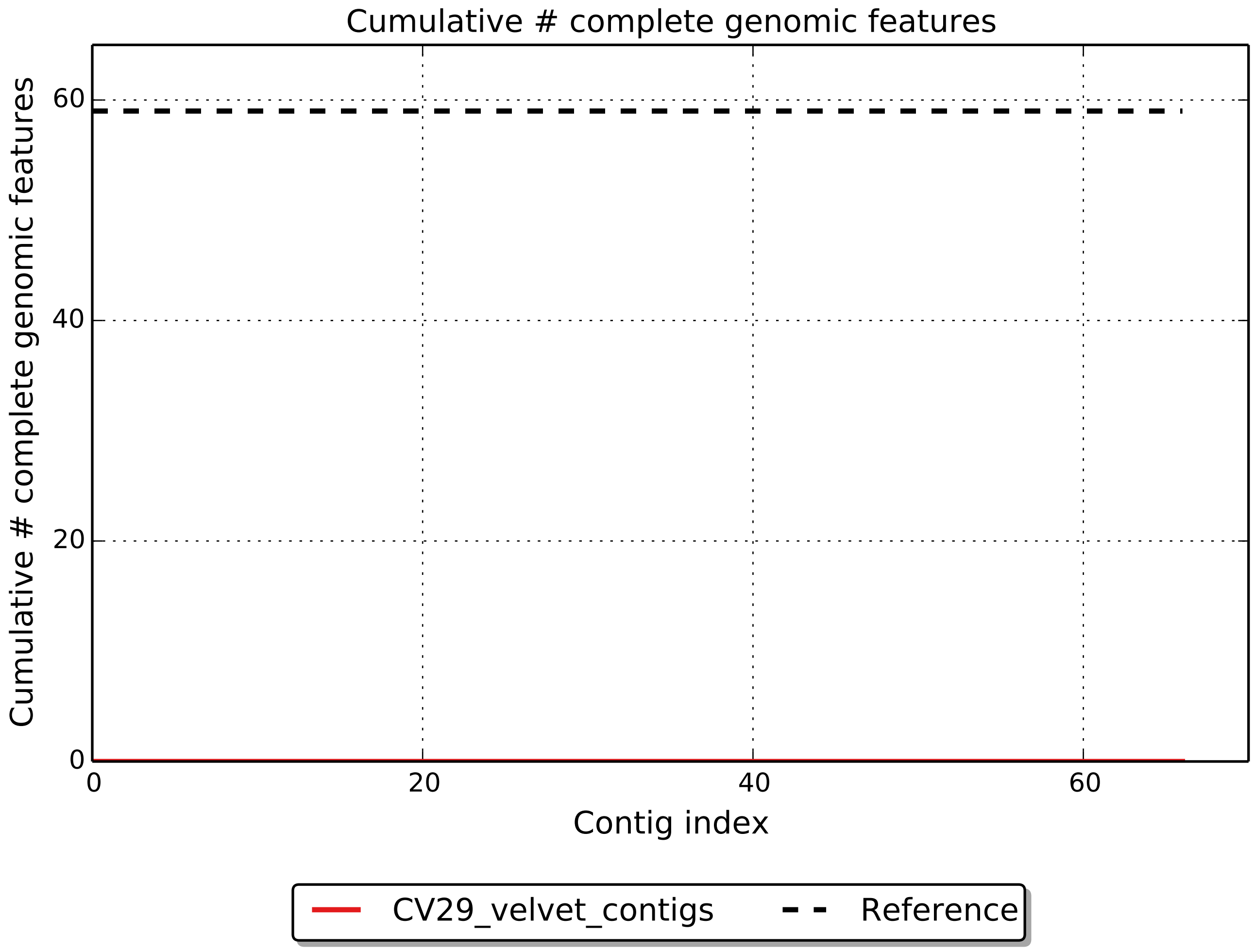

Cumulative # complete genomic features
Cumulative # complete genomic features
Contig index
0
20
40
60
0
20
40
60
CV29_velvet_contigs
Reference

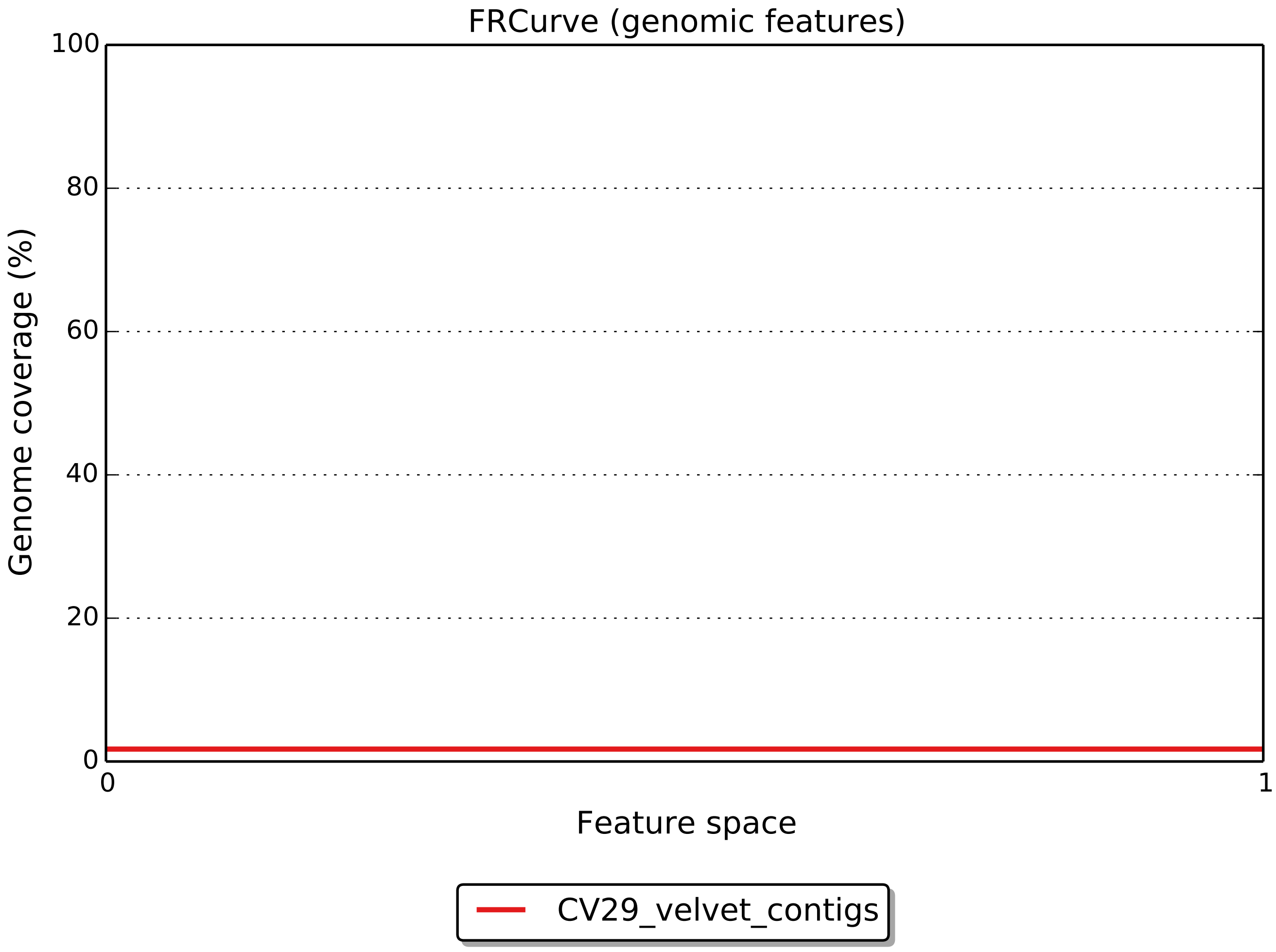
FRCurve (genomic features)
Genome coverage (%)
100
80
60
40
20
0
0
1
Feature space
CV29_velvet_contigs
