# Supplementary material for: VGEA: an RNA viral assembly toolkit
Source: PeerJ. 2021 Sep 6;9:e12129. doi: 10.7717/peerj.12129 (PMC8428259; doi:10.7717/peerj.12129)
Supplement: Supplemental Information 2 [file peerj-09-12129-s002.zip › Supplementary_File_2/Performance_Comparison_Assembly_Pipelines_Table_2/CV29/vgea/quast_results/results_2021_05_13_22_59_11/report.pdf]

Report

	CV29_contigs
# contigs (>= 0 bp)	31
# contigs (>= 1000 bp)	24
# contigs (>= 5000 bp)	3
# contigs (>= 10000 bp)	0
# contigs (>= 25000 bp)	0
# contigs (>= 50000 bp)	0
Total length (>= 0 bp)	66226
Total length (>= 1000 bp)	60605
Total length (>= 5000 bp)	22178
Total length (>= 10000 bp)	0
Total length (>= 25000 bp)	0
Total length (>= 50000 bp)	0
# contigs	31
Largest contig	7731
Total length	66226
Reference length	29903
GC (%)	45.10
Reference GC (%)	37.97
N50	3065
NG50	7534
N75	1433
NG75	4428
L50	7
LG50	2
L75	15
LG75	4
# misassemblies	0
# misassembled contigs	0
Misassembled contigs length	0
# local misassemblies	0
# scaffold gap ext. mis.	0
# scaffold gap loc. mis.	0
# unaligned mis. contigs	0
# unaligned contigs	25 + 0 part
Unaligned length	33568
Genome fraction (%)	99.786
Duplication ratio	1.094
# N's per 100 kbp	0.00
# mismatches per 100 kbp	30.16
# indels per 100 kbp	0.00
# genomic features	49 + 10 part
Largest alignment	7598
Total aligned length	32041
NGA50	7459
NGA75	4321
LGA50	2
LGA75	4

All statistics are based on contigs of size >= 500 bp, unless otherwise noted (e.g., "# contigs (>= 0 bp)" and "Total length (>= 0 bp)" include all contigs).

Misassemblies report

	CV29_contigs
# misassemblies	0
# contig misassemblies	0
# c. relocations	0
# c. translocations	0
# c. inversions	0
# scaffold misassemblies	0
# s. relocations	0
# s. translocations	0
# s. inversions	0
# misassembled contigs	0
Misassembled contigs length	0
# local misassemblies	0
# scaffold gap ext. mis.	0
# scaffold gap loc. mis.	0
# unaligned mis. contigs	0
# mismatches	9
# indels	0
# indels (<= 5 bp)	0
# indels (> 5 bp)	0
Indels length	0

All statistics are based on contigs of size >= 500 bp, unless otherwise noted (e.g., "# contigs (>= 0 bp)" and "Total length (>= 0 bp)" include all contigs).

Unaligned report

	CV29_contigs
# fully unaligned contigs	25
Fully unaligned length	33568
# partially unaligned contigs	0
Partially unaligned length	0
# N's	0

All statistics are based on contigs of size >= 500 bp, unless otherwise noted (e.g., "# contigs (>= 0 bp)" and "Total length (>= 0 bp)" include all contigs).

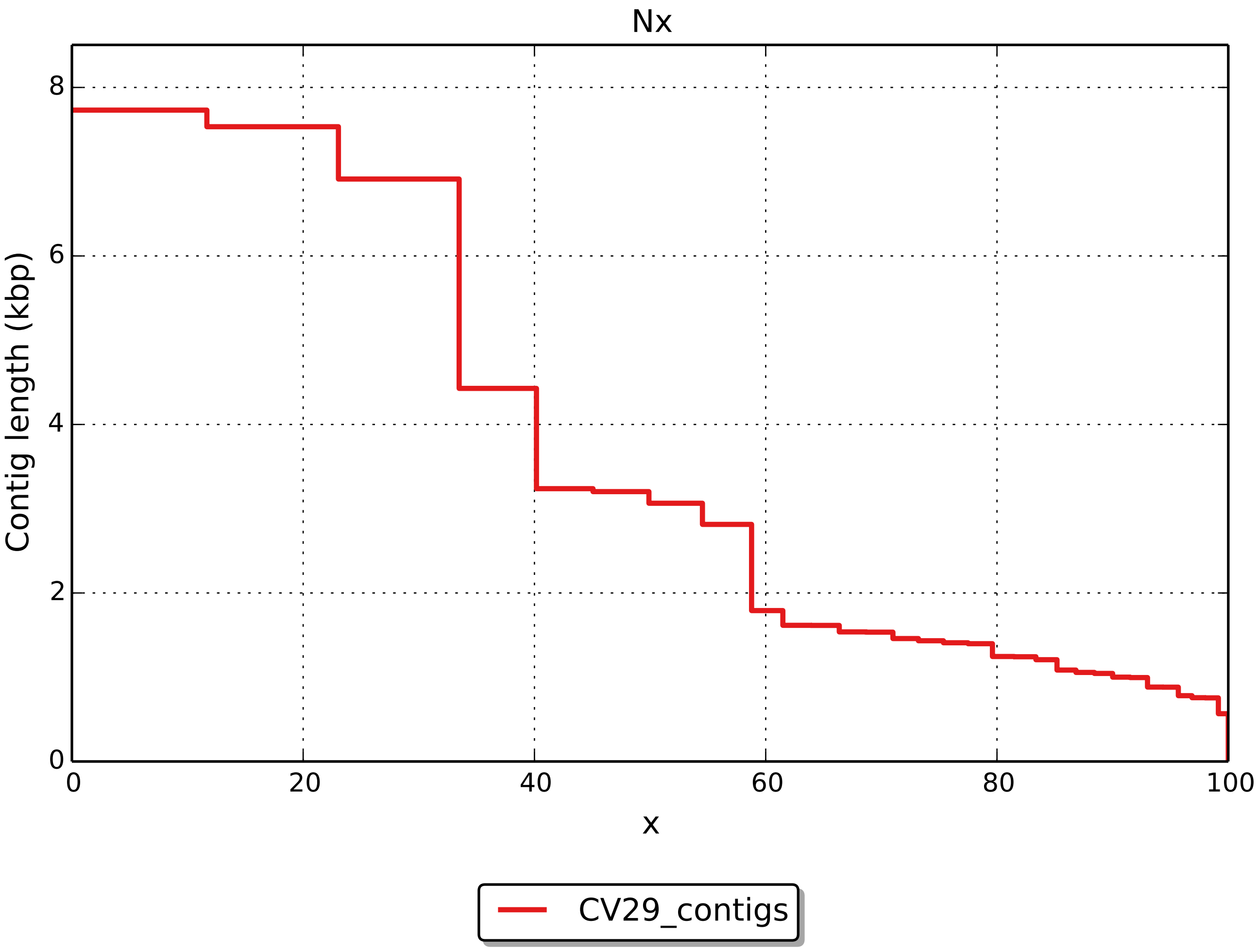
Nx
Contig length (kbp)
0
2
4
6
8
0
20
40
60
80
100
x
CV29_contigs

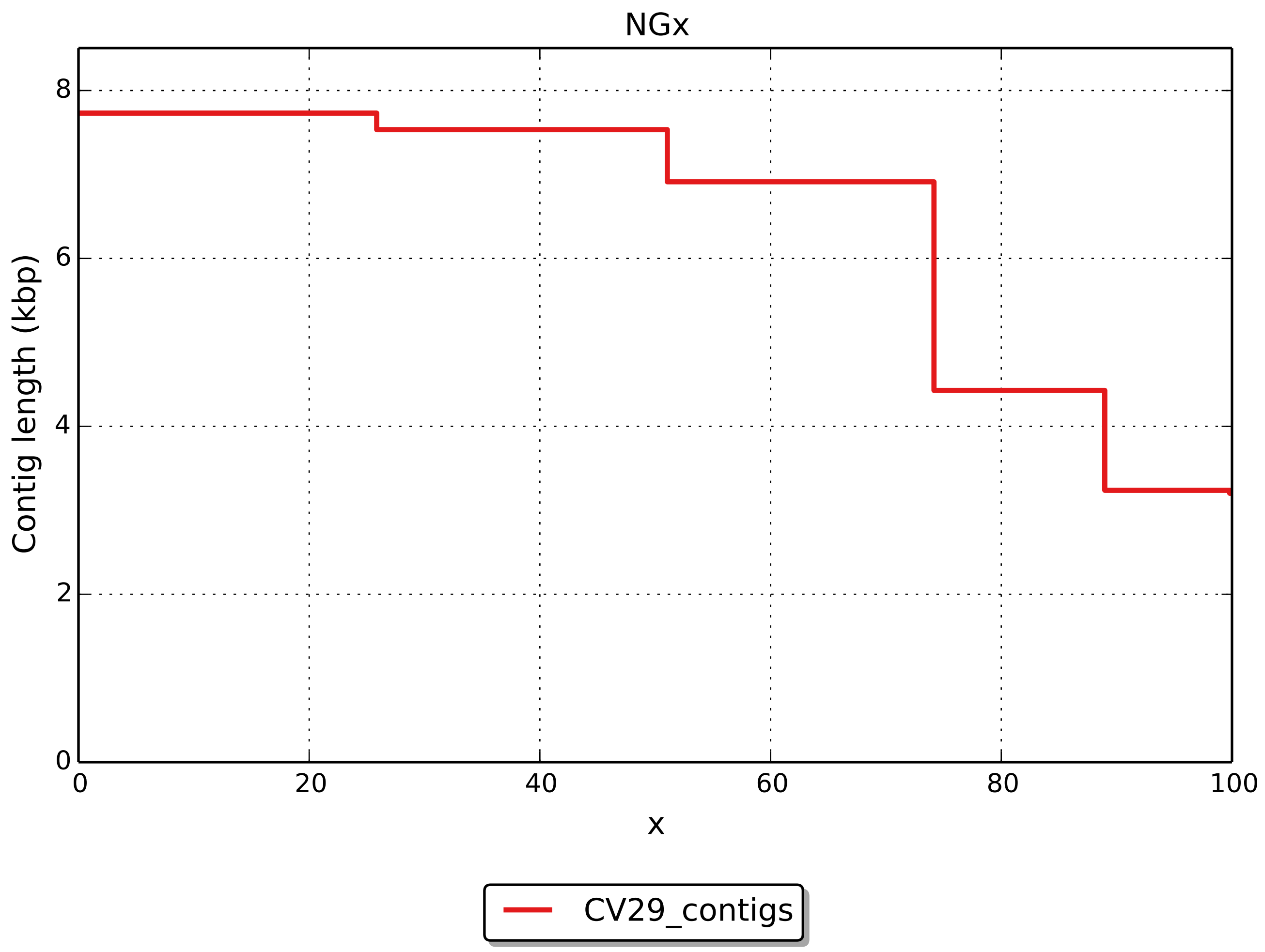
NGx
Contig length (kbp)
0
2
4
6
8
0
20
40
60
80
100
x
CV29_contigs

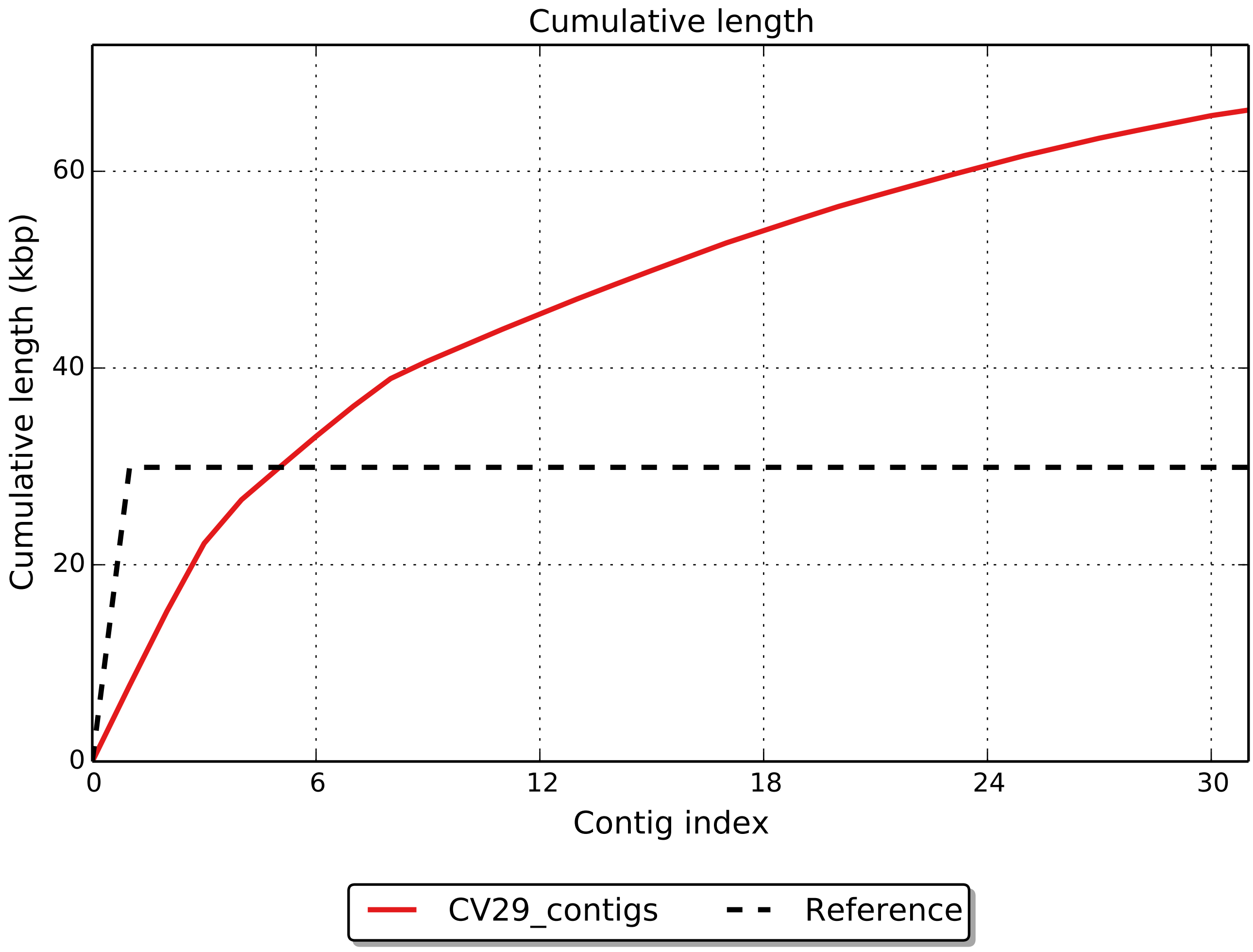

Cumulative length
Cumulative length (kbp)
0
20
40
60
0
6
12
18
24
30
Contig index
CV29_contigs
Reference

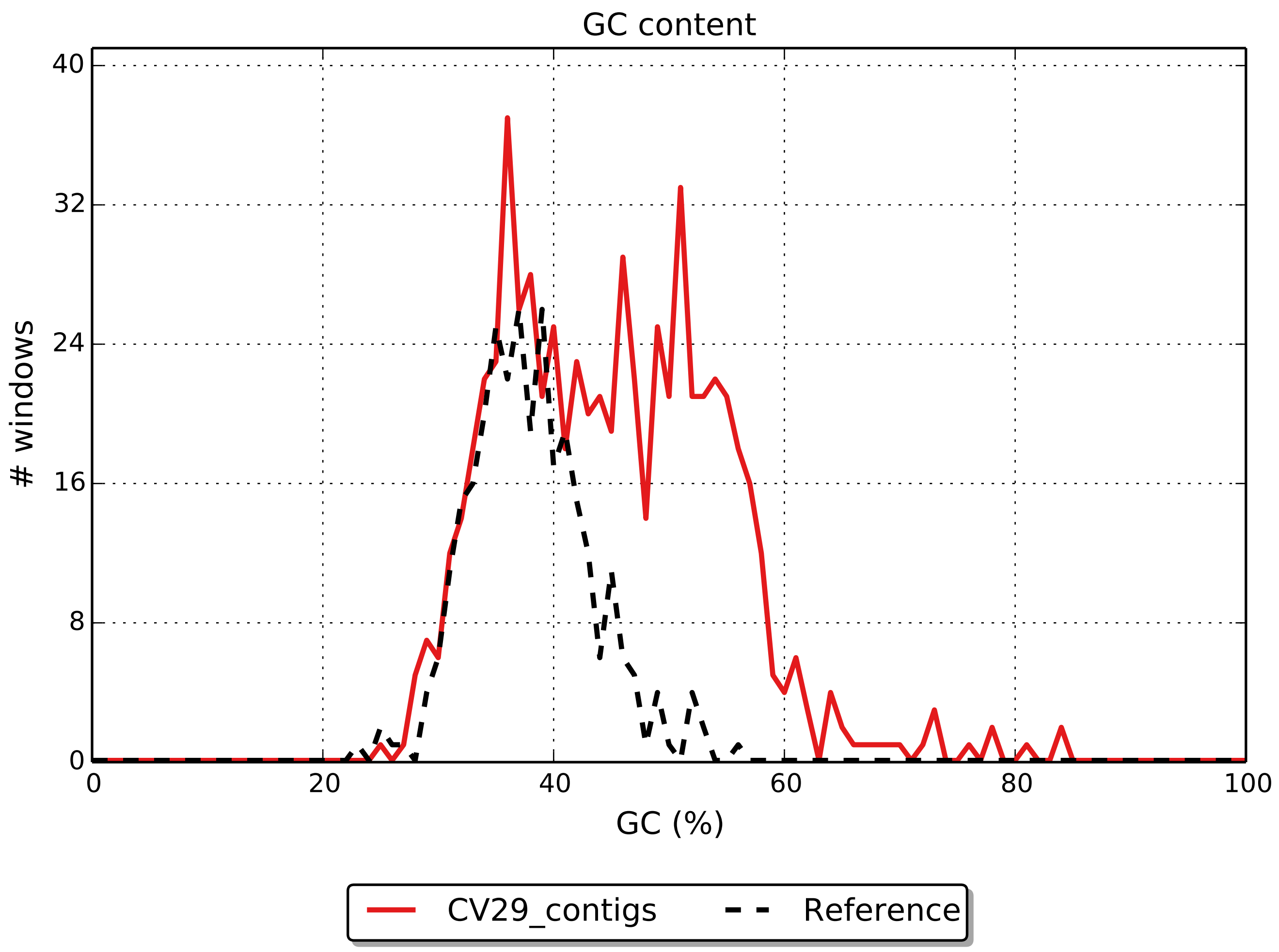
GC content
windows
GC (%)
0
8
16
24
32
40
0
20
40
60
80
100
CV29_contigs
Reference

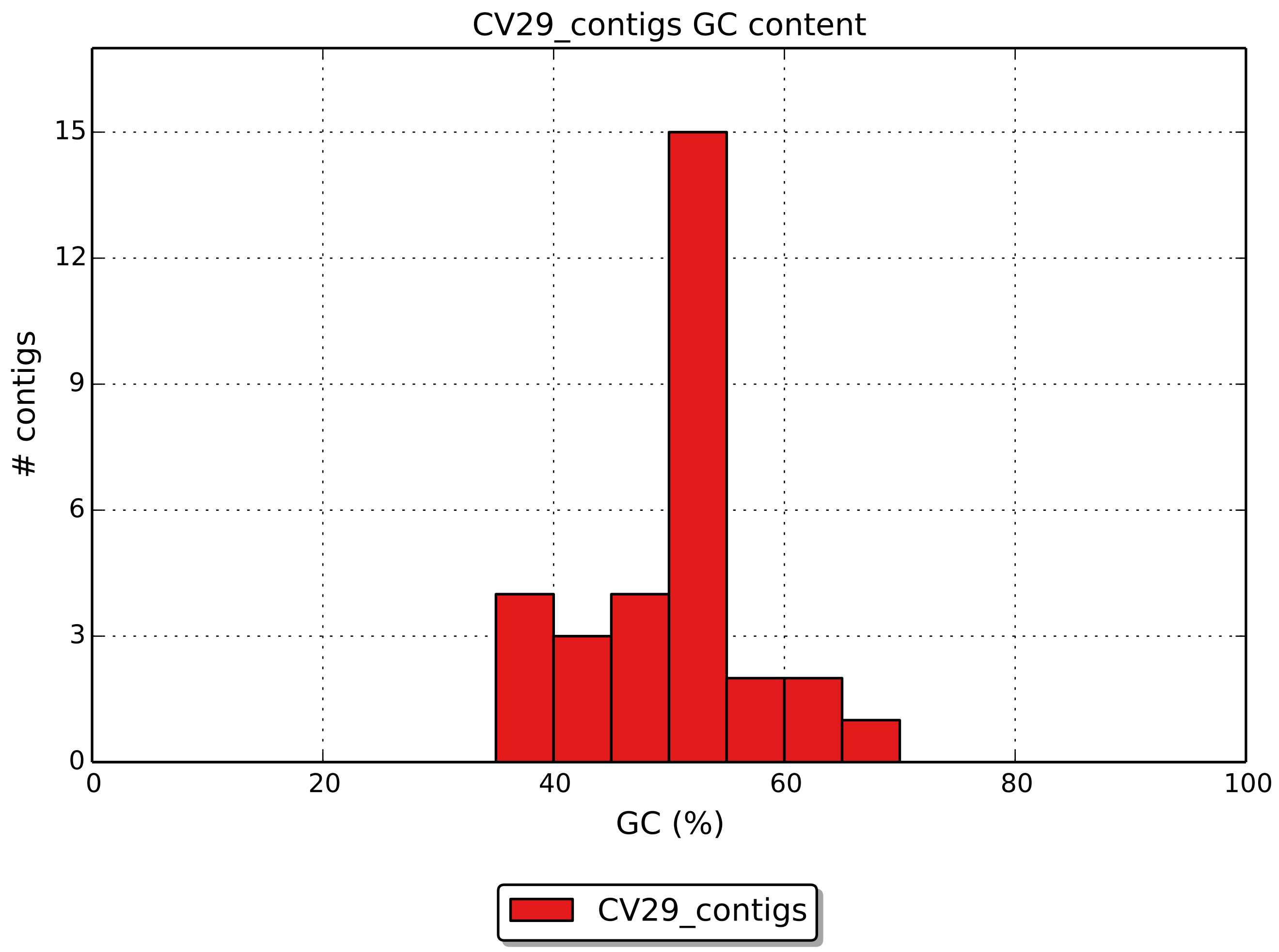
CV29_contigs GC content
contigs
0
3
6
9
12
15
0
20
40
60
80
100
GC (%)
CV29_contigs

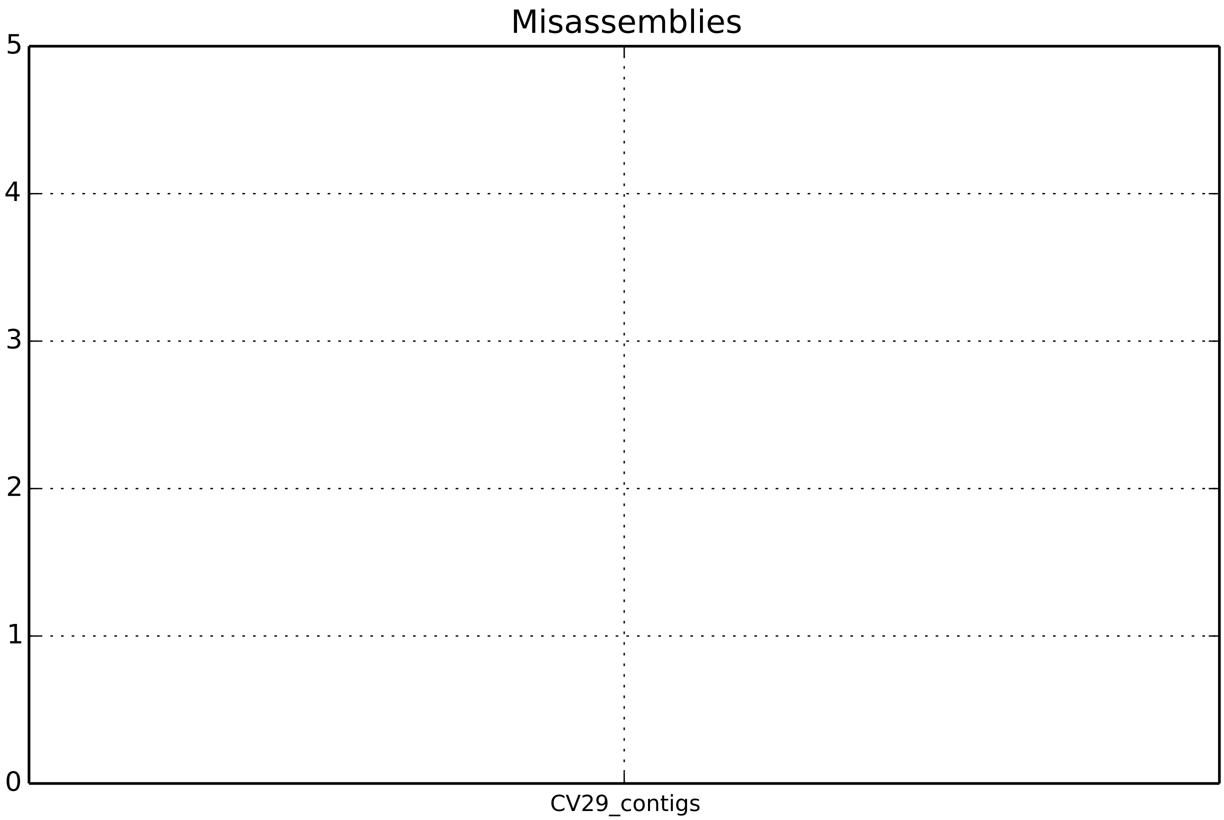
Misassemblies
5
4
3
2
1
0
CV29_contigs

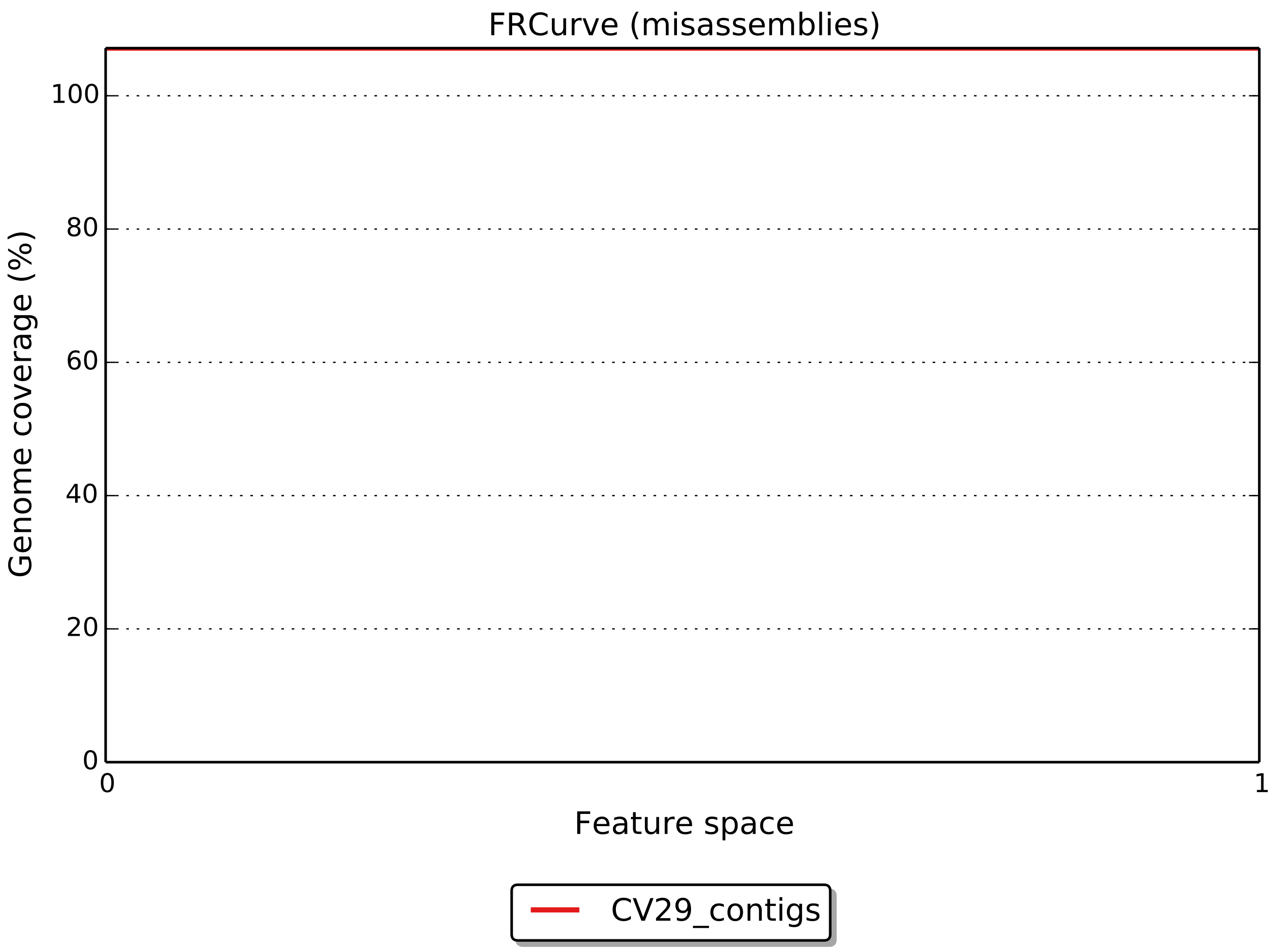
FRCurve (misassemblies)
Genome coverage (%)
100
80
60
40
20
0
0
1
Feature space
CV29_contigs

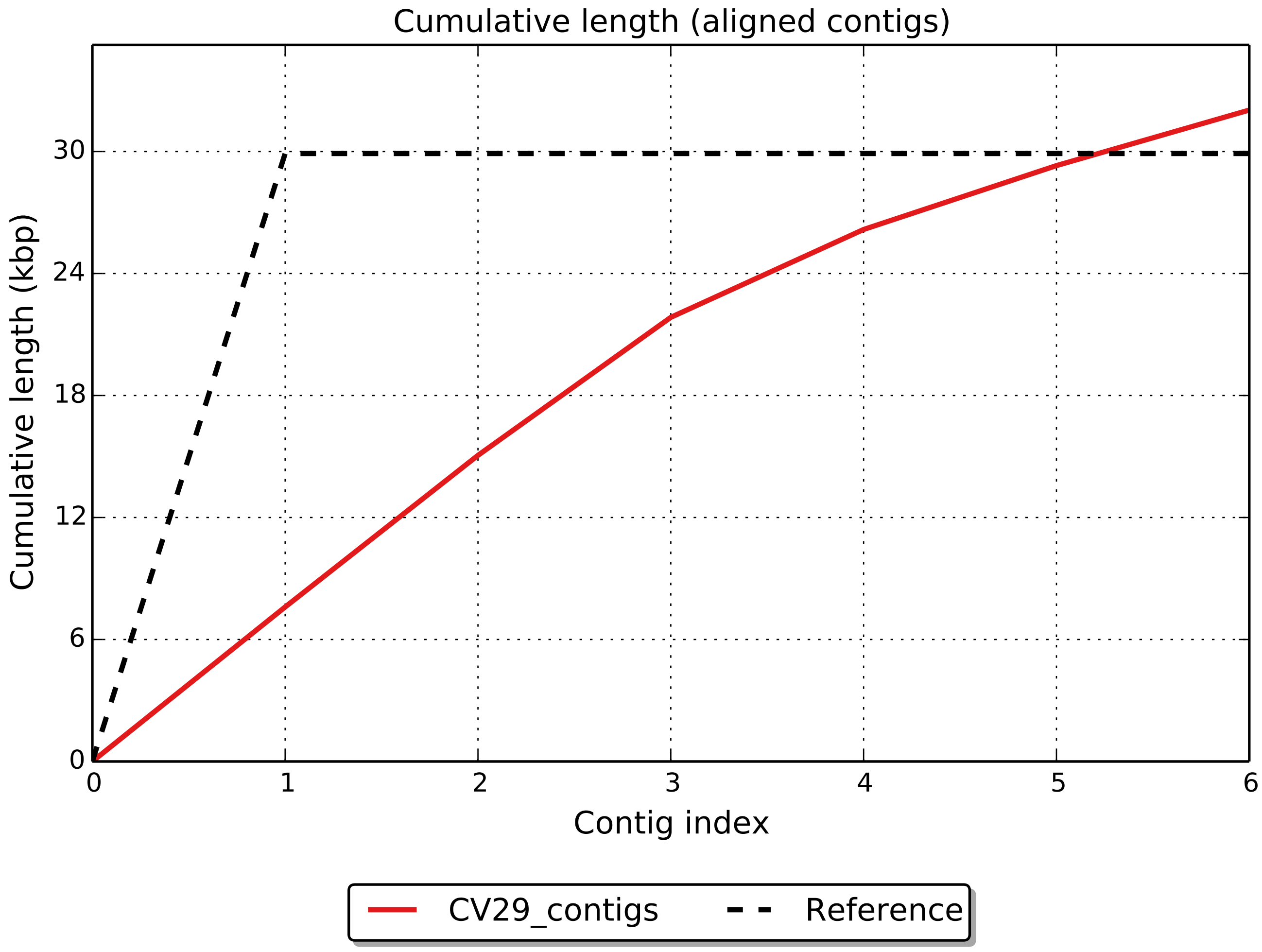
Cumulative length (aligned contigs)
Cumulative length (kbp)
0
6
12
18
24
30
0
1
2
3
4
5
6
Contig index
CV29_contigs
Reference

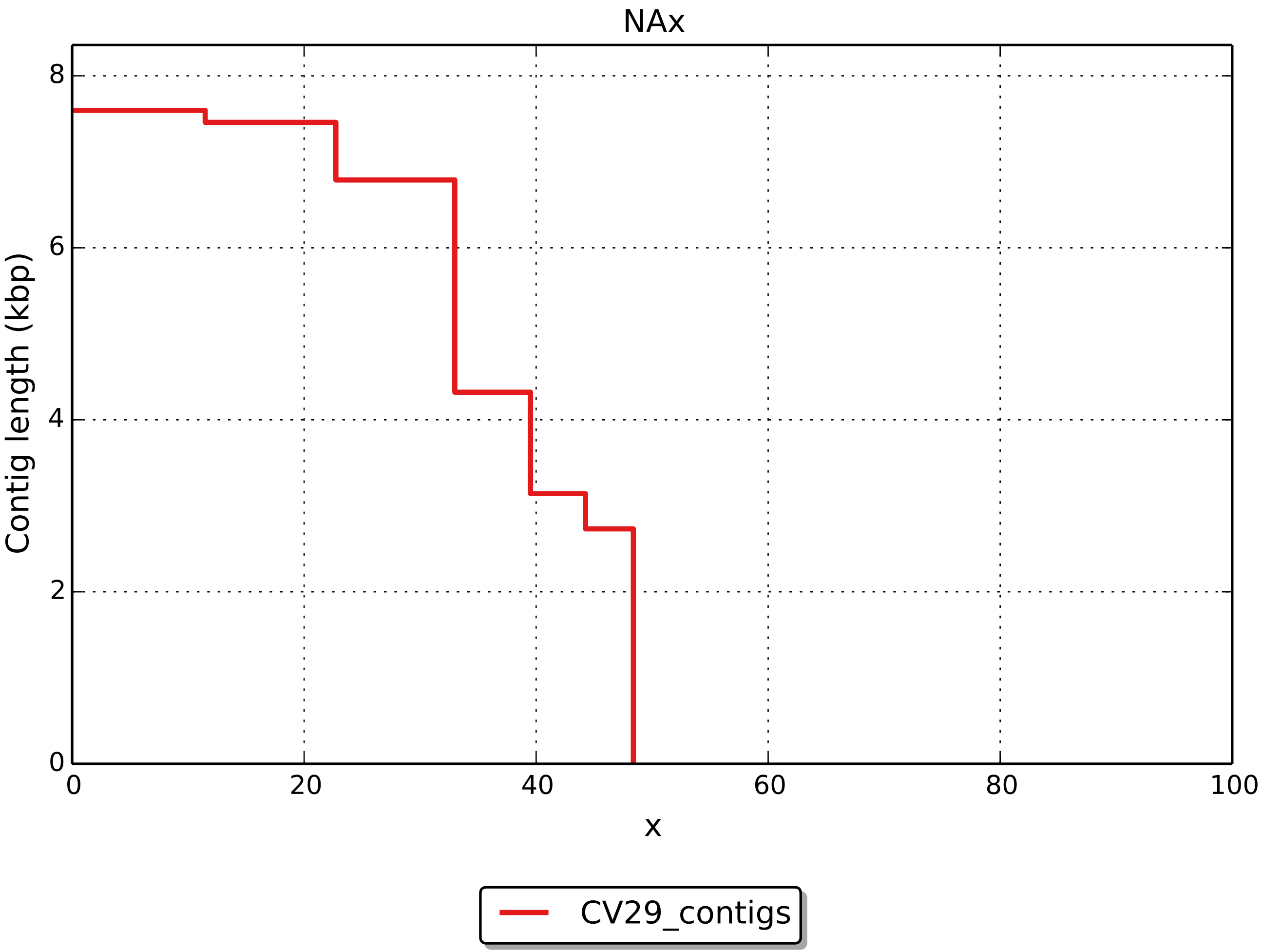
NAx
Contig length (kbp)
8
6
4
2
0
0
20
40
60
80
100
x
CV29_contigs

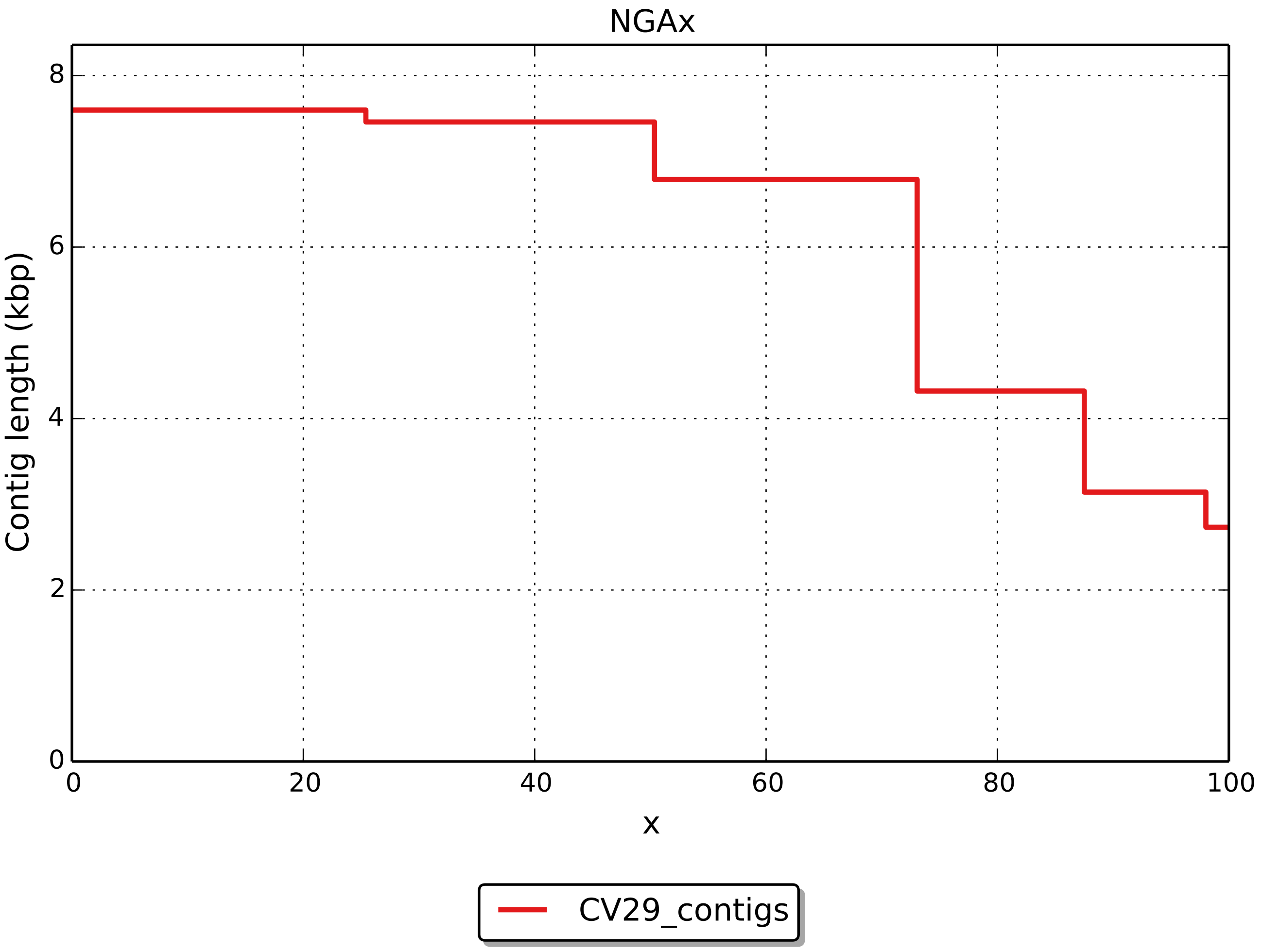
NGAx
Contig length (kbp)
8
6
4
2
0
0
20
40
60
80
100
x
CV29_contigs

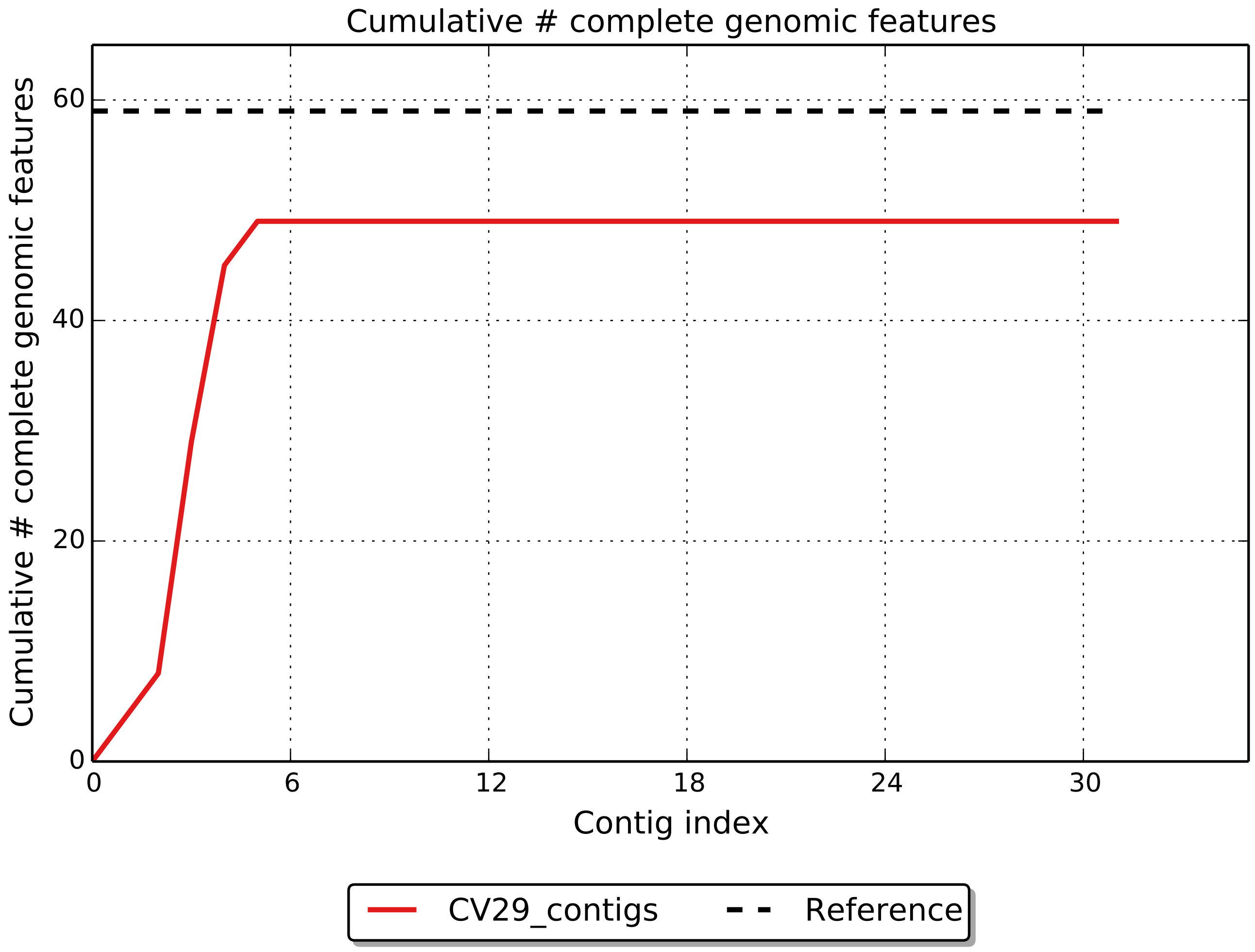
Cumulative # complete genomic features
Cumulative # complete genomic features
0
20
40
60
0
6
12
18
24
30
Contig index
CV29_contigs
Reference

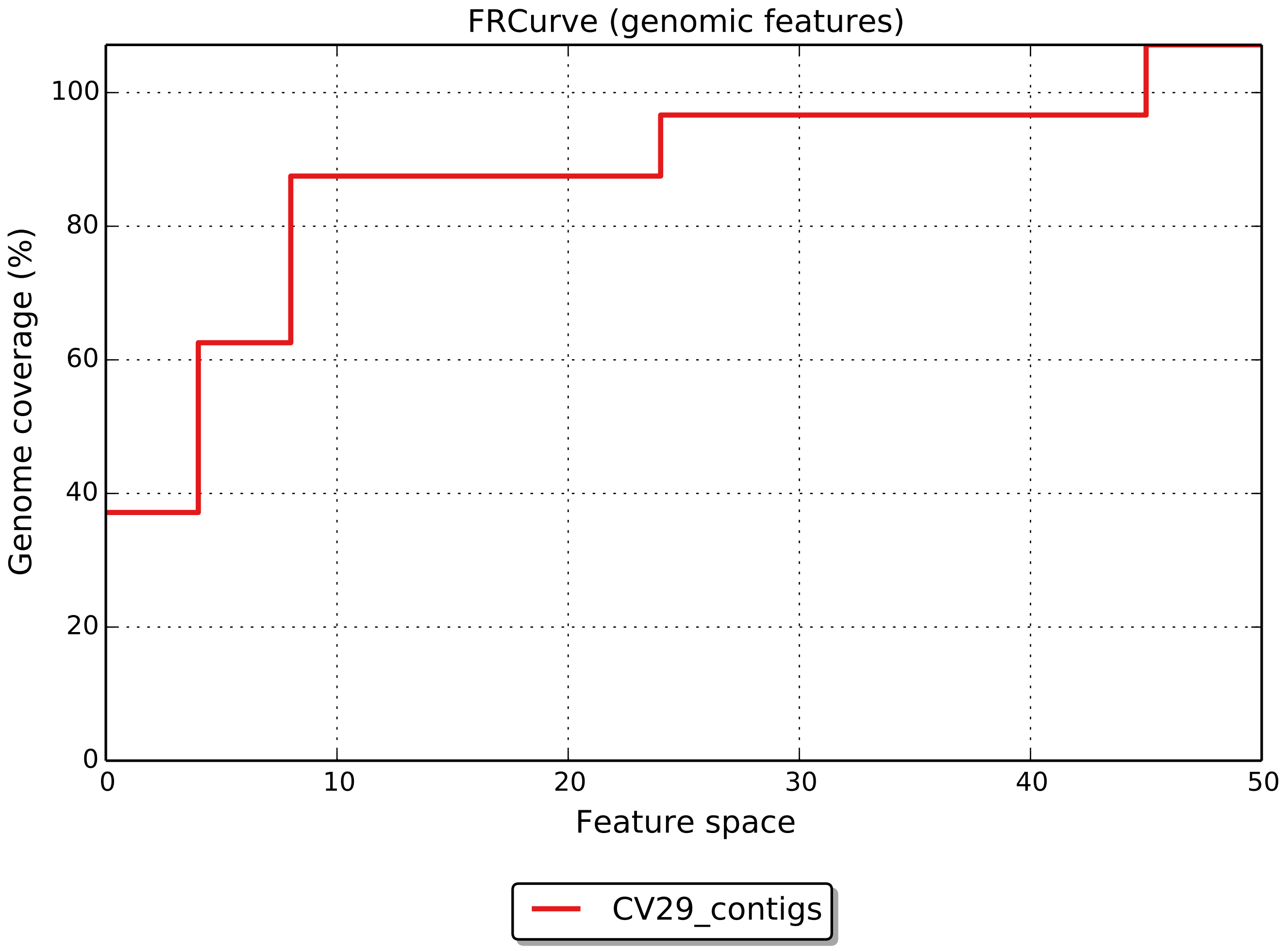

FRCurve (genomic features)
Genome coverage (%)
0
20
40
60
80
100
0
10
20
30
40
50
Feature space
CV29_contigs
