# Supplementary material for: VGEA: an RNA viral assembly toolkit
Source: PeerJ. 2021 Sep 6;9:e12129. doi: 10.7717/peerj.12129 (PMC8428259; doi:10.7717/peerj.12129)
Supplement: Supplemental Information 2 [file peerj-09-12129-s002.zip › Supplementary_File_2/Performance_Comparison_Assembly_Pipelines_Table_2/CV29/spades/quast_results/results_2021_05_14_16_35_34/report.pdf]

Report

	CV29_spades_contigs
# contigs (>= 0 bp)	2165
# contigs (>= 1000 bp)	144
# contigs (>= 5000 bp)	2
# contigs (>= 10000 bp)	2
# contigs (>= 25000 bp)	0
# contigs (>= 50000 bp)	0
Total length (>= 0 bp)	961507
Total length (>= 1000 bp)	283492
Total length (>= 5000 bp)	42733
Total length (>= 10000 bp)	42733
Total length (>= 25000 bp)	0
Total length (>= 50000 bp)	0
# contigs	478
Largest contig	24904
Total length	511049
Reference length	29903
GC (%)	43.71
Reference GC (%)	37.97
N50	1136
NG50	24904
N75	716
NG75	24904
L50	118
LG50	1
L75	262
LG75	1
# misassemblies	0
# misassembled contigs	0
Misassembled contigs length	0
# local misassemblies	0
# scaffold gap ext. mis.	0
# scaffold gap loc. mis.	0
# unaligned mis. contigs	0
# unaligned contigs	476 + 0 part
Unaligned length	481208
Genome fraction (%)	99.632
Duplication ratio	1.002
# N's per 100 kbp	0.00
# mismatches per 100 kbp	23.50
# indels per 100 kbp	0.00
# genomic features	54 + 5 part
Largest alignment	24904
Total aligned length	29793
NGA50	24904
NGA75	24904
LGA50	1
LGA75	1

All statistics are based on contigs of size >= 500 bp, unless otherwise noted (e.g., "# contigs (>= 0 bp)" and "Total length (>= 0 bp)" include all contigs).

Misassemblies report

	CV29_spades_contigs
# misassemblies	0
# contig misassemblies	0
# c. relocations	0
# c. translocations	0
# c. inversions	0
# scaffold misassemblies	0
# s. relocations	0
# s. translocations	0
# s. inversions	0
# misassembled contigs	0
Misassembled contigs length	0
# local misassemblies	0
# scaffold gap ext. mis.	0
# scaffold gap loc. mis.	0
# unaligned mis. contigs	0
# mismatches	7
# indels	0
# indels (<= 5 bp)	0
# indels (> 5 bp)	0
Indels length	0

All statistics are based on contigs of size >= 500 bp, unless otherwise noted (e.g., "# contigs (>= 0 bp)" and "Total length (>= 0 bp)" include all contigs).

Unaligned report

	CV29_spades_contigs
# fully unaligned contigs	476
Fully unaligned length	481208
# partially unaligned contigs	0
Partially unaligned length	0
# N's	0

All statistics are based on contigs of size >= 500 bp, unless otherwise noted (e.g., "# contigs (>= 0 bp)" and "Total length (>= 0 bp)" include all contigs).

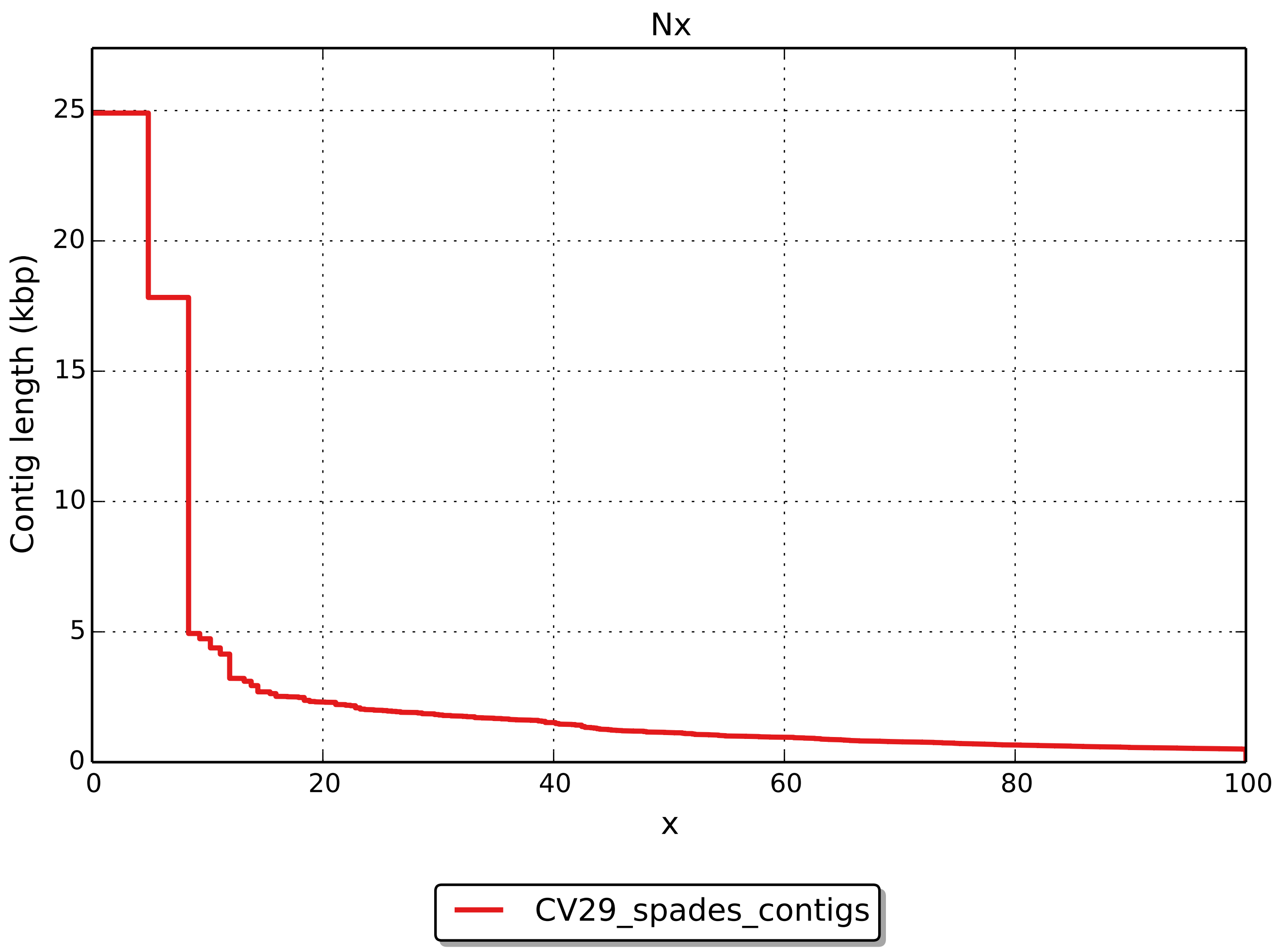

Nx
Contig length (kbp)
25
20
15
10
5
0
0
20
40
60
80
100
x
CV29_spades_contigs

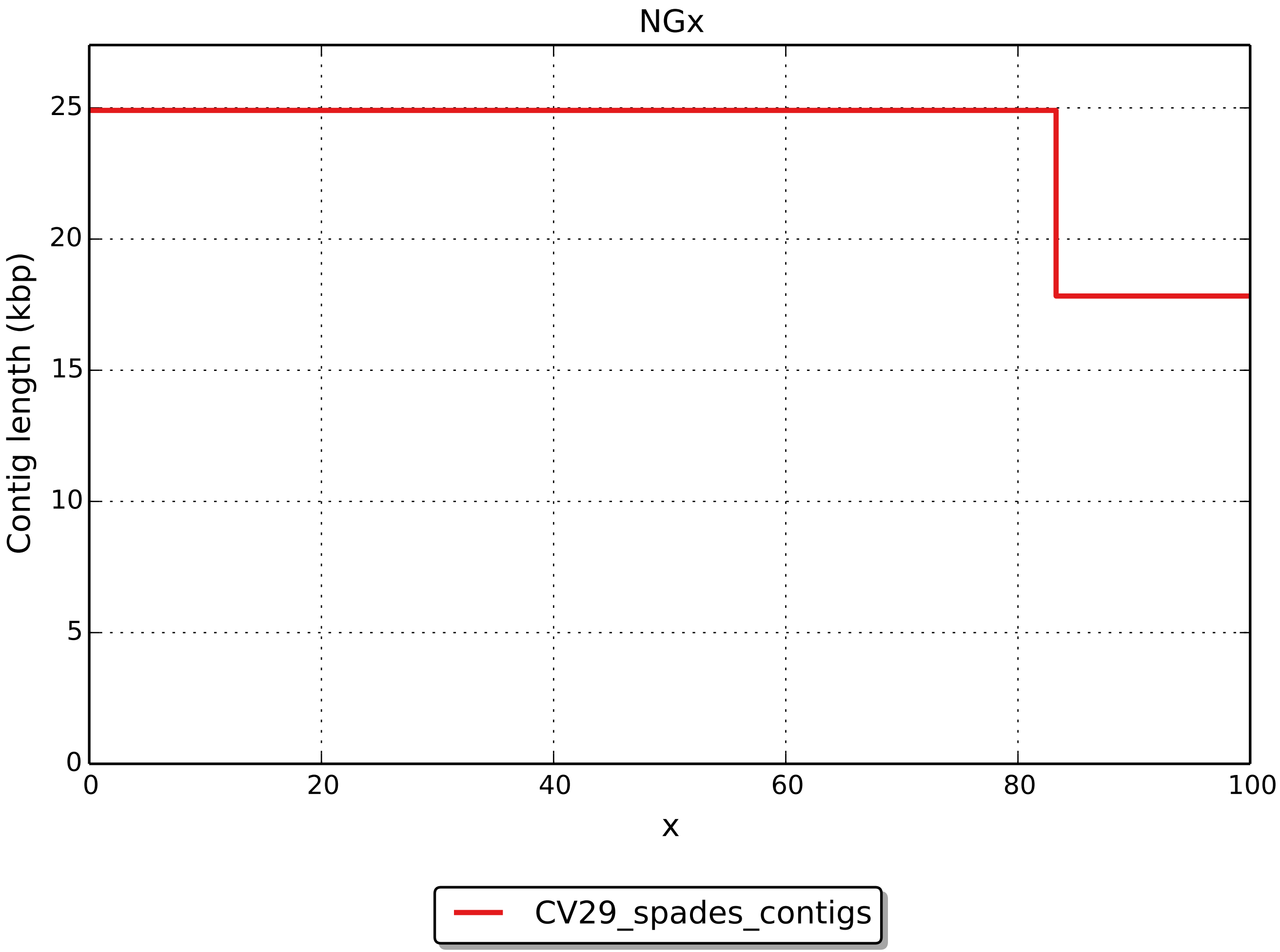

NGx
Contig length (kbp)
0
5
10
15
20
25
0
20
40
60
80
100
x
CV29_spades_contigs

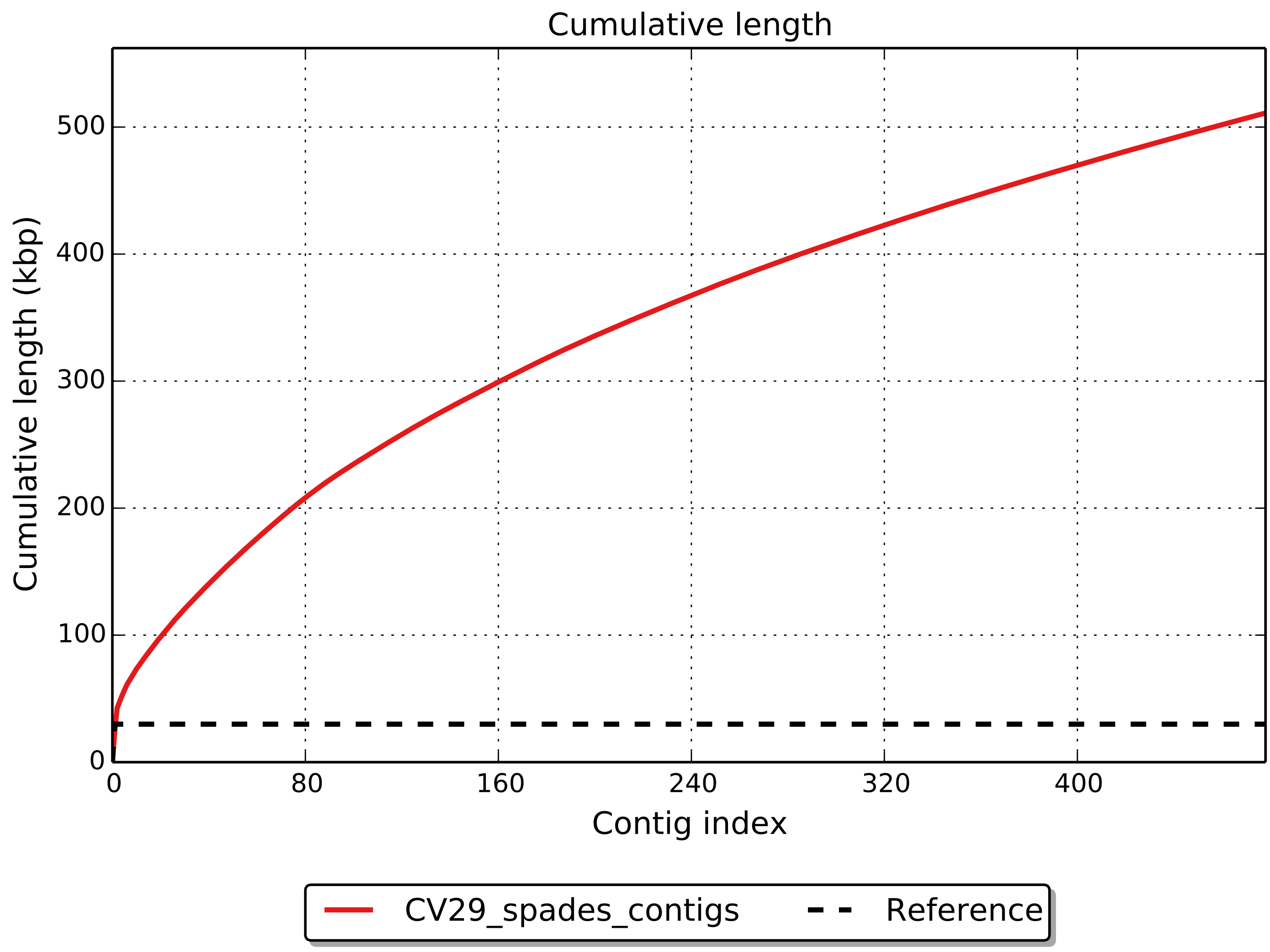

Cumulative length
Cumulative length (kbp)
0
100
200
300
400
500
0
80
160
240
320
400
Contig index
CV29_spades_contigs
Reference

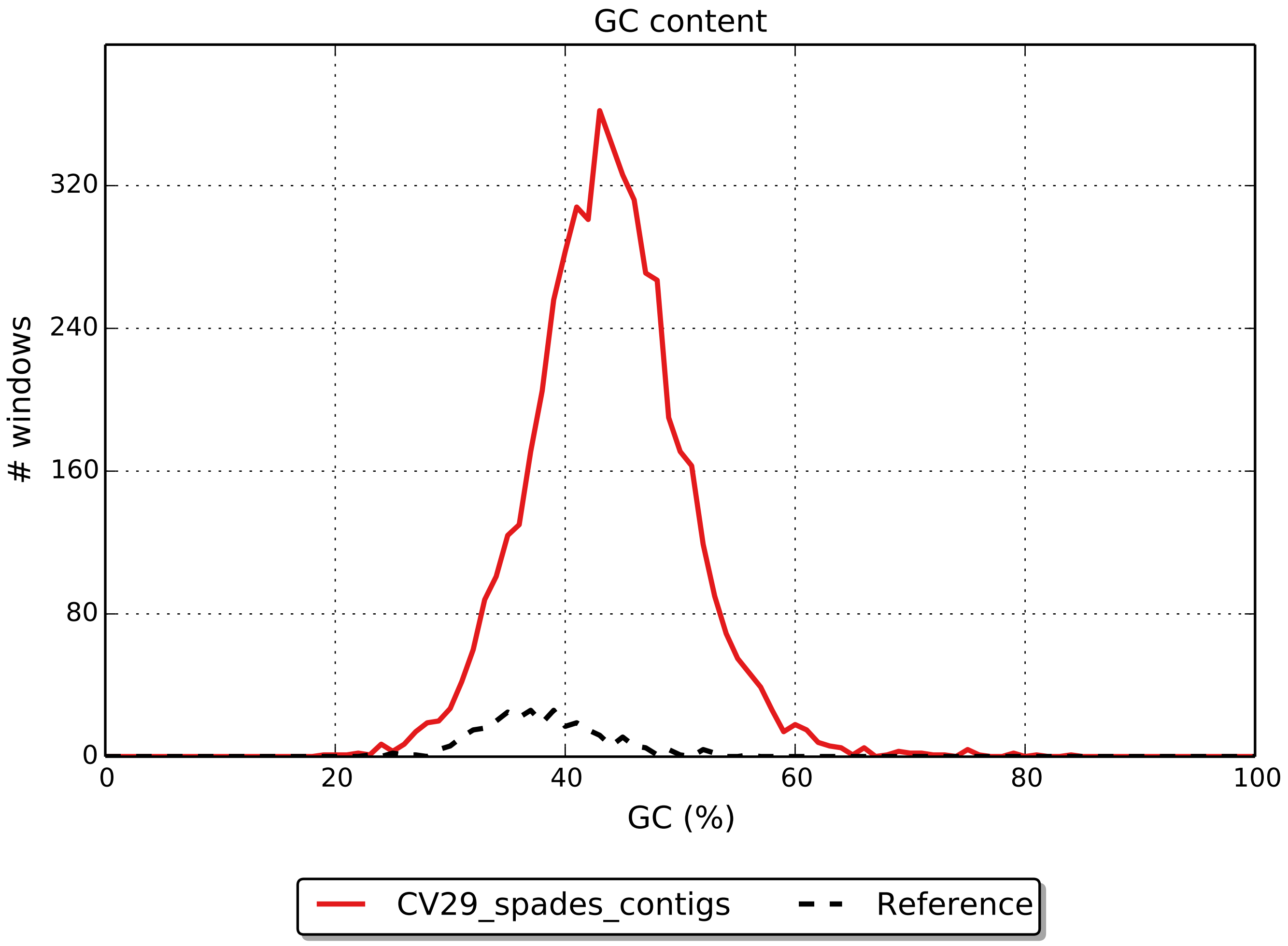

GC content
windows
0
80
160
240
320
0
20
40
60
80
100
GC (%)
CV29_spades_contigs
Reference

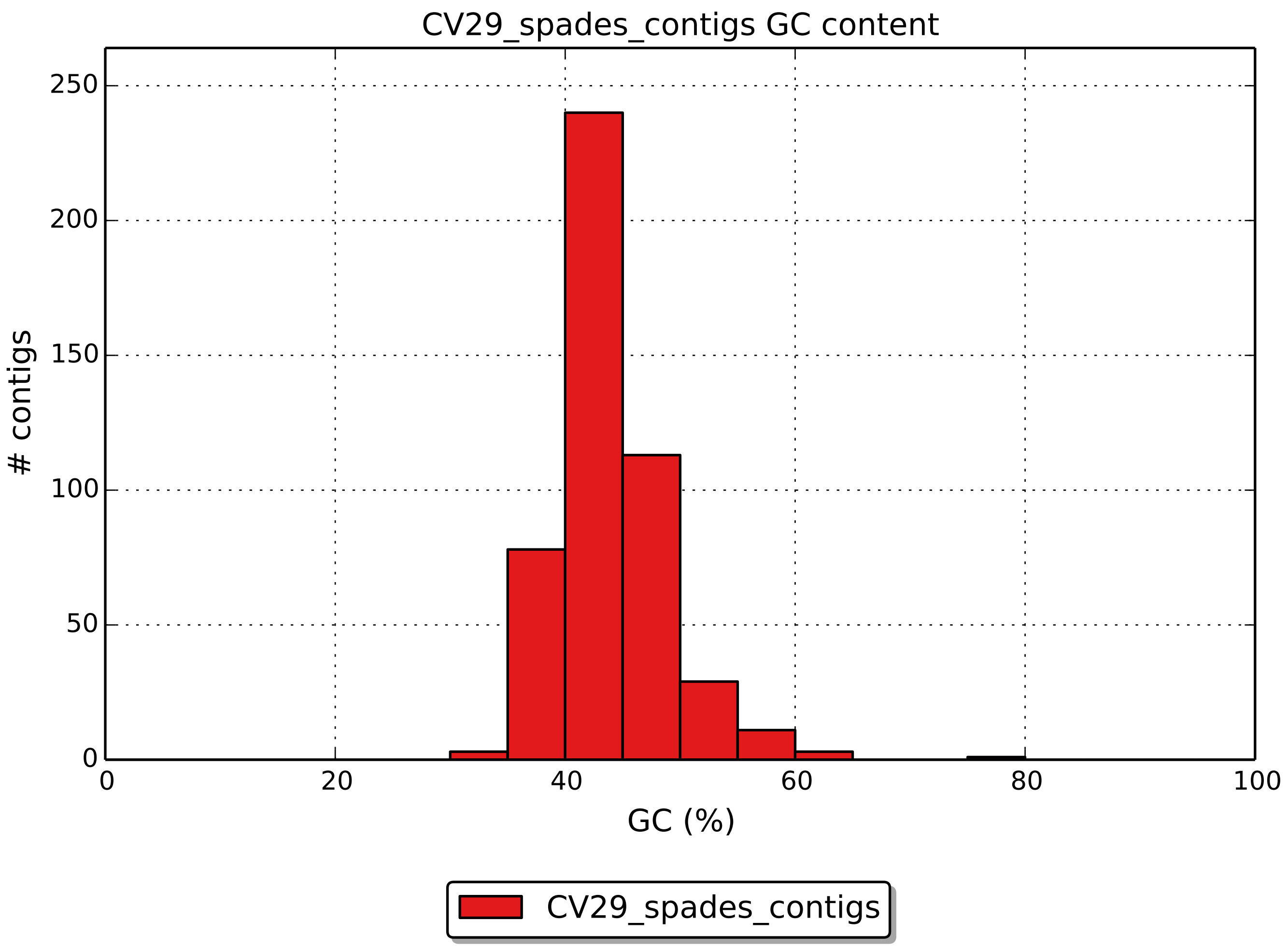
CV29_spades_contigs GC content
contigs
250
200
150
100
50
0
0
20
40
60
80
100
GC (%)
CV29_spades_contigs

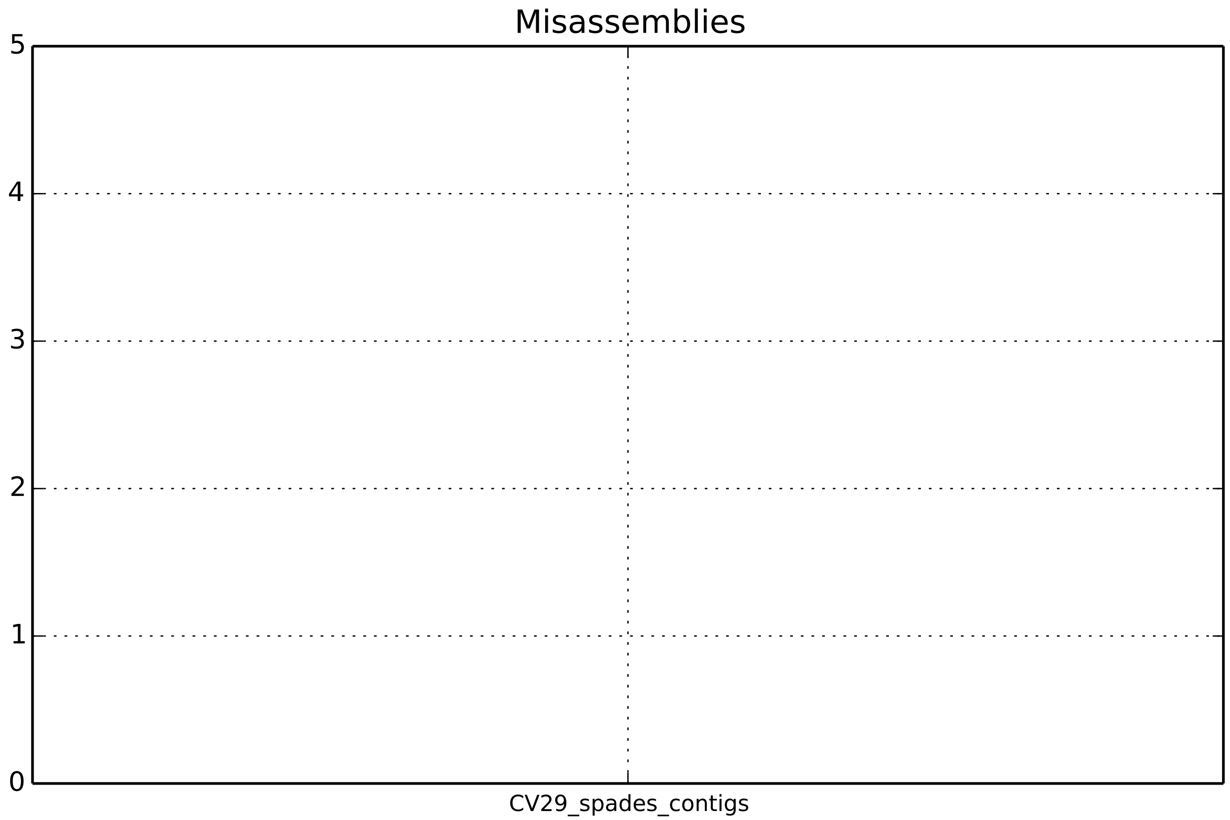

Misassemblies
5
4
3
2
1
0
CV29_spades_contigs

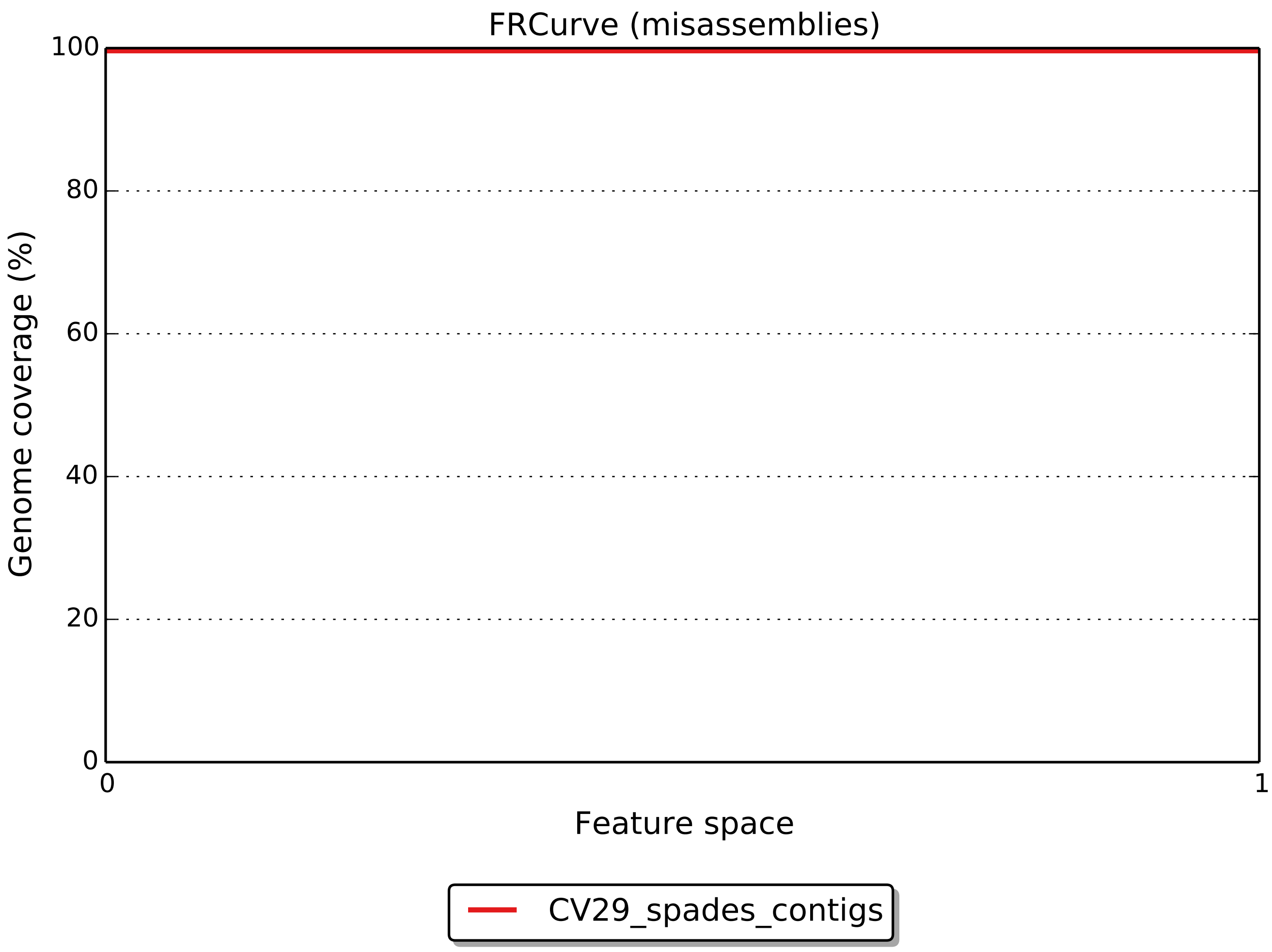

FRCurve (misassemblies)
100
80
60
40
20
0
0
1
Genome coverage (%)
Feature space
CV29_spades_contigs

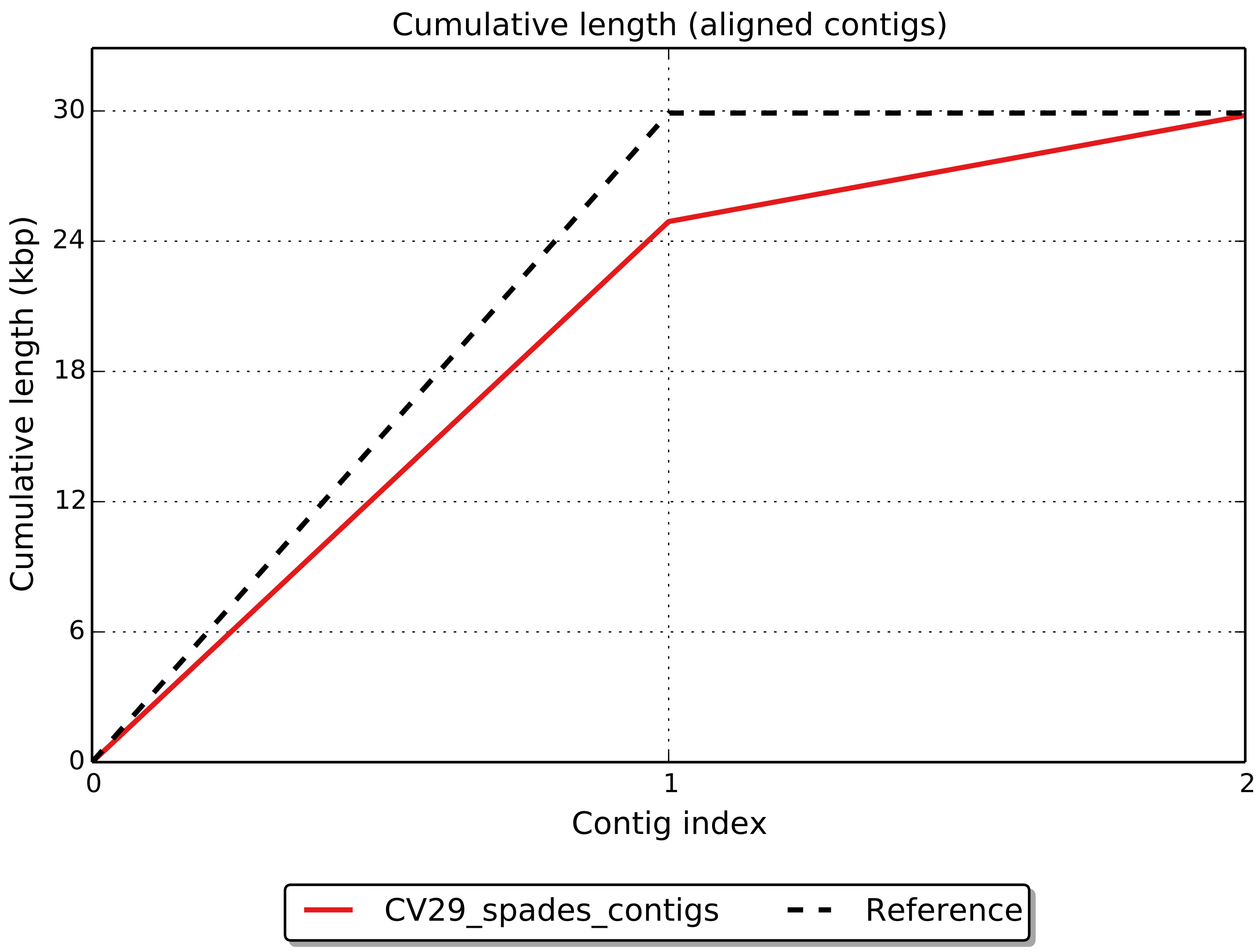

Cumulative length (aligned contigs)
Cumulative length (kbp)
0
6
12
18
24
30
0
1
2
Contig index
CV29_spades_contigs
Reference

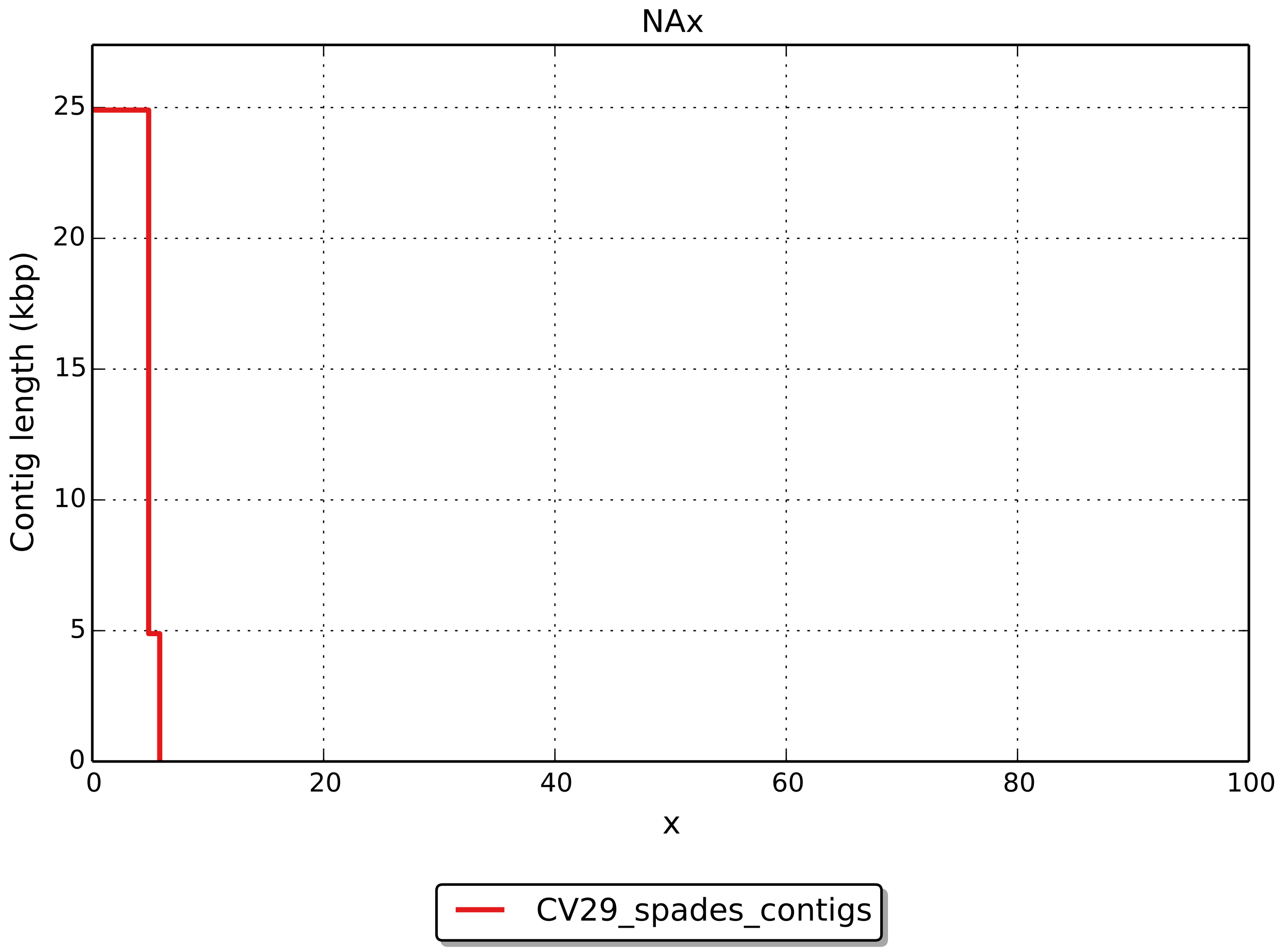

NAx
Contig length (kbp)
0
5
10
15
20
25
0
20
40
60
80
100
x
CV29_spades_contigs

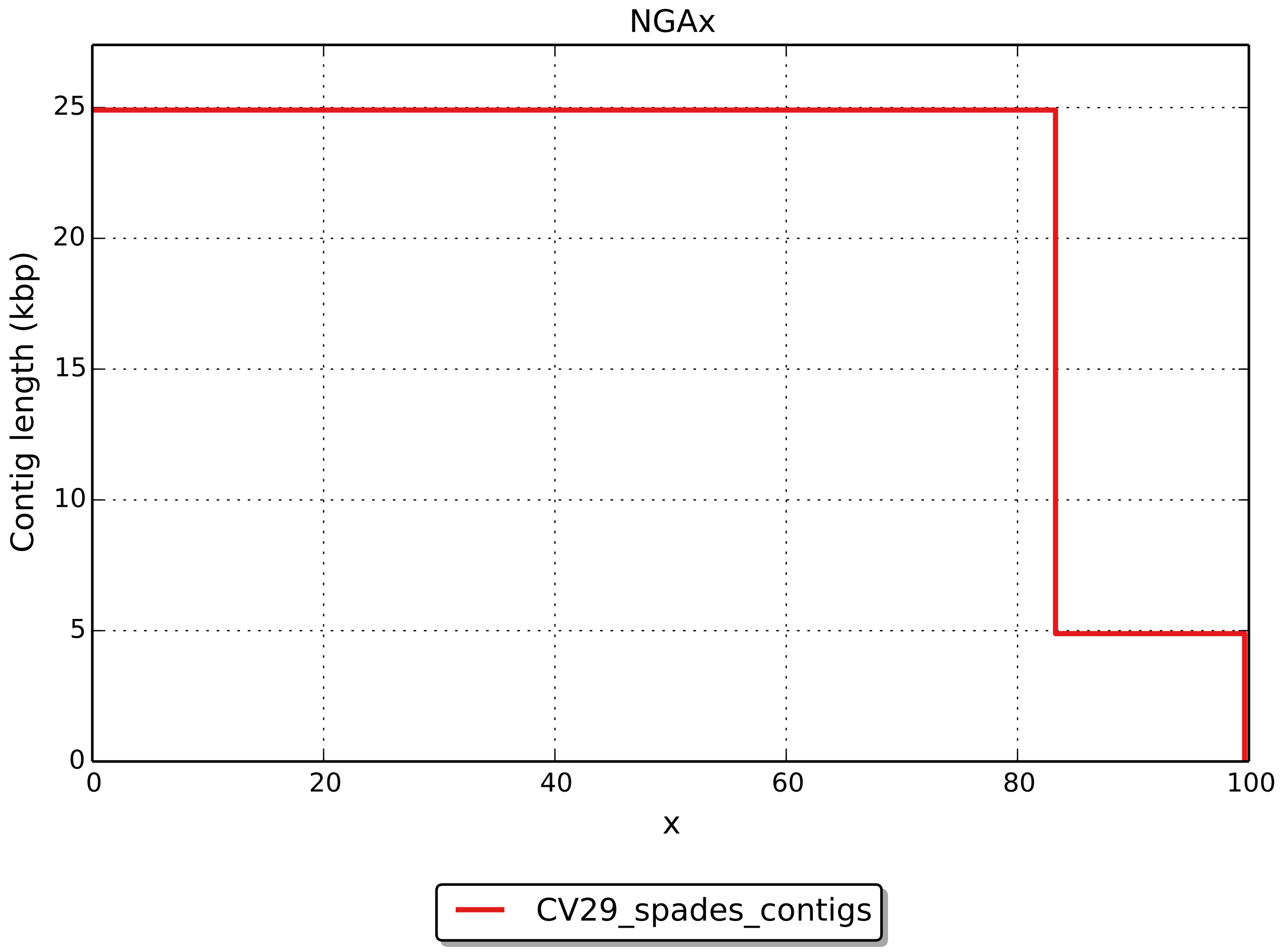
NGAx
Contig length (kbp)
25
20
15
10
5
0
0
20
40
60
80
100
x
CV29_spades_contigs

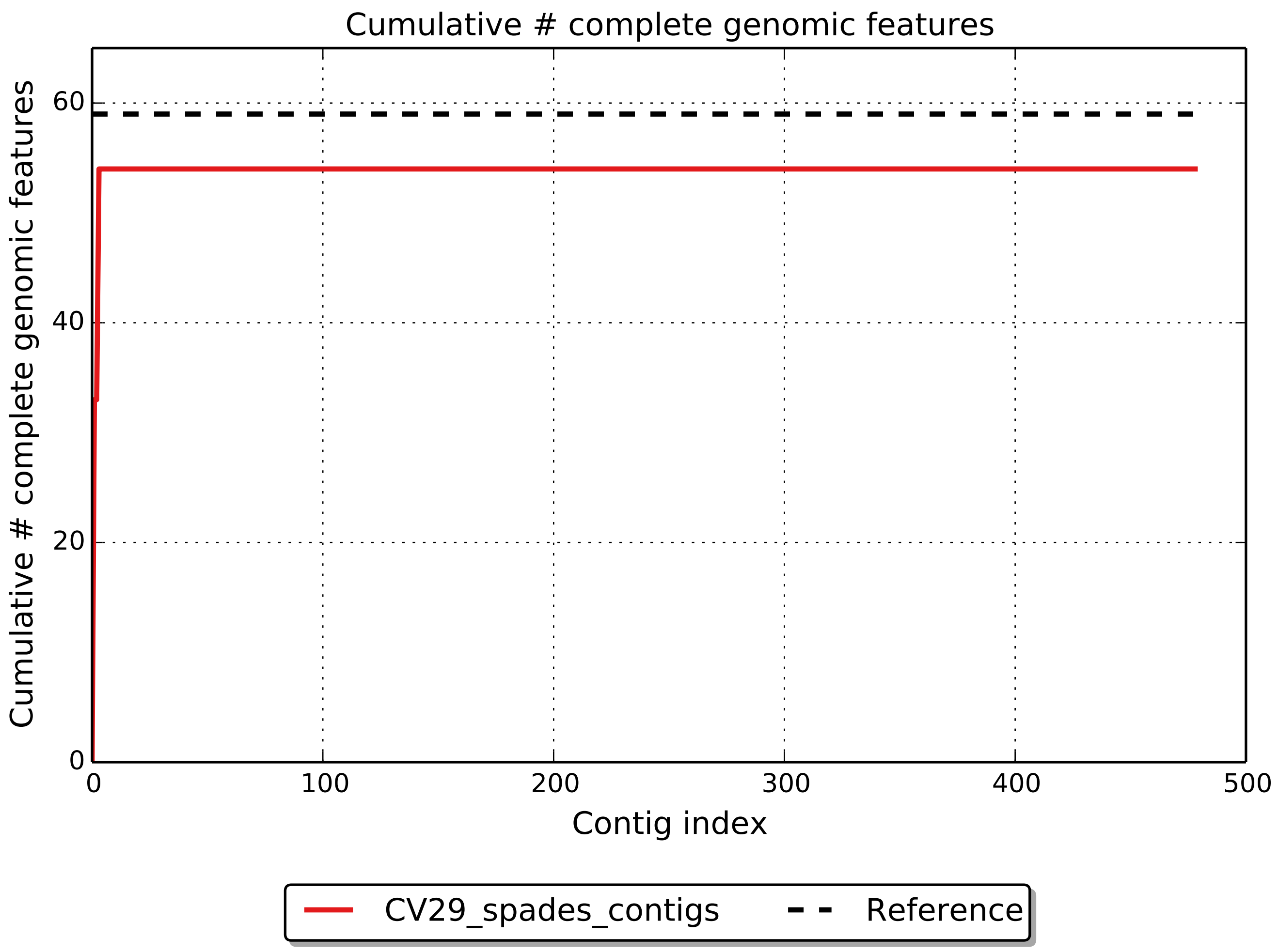

Cumulative # complete genomic features
Cumulative # complete genomic features
0
20
40
60
0
100
200
300
400
500
Contig index
CV29_spades_contigs
Reference

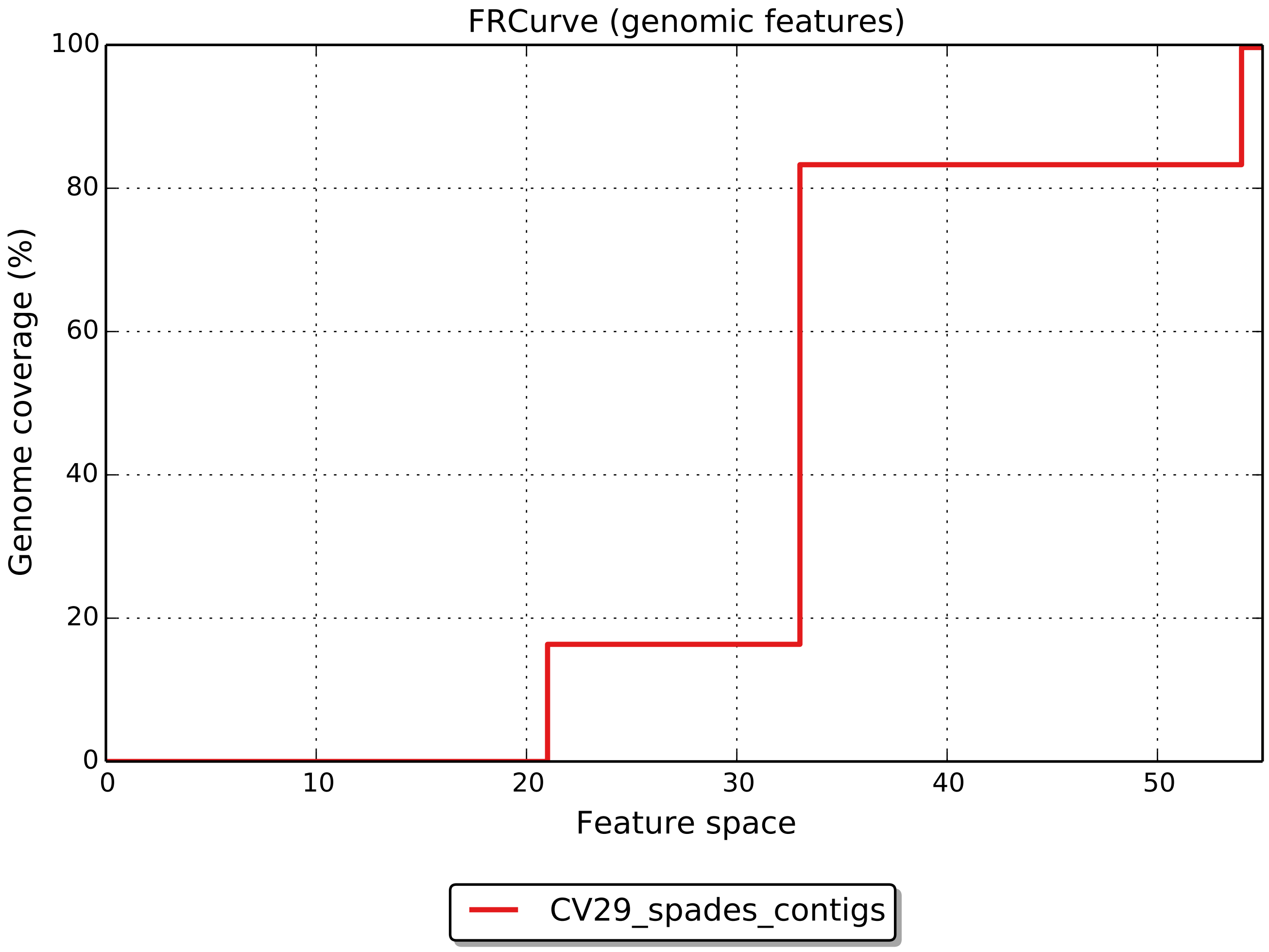
FRCurve (genomic features)
Genome coverage (%)
Feature space
0
20
40
60
80
100
0
10
20
30
40
50
CV29_spades_contigs
