# Supplementary material for: VGEA: an RNA viral assembly toolkit
Source: PeerJ. 2021 Sep 6;9:e12129. doi: 10.7717/peerj.12129 (PMC8428259; doi:10.7717/peerj.12129)
Supplement: Supplemental Information 2 [file peerj-09-12129-s002.zip › Supplementary_File_2/Performance_Comparison_Assembly_Pipelines_Table_2/CV18/velvet/quast_results/results_2021_05_14_01_59_46/report.pdf]

Report

	CV18_velvet_contigs
# contigs (>= 0 bp)	883
# contigs (>= 1000 bp)	8
# contigs (>= 5000 bp)	0
# contigs (>= 10000 bp)	0
# contigs (>= 25000 bp)	0
# contigs (>= 50000 bp)	0
Total length (>= 0 bp)	233772
Total length (>= 1000 bp)	9484
Total length (>= 5000 bp)	0
Total length (>= 10000 bp)	0
Total length (>= 25000 bp)	0
Total length (>= 50000 bp)	0
# contigs	68
Largest contig	1858
Total length	50125
Reference length	29903
GC (%)	47.82
Reference GC (%)	37.97
N50	728
NG50	922
N75	605
NG75	799
L50	27
LG50	14
L75	45
LG75	23
# misassemblies	0
# misassembled contigs	0
Misassembled contigs length	0
# local misassemblies	0
# scaffold gap ext. mis.	0
# scaffold gap loc. mis.	0
# unaligned mis. contigs	0
# unaligned contigs	60 + 0 part
Unaligned length	44197
Genome fraction (%)	19.326
Duplication ratio	1.026
# N's per 100 kbp	297.26
# mismatches per 100 kbp	51.91
# indels per 100 kbp	0.00
# genomic features	0 + 18 part
Largest alignment	1140
Total aligned length	5779
NGA50	-

All statistics are based on contigs of size >= 500 bp, unless otherwise noted (e.g., "# contigs (>= 0 bp)" and "Total length (>= 0 bp)" include all contigs).

Misassemblies report

	CV18_velvet_contigs
# misassemblies	0
# contig misassemblies	0
# c. relocations	0
# c. translocations	0
# c. inversions	0
# scaffold misassemblies	0
# s. relocations	0
# s. translocations	0
# s. inversions	0
# misassembled contigs	0
Misassembled contigs length	0
# local misassemblies	0
# scaffold gap ext. mis.	0
# scaffold gap loc. mis.	0
# unaligned mis. contigs	0
# mismatches	3
# indels	0
# indels (<= 5 bp)	0
# indels (> 5 bp)	0
Indels length	0

All statistics are based on contigs of size >= 500 bp, unless otherwise noted (e.g., "# contigs (>= 0 bp)" and "Total length (>= 0 bp)" include all contigs).

Unaligned report

	CV18_velvet_contigs
# fully unaligned contigs	60
Fully unaligned length	44197
# partially unaligned contigs	0
Partially unaligned length	0
# N's	149

All statistics are based on contigs of size >= 500 bp, unless otherwise noted (e.g., "# contigs (>= 0 bp)" and "Total length (>= 0 bp)" include all contigs).

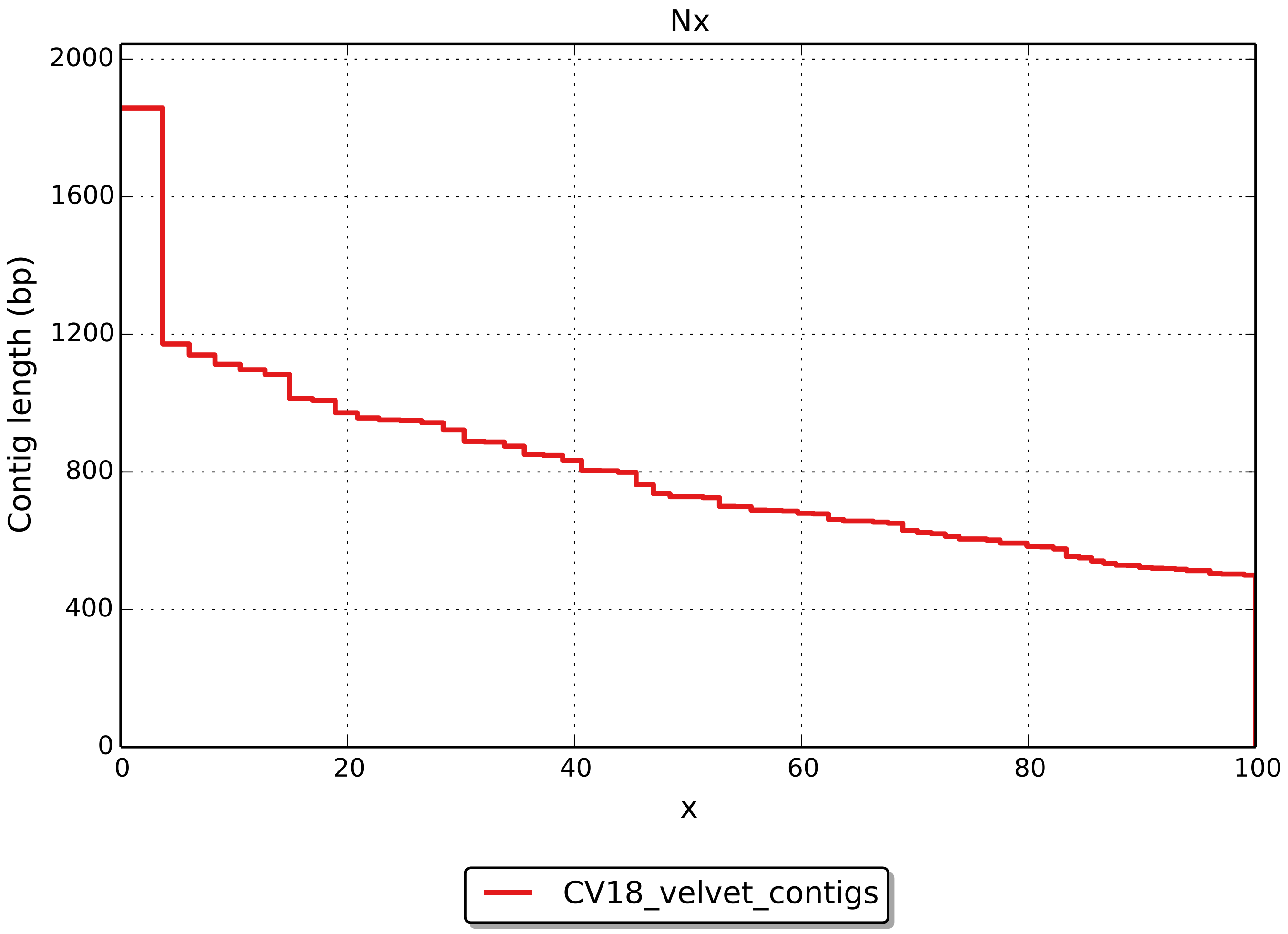

Nx
Contig length (bp)
0
400
800
1200
1600
2000
0
20
40
60
80
100
x
CV18_velvet_contigs

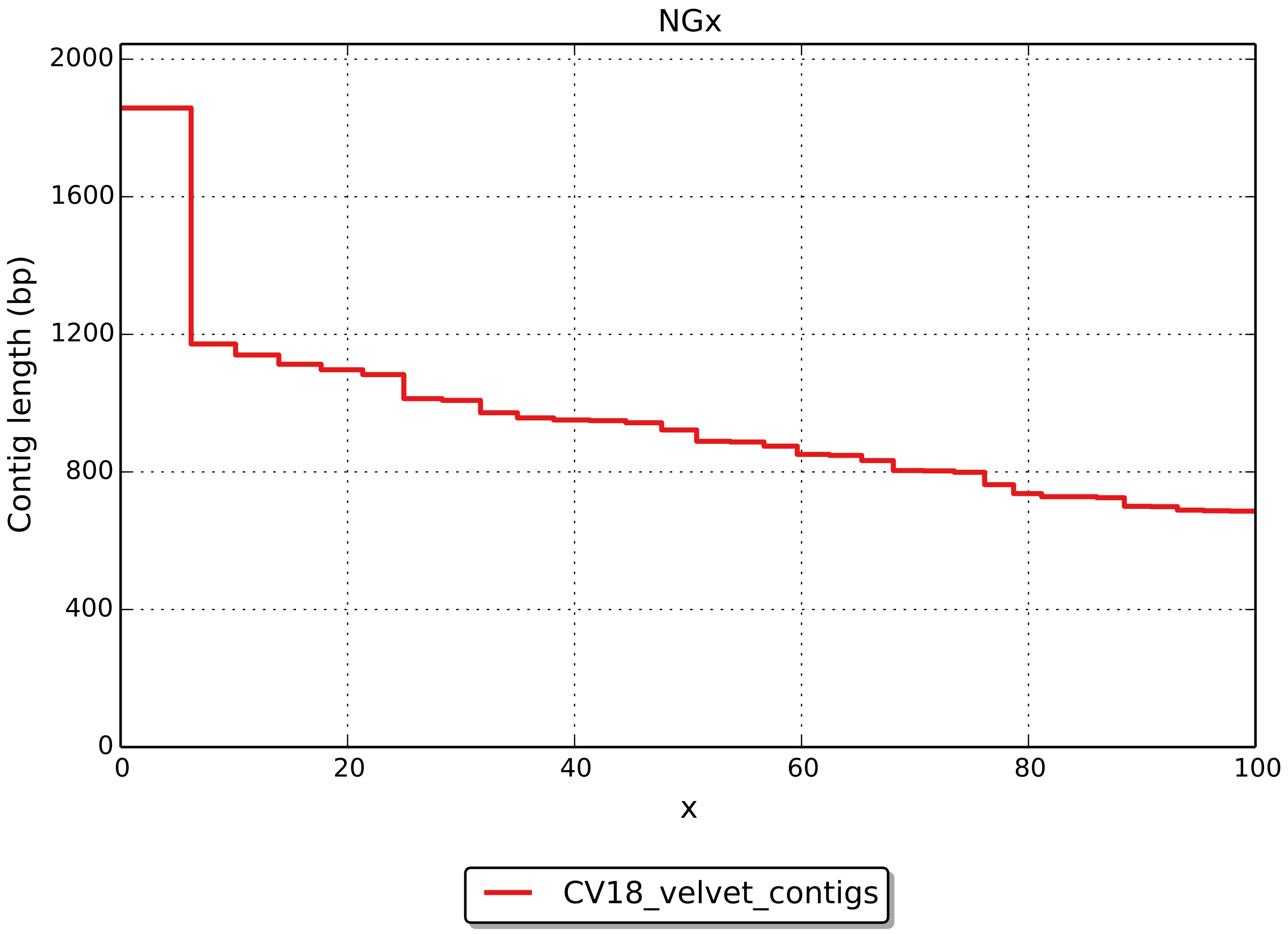
NGx
Contig length (bp)
2000
1600
1200
800
400
0
0
20
40
60
80
100
x
CV18_velvet_contigs

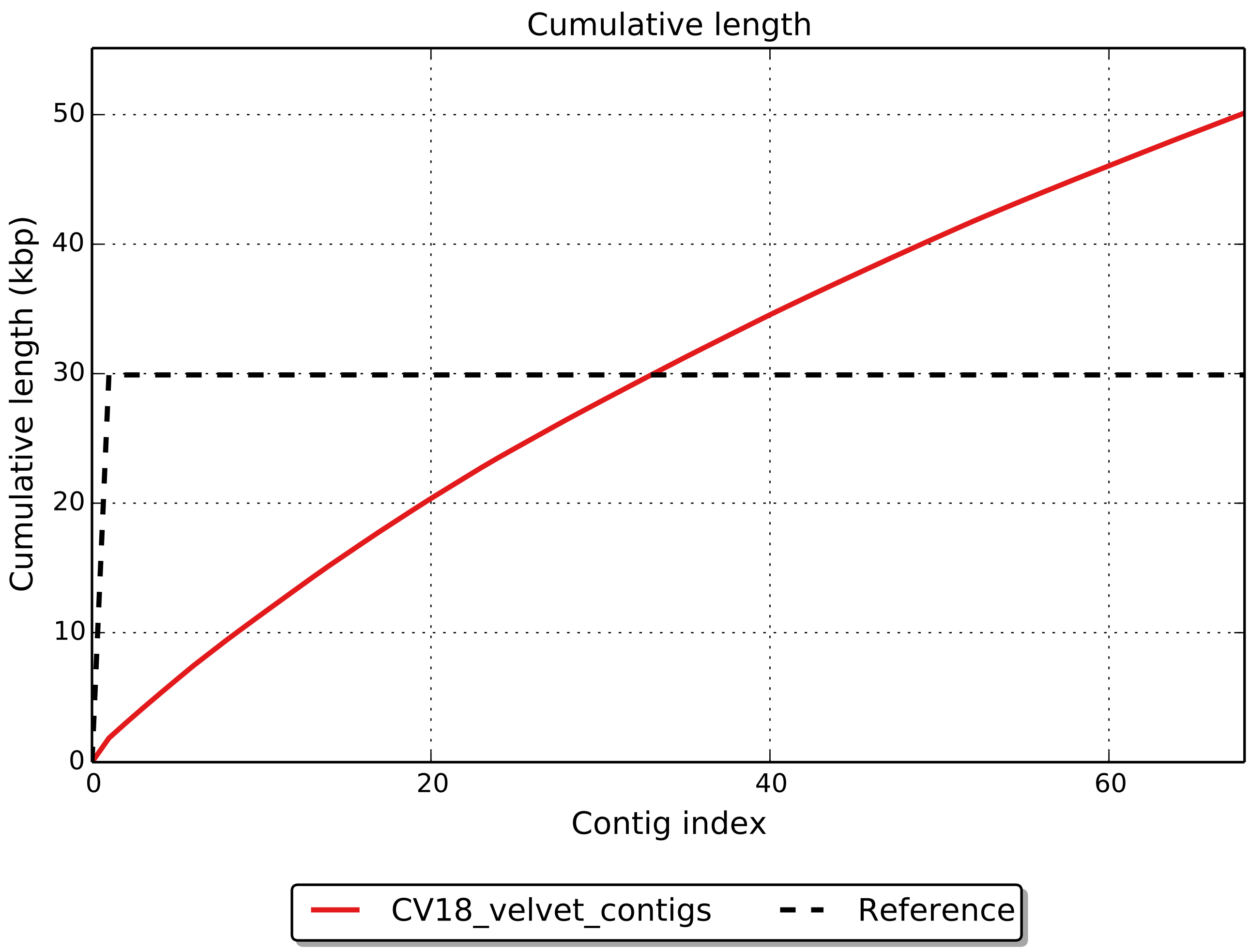
Cumulative length
Cumulative length (kbp)
0
10
20
30
40
50
0
20
40
60
Contig index
CV18_velvet_contigs
Reference

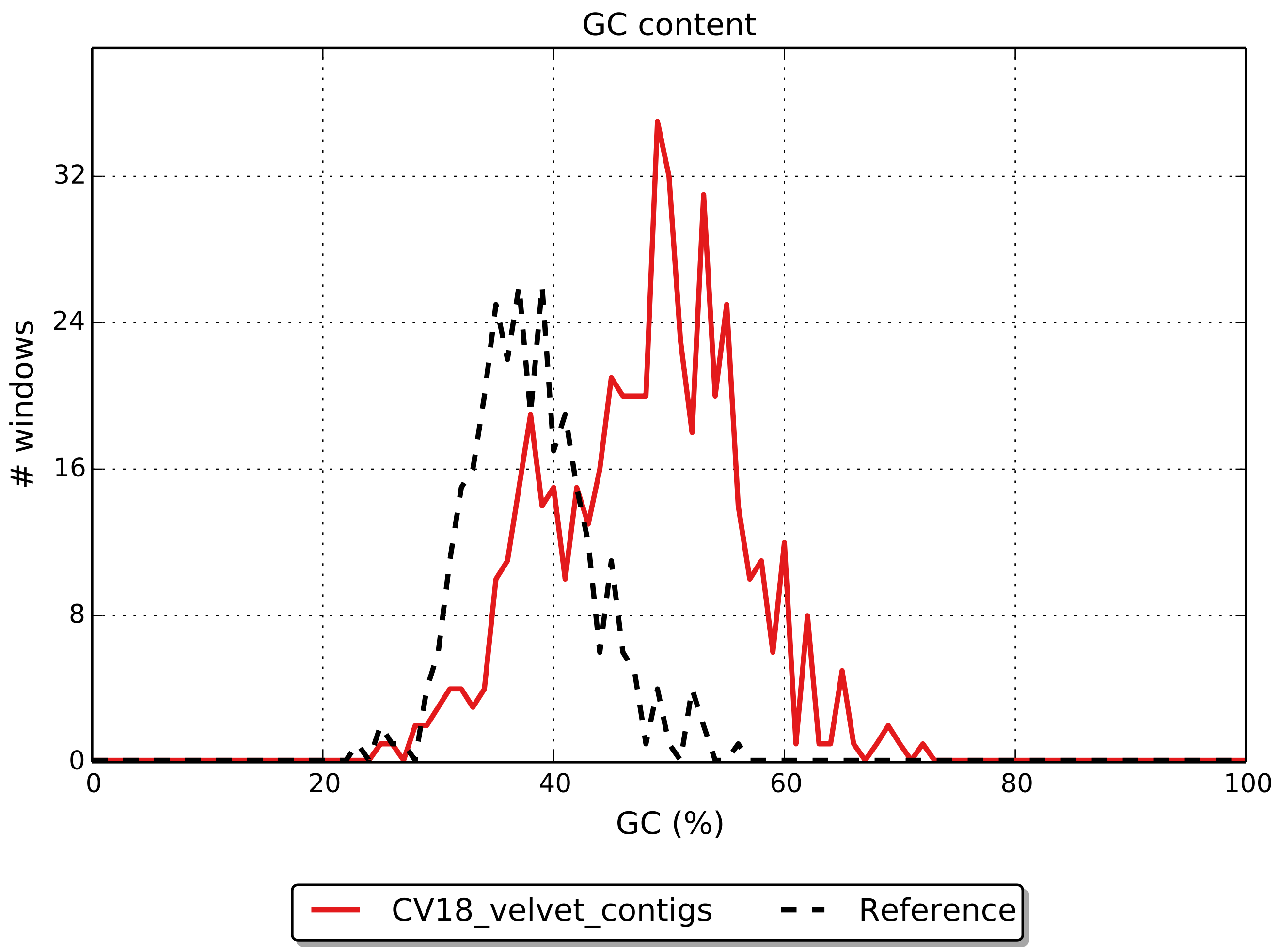

GC content
windows
GC (%)
0
8
16
24
32
0
20
40
60
80
100
CV18_velvet_contigs
Reference

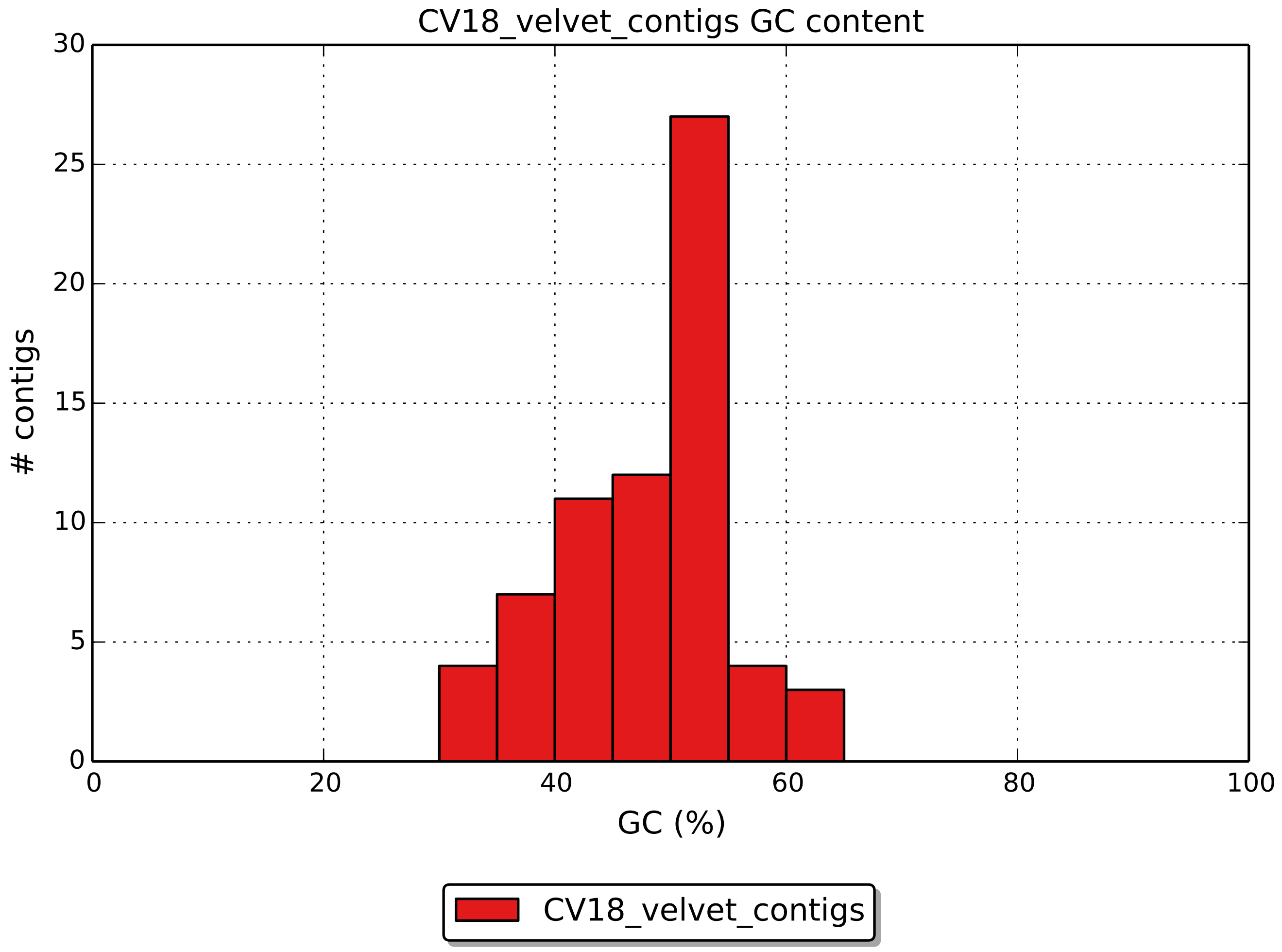

CV18_velvet_contigs GC content
contigs
GC (%)
0
5
10
15
20
25
30
0
20
40
60
80
100
CV18_velvet_contigs

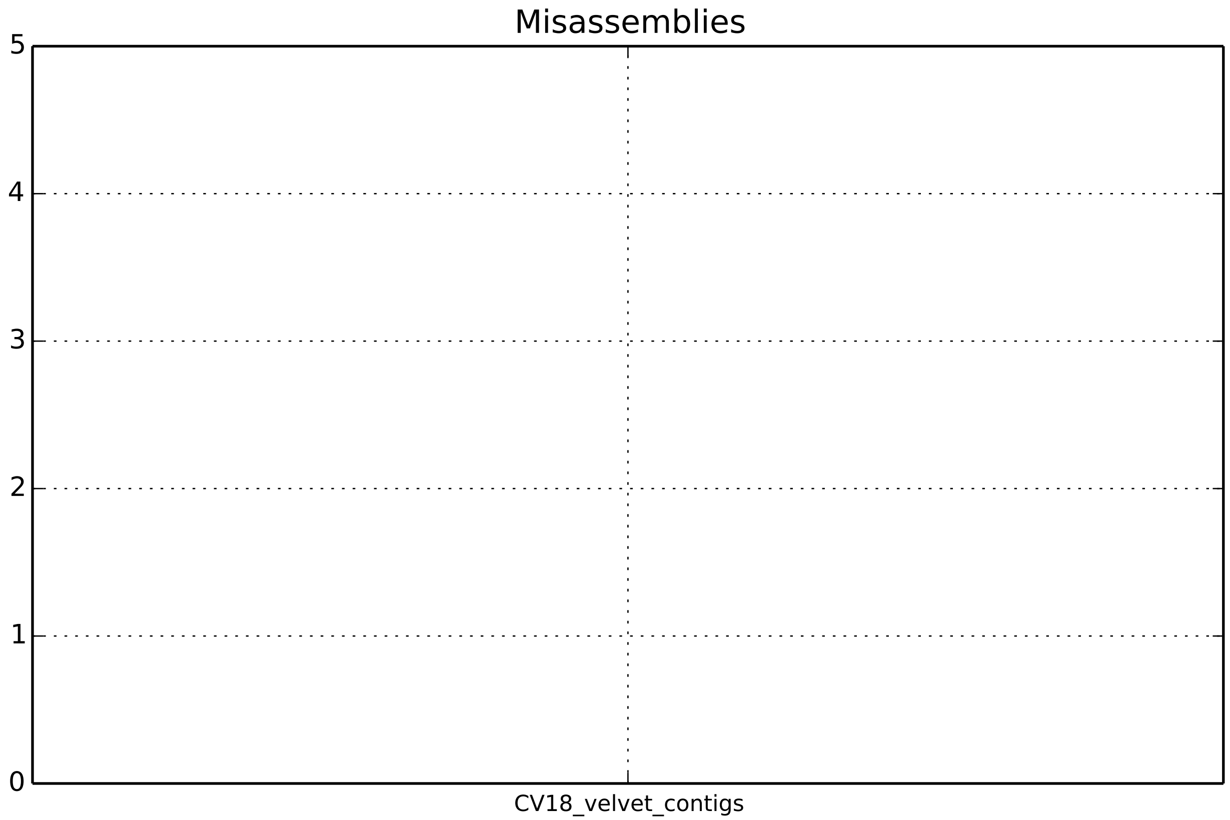

Misassemblies
5
4
3
2
1
0
CV18_velvet_contigs

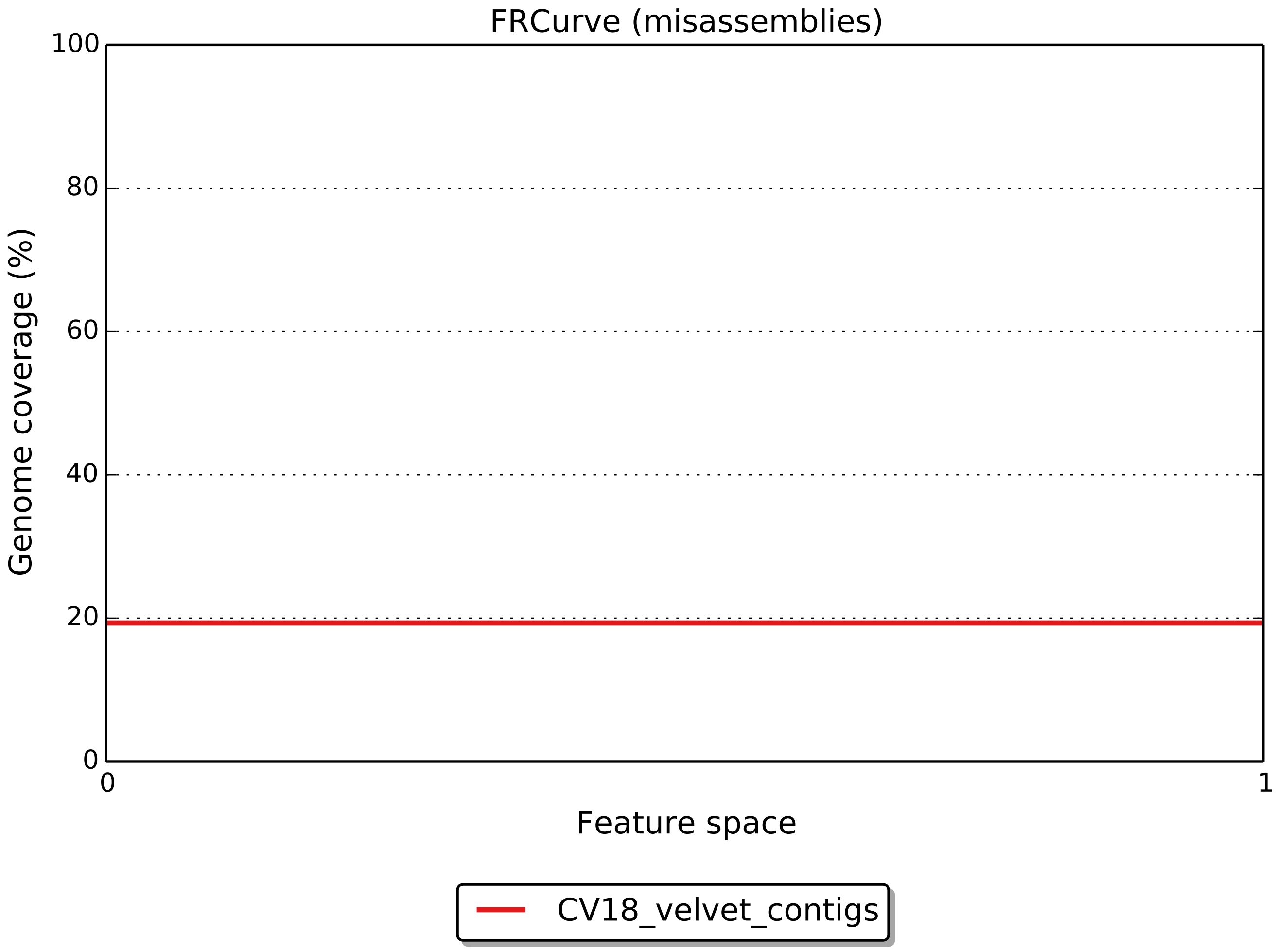
FRCurve (misassemblies)
Genome coverage (%)
100
80
60
40
20
0
0
1
Feature space
CV18_velvet_contigs

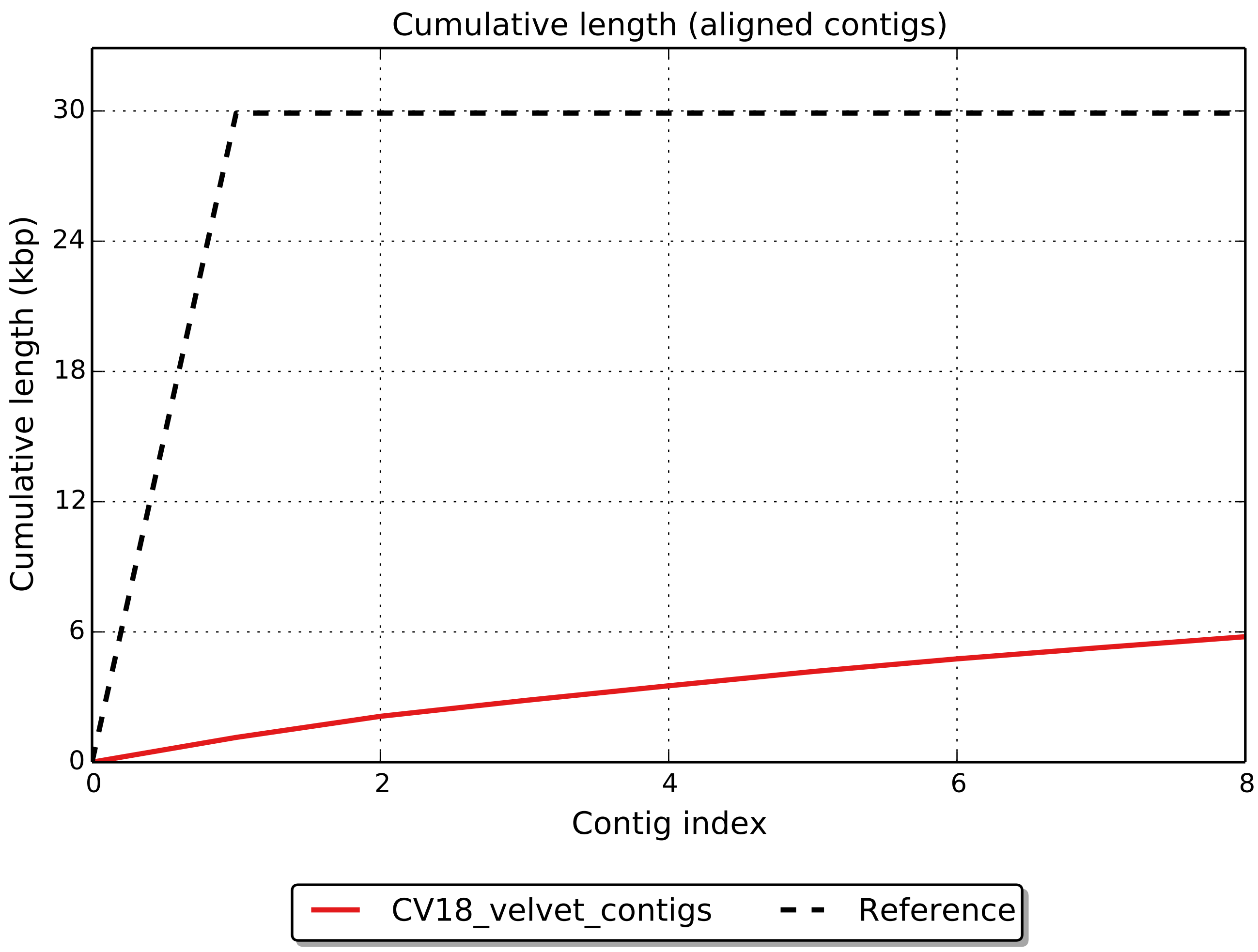
Cumulative length (aligned contigs)
Cumulative length (kbp)
0
6
12
18
24
30
0
2
4
6
8
Contig index
CV18_velvet_contigs
Reference

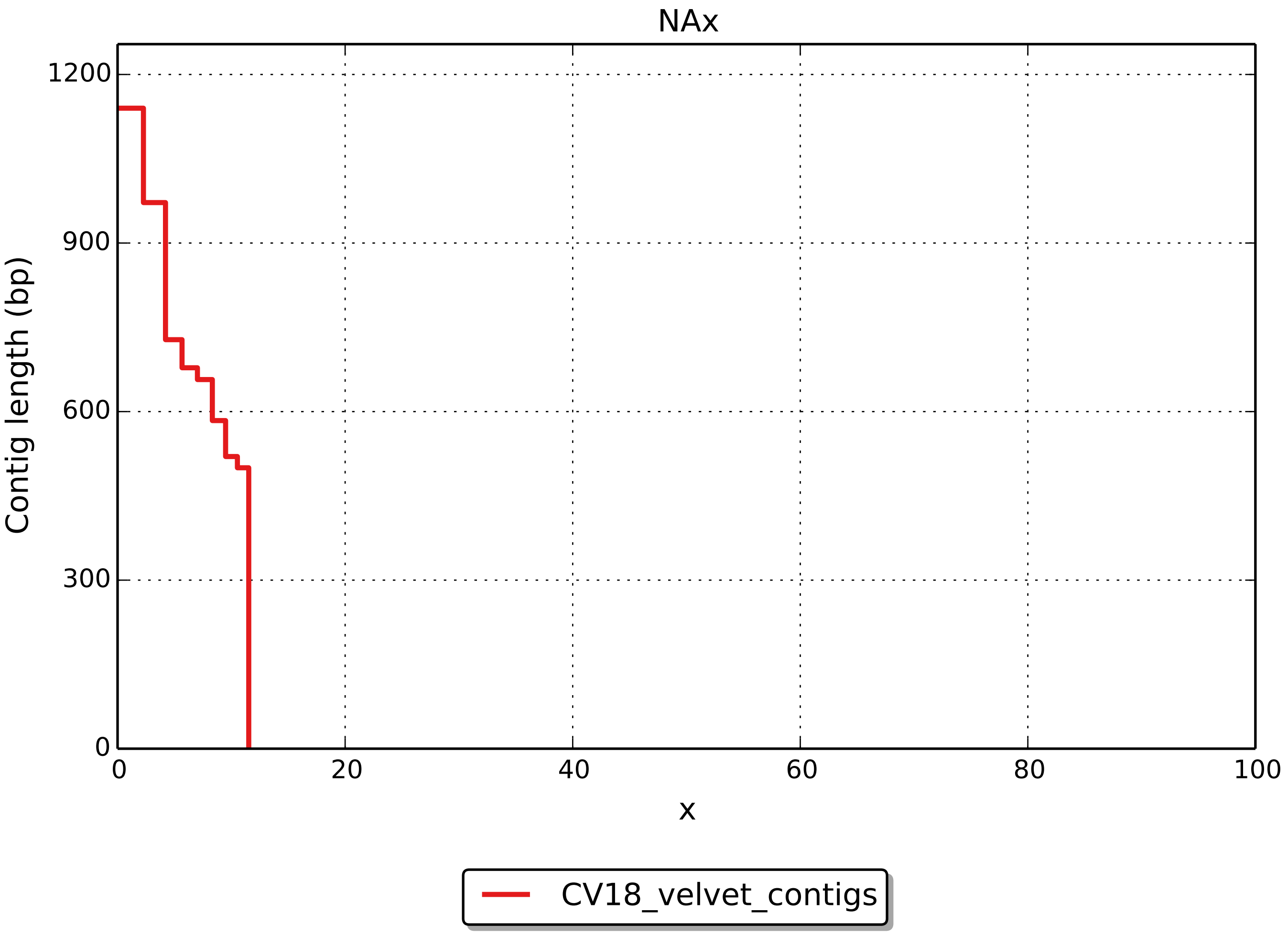
NAx
1200
900
600
300
0
0
20
40
60
80
100
x
Contig length (bp)
CV18_velvet_contigs

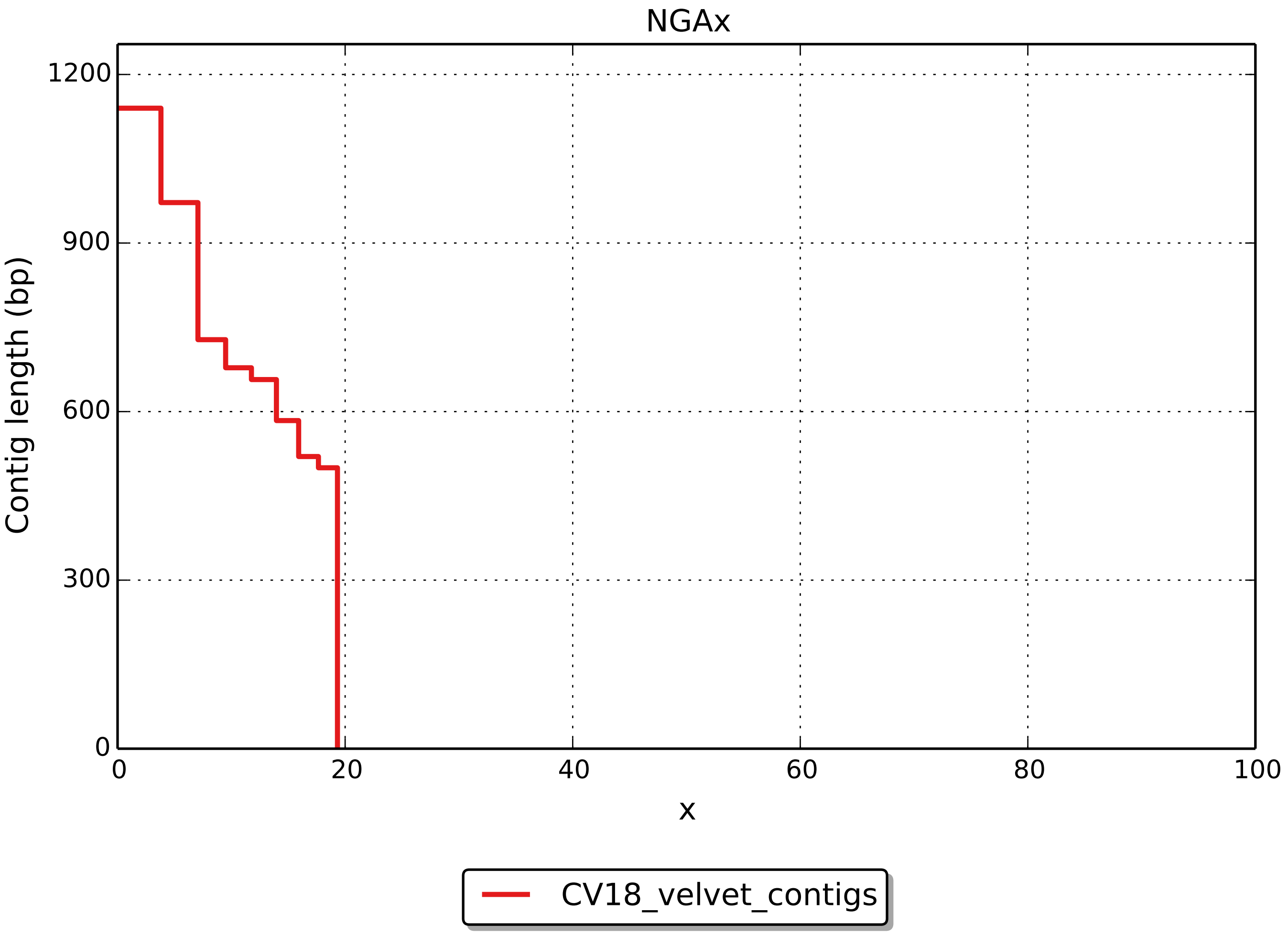
NGAx
Contig length (bp)
1200
900
600
300
0
0
20
40
60
80
100
x
CV18_velvet_contigs

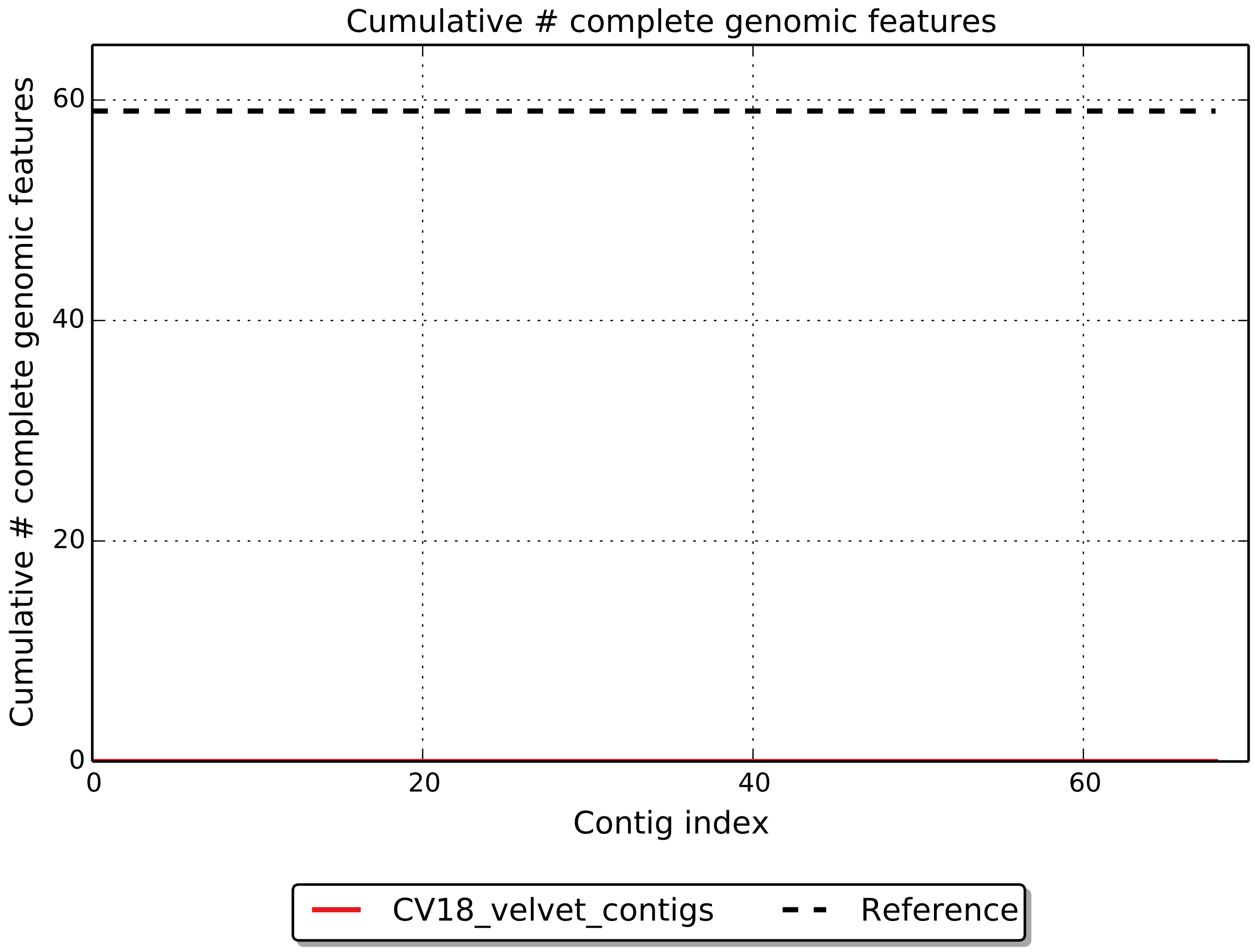

Cumulative # complete genomic features
Cumulative # complete genomic features
0
20
40
60
0
20
40
60
Contig index
CV18_velvet_contigs
Reference

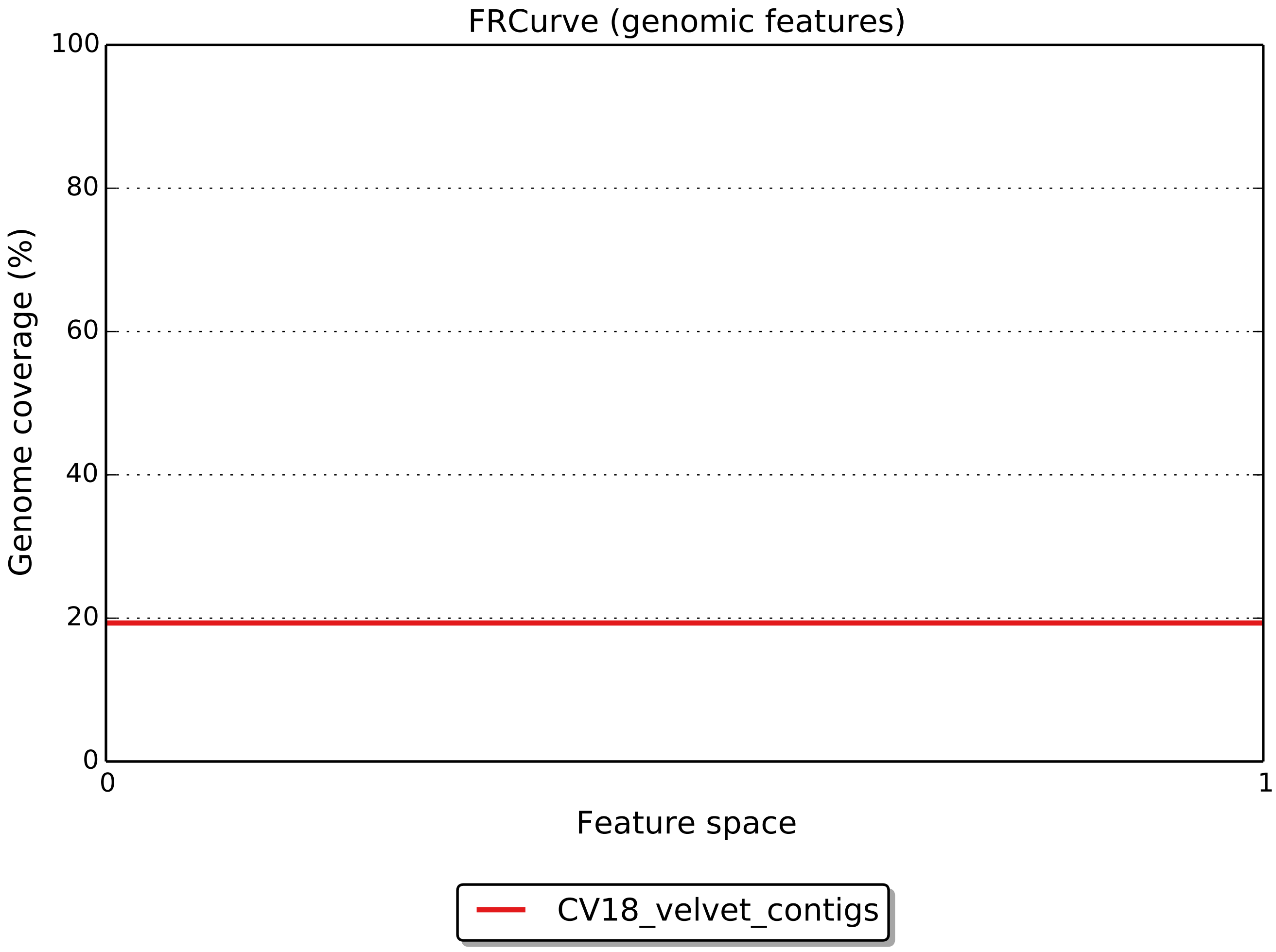
FRCurve (genomic features)
Genome coverage (%)
100
80
60
40
20
0
0
1
Feature space
CV18_velvet_contigs
