# Supplementary material for: VGEA: an RNA viral assembly toolkit
Source: PeerJ. 2021 Sep 6;9:e12129. doi: 10.7717/peerj.12129 (PMC8428259; doi:10.7717/peerj.12129)
Supplement: Supplemental Information 2 [file peerj-09-12129-s002.zip › Supplementary_File_2/Performance_Comparison_Assembly_Pipelines_Table_2/CV18/vgea/quast_results/results_2021_05_13_22_45_01/report.pdf]

Report

	CV18_contigs
# contigs (>= 0 bp)	42
# contigs (>= 1000 bp)	29
# contigs (>= 5000 bp)	2
# contigs (>= 10000 bp)	1
# contigs (>= 25000 bp)	1
# contigs (>= 50000 bp)	0
Total length (>= 0 bp)	86685
Total length (>= 1000 bp)	76625
Total length (>= 5000 bp)	34976
Total length (>= 10000 bp)	29928
Total length (>= 25000 bp)	29928
Total length (>= 50000 bp)	0
# contigs	42
Largest contig	29928
Total length	86685
Reference length	29903
GC (%)	46.97
Reference GC (%)	37.97
N50	2294
NG50	29928
N75	1247
NG75	29928
L50	6
LG50	1
L75	19
LG75	1
# misassemblies	0
# misassembled contigs	0
Misassembled contigs length	0
# local misassemblies	0
# scaffold gap ext. mis.	0
# scaffold gap loc. mis.	0
# unaligned mis. contigs	0
# unaligned contigs	41 + 0 part
Unaligned length	56757
Genome fraction (%)	99.776
Duplication ratio	1.003
# N's per 100 kbp	0.00
# mismatches per 100 kbp	33.52
# indels per 100 kbp	0.00
# genomic features	56 + 3 part
Largest alignment	29836
Total aligned length	29836
NGA50	29836
NGA75	29836
LGA50	1
LGA75	1

All statistics are based on contigs of size >= 500 bp, unless otherwise noted (e.g., "# contigs (>= 0 bp)" and "Total length (>= 0 bp)" include all contigs).

Misassemblies report

	CV18_contigs
# misassemblies	0
# contig misassemblies	0
# c. relocations	0
# c. translocations	0
# c. inversions	0
# scaffold misassemblies	0
# s. relocations	0
# s. translocations	0
# s. inversions	0
# misassembled contigs	0
Misassembled contigs length	0
# local misassemblies	0
# scaffold gap ext. mis.	0
# scaffold gap loc. mis.	0
# unaligned mis. contigs	0
# mismatches	10
# indels	0
# indels (<= 5 bp)	0
# indels (> 5 bp)	0
Indels length	0

All statistics are based on contigs of size >= 500 bp, unless otherwise noted (e.g., "# contigs (>= 0 bp)" and "Total length (>= 0 bp)" include all contigs).

Unaligned report

	CV18_contigs
# fully unaligned contigs	41
Fully unaligned length	56757
# partially unaligned contigs	0
Partially unaligned length	0
# N's	0

All statistics are based on contigs of size >= 500 bp, unless otherwise noted (e.g., "# contigs (>= 0 bp)" and "Total length (>= 0 bp)" include all contigs).

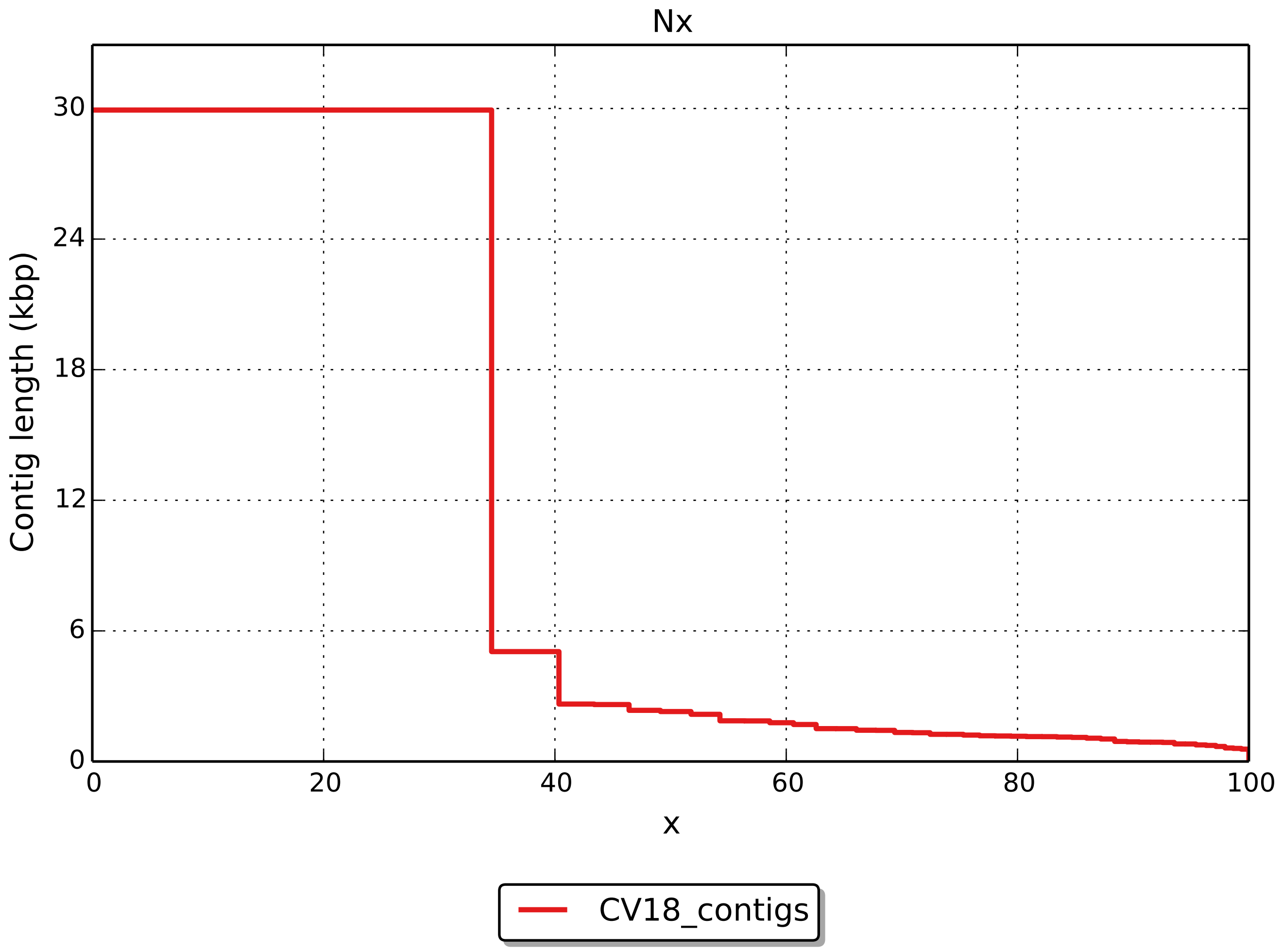
Nx
Contig length (kbp)
30
24
18
12
6
0
0
20
40
60
80
100
x
CV18_contigs

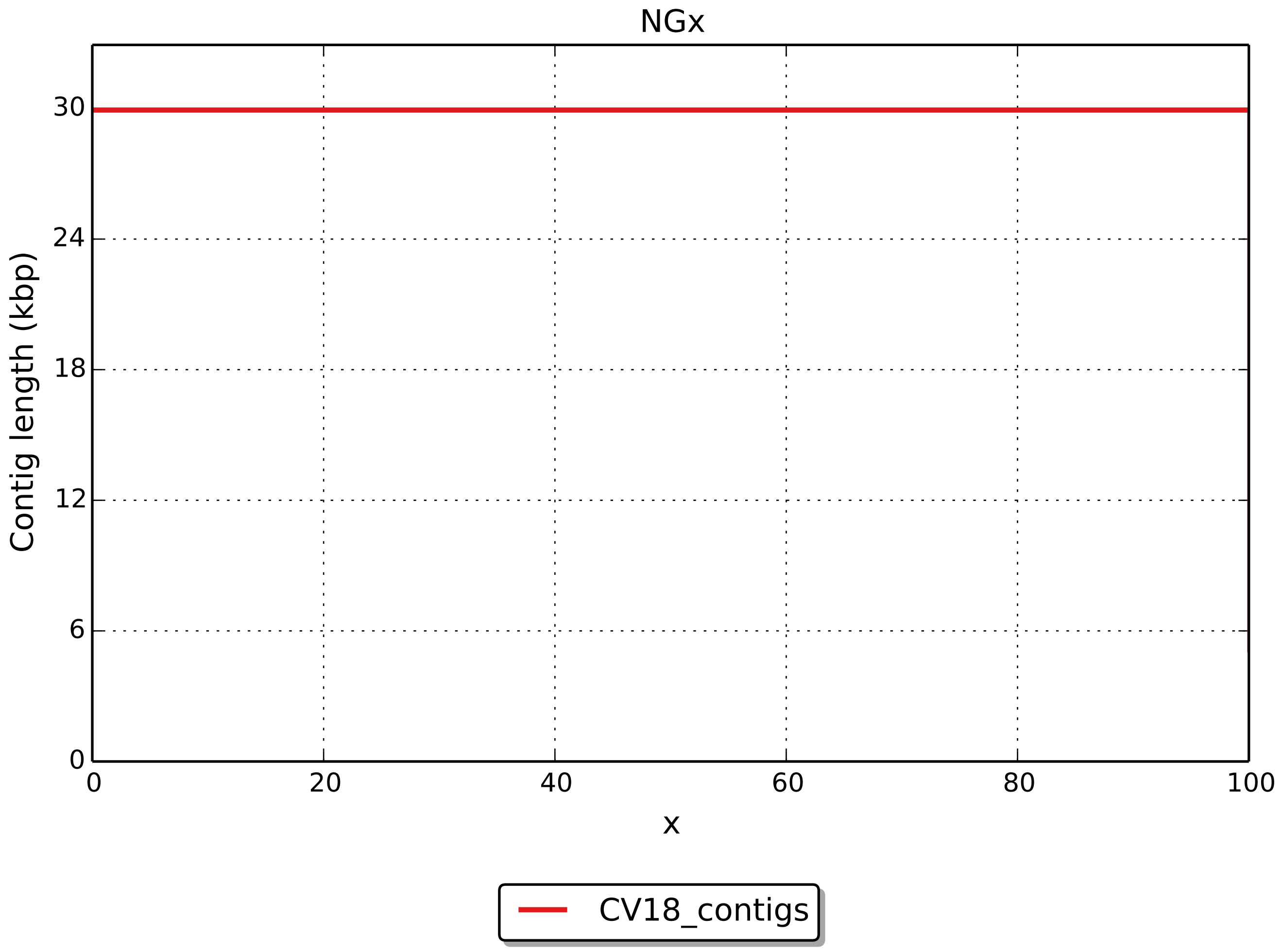
NGx
Contig length (kbp)
30
24
18
12
6
0
0
20
40
60
80
100
x
CV18_contigs

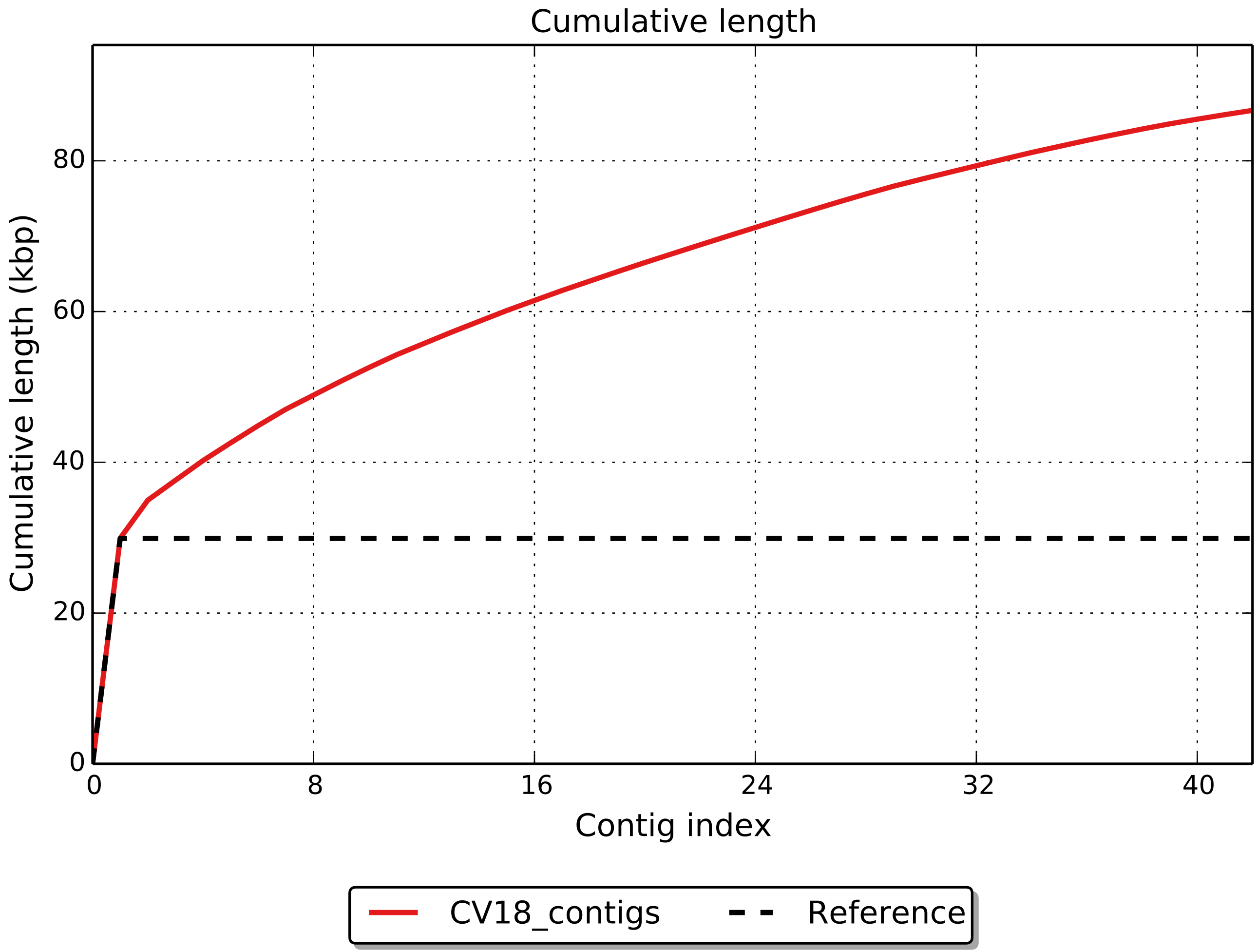
Cumulative length
Cumulative length (kbp)
0
20
40
60
80
0
8
16
24
32
40
Contig index
CV18_contigs
Reference

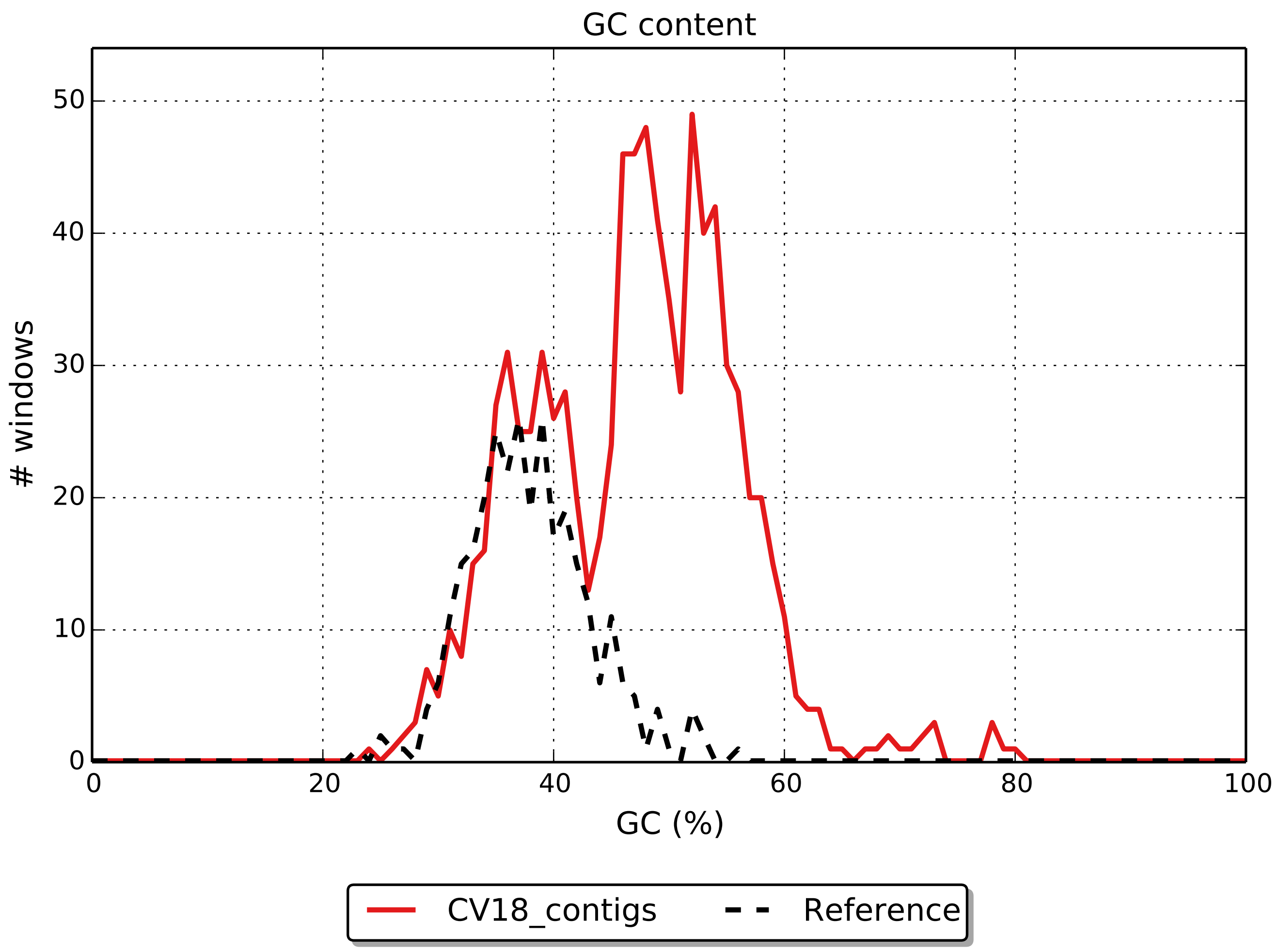

GC content
windows
GC (%)
0
10
20
30
40
50
0
20
40
60
80
100
CV18_contigs
Reference

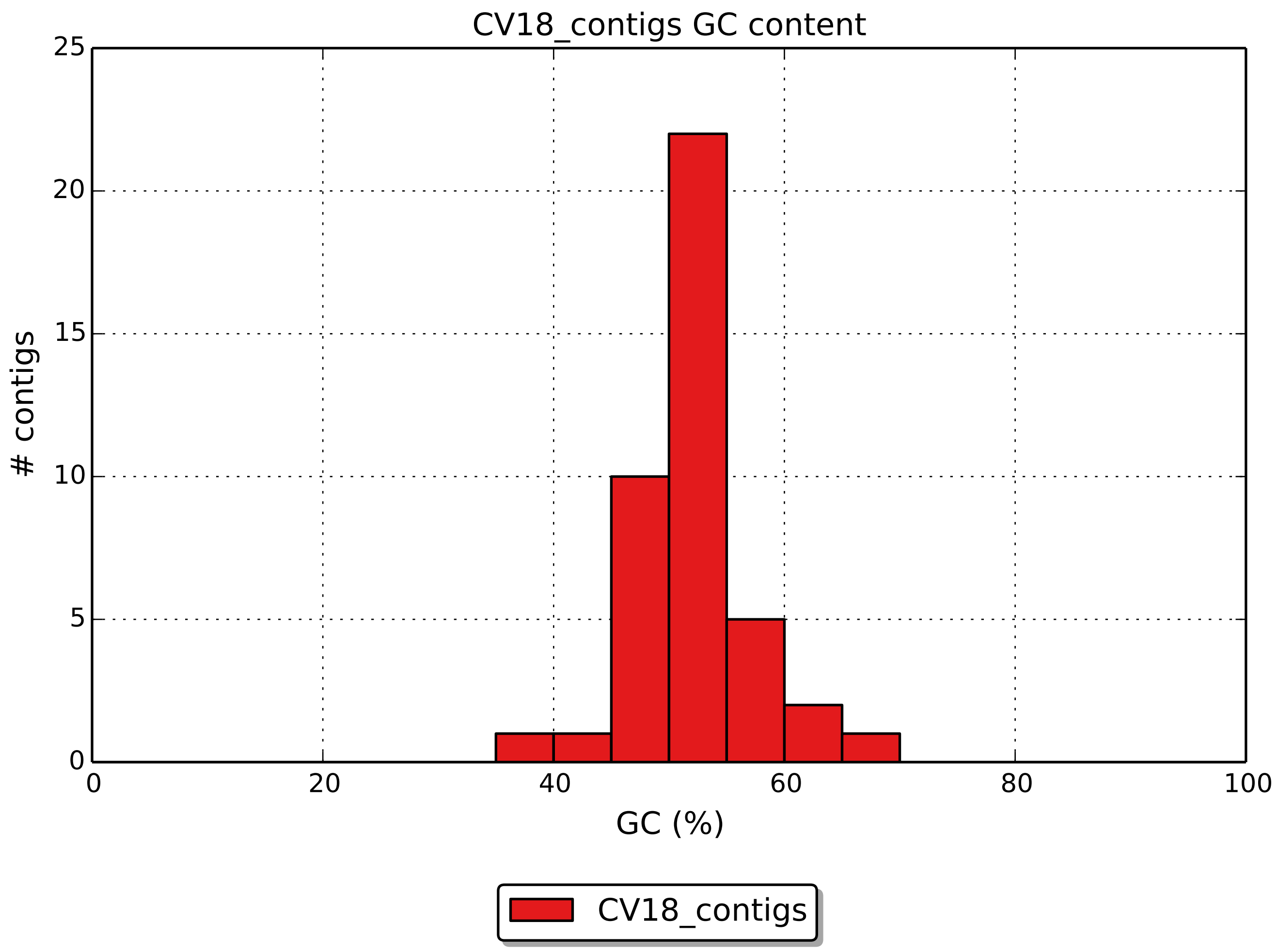

CV18_contigs GC content
contigs
GC (%)
0
5
10
15
20
25
0
20
40
60
80
100
CV18_contigs

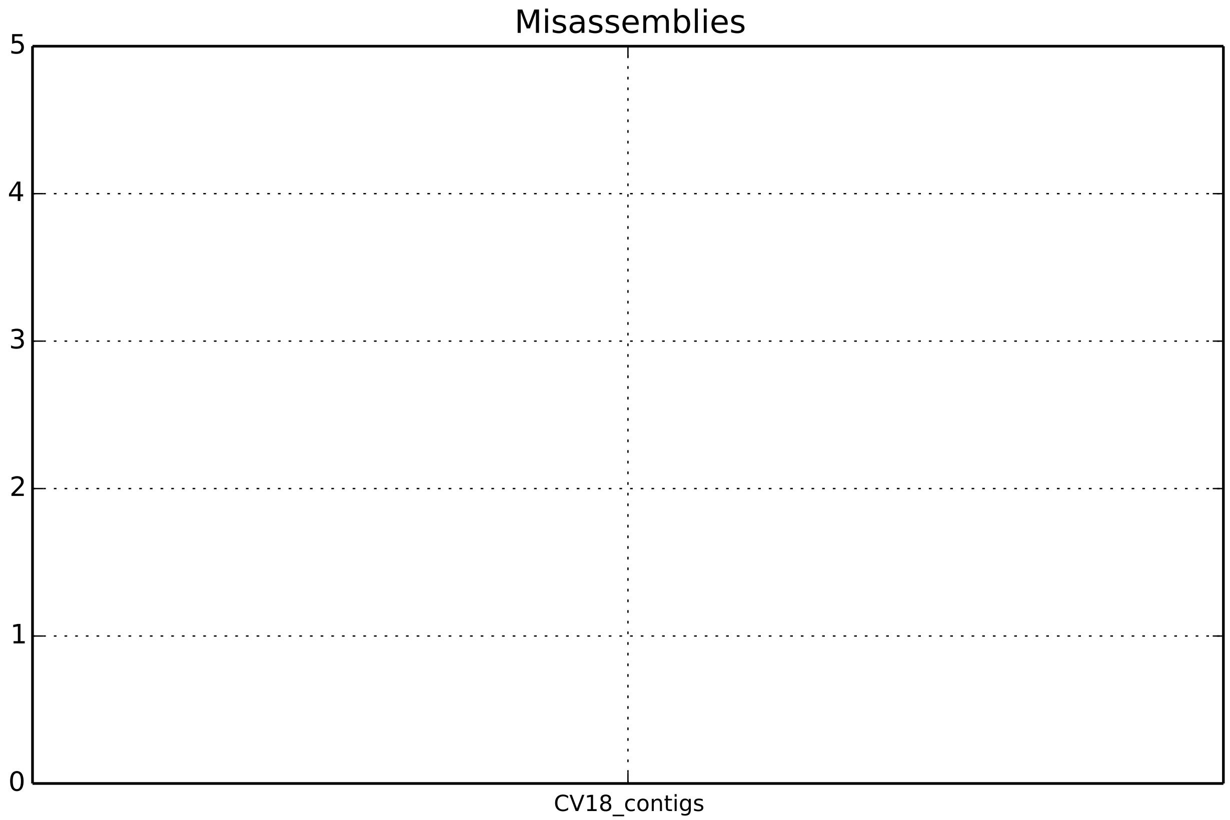
Misassemblies
5
4
3
2
1
0
CV18_contigs

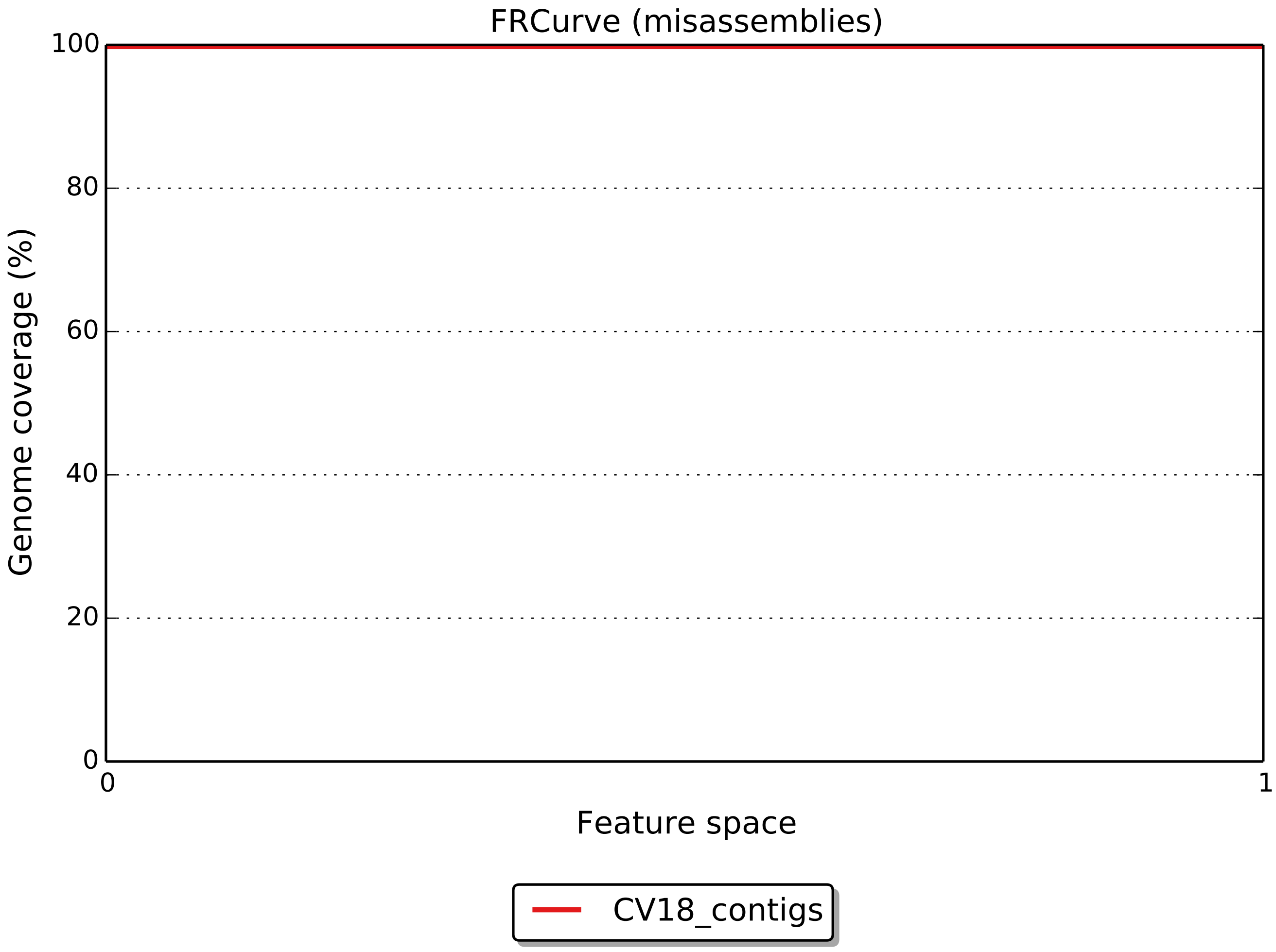
FRCurve (misassemblies)
100
80
60
40
20
0
Genome coverage (%)
0
1
Feature space
CV18_contigs

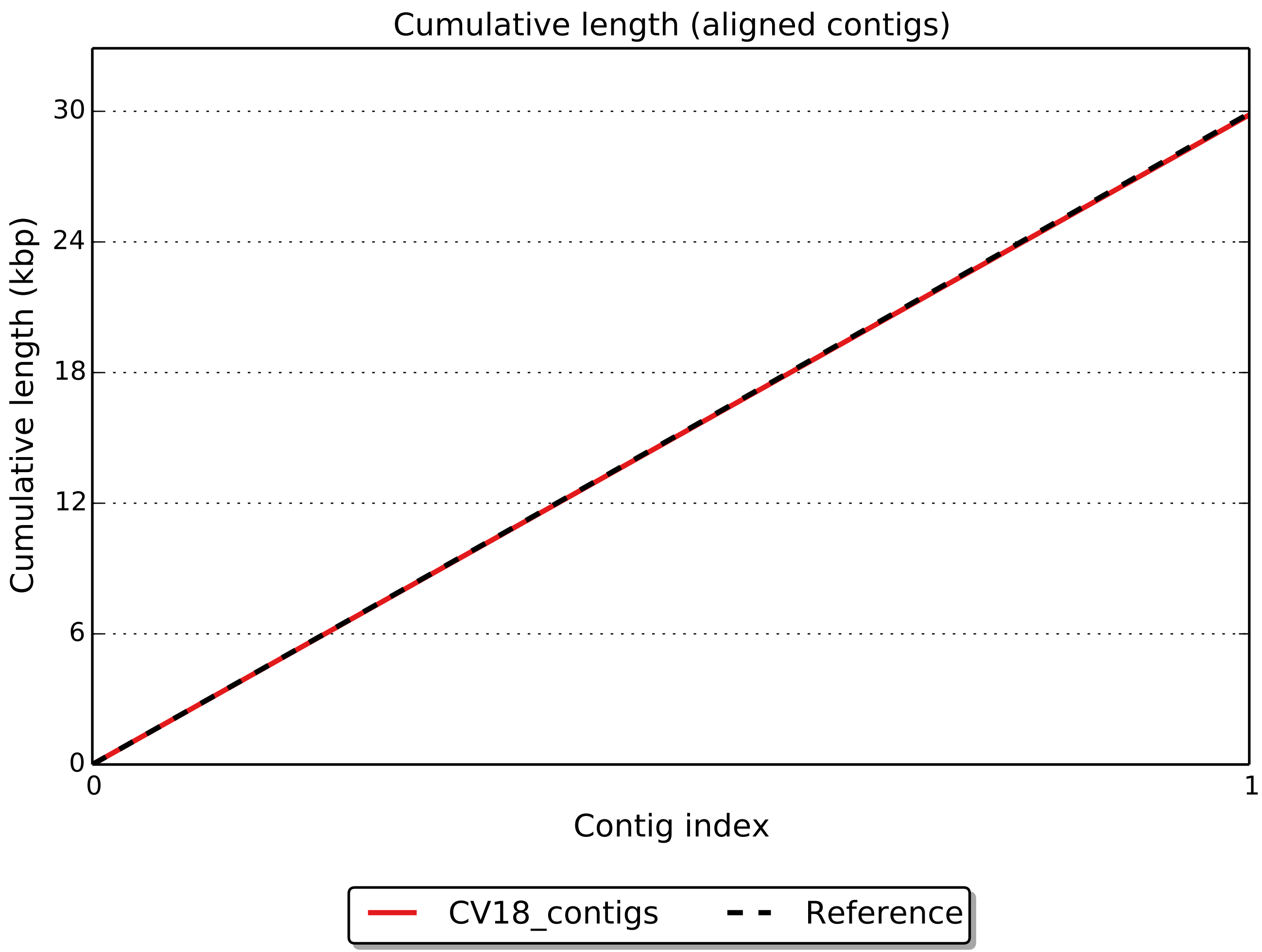

Cumulative length (aligned contigs)
Cumulative length (kbp)
30
24
18
12
6
0
0
1
Contig index
CV18_contigs
Reference

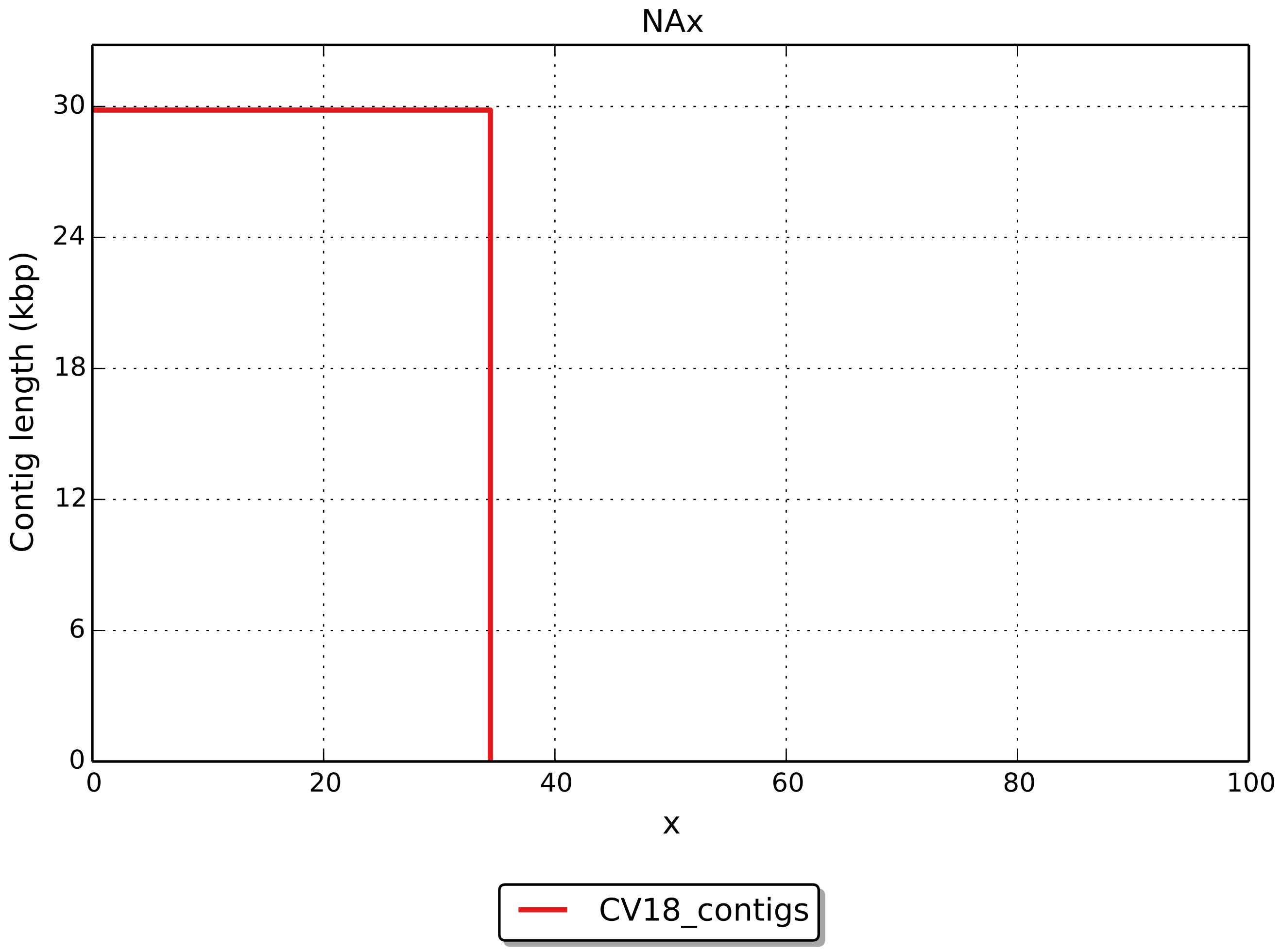

NAx
Contig length (kbp)
30
24
18
12
6
0
0
20
40
60
80
100
x
CV18_contigs

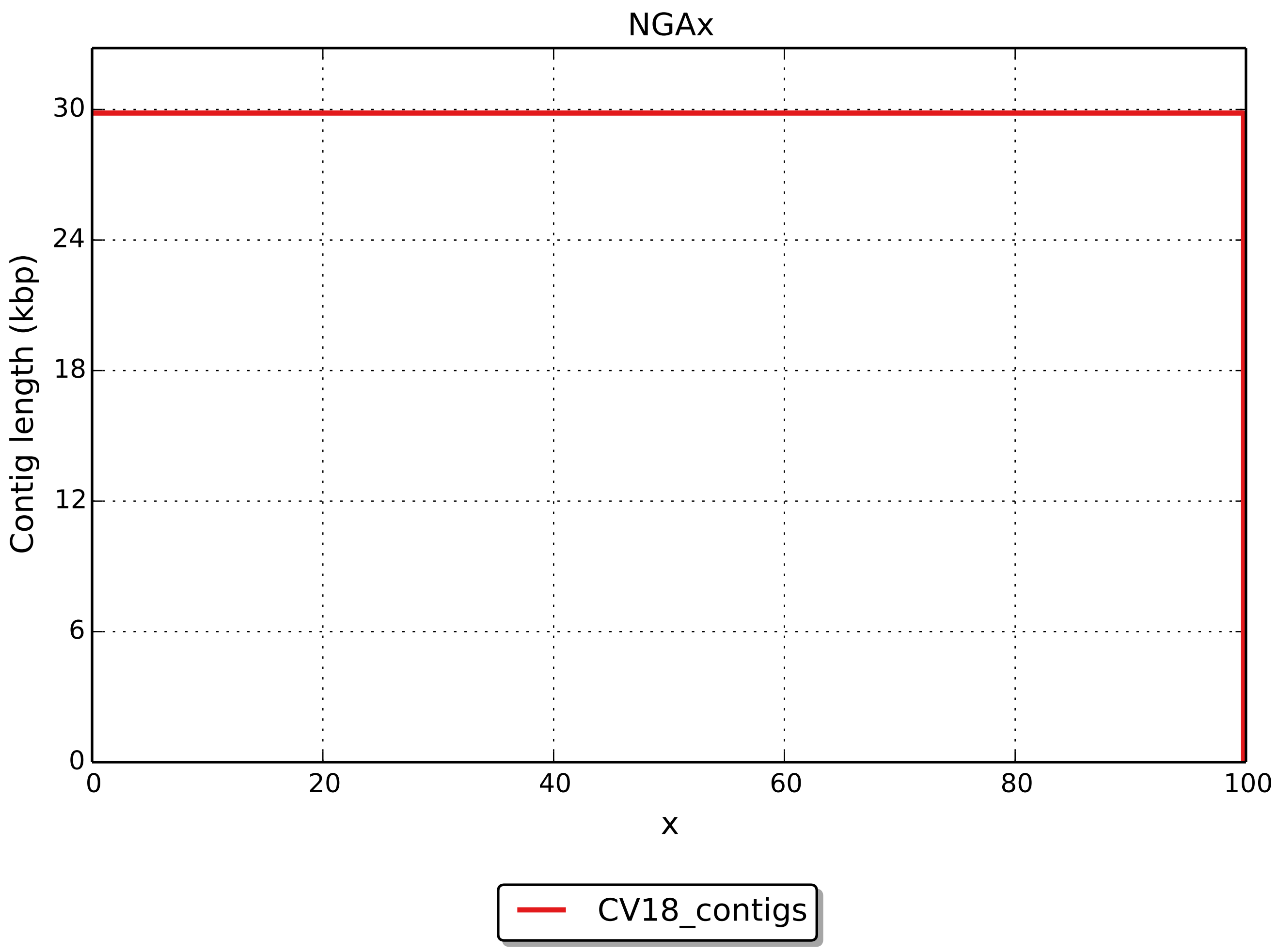
NGAx
Contig length (kbp)
30
24
18
12
6
0
0
20
40
60
80
100
x
CV18_contigs

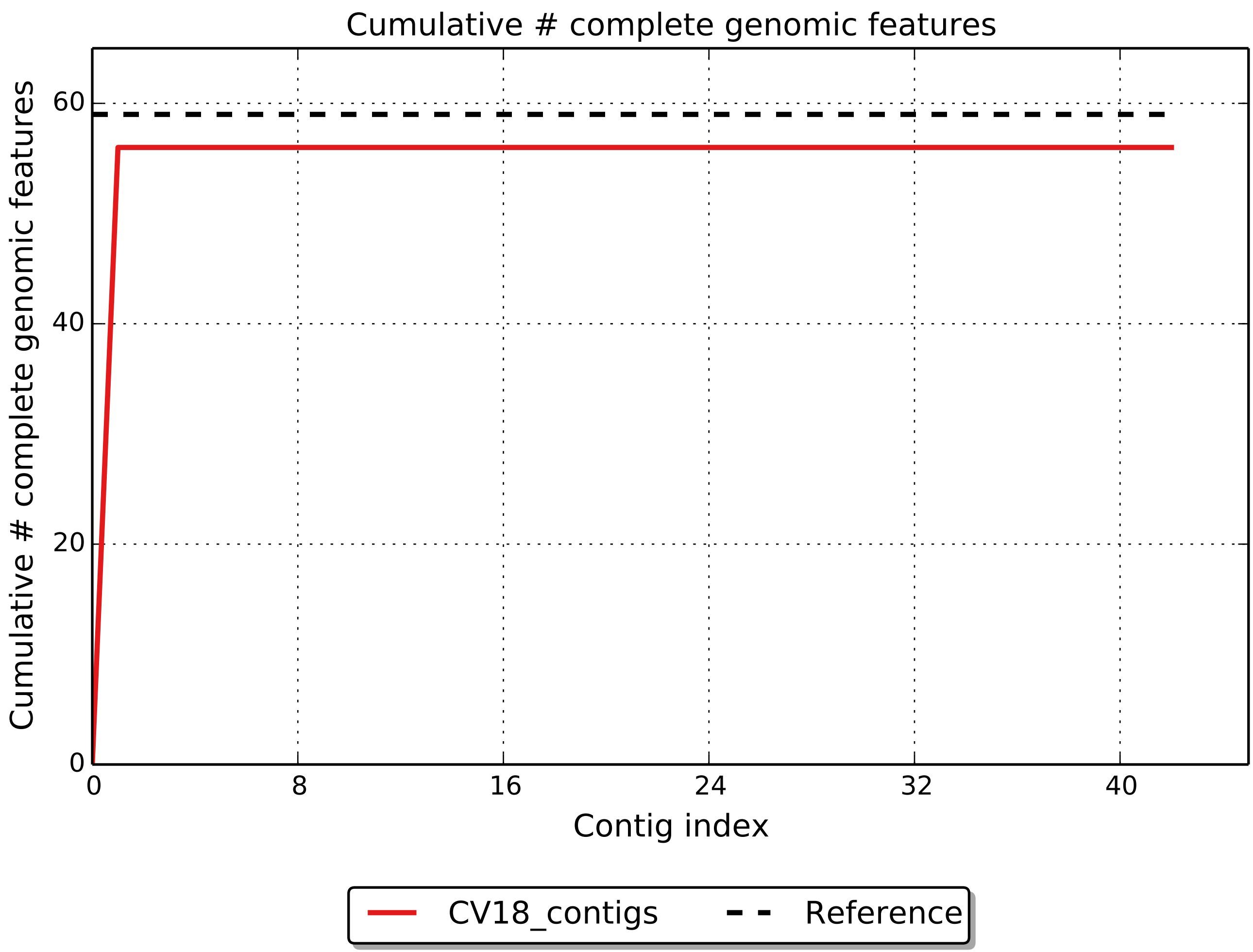
Cumulative # complete genomic features
Cumulative # complete genomic features
0
20
40
60
0
8
16
24
32
40
Contig index
CV18_contigs
Reference

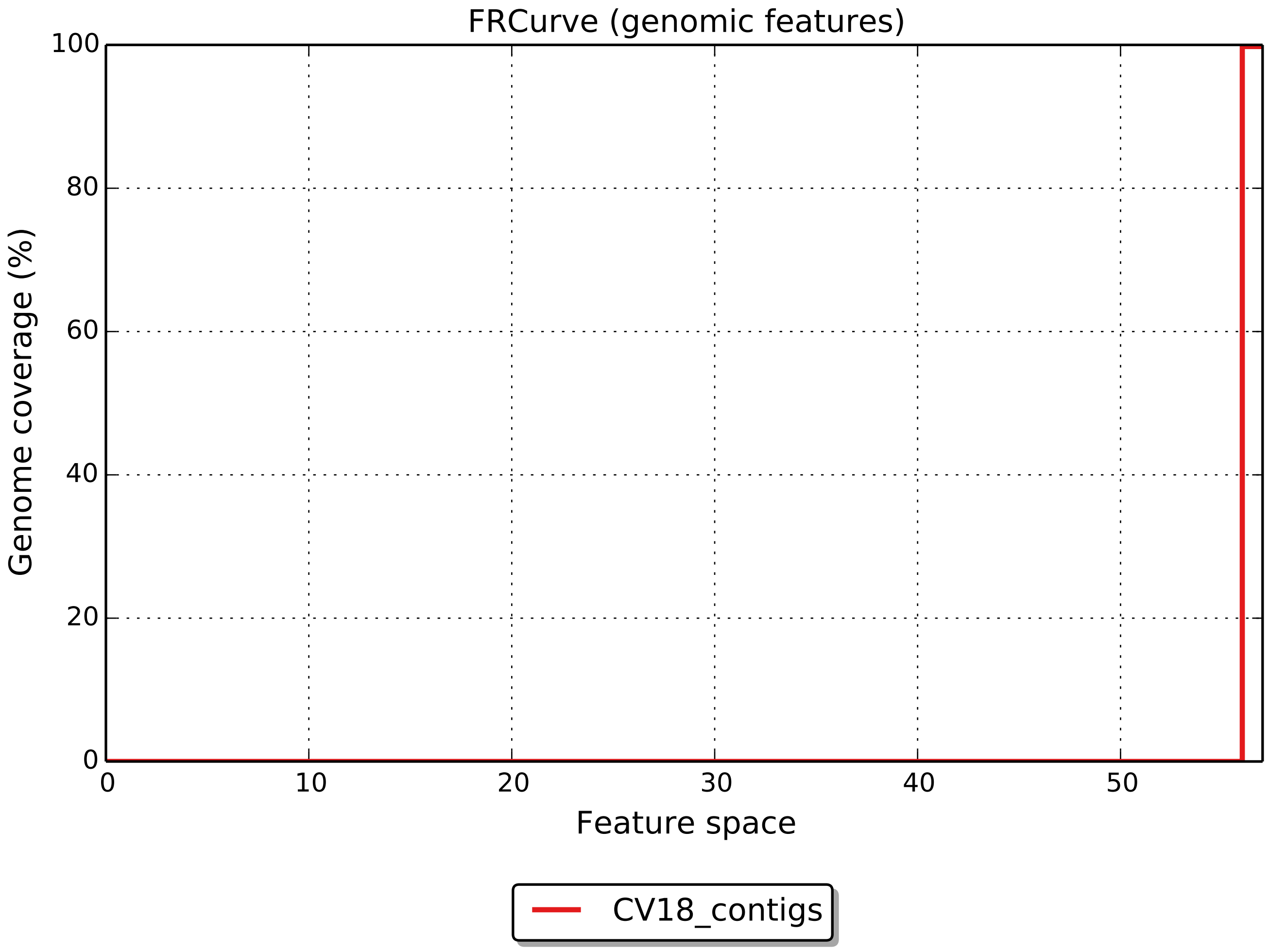
FRCurve (genomic features)
100
80
60
40
20
0
Genome coverage (%)
0
10
20
30
40
50
Feature space
CV18_contigs
