# Supplementary material for: VGEA: an RNA viral assembly toolkit
Source: PeerJ. 2021 Sep 6;9:e12129. doi: 10.7717/peerj.12129 (PMC8428259; doi:10.7717/peerj.12129)
Supplement: Supplemental Information 2 [file peerj-09-12129-s002.zip › Supplementary_File_2/Performance_Comparison_Assembly_Pipelines_Table_2/CV18/spades/quast_results/results_2021_05_14_01_47_23/report.pdf]

Report

	CV18_spades_contigs
# contigs (>= 0 bp)	1198
# contigs (>= 1000 bp)	190
# contigs (>= 5000 bp)	4
# contigs (>= 10000 bp)	1
# contigs (>= 25000 bp)	0
# contigs (>= 50000 bp)	0
Total length (>= 0 bp)	614425
Total length (>= 1000 bp)	353433
Total length (>= 5000 bp)	44723
Total length (>= 10000 bp)	22141
Total length (>= 25000 bp)	0
Total length (>= 50000 bp)	0
# contigs	384
Largest contig	22141
Total length	493639
Reference length	29903
GC (%)	50.68
Reference GC (%)	37.97
N50	1435
NG50	22141
N75	940
NG75	9089
L50	101
LG50	1
L75	208
LG75	2
# misassemblies	1
# misassembled contigs	1
Misassembled contigs length	9089
# local misassemblies	0
# scaffold gap ext. mis.	0
# scaffold gap loc. mis.	0
# unaligned mis. contigs	0
# unaligned contigs	381 + 0 part
Unaligned length	454716
Genome fraction (%)	99.652
Duplication ratio	1.306
# N's per 100 kbp	0.00
# mismatches per 100 kbp	60.40
# indels per 100 kbp	3.36
# genomic features	54 + 5 part
Largest alignment	22069
Total aligned length	38746
NGA50	22069
NGA75	7689
LGA50	1
LGA75	2

All statistics are based on contigs of size >= 500 bp, unless otherwise noted (e.g., "# contigs (>= 0 bp)" and "Total length (>= 0 bp)" include all contigs).

Misassemblies report

	CV18_spades_contigs
# misassemblies	1
# contig misassemblies	1
# c. relocations	1
# c. translocations	0
# c. inversions	0
# scaffold misassemblies	0
# s. relocations	0
# s. translocations	0
# s. inversions	0
# misassembled contigs	1
Misassembled contigs length	9089
# local misassemblies	0
# scaffold gap ext. mis.	0
# scaffold gap loc. mis.	0
# unaligned mis. contigs	0
# mismatches	18
# indels	1
# indels (<= 5 bp)	0
# indels (> 5 bp)	1
Indels length	41

All statistics are based on contigs of size >= 500 bp, unless otherwise noted (e.g., "# contigs (>= 0 bp)" and "Total length (>= 0 bp)" include all contigs).

Unaligned report

	CV18_spades_contigs
# fully unaligned contigs	381
Fully unaligned length	454716
# partially unaligned contigs	0
Partially unaligned length	0
# N's	0

All statistics are based on contigs of size >= 500 bp, unless otherwise noted (e.g., "# contigs (>= 0 bp)" and "Total length (>= 0 bp)" include all contigs).

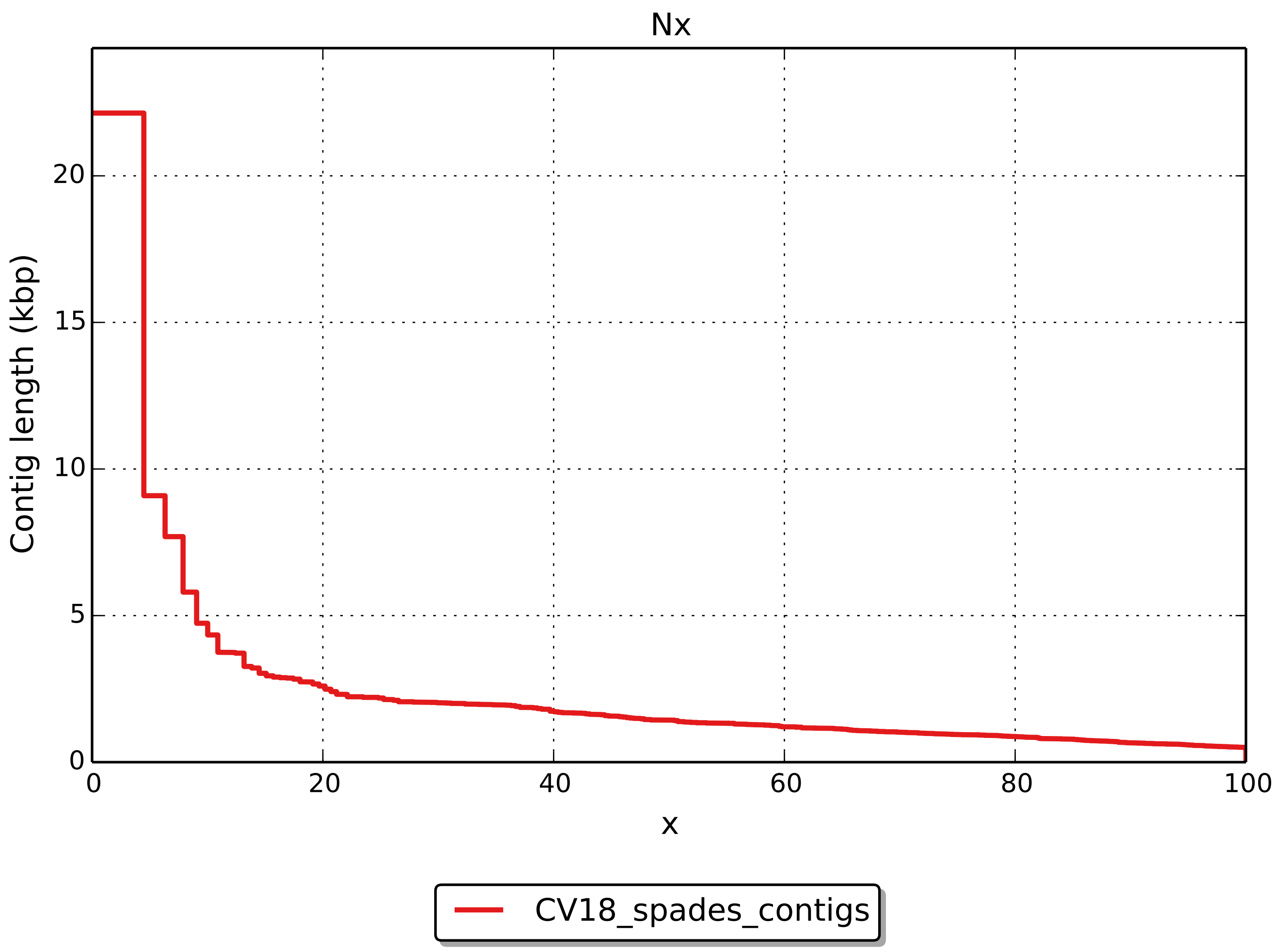

Nx
Contig length (kbp)
0
5
10
15
20
0
20
40
60
80
100
x
CV18_spades_contigs

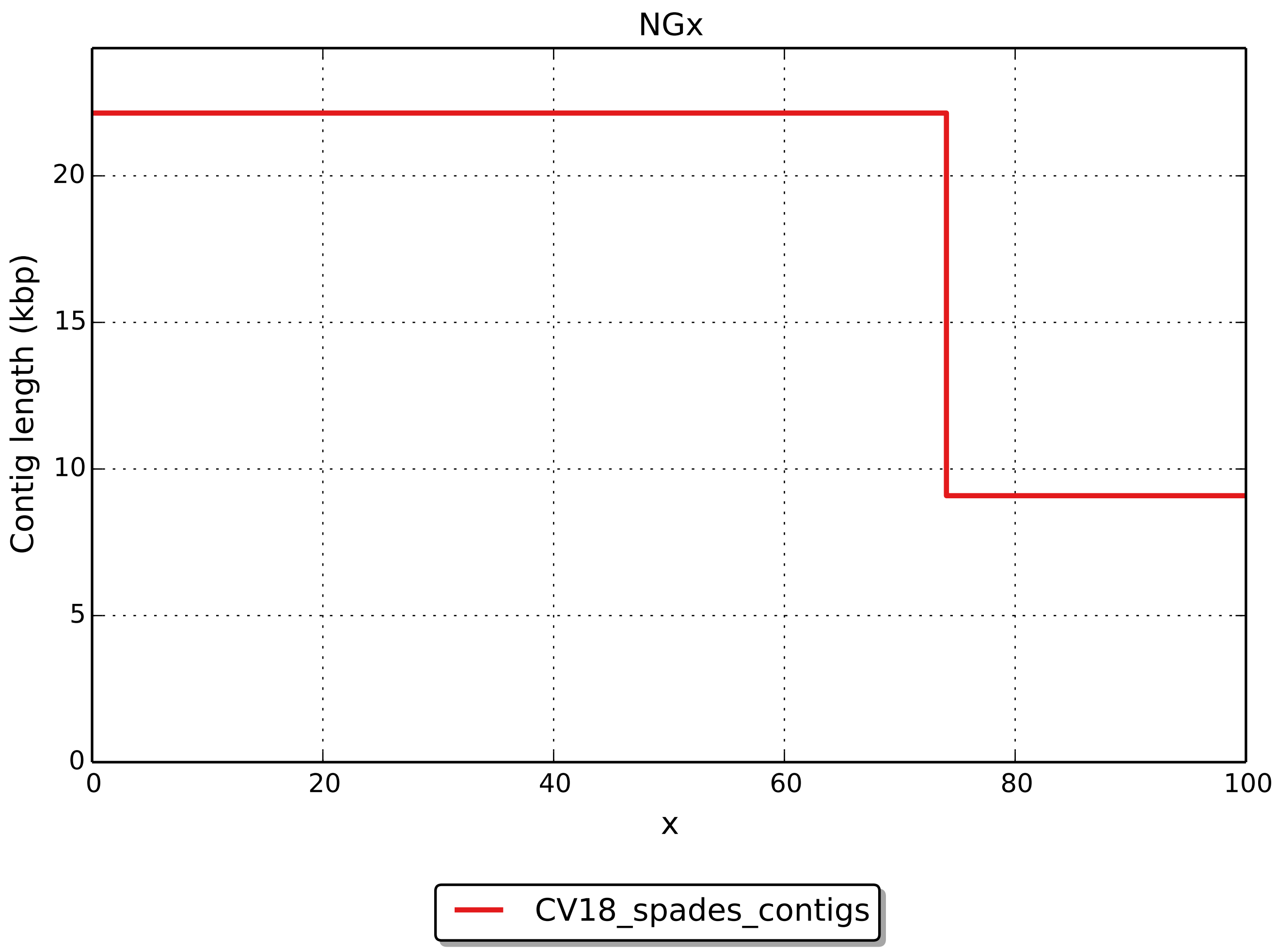
NGx
Contig length (kbp)
0
5
10
15
20
0
20
40
60
80
100
x
CV18_spades_contigs

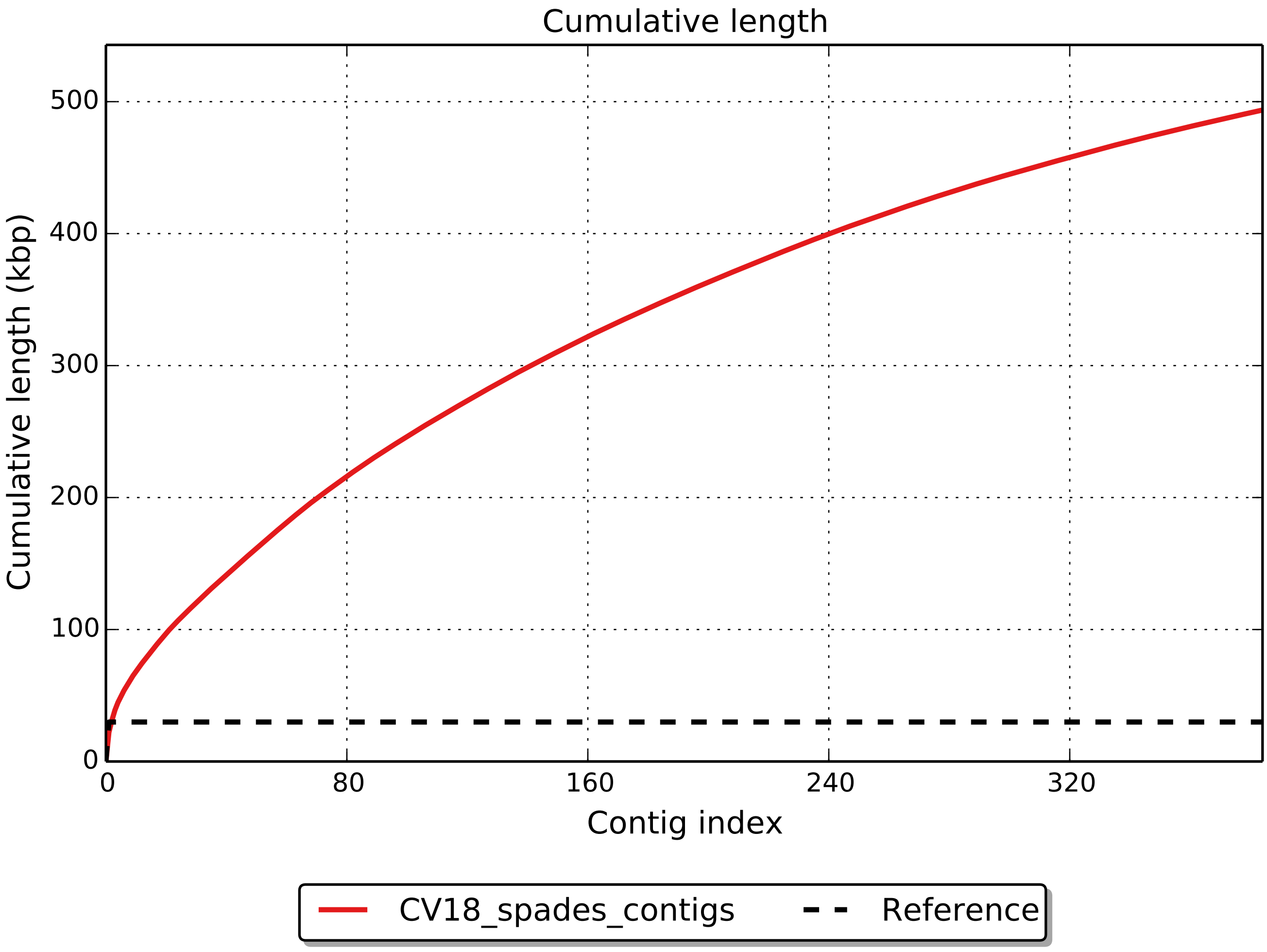
Cumulative length
Cumulative length (kbp)
0
100
200
300
400
500
0
80
160
240
320
Contig index
CV18_spades_contigs
Reference

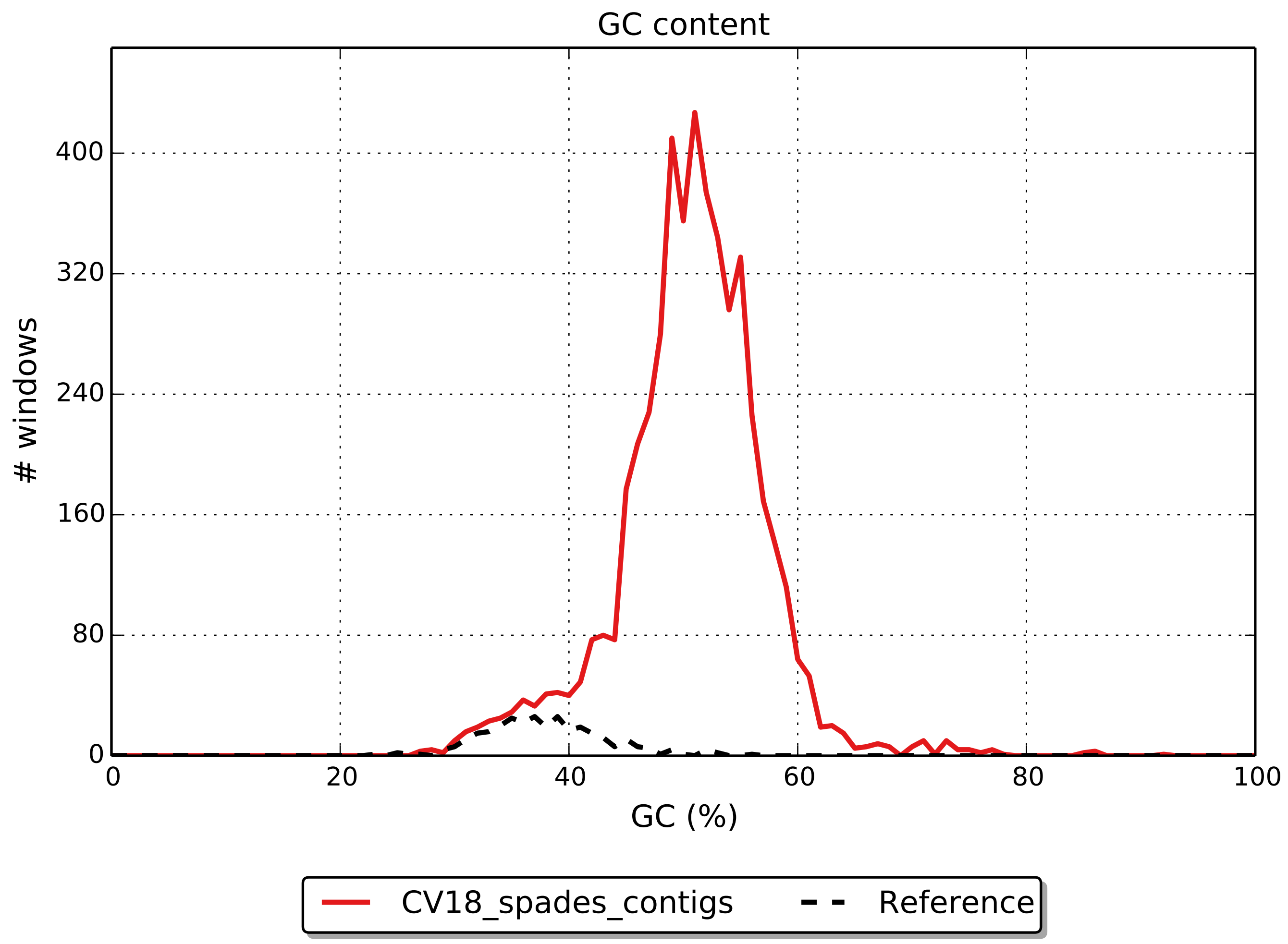

GC content
windows
GC (%)
0
80
160
240
320
400
0
20
40
60
80
100
CV18_spades_contigs
Reference

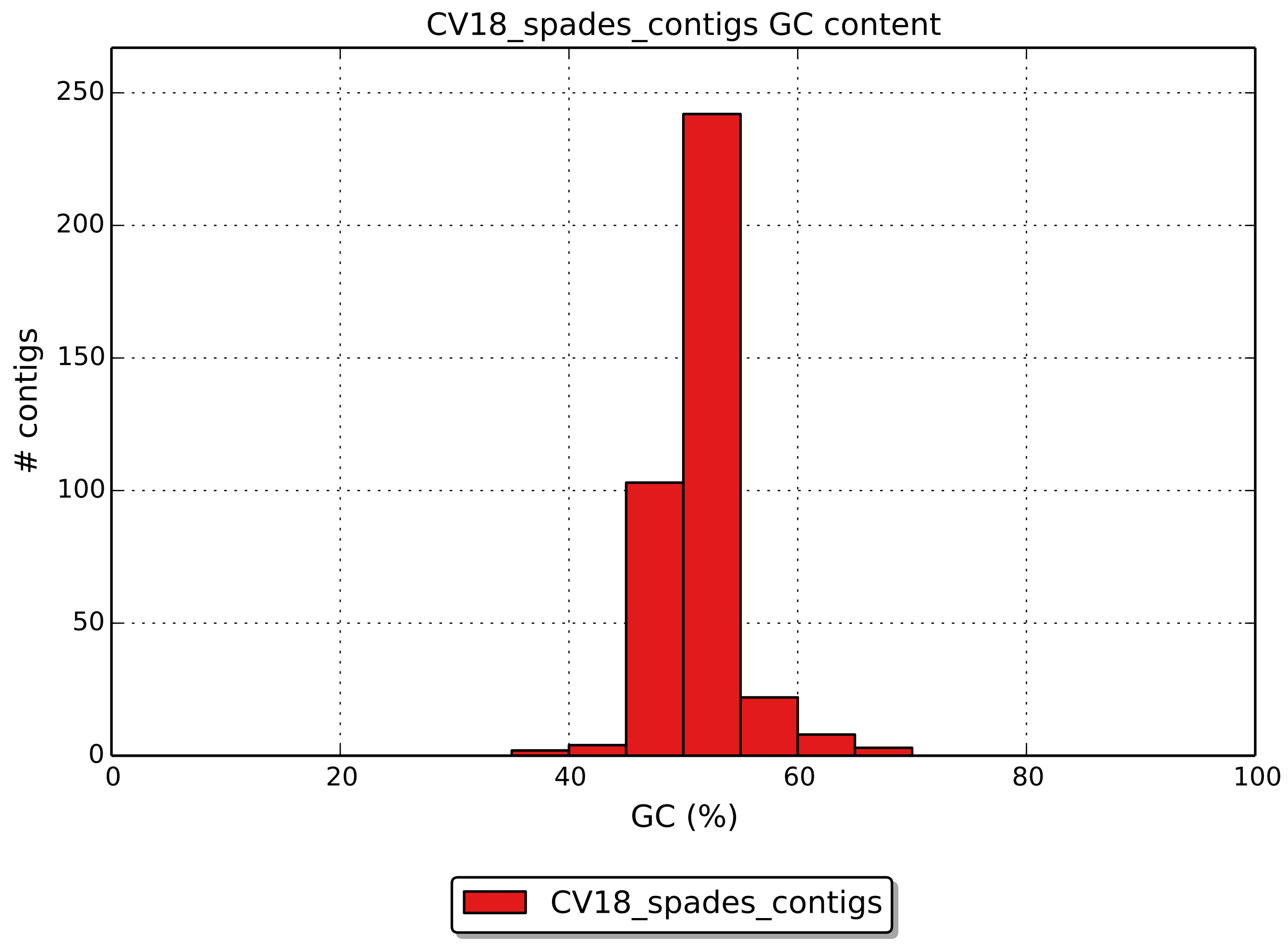
CV18_spades_contigs GC content
contigs
GC (%)
0
50
100
150
200
250
0
20
40
60
80
100
CV18_spades_contigs

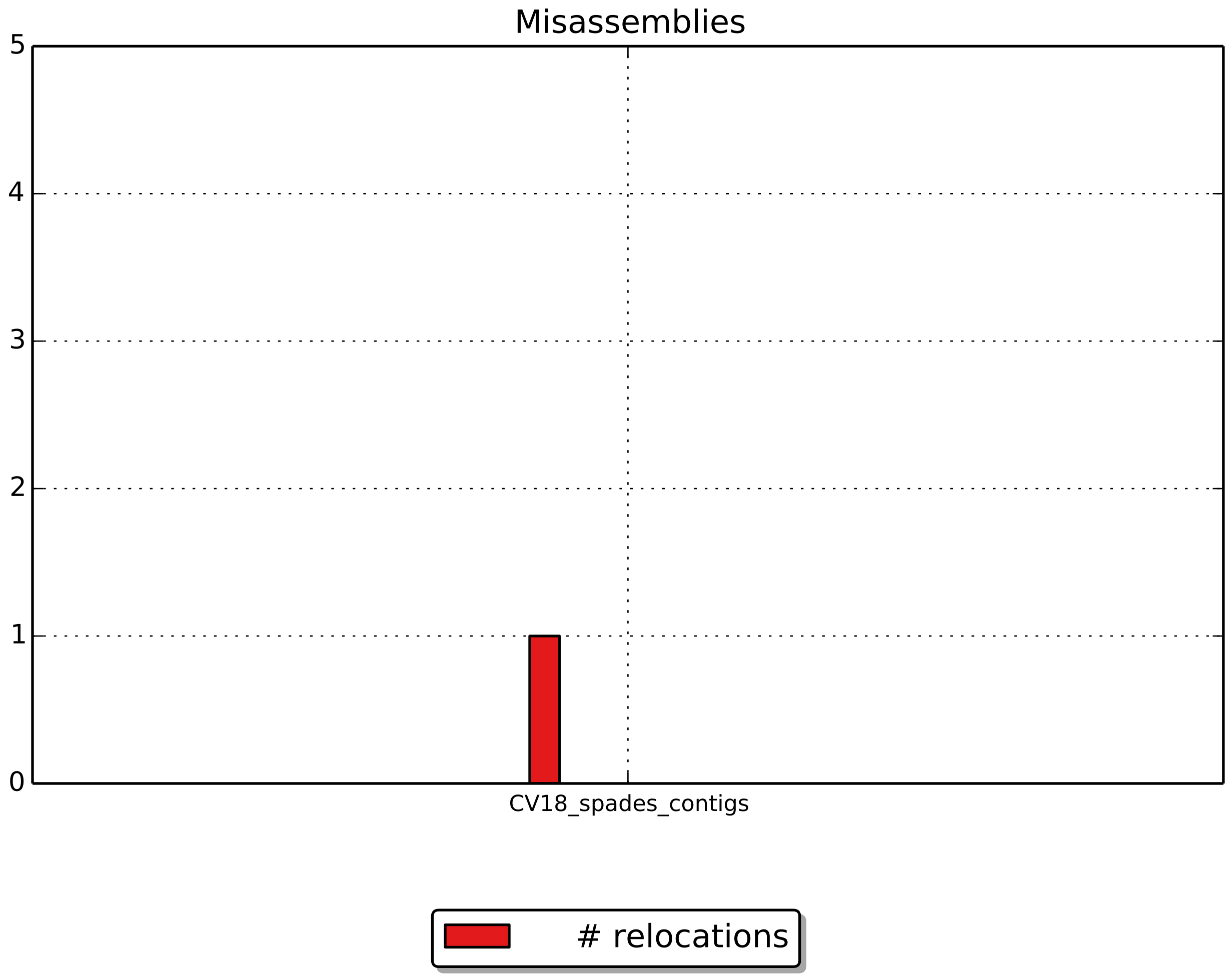
Misassemblies
5
4
3
2
1
0
CV18_spades_contigs
relocations

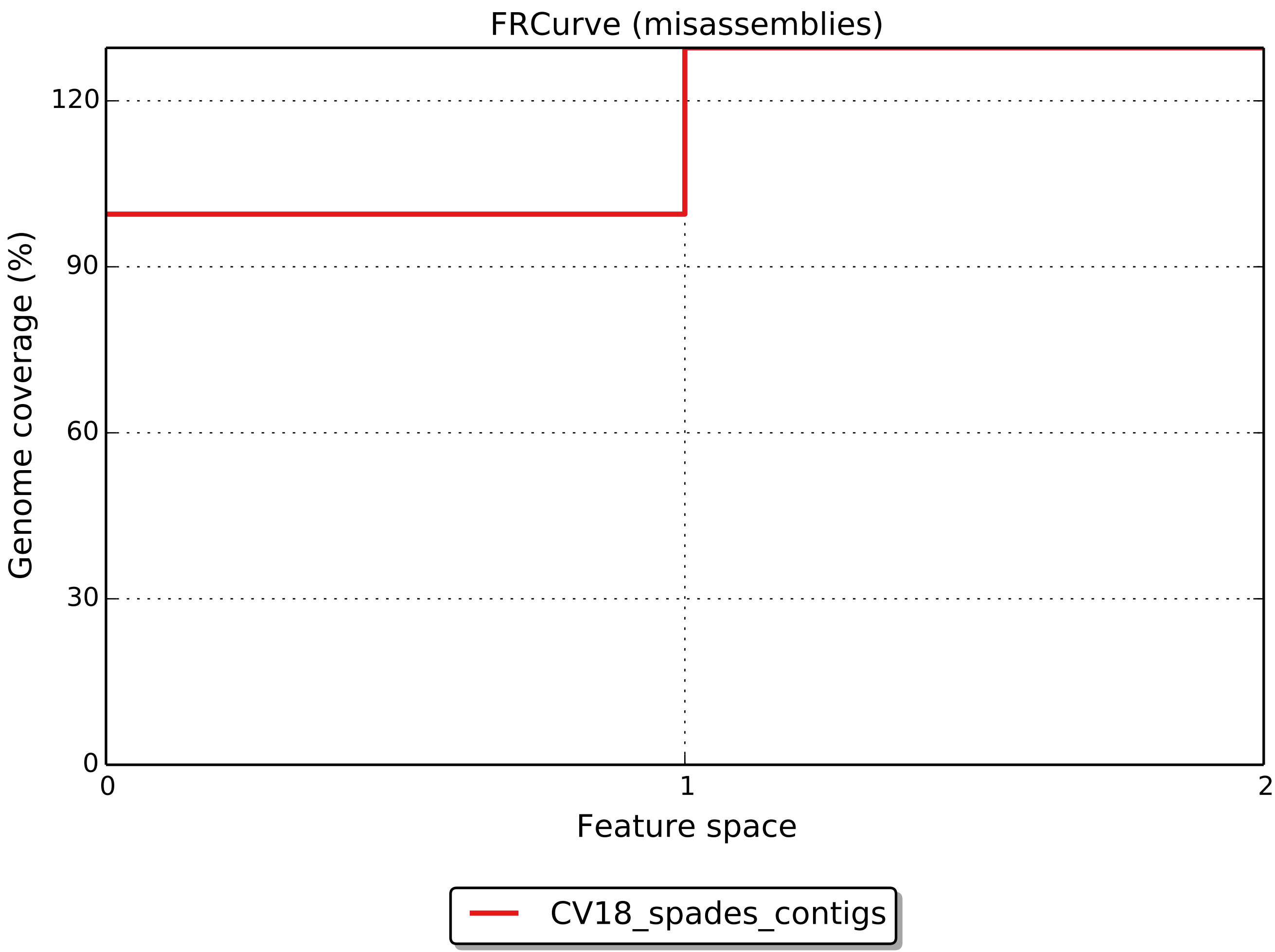

FRCurve (misassemblies)
Genome coverage (%)
120
90
60
30
0
0
1
2
Feature space
CV18_spades_contigs

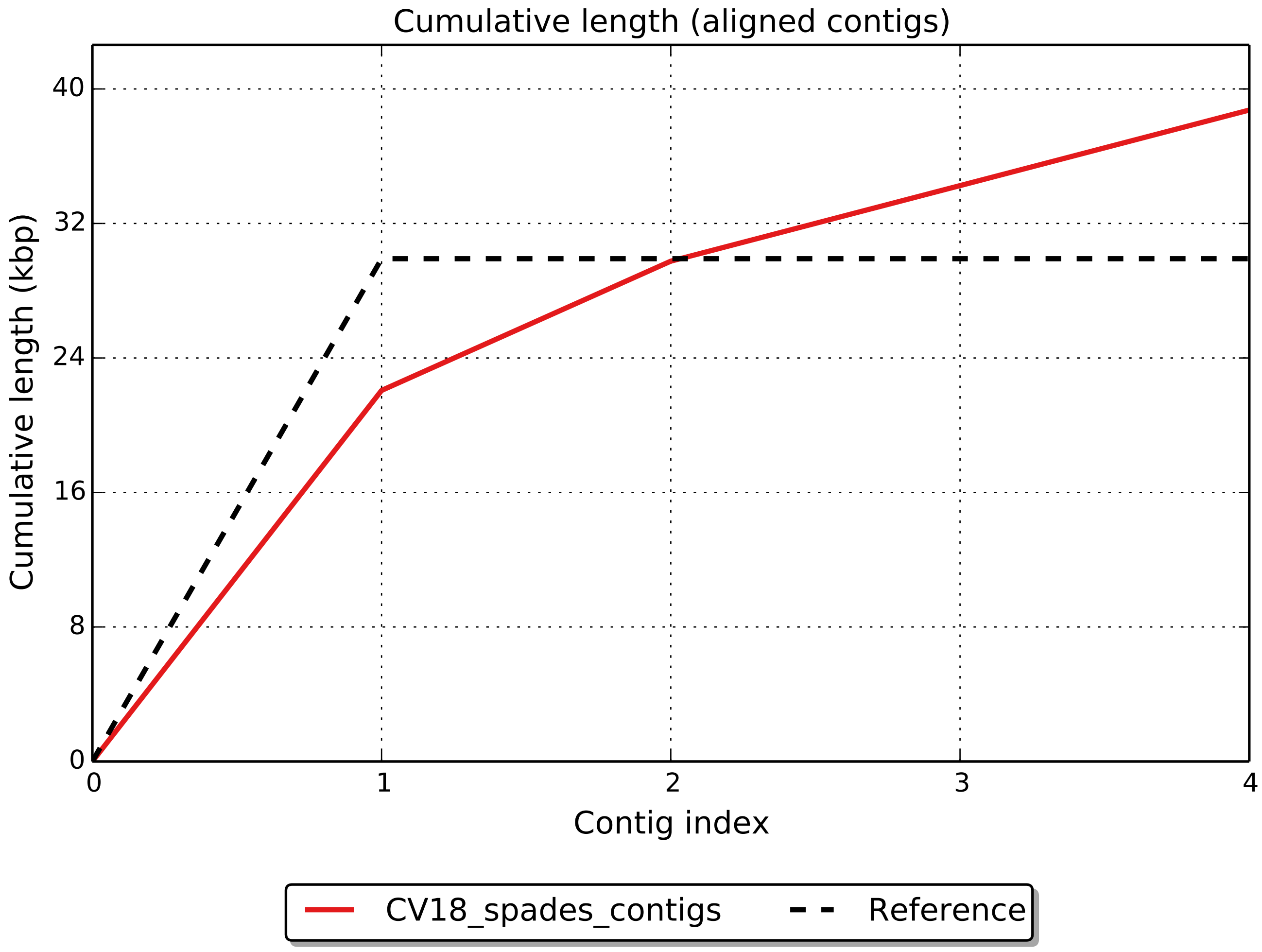

Cumulative length (aligned contigs)
Cumulative length (kbp)
0
8
16
24
32
40
0
1
2
3
4
Contig index
CV18_spades_contigs
Reference

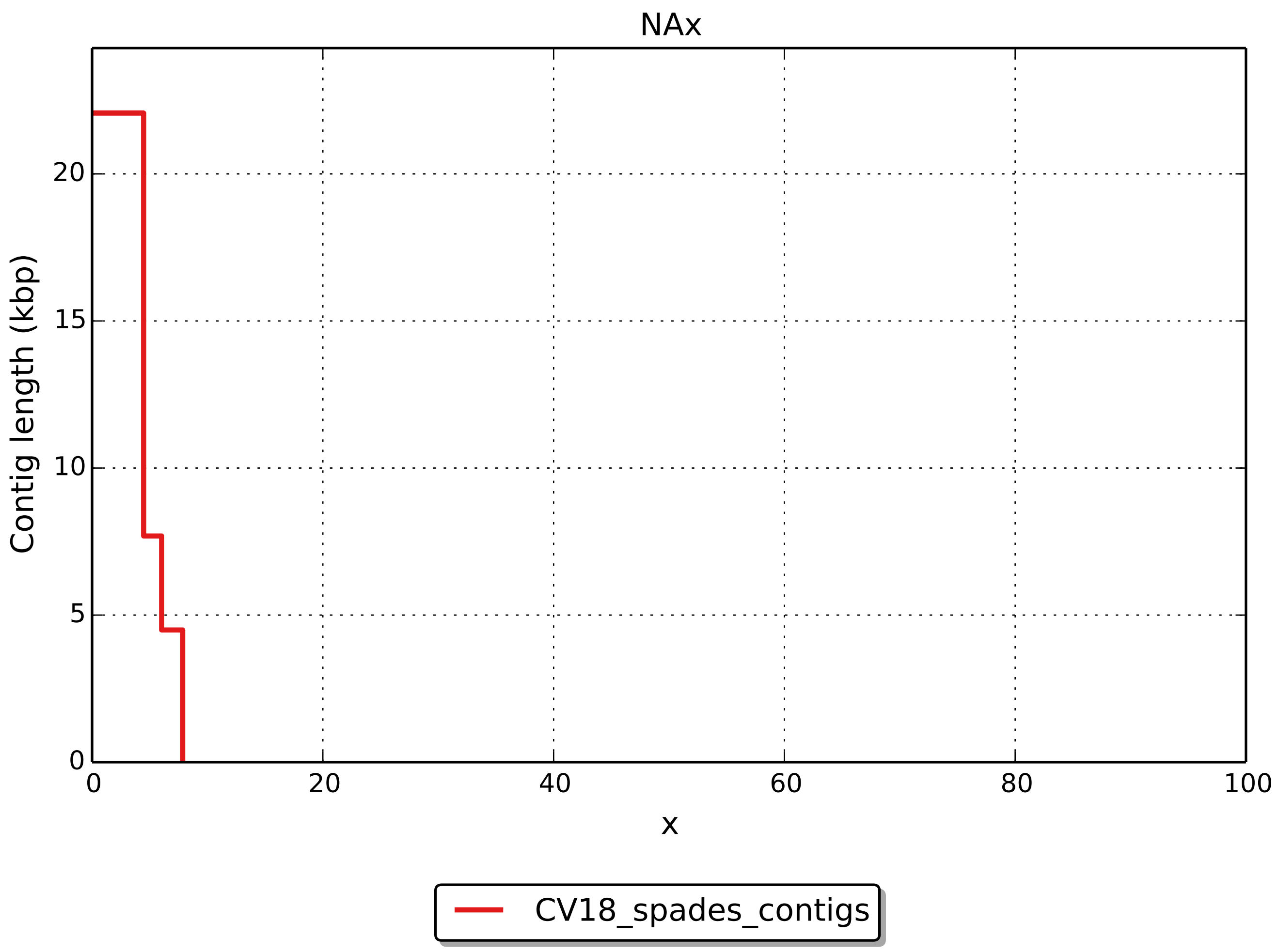
NAx
Contig length (kbp)
0
5
10
15
20
0
20
40
60
80
100
x
CV18_spades_contigs

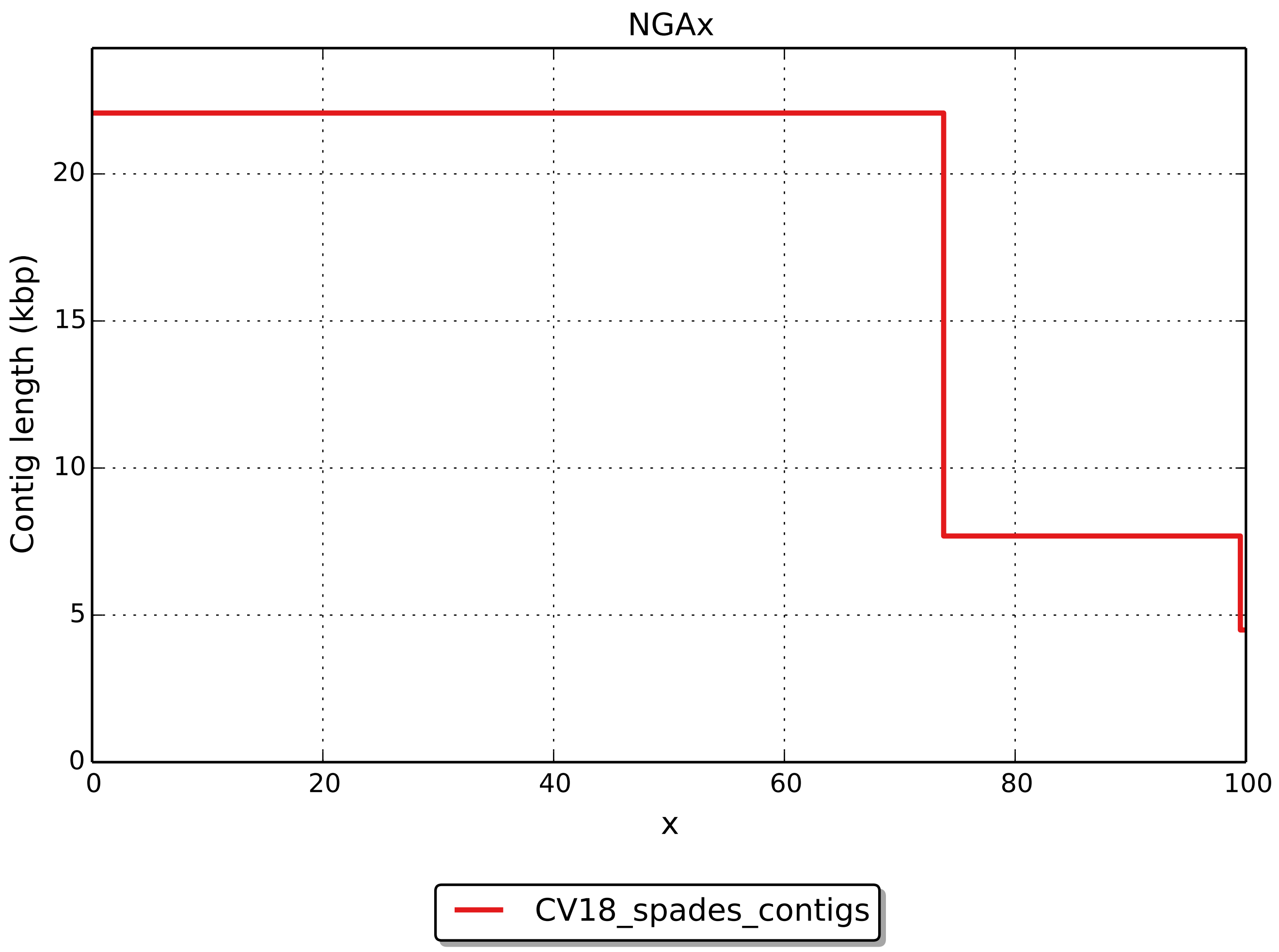
NGAx
Contig length (kbp)
0
5
10
15
20
0
20
40
60
80
100
x
CV18_spades_contigs

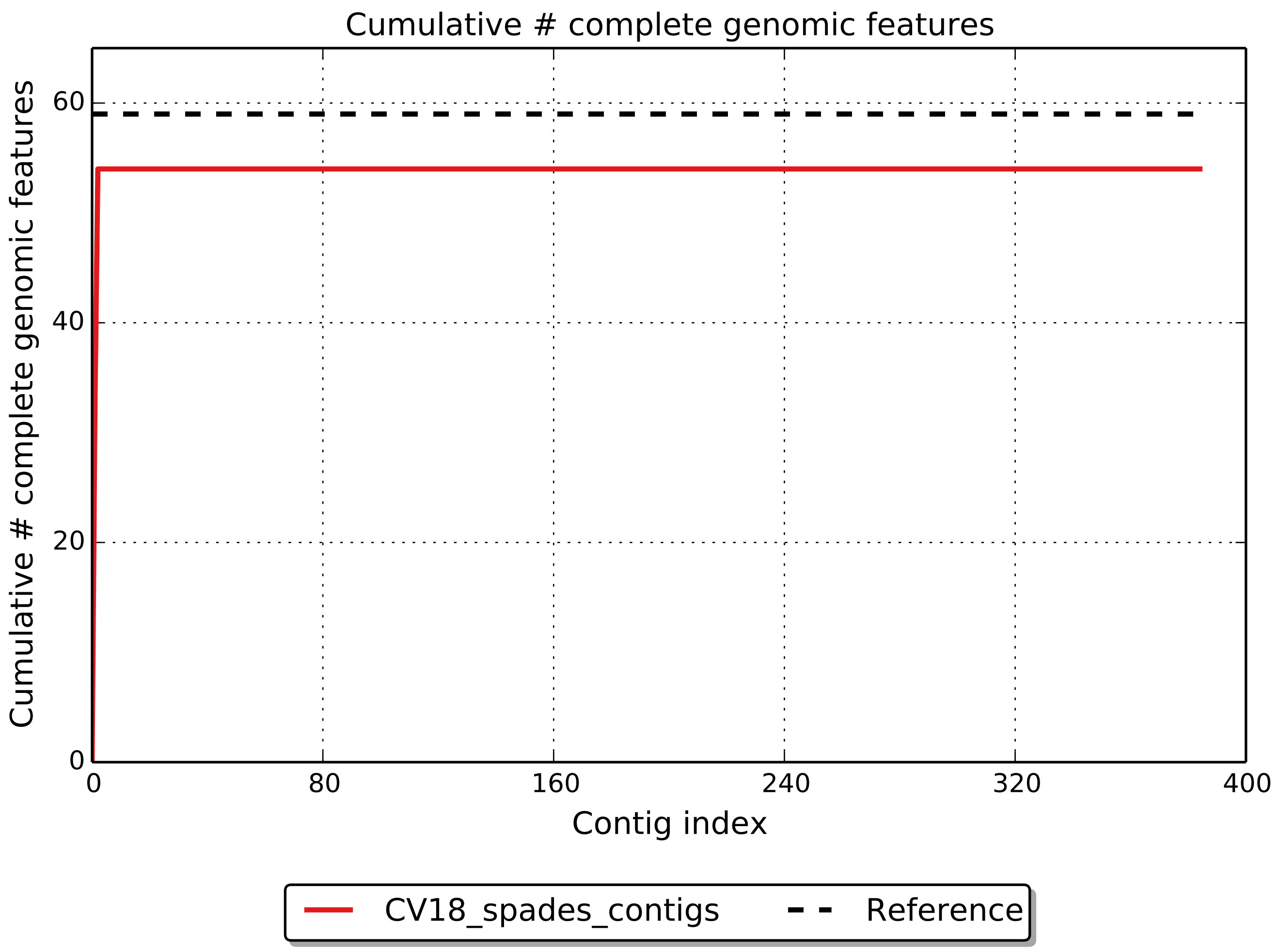

Cumulative # complete genomic features
Cumulative # complete genomic features
Contig index
0
20
40
60
0
80
160
240
320
400
CV18_spades_contigs
Reference

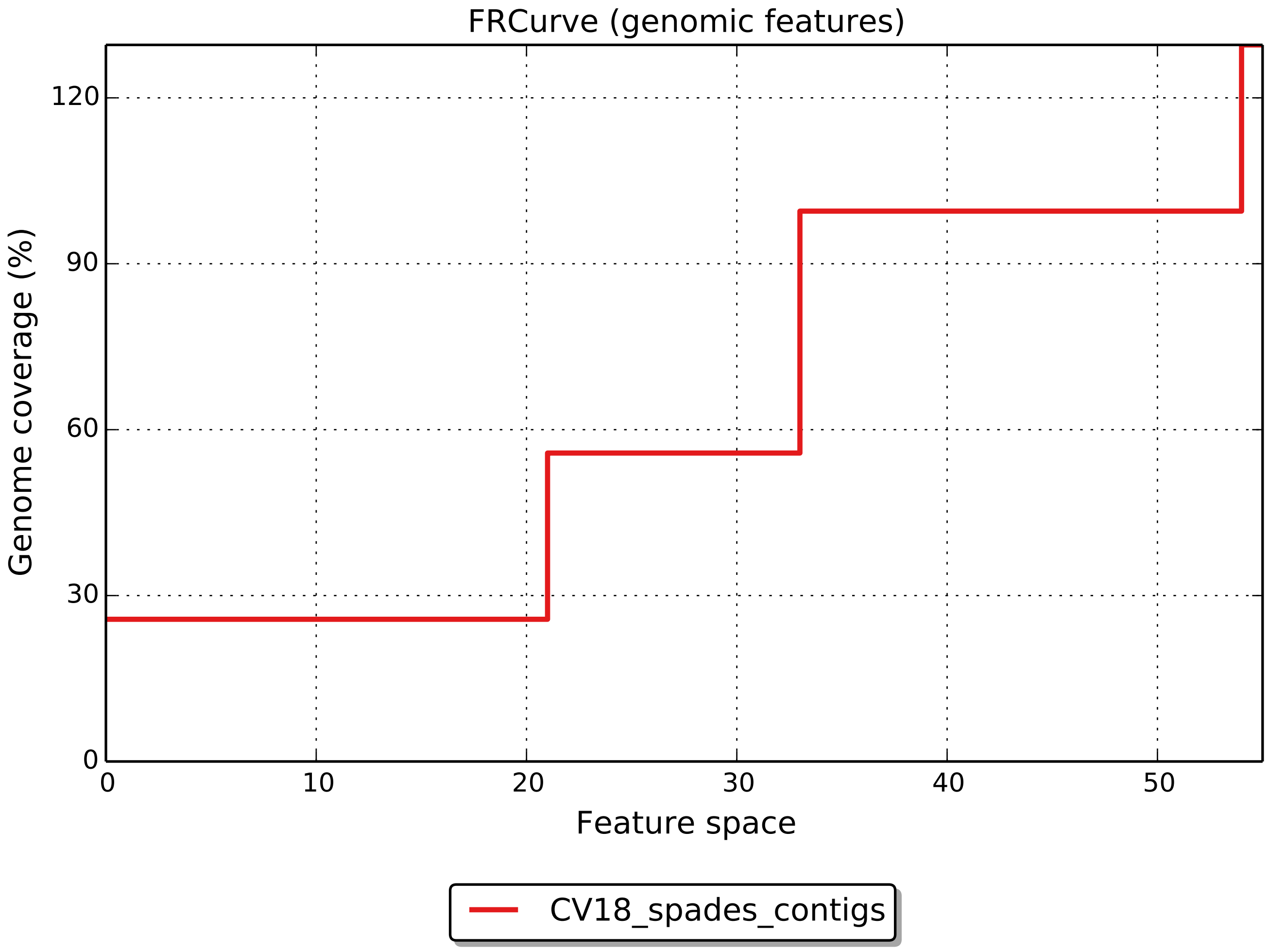
FRCurve (genomic features)
Genome coverage (%)
0
30
60
90
120
0
10
20
30
40
50
Feature space
CV18_spades_contigs
