# Supplementary material for: VGEA: an RNA viral assembly toolkit
Source: PeerJ. 2021 Sep 6;9:e12129. doi: 10.7717/peerj.12129 (PMC8428259; doi:10.7717/peerj.12129)
Supplement: Supplemental Information 2 [file peerj-09-12129-s002.zip › Supplementary_File_2/Performance_Comparison_Assembly_Pipelines_Table_2/CV45/vgea/quast_results/results_2021_05_13_23_04_04/icarus.html]

|  |
| --- |
| Icarus **QUAST Contig Browser** by CAB |

**Assemblies:** CV45\_contigs| Contig size viewer |
| QUAST report |

  

Contig alignment viewer

Aligned to sequences from NC\_045512.2.fasta

Fragments: 1, length: 29 903 bp, mean genome fraction: 98.291%,
misassembled blocks: 2
