# Supplementary material for: VGEA: an RNA viral assembly toolkit
Source: PeerJ. 2021 Sep 6;9:e12129. doi: 10.7717/peerj.12129 (PMC8428259; doi:10.7717/peerj.12129)
Supplement: Supplemental Information 2 [file peerj-09-12129-s002.zip › Supplementary_File_2/Performance_Comparison_Assembly_Pipelines_Table_2/CV45/vgea/quast_results/results_2021_05_13_23_04_04/report.pdf]

Report

	CV45_contigs
# contigs (>= 0 bp)	30
# contigs (>= 1000 bp)	20
# contigs (>= 5000 bp)	1
# contigs (>= 10000 bp)	1
# contigs (>= 25000 bp)	0
# contigs (>= 50000 bp)	0
Total length (>= 0 bp)	61636
Total length (>= 1000 bp)	53606
Total length (>= 5000 bp)	16248
Total length (>= 10000 bp)	16248
Total length (>= 25000 bp)	0
Total length (>= 50000 bp)	0
# contigs	30
Largest contig	16248
Total length	61636
Reference length	29903
GC (%)	44.28
Reference GC (%)	37.97
N50	2603
NG50	16248
N75	1452
NG75	3419
L50	6
LG50	1
L75	14
LG75	3
# misassemblies	1
# misassembled contigs	1
Misassembled contigs length	1135
# local misassemblies	0
# scaffold gap ext. mis.	0
# scaffold gap loc. mis.	0
# unaligned mis. contigs	0
# unaligned contigs	24 + 0 part
Unaligned length	31092
Genome fraction (%)	98.291
Duplication ratio	1.039
# N's per 100 kbp	0.00
# mismatches per 100 kbp	37.43
# indels per 100 kbp	0.00
# genomic features	43 + 16 part
Largest alignment	16110
Total aligned length	30285
NGA50	16110
NGA75	3205
LGA50	1
LGA75	3

All statistics are based on contigs of size >= 500 bp, unless otherwise noted (e.g., "# contigs (>= 0 bp)" and "Total length (>= 0 bp)" include all contigs).

Misassemblies report

	CV45_contigs
# misassemblies	1
# contig misassemblies	1
# c. relocations	0
# c. translocations	0
# c. inversions	1
# scaffold misassemblies	0
# s. relocations	0
# s. translocations	0
# s. inversions	0
# misassembled contigs	1
Misassembled contigs length	1135
# local misassemblies	0
# scaffold gap ext. mis.	0
# scaffold gap loc. mis.	0
# unaligned mis. contigs	0
# mismatches	11
# indels	0
# indels (<= 5 bp)	0
# indels (> 5 bp)	0
Indels length	0

All statistics are based on contigs of size >= 500 bp, unless otherwise noted (e.g., "# contigs (>= 0 bp)" and "Total length (>= 0 bp)" include all contigs).

Unaligned report

	CV45_contigs
# fully unaligned contigs	24
Fully unaligned length	31092
# partially unaligned contigs	0
Partially unaligned length	0
# N's	0

All statistics are based on contigs of size >= 500 bp, unless otherwise noted (e.g., "# contigs (>= 0 bp)" and "Total length (>= 0 bp)" include all contigs).

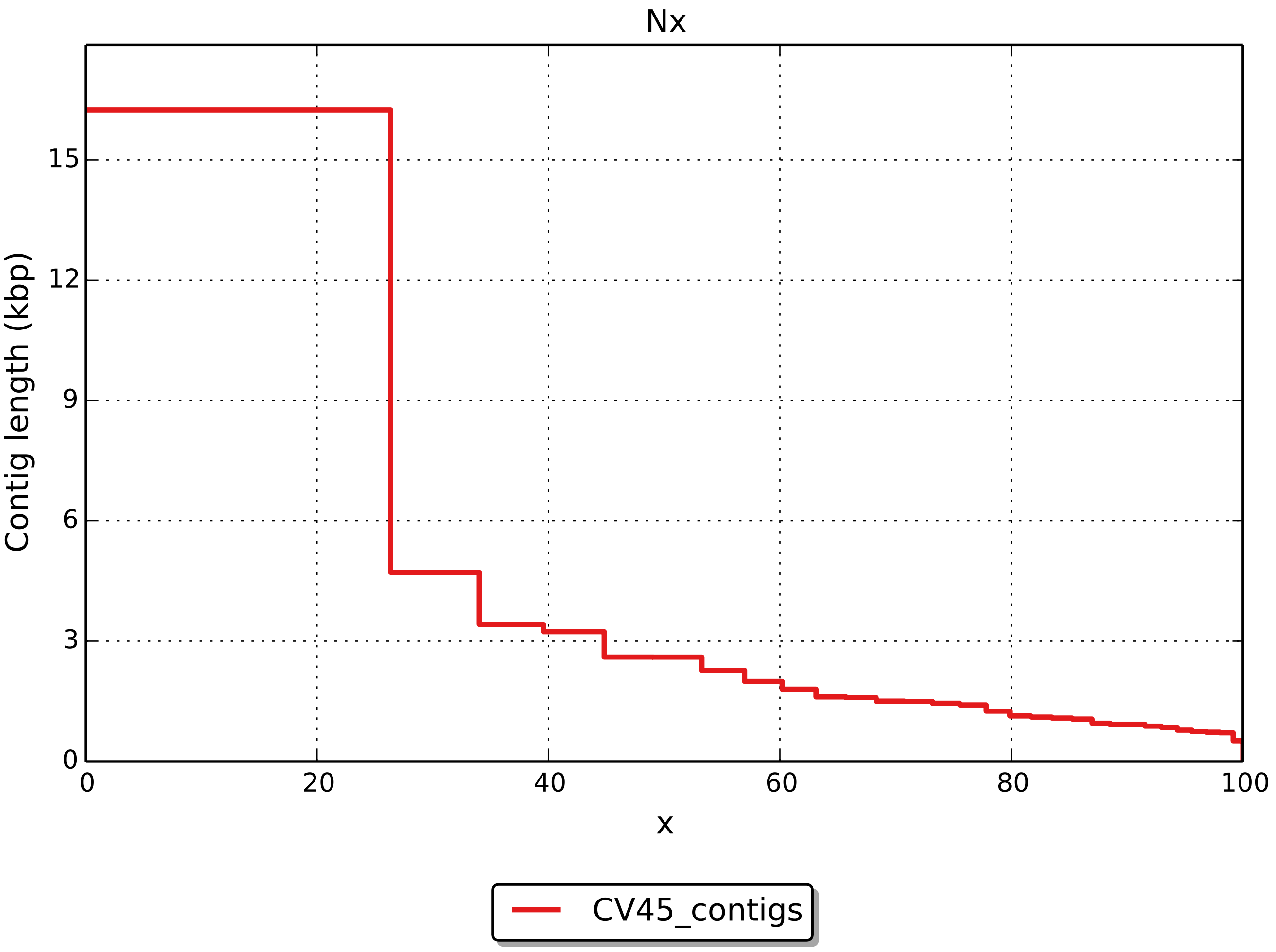

Nx
Contig length (kbp)
0
3
6
9
12
15
0
20
40
60
80
100
x
CV45_contigs

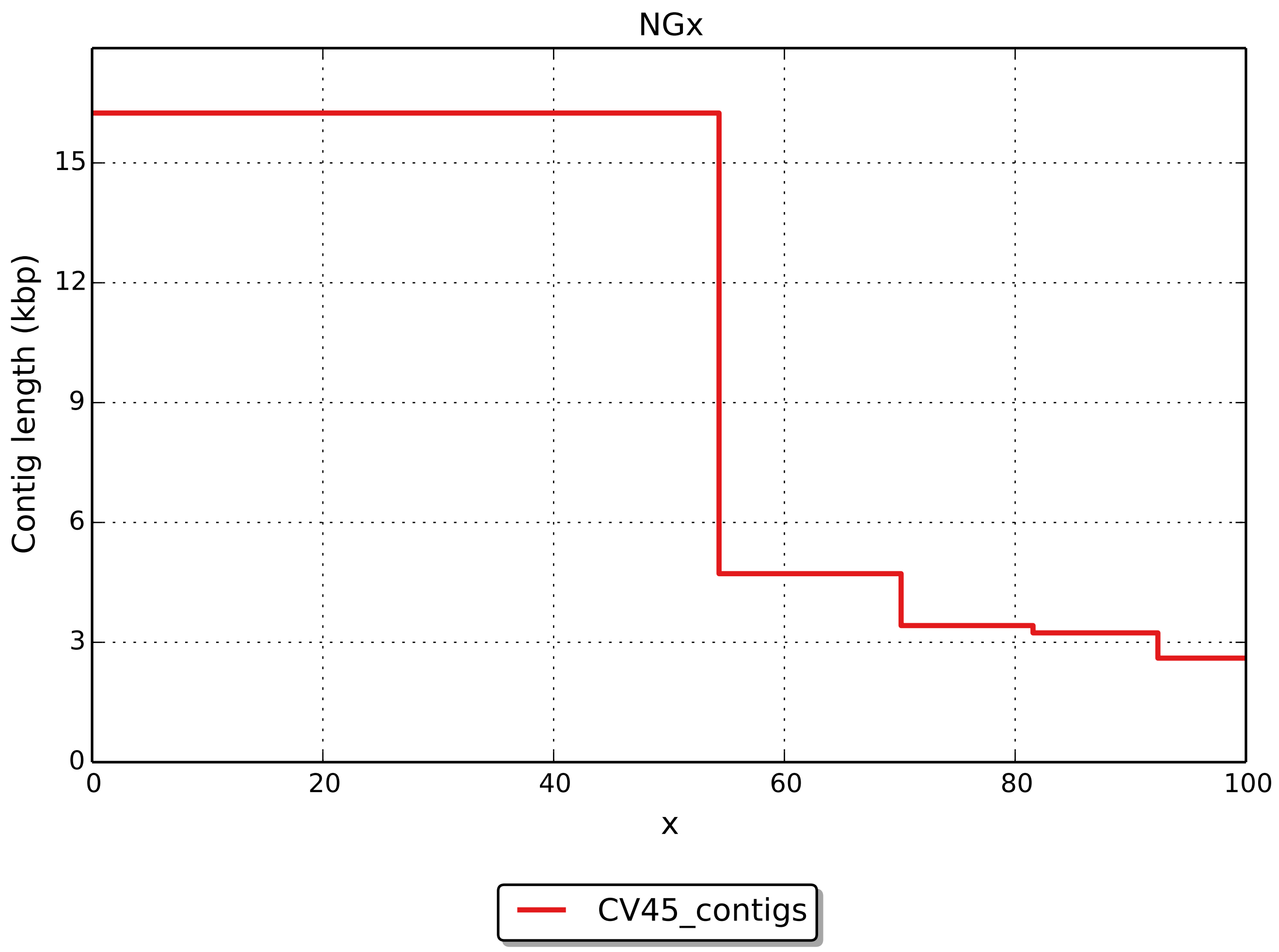
NGx
Contig length (kbp)
0
3
6
9
12
15
0
20
40
60
80
100
x
CV45_contigs

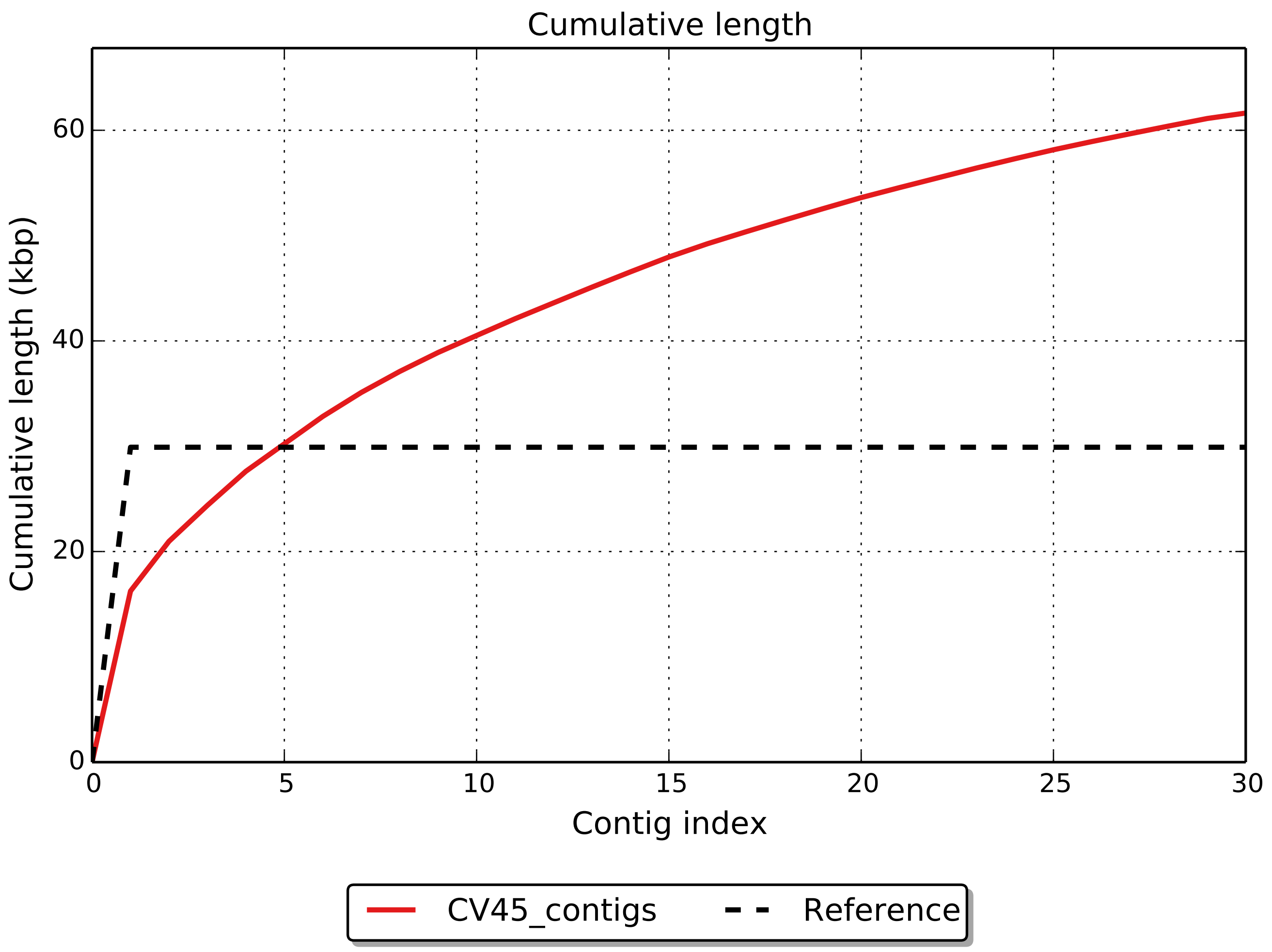

Cumulative length
Cumulative length (kbp)
0
20
40
60
0
5
10
15
20
25
30
Contig index
CV45_contigs
Reference

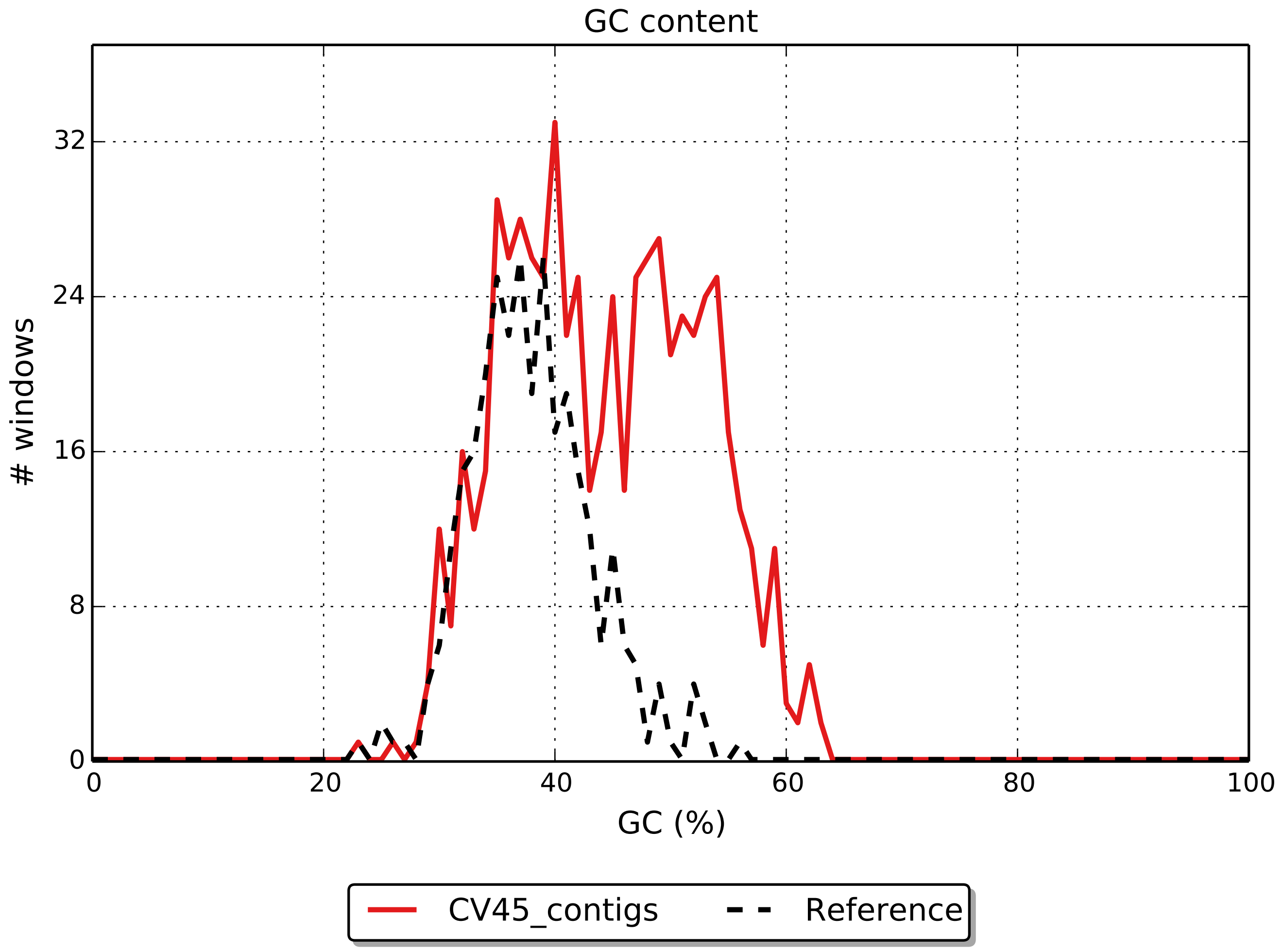

GC content
windows
0
8
16
24
32
0
20
40
60
80
100
GC (%)
CV45_contigs
Reference

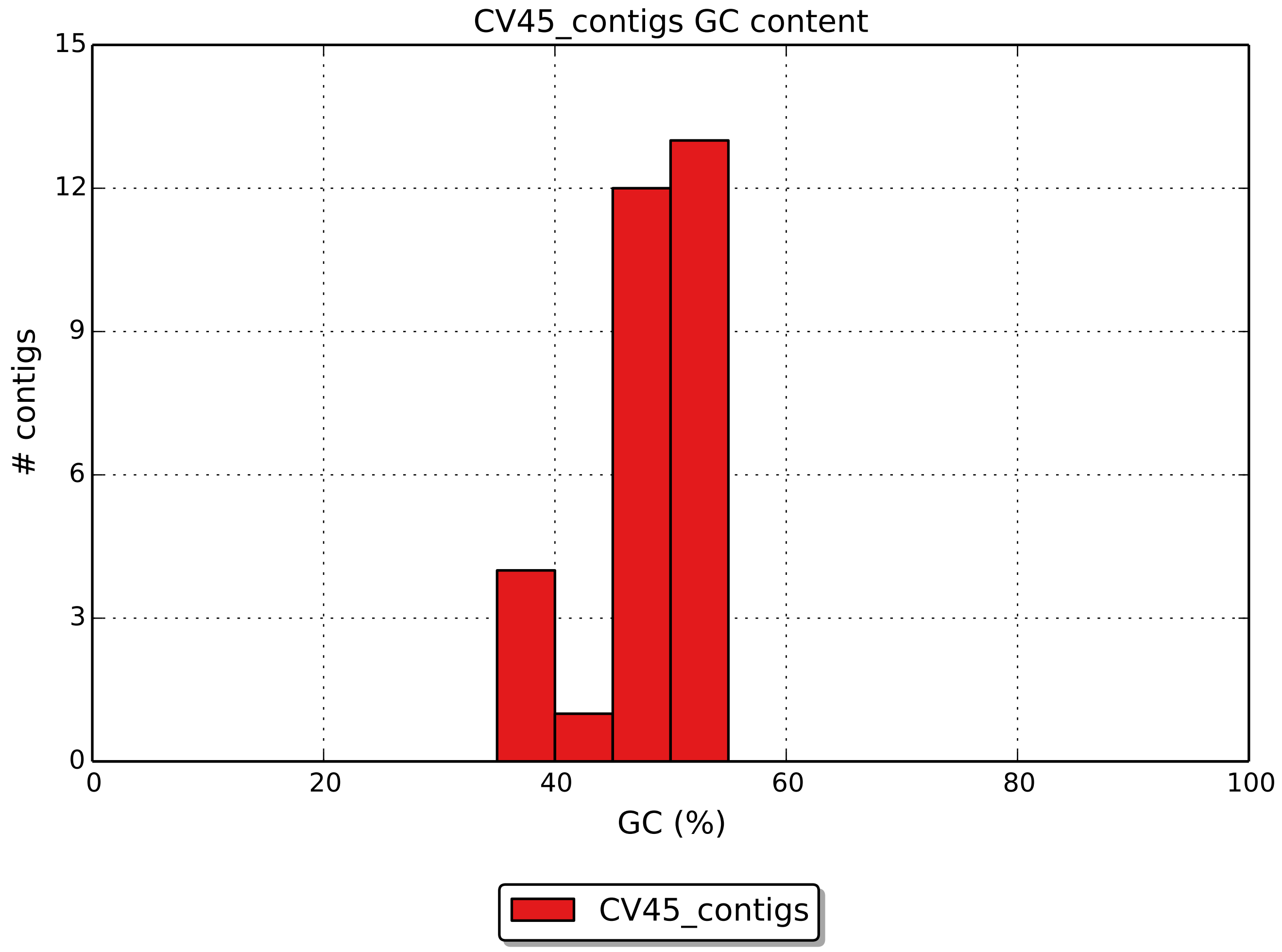

CV45_contigs GC content
contigs
GC (%)
0
3
6
9
12
15
0
20
40
60
80
100
CV45_contigs

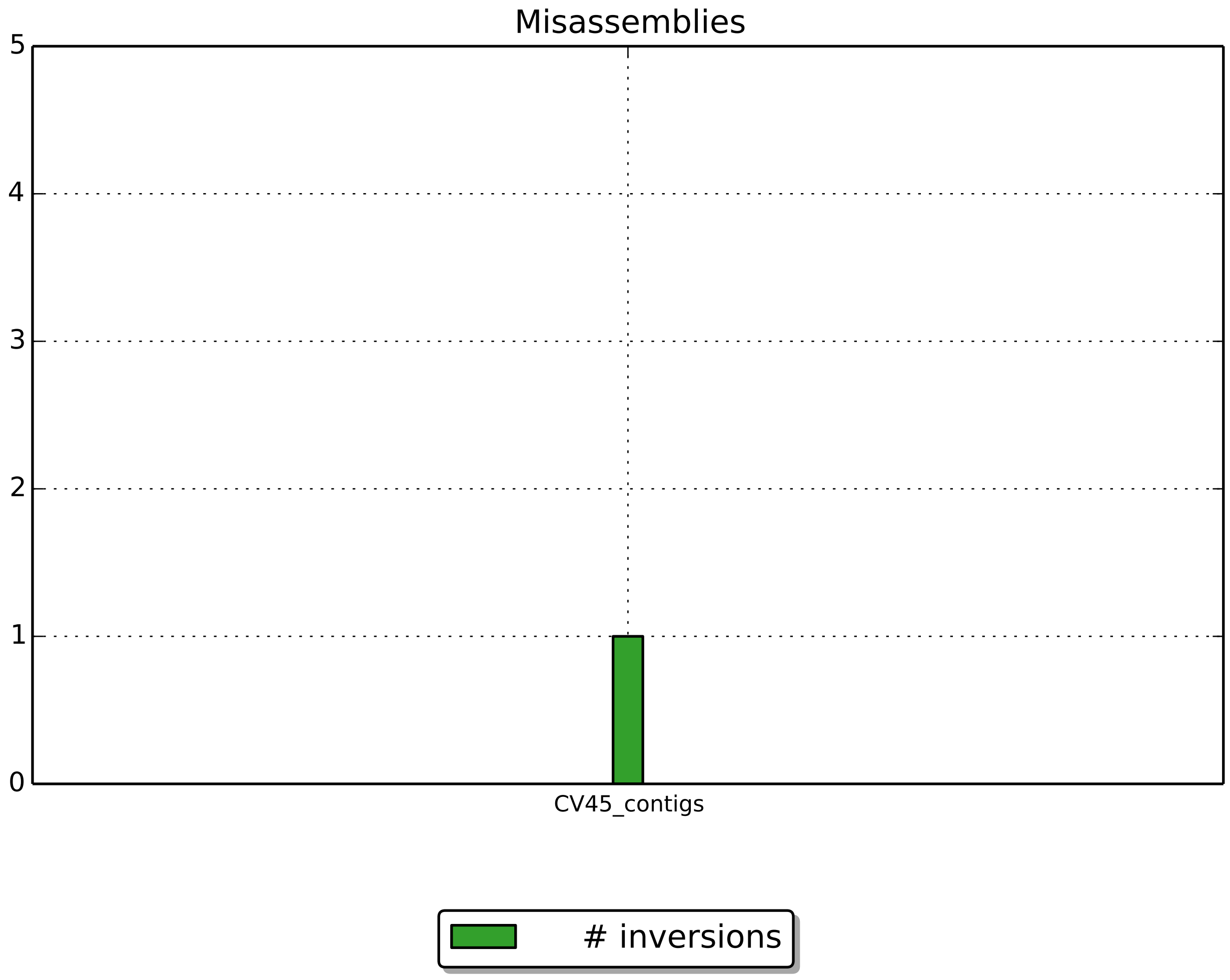

Misassemblies
5
4
3
2
1
0
CV45_contigs
inversions

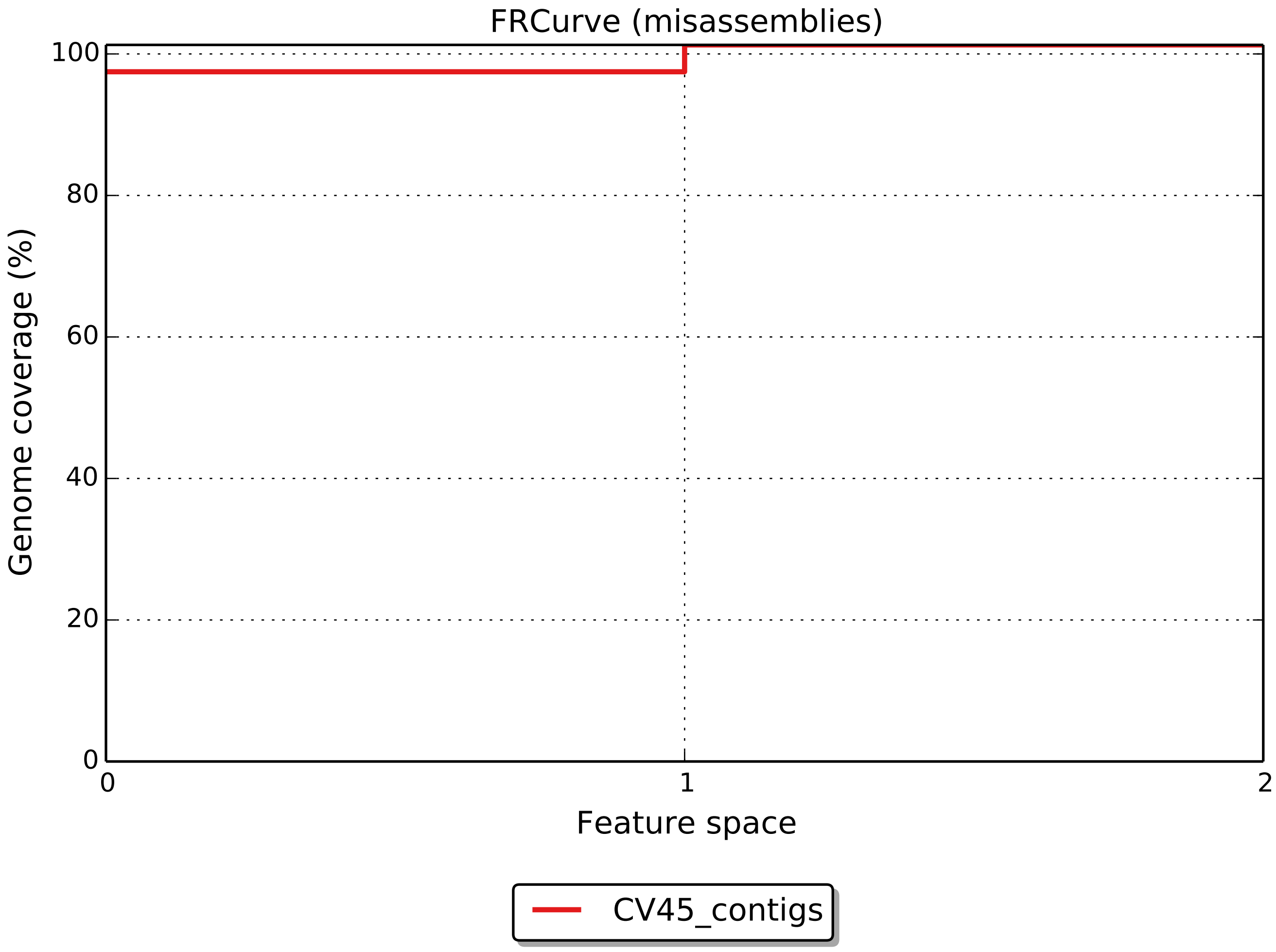

FRCurve (misassemblies)
Genome coverage (%)
100
80
60
40
20
0
0
1
2
Feature space
CV45_contigs

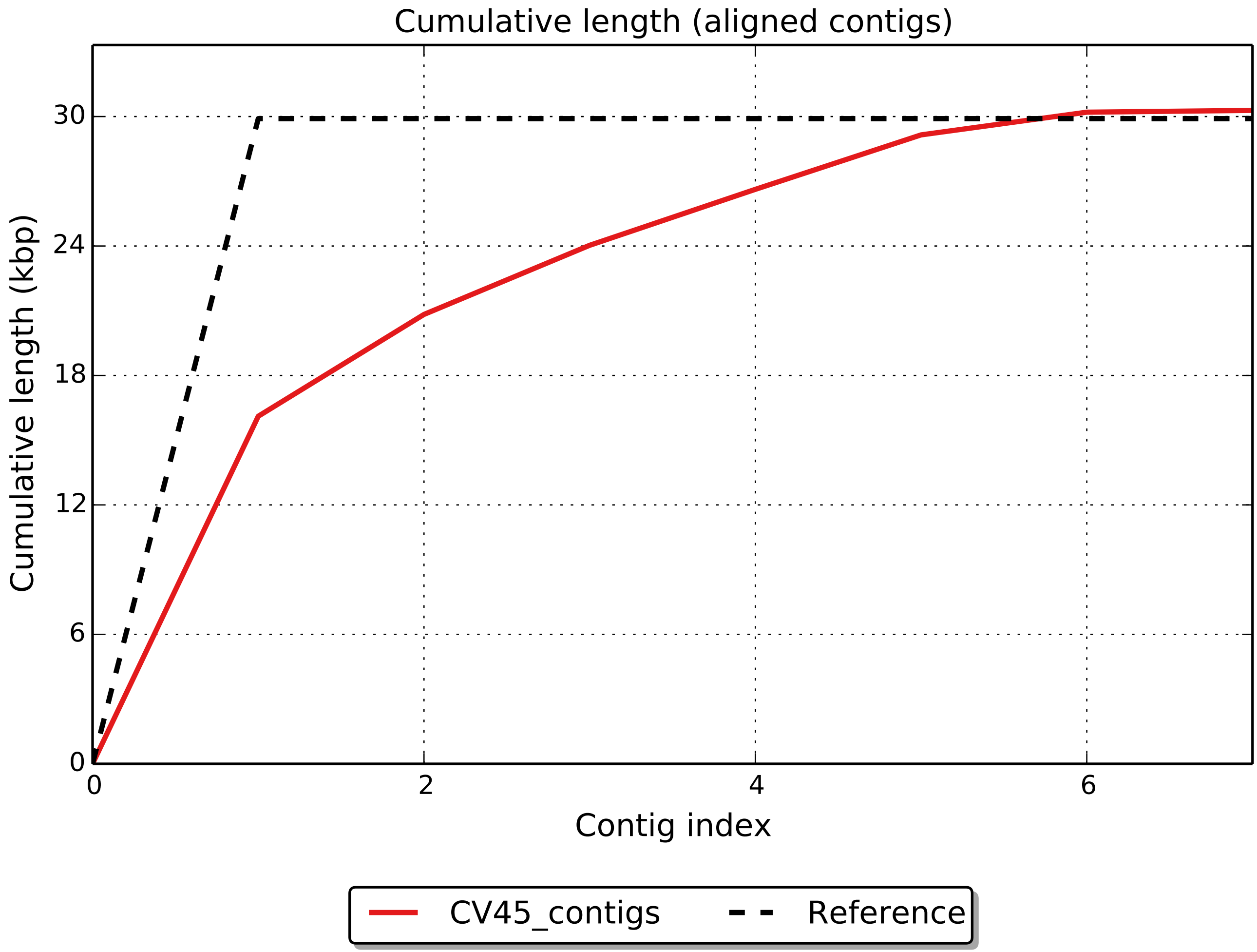

Cumulative length (aligned contigs)
Cumulative length (kbp)
0
6
12
18
24
30
0
2
4
6
Contig index
CV45_contigs
Reference

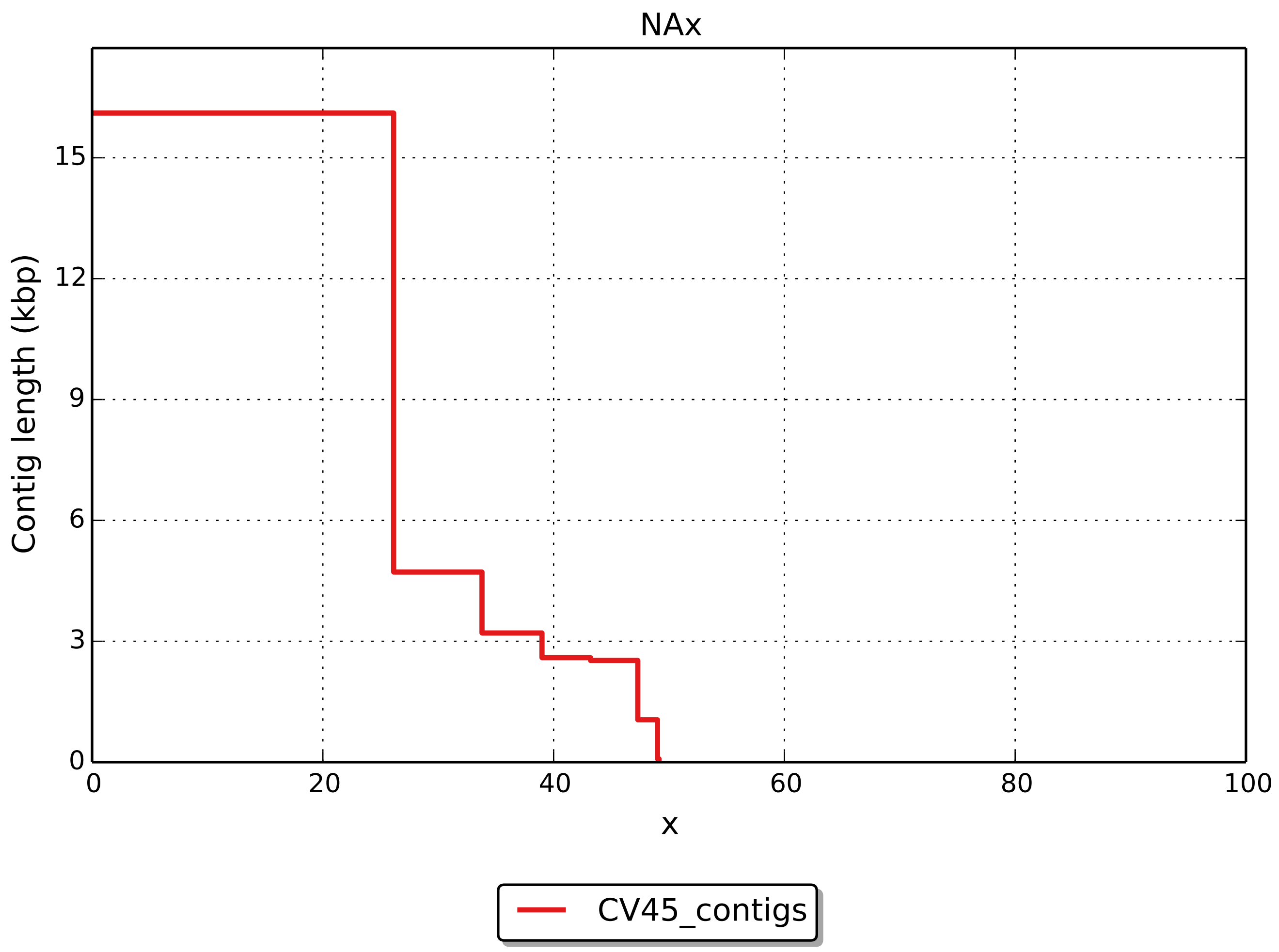

NAx
Contig length (kbp)
0
3
6
9
12
15
0
20
40
60
80
100
x
CV45_contigs

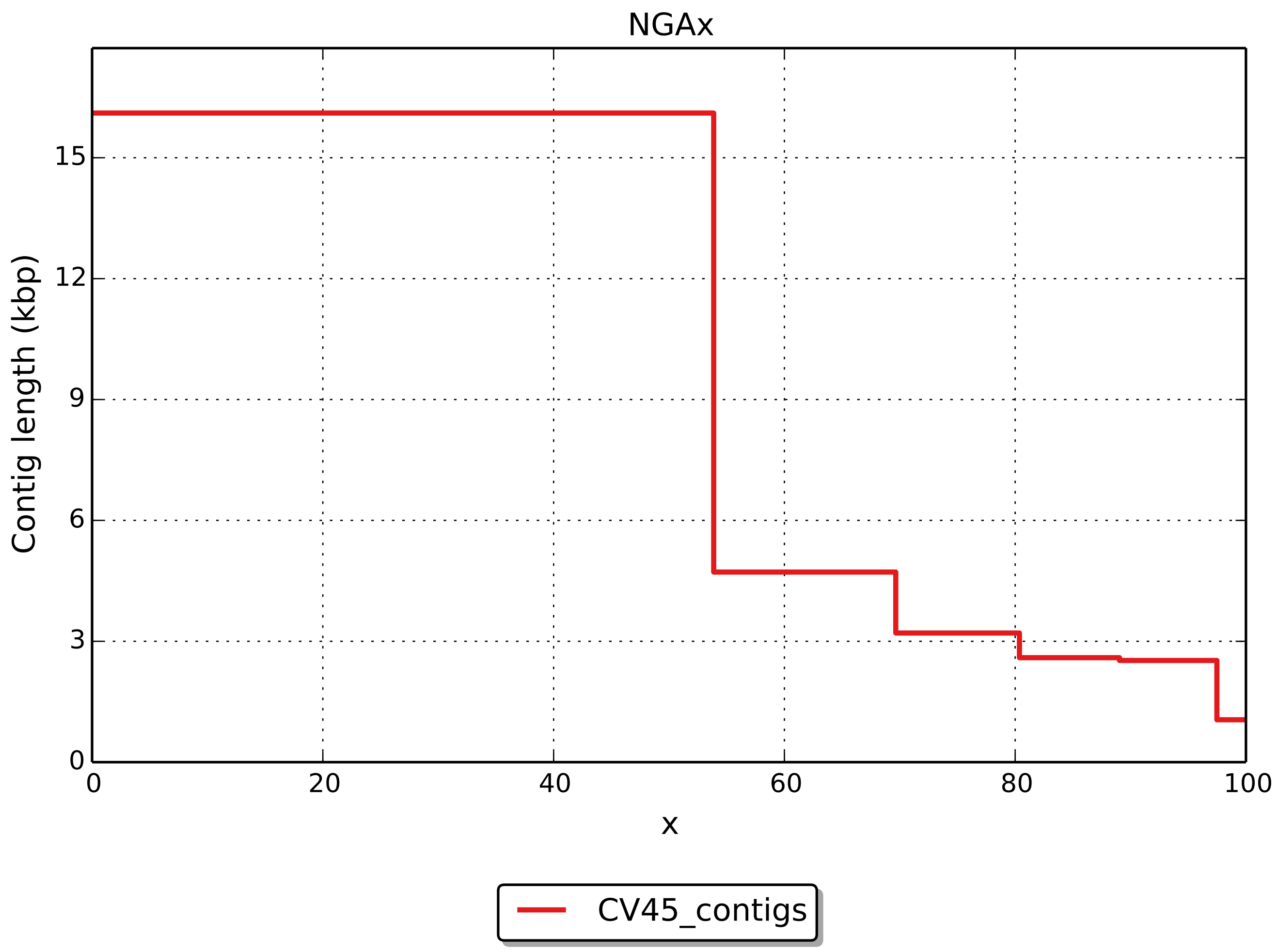

NGAx
Contig length (kbp)
0
3
6
9
12
15
0
20
40
60
80
100
x
CV45_contigs

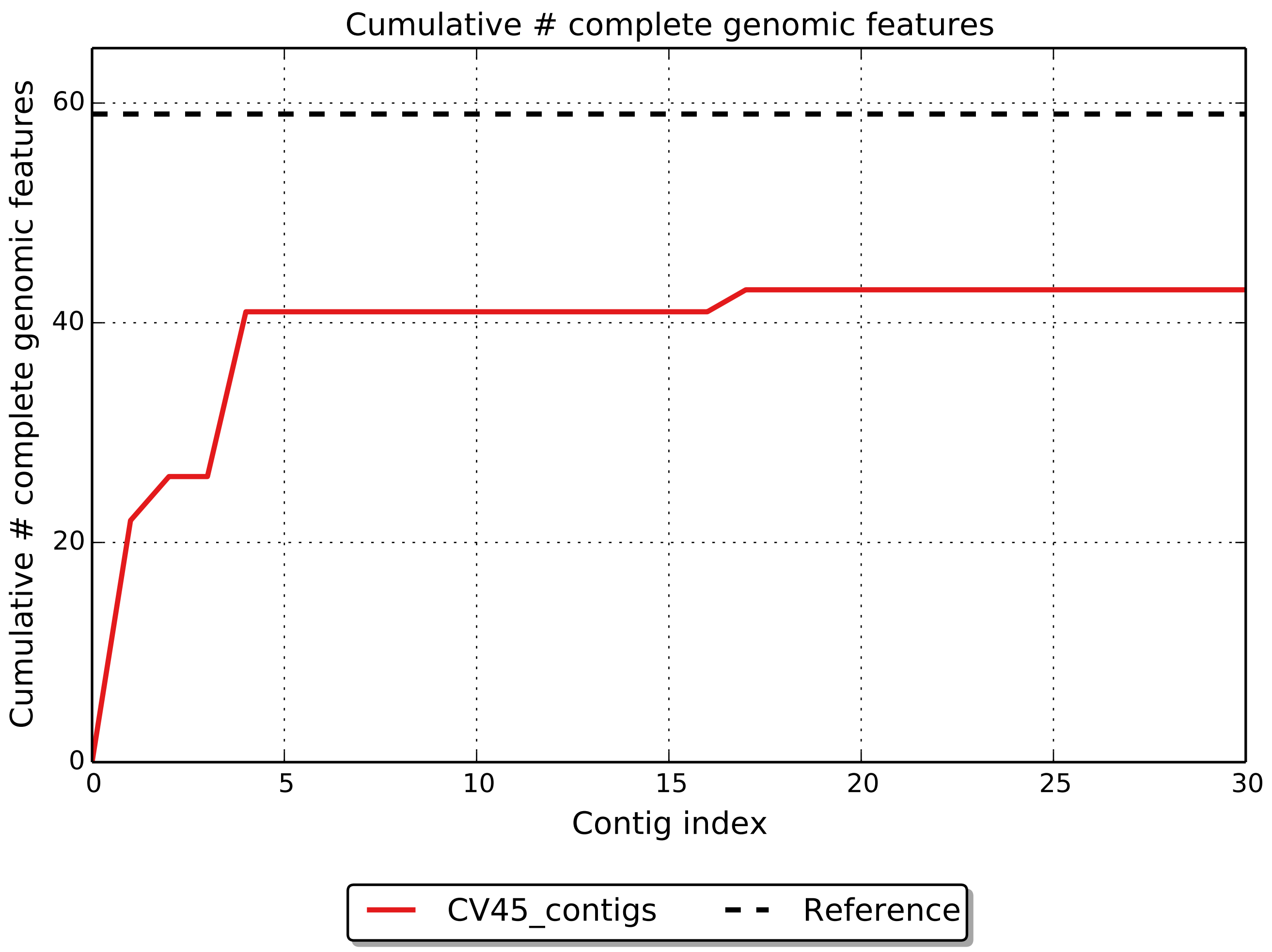
Cumulative # complete genomic features
Cumulative # complete genomic features
Contig index
0
20
40
60
0
5
10
15
20
25
30
CV45_contigs
Reference

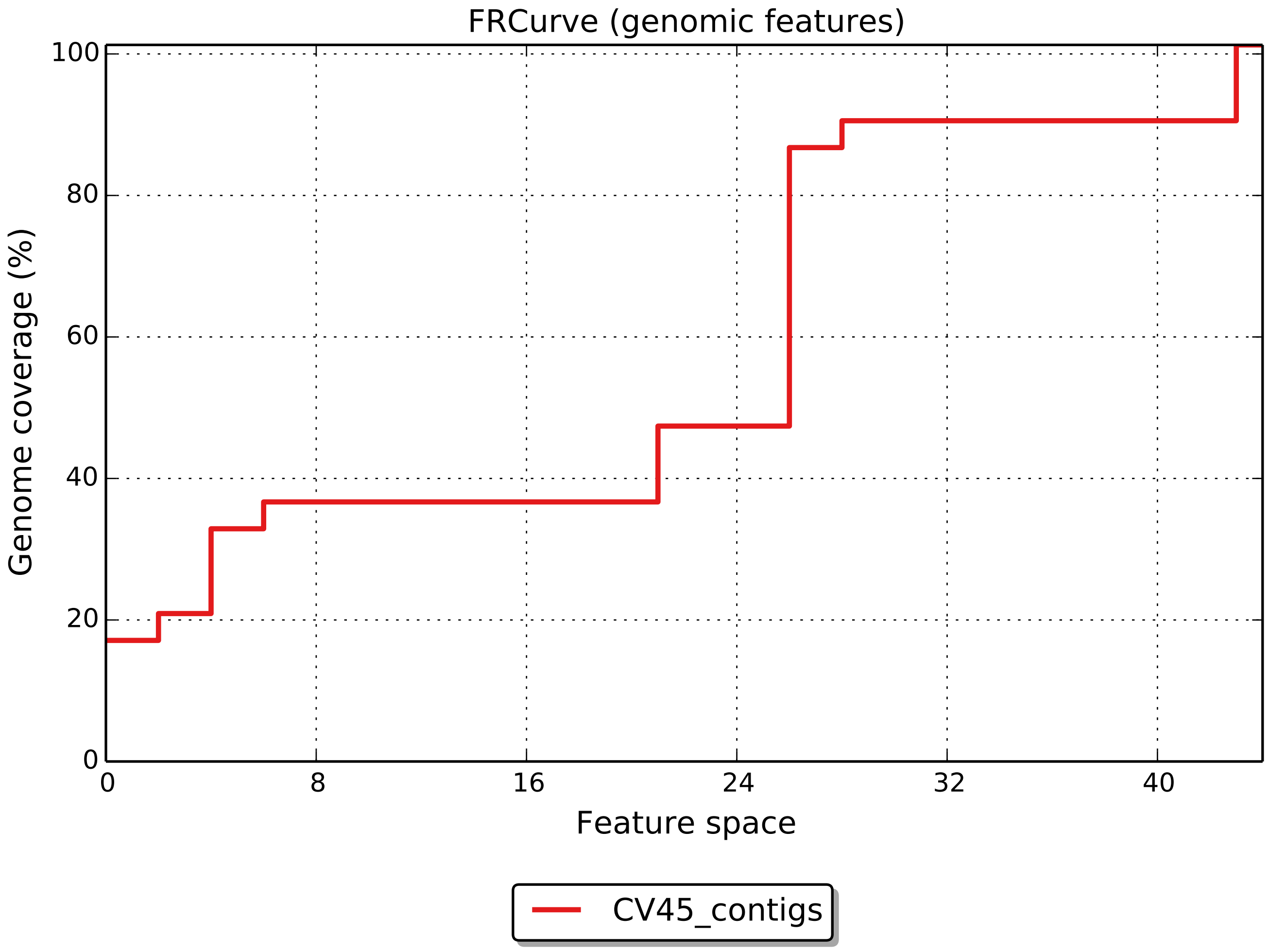

FRCurve (genomic features)
Genome coverage (%)
0
20
40
60
80
100
0
8
16
24
32
40
Feature space
CV45_contigs
