# Supplementary material for: VGEA: an RNA viral assembly toolkit
Source: PeerJ. 2021 Sep 6;9:e12129. doi: 10.7717/peerj.12129 (PMC8428259; doi:10.7717/peerj.12129)
Supplement: Supplemental Information 2 [file peerj-09-12129-s002.zip › Supplementary_File_2/Performance_Comparison_Assembly_Pipelines_Table_2/CV145/vgea/quast_results/results_2021_05_13_23_09_24/report.pdf]

Report

	CV145_contigs
# contigs (>= 0 bp)	28
# contigs (>= 1000 bp)	20
# contigs (>= 5000 bp)	1
# contigs (>= 10000 bp)	0
# contigs (>= 25000 bp)	0
# contigs (>= 50000 bp)	0
Total length (>= 0 bp)	51614
Total length (>= 1000 bp)	44724
Total length (>= 5000 bp)	6807
Total length (>= 10000 bp)	0
Total length (>= 25000 bp)	0
Total length (>= 50000 bp)	0
# contigs	28
Largest contig	6807
Total length	51614
Reference length	29903
GC (%)	45.65
Reference GC (%)	37.97
N50	2049
NG50	4214
N75	1213
NG75	2413
L50	7
LG50	3
L75	15
LG75	6
# misassemblies	0
# misassembled contigs	0
Misassembled contigs length	0
# local misassemblies	0
# scaffold gap ext. mis.	0
# scaffold gap loc. mis.	0
# unaligned mis. contigs	0
# unaligned contigs	22 + 0 part
Unaligned length	29418
Genome fraction (%)	73.093
Duplication ratio	1.016
# N's per 100 kbp	0.00
# mismatches per 100 kbp	64.05
# indels per 100 kbp	0.00
# genomic features	29 + 17 part
Largest alignment	6734
Total aligned length	21984
NGA50	4214
LGA50	3

All statistics are based on contigs of size >= 500 bp, unless otherwise noted (e.g., "# contigs (>= 0 bp)" and "Total length (>= 0 bp)" include all contigs).

Misassemblies report

	CV145_contigs
# misassemblies	0
# contig misassemblies	0
# c. relocations	0
# c. translocations	0
# c. inversions	0
# scaffold misassemblies	0
# s. relocations	0
# s. translocations	0
# s. inversions	0
# misassembled contigs	0
Misassembled contigs length	0
# local misassemblies	0
# scaffold gap ext. mis.	0
# scaffold gap loc. mis.	0
# unaligned mis. contigs	0
# mismatches	14
# indels	0
# indels (<= 5 bp)	0
# indels (> 5 bp)	0
Indels length	0

All statistics are based on contigs of size >= 500 bp, unless otherwise noted (e.g., "# contigs (>= 0 bp)" and "Total length (>= 0 bp)" include all contigs).

Unaligned report

	CV145_contigs
# fully unaligned contigs	22
Fully unaligned length	29418
# partially unaligned contigs	0
Partially unaligned length	0
# N's	0

All statistics are based on contigs of size >= 500 bp, unless otherwise noted (e.g., "# contigs (>= 0 bp)" and "Total length (>= 0 bp)" include all contigs).

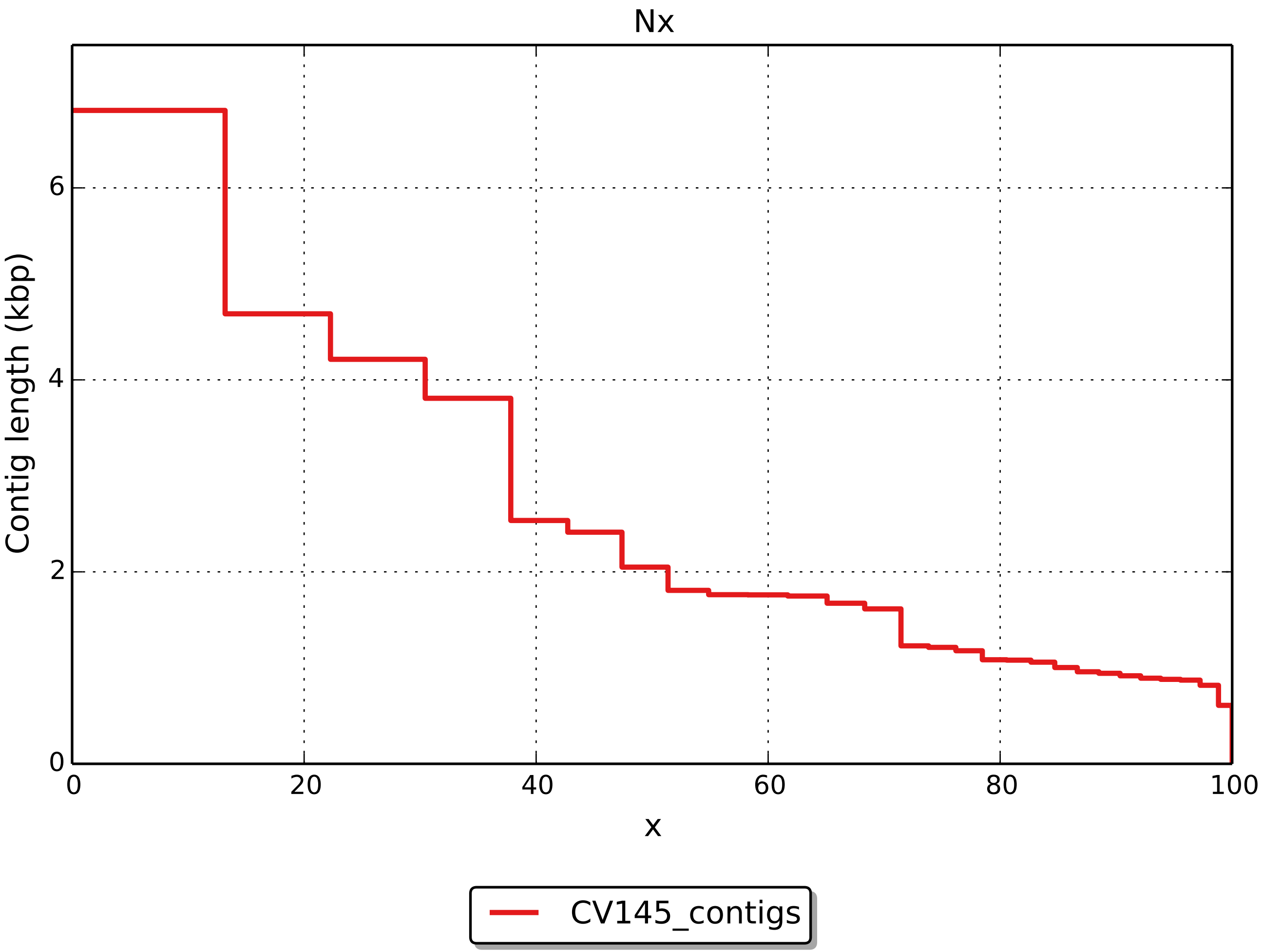

Nx
Contig length (kbp)
0
2
4
6
0
20
40
60
80
100
x
CV145_contigs

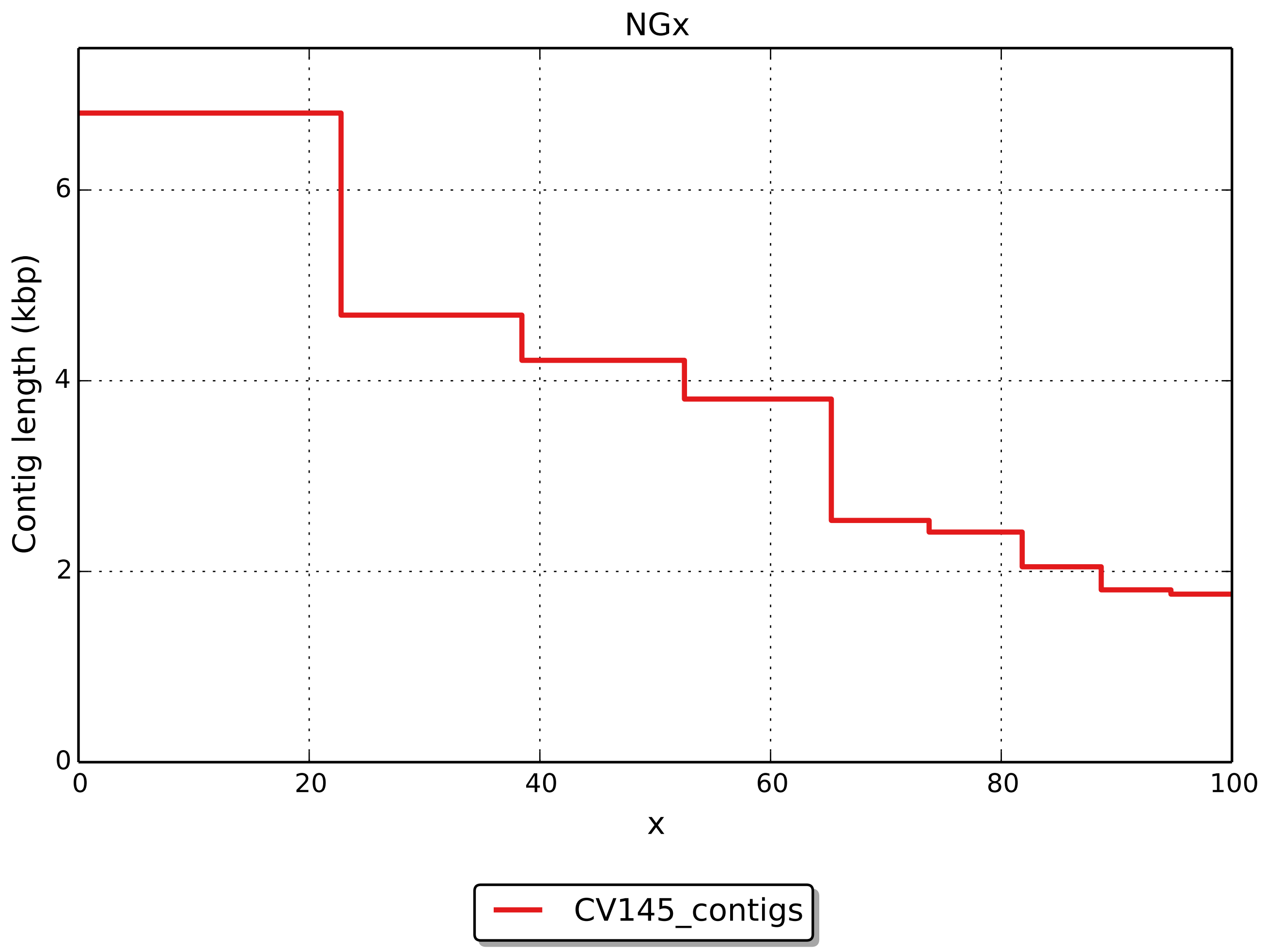
NGx
Contig length (kbp)
0
2
4
6
0
20
40
60
80
100
x
CV145_contigs

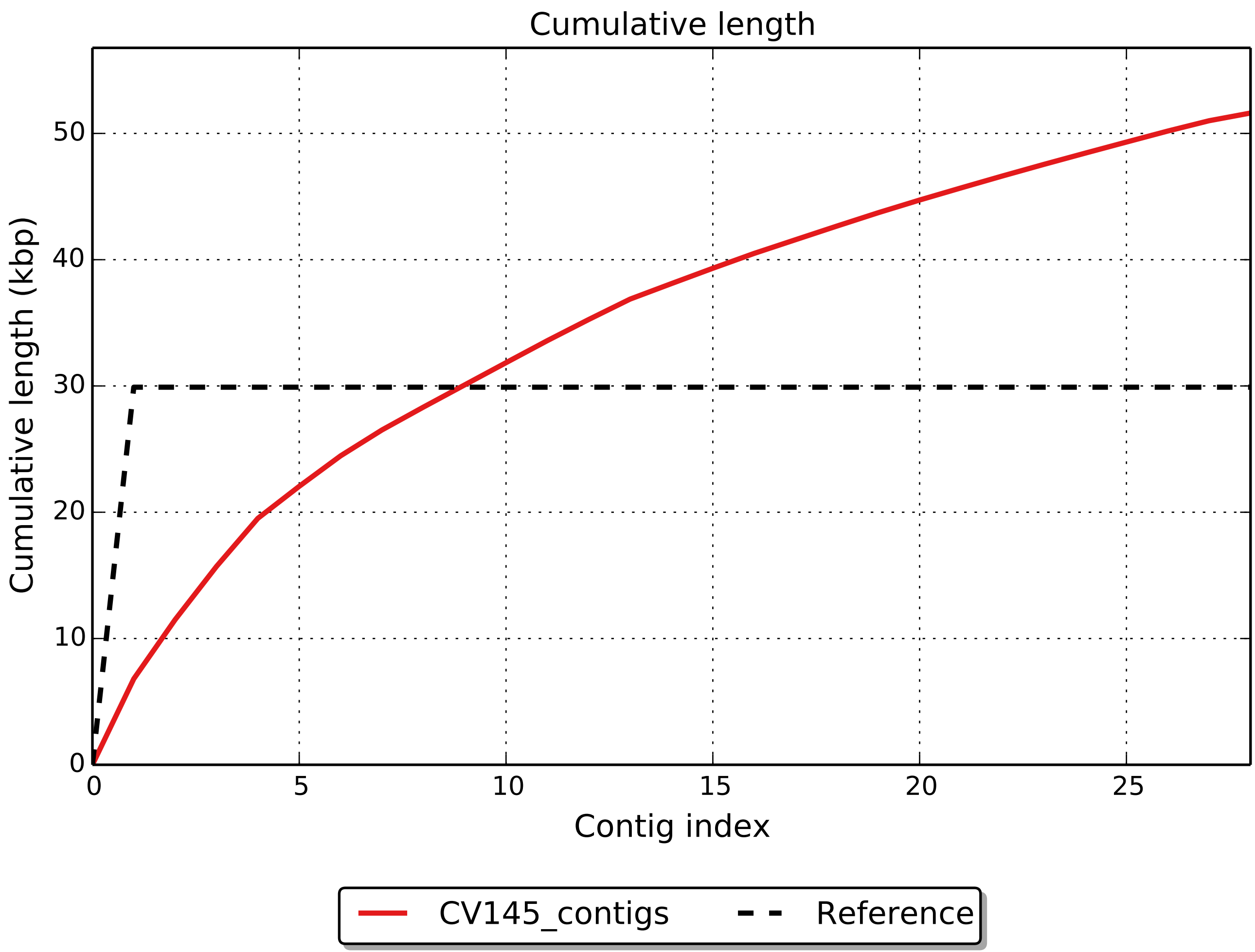
Cumulative length
Cumulative length (kbp)
0
10
20
30
40
50
0
5
10
15
20
25
Contig index
CV145_contigs
Reference

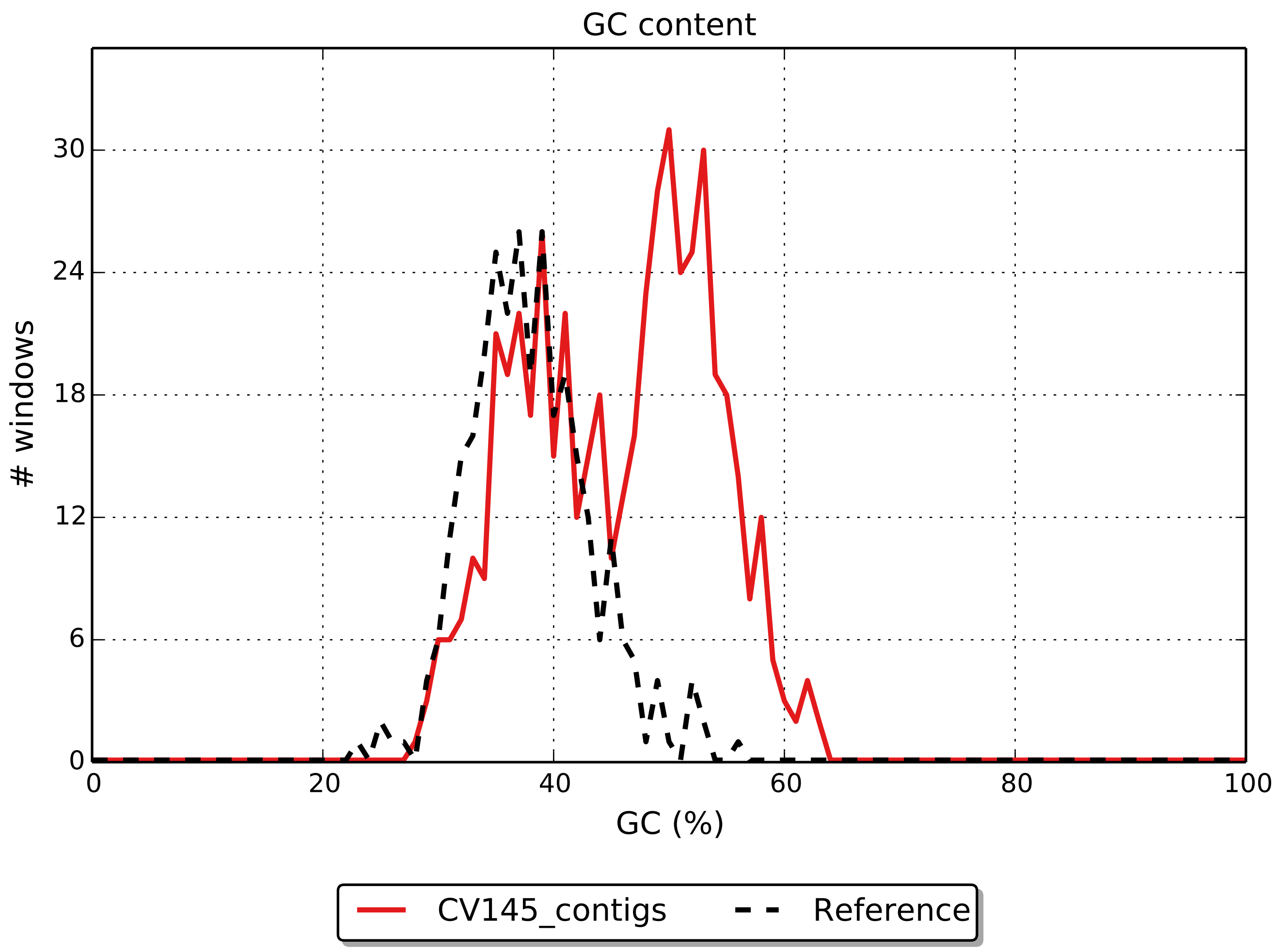

GC content
30
24
18
12
6
0
0
20
40
60
80
100
windows
GC (%)
CV145_contigs
Reference

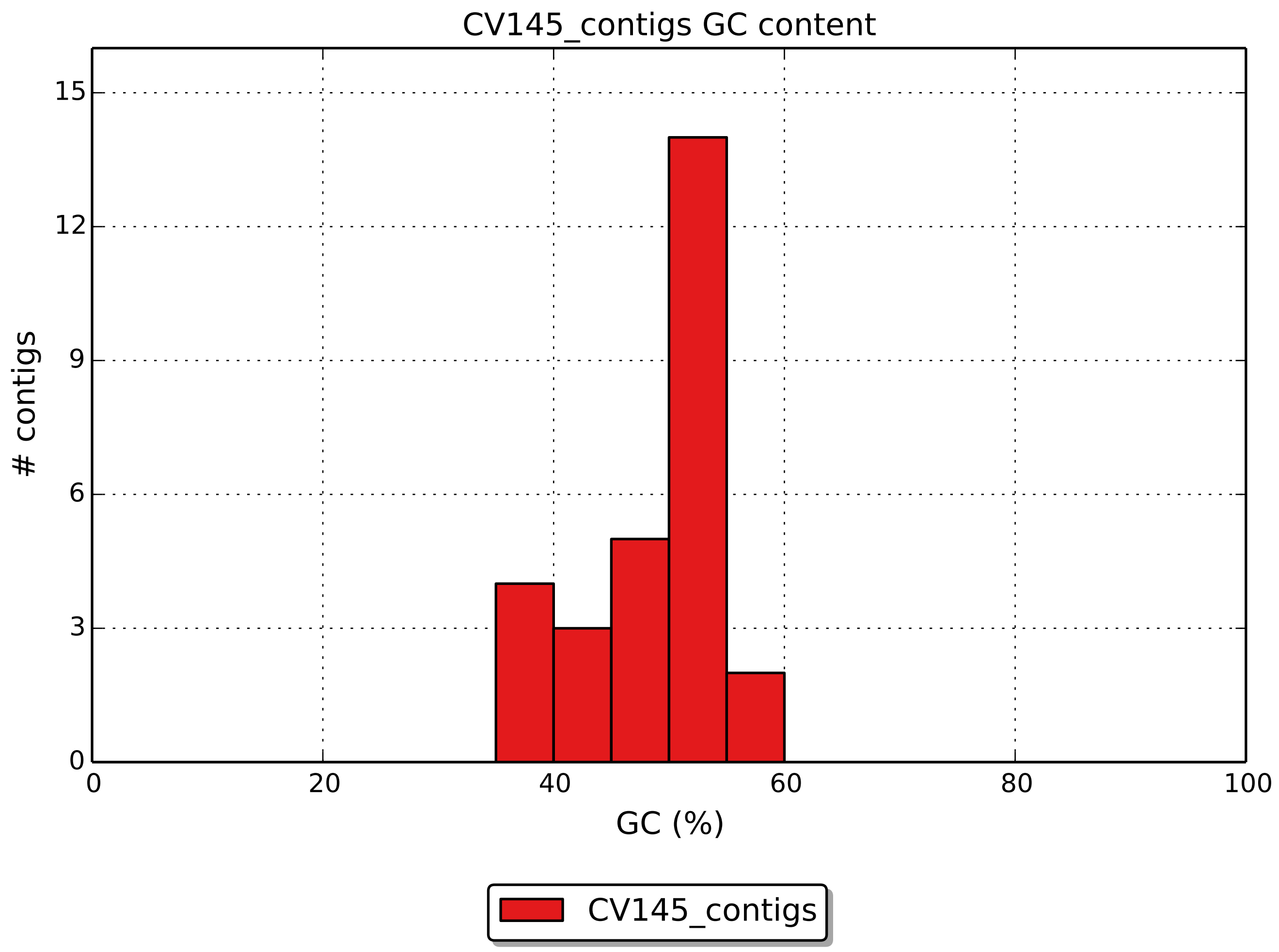
CV145_contigs GC content
contigs
GC (%)
0
3
6
9
12
15
0
20
40
60
80
100
CV145_contigs

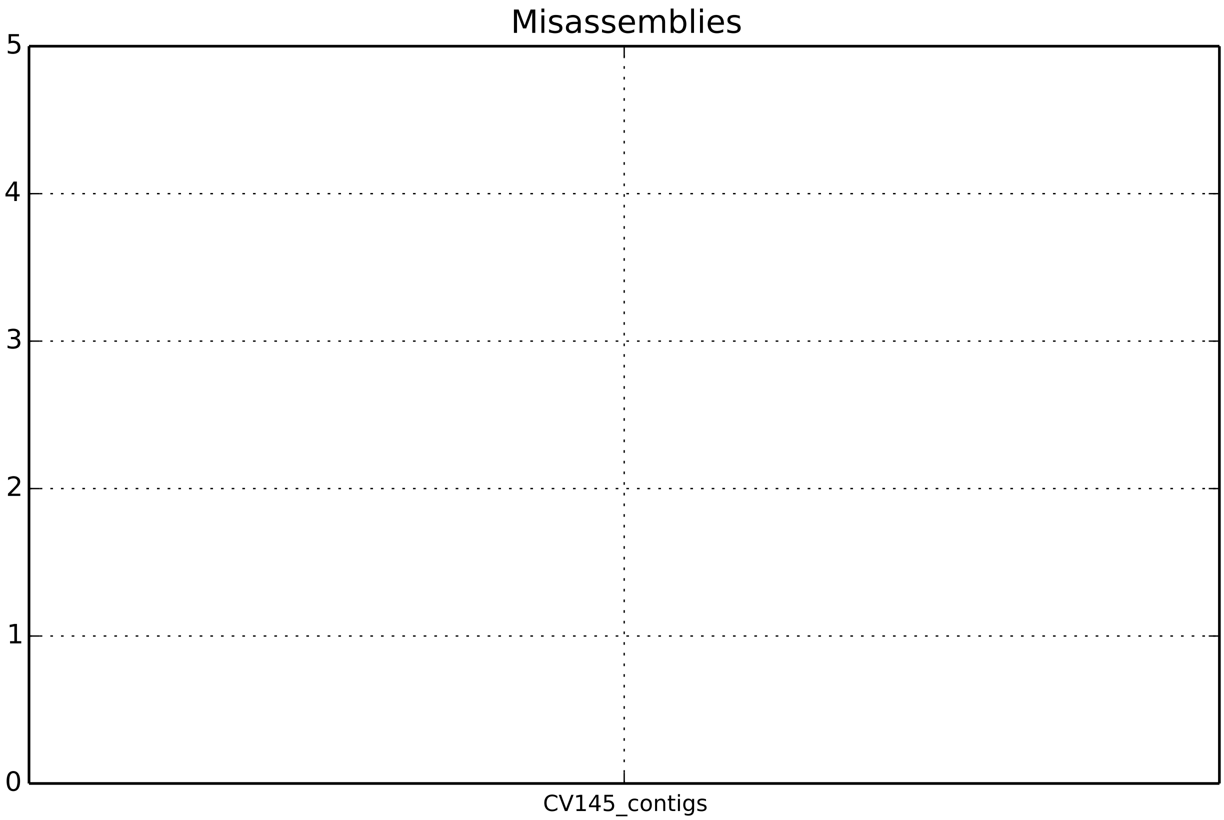
Misassemblies
5
4
3
2
1
0
CV145_contigs

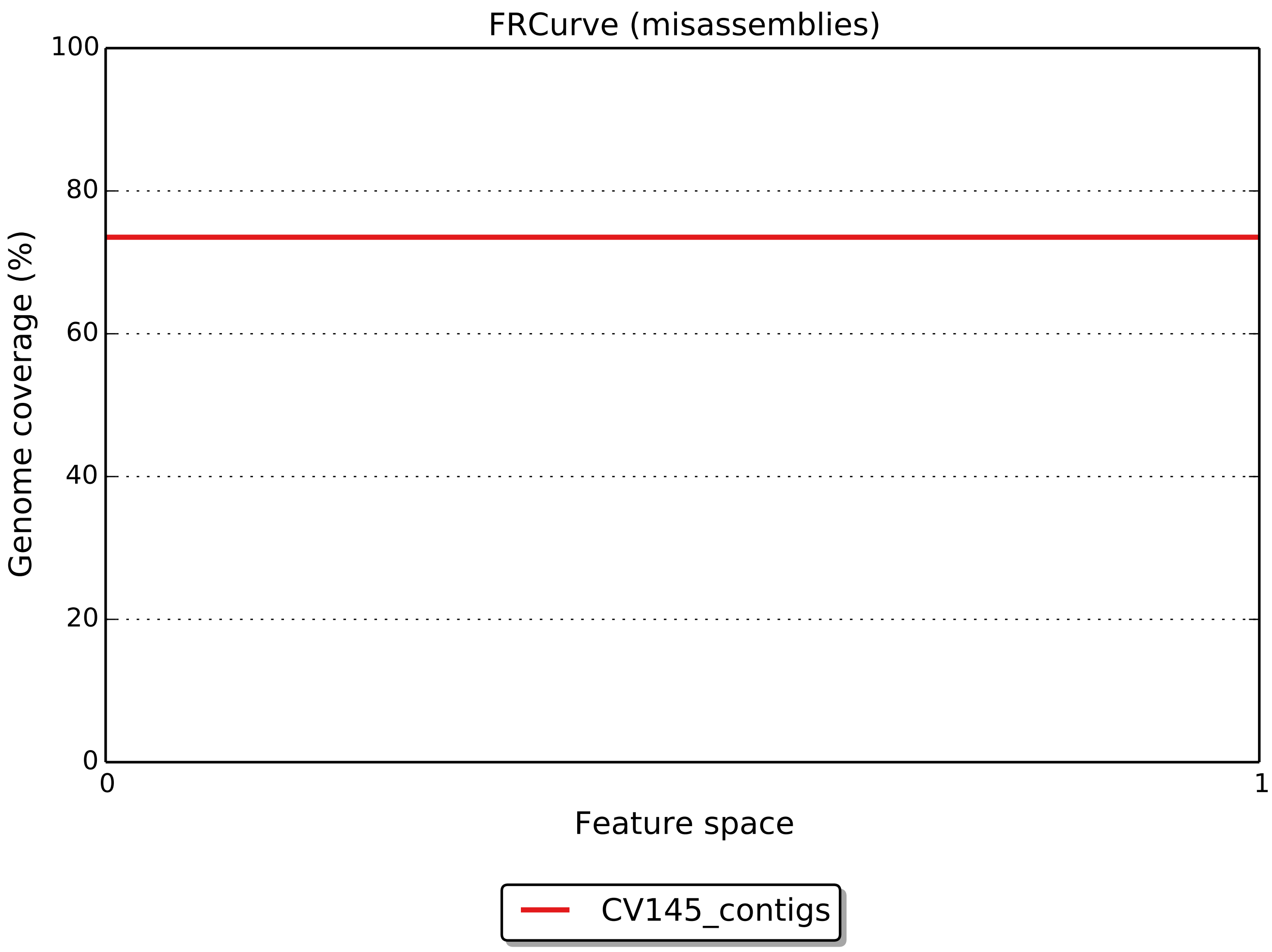
FRCurve (misassemblies)
Genome coverage (%)
100
80
60
40
20
0
0
1
Feature space
CV145_contigs

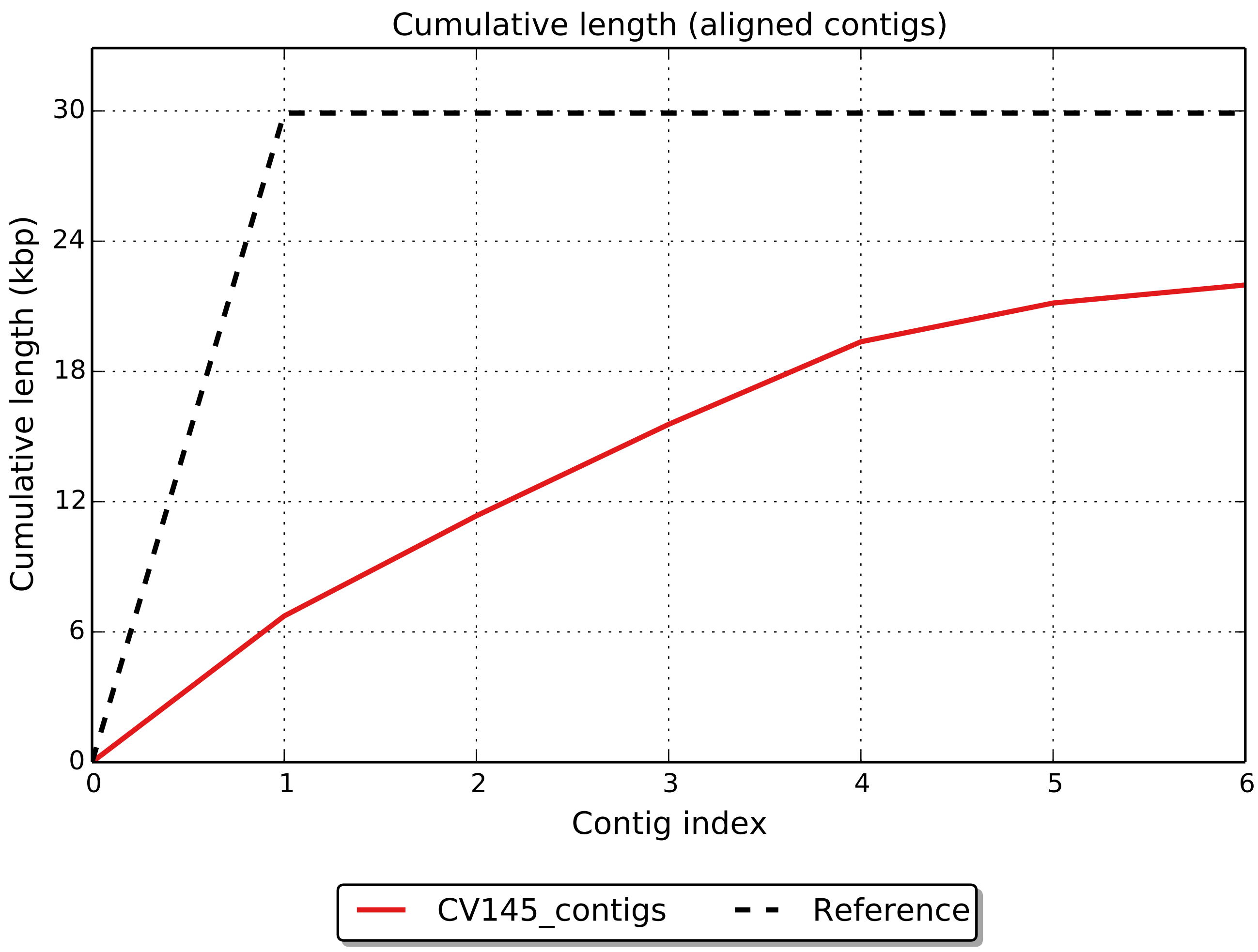

Cumulative length (aligned contigs)
Cumulative length (kbp)
0
6
12
18
24
30
0
1
2
3
4
5
6
Contig index
CV145_contigs
Reference

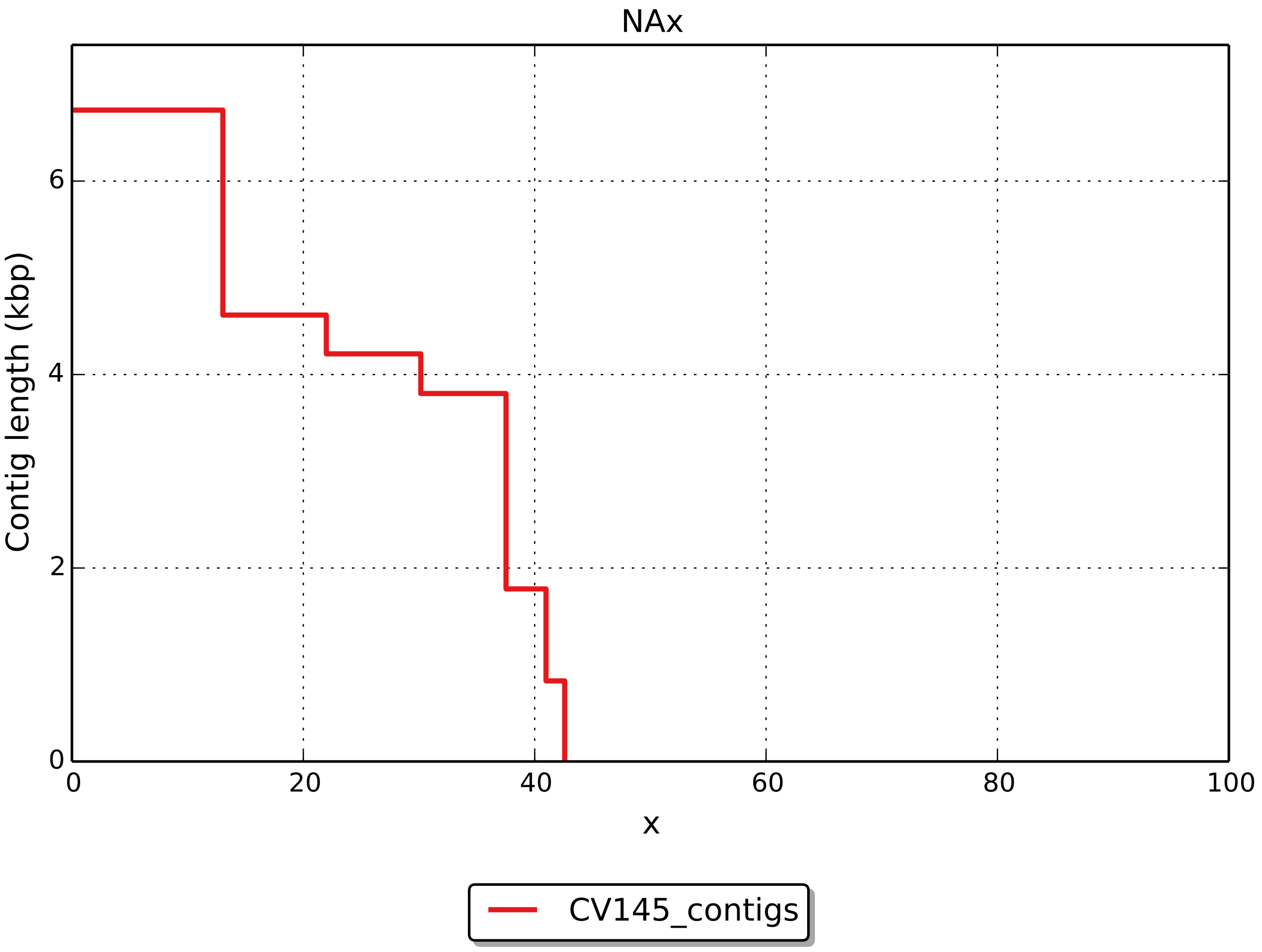

NAx
Contig length (kbp)
0
2
4
6
0
20
40
60
80
100
x
CV145_contigs

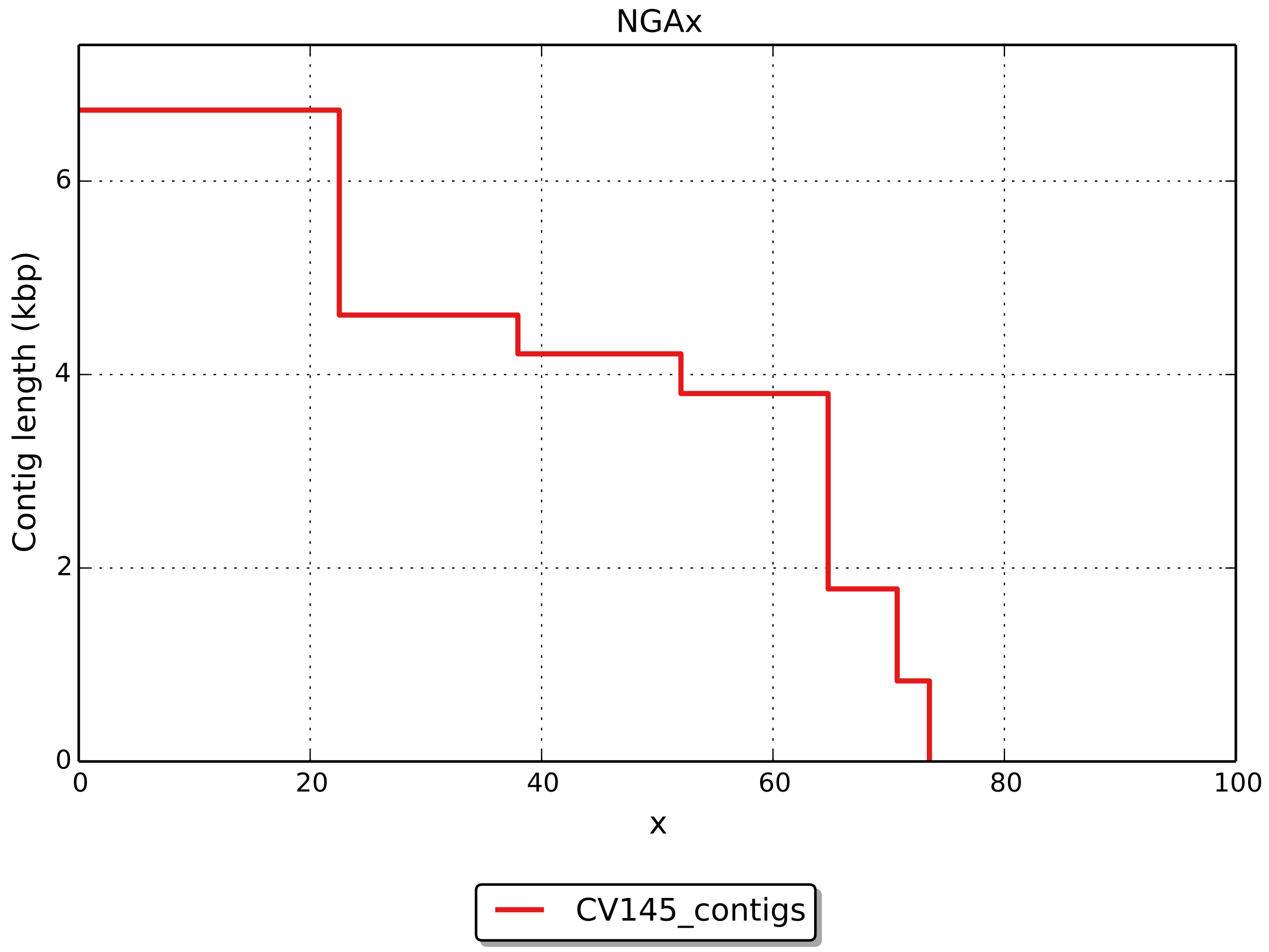
NGAx
Contig length (kbp)
0
2
4
6
0
20
40
60
80
100
x
CV145_contigs

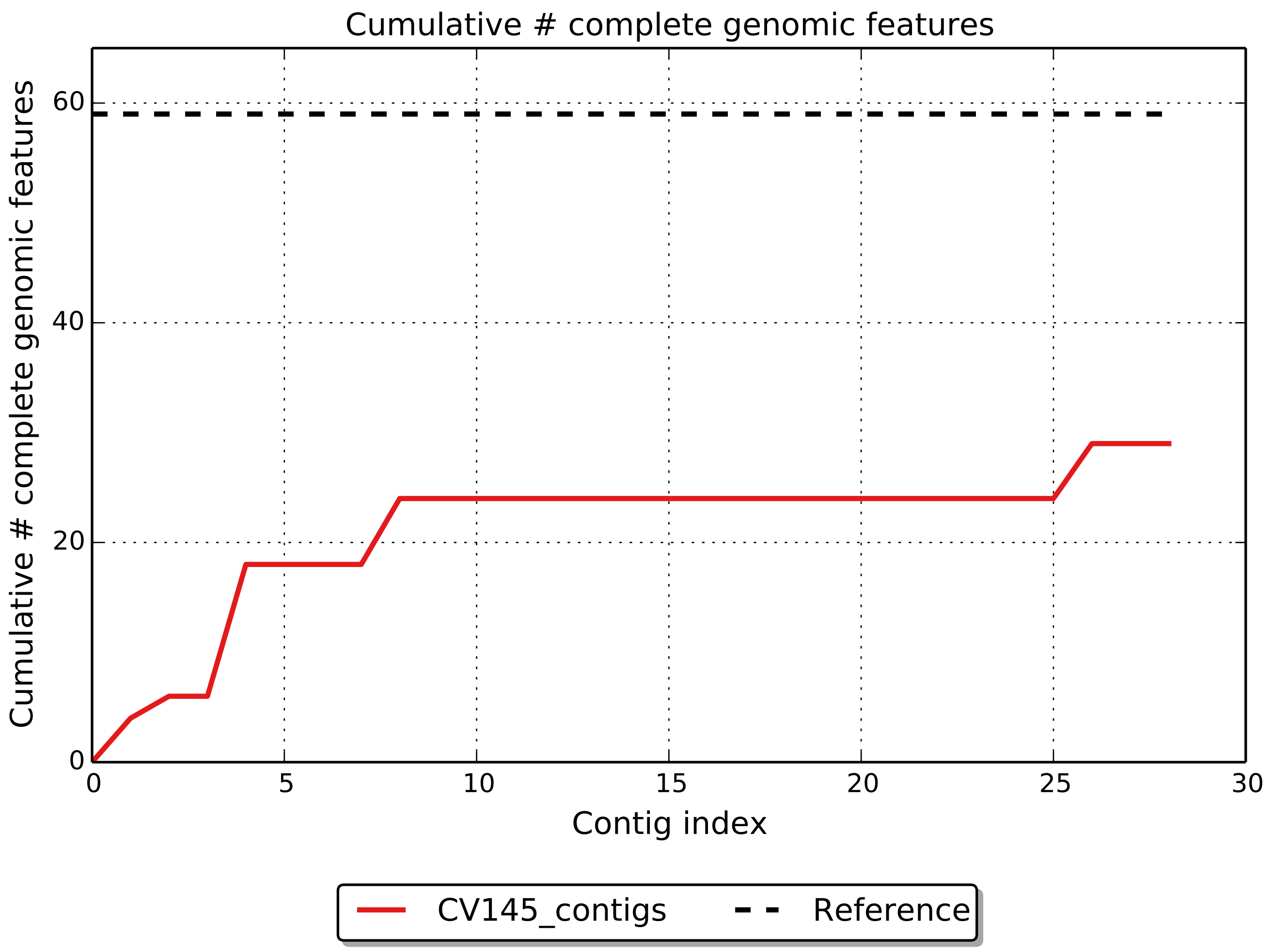
Cumulative # complete genomic features
Cumulative # complete genomic features
Contig index
0
5
10
15
20
25
30
0
20
40
60
CV145_contigs
Reference

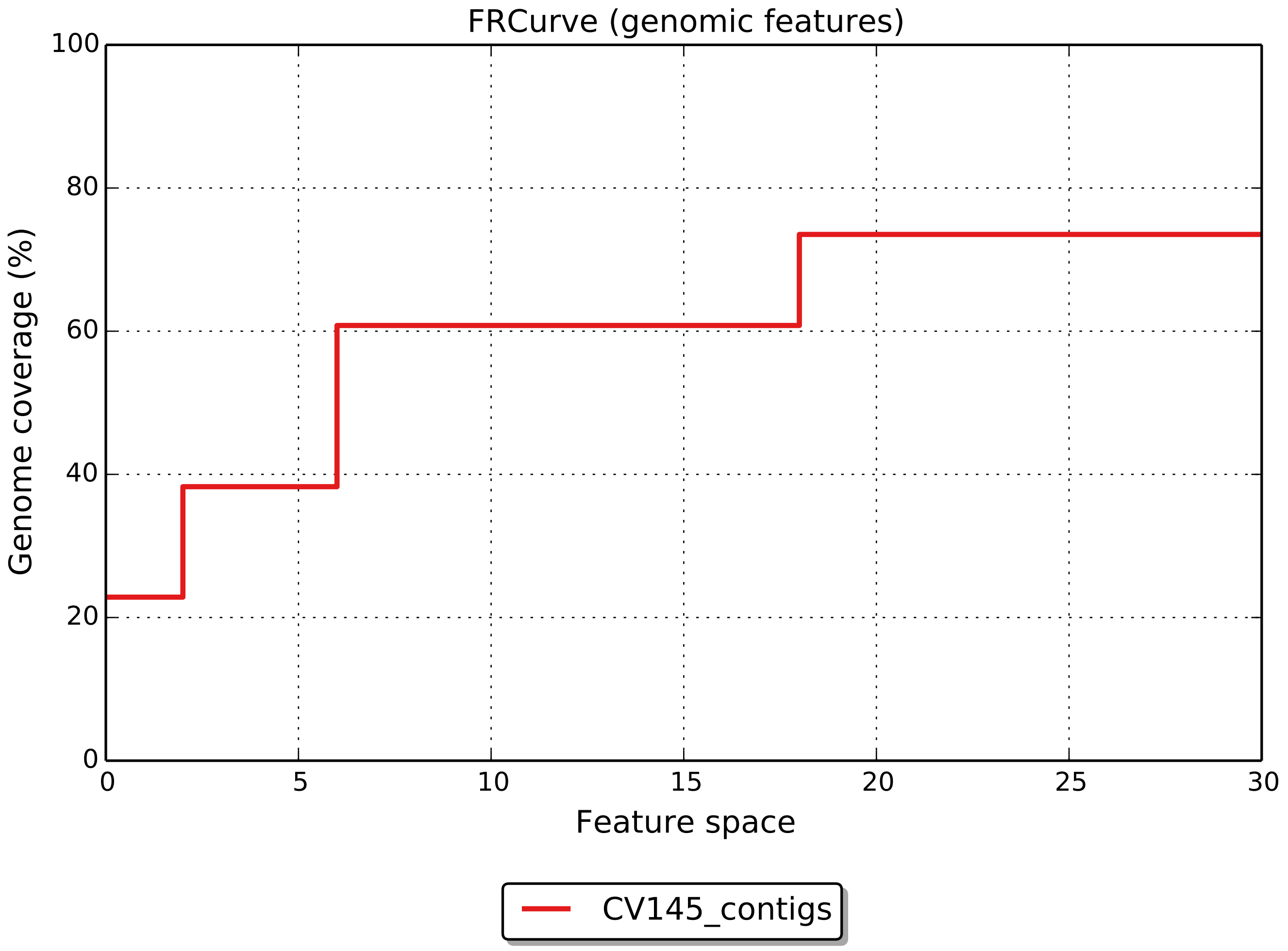
FRCurve (genomic features)
Genome coverage (%)
Feature space
0
20
40
60
80
100
0
5
10
15
20
25
30
CV145_contigs
